# Green Knoevenagel condensation of indolin-2-one derivatives using undivided electrochemical cell

**DOI:** 10.1016/j.mex.2026.104145

**Published:** 2026-09-06

**Authors:** Robby Gus Mahardika, Ade Danova, Warinthorn Chavasiri, Elvira Hermawati, Anita Alni

**Affiliations:** a Doctoral Program of Chemistry, Faculty of Mathematics and Natural Sciences, Institut Teknologi Bandung, Jl. Ganesha No. 10, Bandung, West Java, 40132, Indonesia; b Organic Chemistry Division, Faculty of Mathematics and Natural Sciences, Institut Teknologi Bandung, Jl. Ganesha No. 10, Bandung, West Java, 40132, Indonesia; c Center of Excellence in Natural Products Chemistry, Department of Chemistry, Faculty of Science, Chulalongkorn University, Bangkok, Thailand

**Keywords:** Knoevenagel condensation, Electrosynthesis, Green chemistry, Sustainable synthesis, Indoline-2-one

## Abstract

Electrochemical Knoevenagel condensation has emerged as an attractive alternative to conventional condensation methods because it avoids the use of stoichiometric oxidants and enables reactions under mild conditions. However, a general electrochemical methodology for the condensation of indolin-2-one derivatives has not been established. Herein, we describe a practical electrochemical method for the Knoevenagel condensation of indolin-2-one derivatives with aromatic aldehydes in an ethanol-water medium using a simple undivided cell. The reaction conditions were systematically optimized by evaluating the supporting electrolyte, solvent composition, and electrolysis parameters. The developed method proceeds at room temperature within a short reaction time and affords the desired products in good to excellent yields. The robustness and general applicability of the methodology were validated across a diverse range of aromatic and heteroaromatic aldehydes as well as indolin-2-one derivatives. The use of a green solvent system, operational simplicity, and broad substrate compatibility make this methodology readily applicable to the synthesis of 3-alkenylindolin-2-one derivatives for synthetic and medicinal chemistry studies.

A practical electrochemical methodology for the Knoevenagel condensation of indolin-2-one derivatives in an ethanol-water medium.

Validated across structurally diverse substrates with short reaction times under mild, room-temperature conditions using a simple undivided electrochemical cell.

## Graphical abstract

**Figure:**
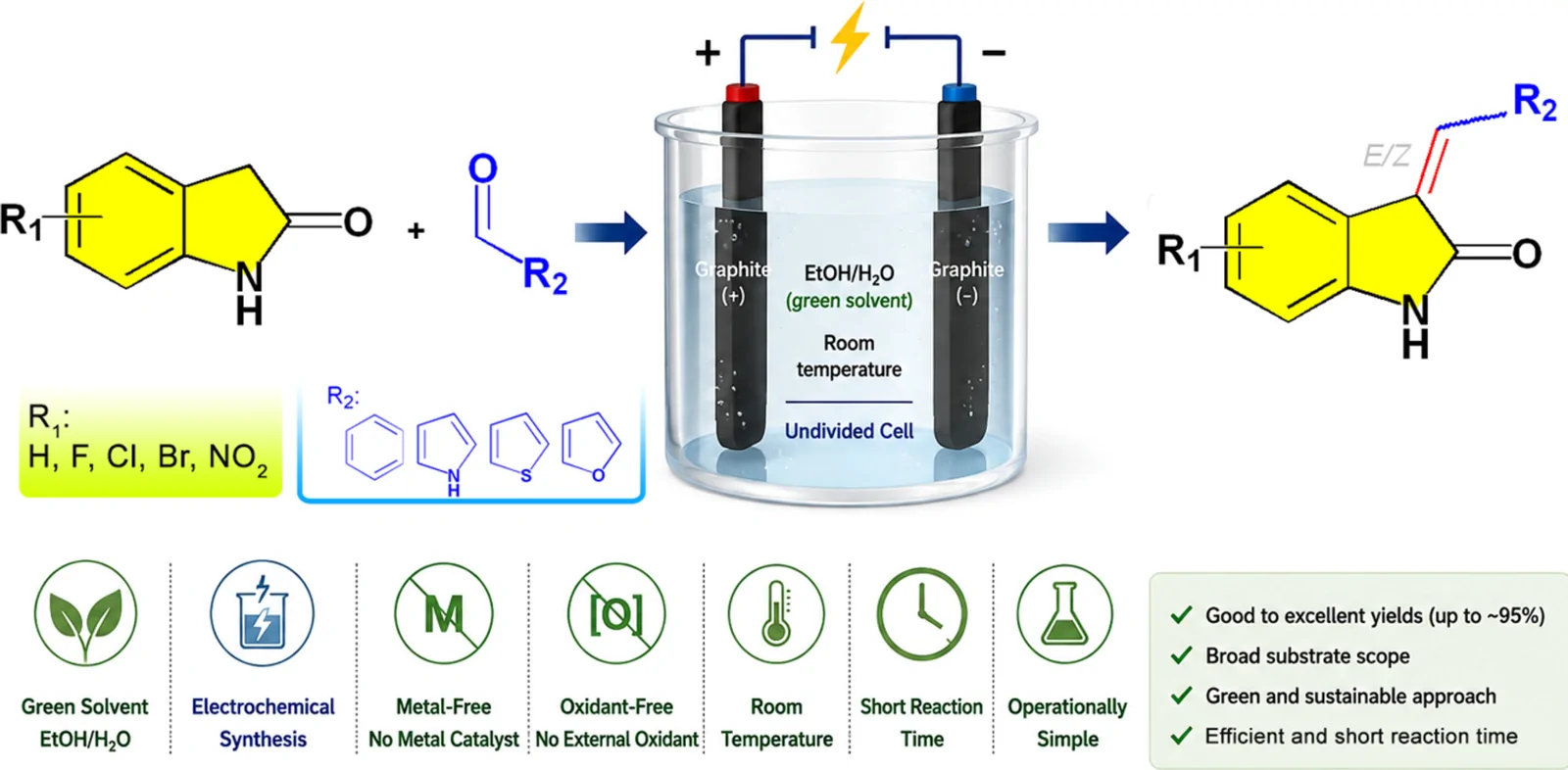

Specifications table

**Table:** 

Subject area	Chemistry
More specific subject area	Organic Synthesis
Name of your method	Electrochemical Knoevenagel Condensation of Indolin-2-one Derivatives
Name and reference of original method	A. Alni, R.G. Mahardika, A. Danova, E. Hermawati, Two-step electrochemically driven synthesis of sunitinib, Org. Biomol. Chem. 23 (2025) 5542–5548. https://doi.org/10.1039/D5OB00377F
Resource availability	Resources are included in the text

## Background

**Fig. 1:**
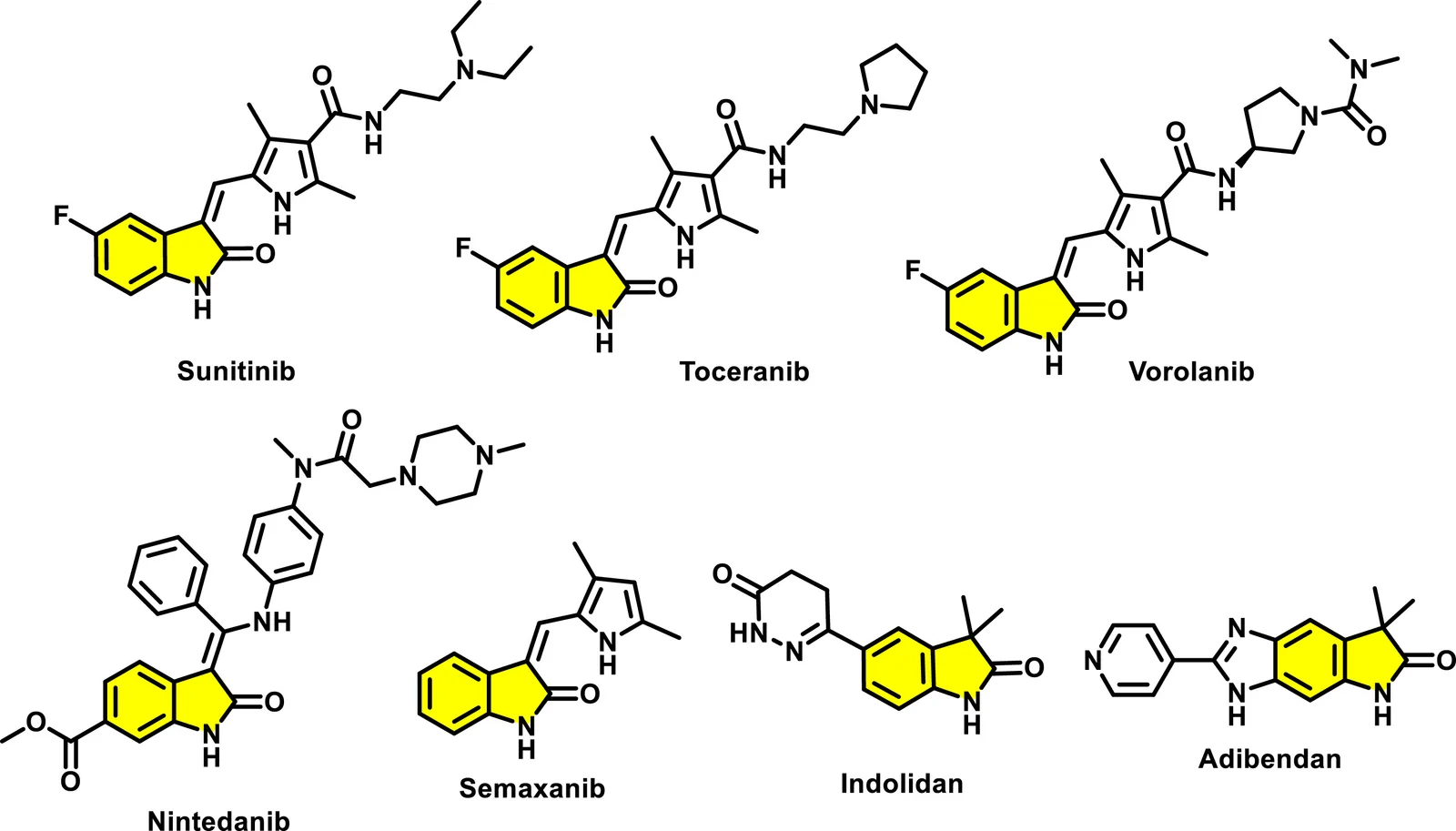
Representative drugs containing the indolin-2-one scaffold.

Indolin-2-one derivatives are a significant category of heterocyclic compounds that are prevalent in natural products [1,2], pharmaceuticals [[3], [4], [5]], and biologically active molecules [4,6]. The indolin-2-one scaffold appears in numerous compounds that exhibit diverse biological activities, including anticancer [[7], [8], [9]], antimicrobial [10], antiviral [11,12], antidiabetics [13], and anti-inflammatory properties [14,15]. Its pharmaceutical relevance is further demonstrated by clinically important drugs such as sunitinib and other bioactive molecules containing this scaffold (Fig. 1). In addition, 3-(benzylidene)indolin-2-ones can be used as photo switches with high yields, robust photochemical switching, and quantum yields reaching up to 50 % [16]. Because of their significant pharmacological relevance and photo switches, the development of efficient and sustainable methods for the functionalization of indolin-2-one derivatives has attracted considerable attention in contemporary organic synthesis.

**Fig. 2:**
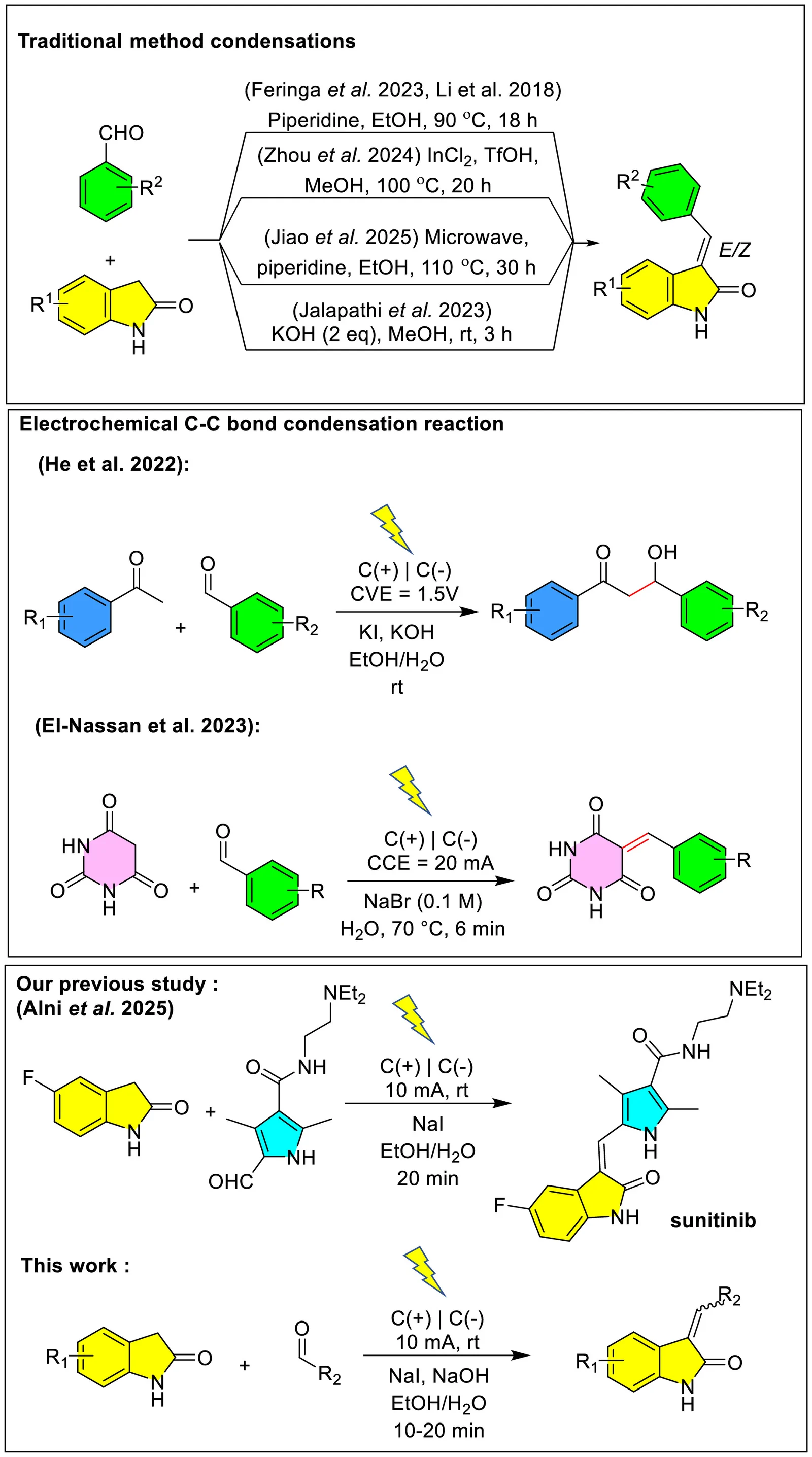
Condensation of indolin-2-one derivatives: previous studies and our approach.

Condensation reactions involving indolin-2-one derivatives are a straightforward strategy for constructing structurally diverse molecules with extended conjugated systems. Conventionally, these transformations are typically achieved through base-catalyzed reactions using piperidine [16,17], pyrrolidine [18], KOH [19] and metal-catalyzed i.e. InCl_2_/TfOH [20], nano-Ni_2_P/CeO_2_ [21], Fe(OAc)_2_ [22] (Fig. 2). Nevertheless, these approaches typically rely on organic solvents and often require elevated temperatures [23] or prolonged reaction times. Although these approaches can be effective, they often require harsh reaction conditions and generate undesirable chemical waste, which limits their sustainability from a green chemistry perspective.

Electrochemical synthesis has recently gained prominence as a potent and environmentally sustainable alternative for organic transformations, including condensation reactions [24,25]. By employing electric current as a clean redox reagent, electrochemical methods allow oxidative or reductive transformations without the need for external oxidants or reductants [[26], [27], [28]]. This strategy offers several advantages, including improved atom economy, operational simplicity, and compatibility with mild reaction conditions. In addition, the use of green solvent systems, such as ethanol-water mixtures, further enhances the sustainability of electrochemical processes [24,29]. In recent years, electrosynthesis has been applied to various carbon-carbon bond-forming condensation reactions such as electrochemical aldol-type condensation between acetophenone and aldehydes [30]. In addition, electrochemical Knoevenagel condensation involving barbituric acid with aldehydes has also been described, although this transformation typically requires relatively high reaction temperatures (70°C) [31]. Furthermore, detailed methodological information regarding electrochemical setup, optimization strategy, and reproducibility is often limited, making it difficult for other researchers to readily implement these procedures in their own laboratories.

Herein, we describe a practical electrochemical method for the Knoevenagel condensation of indolin-2-one derivatives with aromatic aldehydes using a simple undivided electrochemical cell. The methodology employs an ethanol-water medium, commercially available electrodes, and inexpensive iodide-based supporting electrolytes under ambient conditions. Systematic optimization of the reaction parameters is presented together with representative examples demonstrating the applicability of the method across structurally diverse substrates. The methodology was validated across a broad range of indolin-2-one derivatives and (hetero)aromatic aldehydes and further evaluated using green chemistry metrics to demonstrate its practicality and sustainability. This article provides sufficient experimental detail to facilitate the straightforward implementation and adaptation of the method for synthetic and medicinal chemistry research.

## Method details

### Materials

**Fig. 3:**
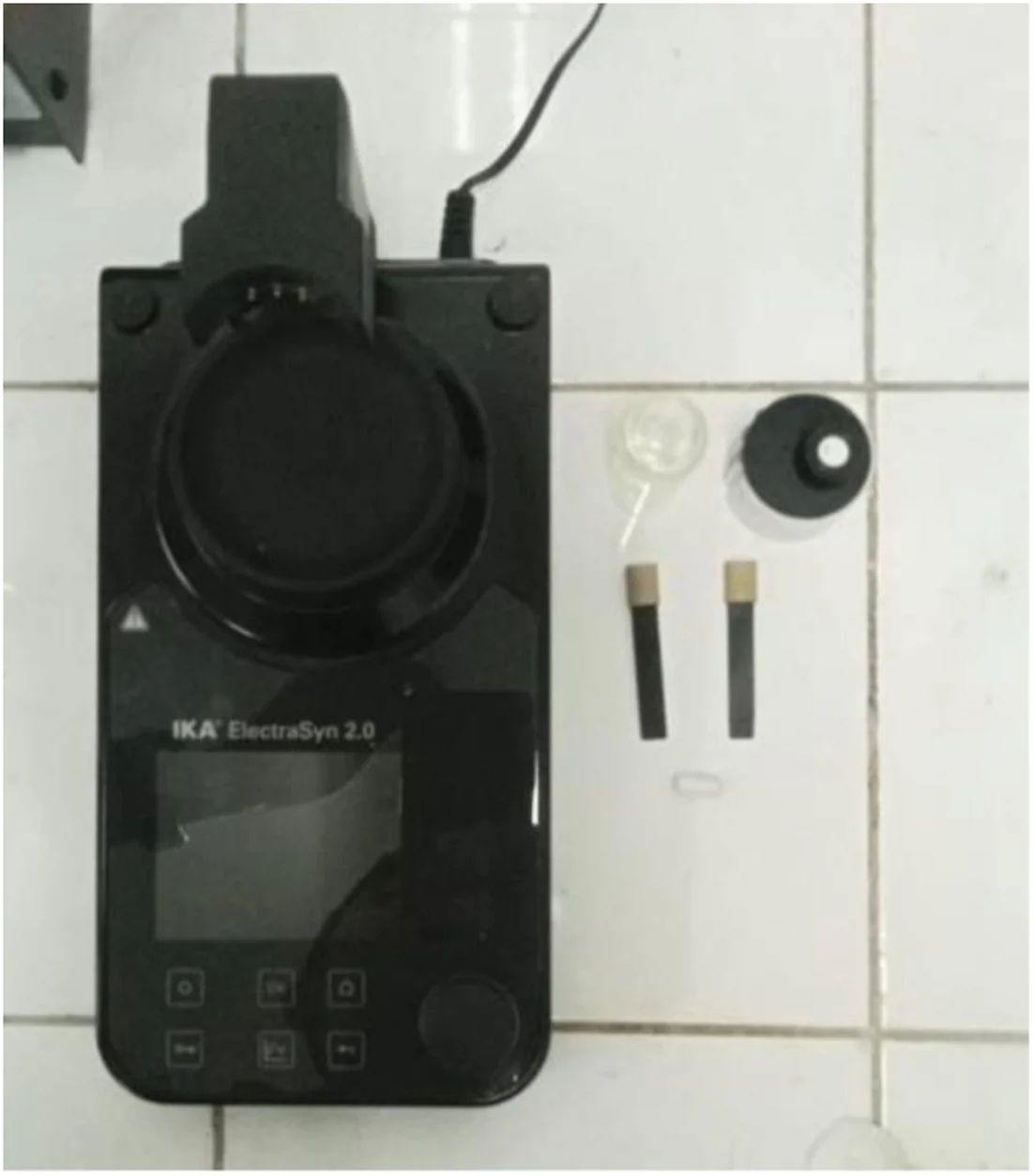
Electrochemical setup.

5-Fluoroindolin-2-one, 5-chloroindolin-2-one, 5-bromoindolin-2-one, 6-chloroindolin-2-one, indolin-2-one, ethyl 5-formyl-2,4-dimethyl-1H-pyrrole-3-carboxylate (**2**), 1*H*-pyrrole-2-carbaldehyde, furan-2-carbaldehyde, 5-(hydroxymethyl)furan-2-carbaldehyde, thiophene-2-carbaldehyde, benzaldehyde, 4-methoxybenzaldehyde, 2,4-dimethoxybenzaldehyde, 2,3,4-trimethoxybenzaldehyde, 2,4,5-trimethoxybenzaldehyde, 4-chlorobenzaldehyde, 3,4-dichlorobenzaldehyde, 4-(dimethylamino)benzaldehyde, 2-nitrobenzaldehyde, 4-methylbenzaldehyde, 2-chloro-4-nitrobenzaldehyde and sodium hydroxide analytical-grade pellets were purchased from Sigma-Aldrich. Ethanol absolute, acetonitrile, tetrahydrofuran (THF) were sourced from Smart Lab Indonesia. Electrochemical synthesis was performed in undivided cells exposed to air using an IKA ElectraSyn 2.0 with graphite electrode (Fig. 3).

The melting points of the compounds were determined using a Stuart Melting Point Apparatus. The synthesized products were characterized using an NMR spectrometer (DD2, 500 MHz, Agilent, Varian). Chemical shifts (δ) are reported in ppm relative to tetramethylsilane as the standard. Spectrophotometer ATR-IR (Alpha II, Bruker), high-resolution mass spectrometry (HRMS) using an ESI-TOF mass spectrometer (LCT Premier XE, Waters).

### Electrosynthesis of 5-fluoroindolin-2-one-pyrrole hybrid derivatives

Indolin-2-one derivatives with pyrrole scaffolds have demonstrated promising anticancer activity [[31], [32], [33]]. The indolin-2-one-pyrrole scaffold was constructed through a condensation reaction between 5-fluoroindolin-2-one (**1**) and pyrrolecarbaldehyde (**2**). The condensation reaction was explored using a halide electrolyte at a current of 10 mA in an H_2_O/EtOH (3:1) solvent system, as referenced in our previous research [34]. Indolin-2-one derivative **1** (0.30 mmol, 1.5 equiv.), pyrrolecarbaldehyde **2** (0.20 mmol, 1.0 equiv.) or benzaldehyde **3** (0.20 mmol, 1.0 equiv), and the supporting electrolyte (0.11 mmol, 0.55 equiv.) were added to a 10 mL ElectraSyn® reaction vial, followed by ethanol (1.0 mL). After complete dissolution of the reagents, distilled water (3.0 mL) and the base additive (Table 1) were added. The vial was equipped with two graphite electrodes (8 × 52.5 × 2 mm), serving as the anode and cathode, and inserted into an ElectraSyn® 2.0 instrument. The reaction mixture was electrolyzed under galvanostatic conditions at a constant current of 10 mA with continuous magnetic stirring at 500 rpm at room temperature for 10 min. The reactions were monitored using thin-layer chromatography (TLC) on 0.25 mm silica gel plates (60F-254) using the following solvents: (A) chloroform-methanol (9:1, v/v), (B) hexane-ethyl acetate (7:3 v/v). TLC was analyzed by observing fluorescence under a UV lamp at 254 nm. Upon completion of the electrolysis, distilled water (5 mL) and a few drops of ethanol were added to the reaction mixture and recrystallized with ethanol to obtain pure products. The optimization of this reaction continued with various modifications, such as different electrolytes, solvents, and additives, as presented in Table 1.

**Table 1:** Optimization of electro-condensation reactions.


No.	Solvent	Electrolyte	Additive	Electricity (mA)	Time (min)	Yield^a^ (%)	
						4	5a
1	H_2_O/EtOH (3:1)	NaBr	NaOH 20% mol	10	10	80	58
2	H_2_O/EtOH (3:1)	NaF	NaOH 20% mol	10	10	61	8
3	H_2_O/EtOH (3:1)	NaCl	NaOH 20% mol	10	10	66	4
4	H_2_O/EtOH (3:1)	NaI	NaOH 20% mol	10	10	94	71
5	H_2_O/EtOH (3:1)	TBAI	NaOH 20% mol	10	10	93	98
6	H_2_O/EtOH (3:1)	TBAB	NaOH 20% mol	10	10	70	97
7	EtOH	NaI	NaOH 20% mol	10	10	88	32
8	CH_3_CN	NaI	NaOH 20% mol	10	10	69	Trace
9	THF	NaI	NaOH 20% mol	10	10	58	22
10	H_2_O	NaI	NaOH 20% mol	10	10	48	39
11	H_2_O/EtOH (3:1)	NaI	K_2_CO_3_ 20% mol	10	10	77	28
12	H_2_O/EtOH (3:1)	NaI	TEA 20% mol	10	10	76	36
13	H_2_O/EtOH (3:1)	NaI	-	10	10	70	13
14	H_2_O/EtOH (3:1)	NaI	NaOH 2.5% mol	10	10	72	10
15	H_2_O/EtOH (3:1)	NaI	NaOH 10% mol	10	10	93	63
16	H_2_O/EtOH (3:1)	NaI	-	-	10	Trace	Trace
17	H_2_O/EtOH (3:1)	NaI	NaOH 20% mol	-	10	60	8

a Isolated yields.

Optimization was performed using two aldehyde substrates, pyrrolecarbaldehyde **2** and benzaldehyde **3**. Product **4** generally afforded higher yields than product **5a**. However, variations in the reaction parameters, including the electrolyte, additive, and solvent, resulted in similar yield trends for both products. Among the electrolytes examined, iodide-based electrolytes (NaI and TBAI) afforded the highest yields of both products **4** and **5a** (entry 4 and 5), with NaI selected for subsequent studies because of its excellent performance and cost-effectiveness. The H₂O/EtOH (3:1, v/v) solvent system afforded the highest yields (94% for **4** and 71% for **5a**), providing a balance between substrate solubility and electrolyte conductivity. Furthermore, the influence of the base additives was investigated. The absence of a base, the reaction afforded compound **4** in 70% yield. The addition of 2.5 mol% NaOH slightly improved the yield **4** and **5a** (72% and 10%), whereas increasing the base loading to 20 mol% NaOH significantly enhanced the yield (entry 4). These results suggested a synergistic effect between electrolysis and basic conditions, as the reaction proceeded more efficiently under basic conditions. In the absence of electricity (entry 17), a lower yield was obtained compared with that under electrochemical conditions. The external base likely acted as an initiator of the catalytic cycle by promoting the formation of hydroxide ions from water. Under base conditions, water was more readily reduced at the cathode to generate hydroxide ions [35]. Finally, electrolysis at a constant current of 10 mA for 10 min was identified as the optimal condition.

### Electrosynthesis of 3-benzylidene-5-fluoroindolin-2-one derivatives under the optimized conditions

These optimized conditions were subsequently employed throughout the study to evaluate the applicability of the method toward various (hetero)aromatic aldehydes (**3-o**) and 5-fluoroindolin-2-one (**1**) (Table 2). For the general electrochemical synthesis of 3-(benzylidene)indolin-2-one derivatives (**4**), 5-fluoroindolin-2-one **1** (0.30 mmol, 1.5 equiv.) and a (hetero)aromatic aldehyde **3a-o** (0.20 mmol, 1.0 equiv.), and NaI (0.11 mmol, 0.55 equiv.) were added to a 10 mL ElectraSyn® reaction vial, followed by ethanol (1.0 mL). After complete dissolution of the reagents, destilled water (3.0 mL) and NaOH (1 M, 40 μL, 20 mol%) were added. The vial was equipped with two graphite electrodes (8 × 52.5 × 2 mm), serving as the anode and cathode, and inserted into an ElectraSyn® 2.0 instrument. The reaction mixture was electrolyzed under galvanostatic conditions at a constant current of 10 mA with continuous magnetic stirring at 500 rpm at room temperature for 10 min. The reactions were monitored using thin-layer chromatography (TLC) on 0.25 mm silica gel plates (60F-254) using the following solvents: (A) chloroform-methanol (9.5:0.5, v/v), (B) hexane-ethyl acetate (8:2 v/v). TLC was analyzed by observing fluorescence under a UV lamp at 254 nm. Upon completion of the electrolysis, distilled water (5 mL) and a few drops of ethanol were added to the reaction mixture and recrystallized with ethanol to obtain pure products.

**Table 2:** Electrosynthesis of 3-benzylidene-5-fluoroindolin-2-one derivatives.


Entry	Comp. **5**	R	Yield (%)^a^	% *E/Z* -isomer		δ_H_ 2’,6’-H or H-vinyl (ppm)	
				Ratio	Integration	*E-*isomer	*Z-*isomer
1	**5a**	C_6_H_5_	71	*E* only	2.08:0	7.70	N/A
2	**5b**	4-OMeC_6_H_4_	30	93:7	2.09:0.15	7.71 (2’,6’)	8.49 (2’,6’)
3	**5c**	2,4-(OMe)_2_C_6_H_3_	96	*E* only	1.00:0	7.66 (6’)	N/A
4	**5d**	2,3,4-(OMe)_3_C_6_H_2_	97	*E* only	1.01:0	7.48 (6’)	N/A
5	**5e**	2,4,5-(OMe)_3_C_6_H_2_	70	98:2	0.93:0.02	7.71 (6’)	7.99 (6’)
6	**5f**	4-ClC_6_H_4_	76	93:7	2.00:0.16	7.73 (2’,6’)	8.40 (2’,6’)
7	**5g**	3,4-Cl_2_C_6_H_4_	58	97:3	1.09:0.02 1.13:0.03	7.91 (2’) 7.81 (6’)	8.84 (2’) 8.21 (6’)
8	**5h**	2-NO_2_C_6_H_4_	51	*E* only	1.00:0	8.33 (6’)	N/A
9	**5i**	4-*N(*CH_3_)_2_C_6_H_4_	57	96:4	2.05:0.09	7.64 (2’,6’)	8.43 (2’,6’)
10	**5j**	4-MeC_6_H_4_	94	97:3	2.15:0.06	7.60 (2’,6’)	8.32 (2’,6’)
11	**5k**	2-Cl-4-NO_2_C_6_H_4_	92	86:14	0.96:0.16	7.85 (6’)	7.96 (6’)
12	**5l**	pyrroline	98	*Z* only	0:1.00	N/A	7.83 (vinyl)
13	**5m**	thiophene	94	*Z* only	0:1.00	N/A	7.85 (vinyl)
14	**5n**	furan	96	97:3	1.00:0.07	7.39 (vinyl)	7.79 (vinyl)
15	**5o**	5-hydroxymethylfurfural	81	97:3	1.12:0.04	7.35 (vinyl)	7.75 (vinyl)

a Isolated yield, N/A: Not available.

This electrochemical protocol afforded fifteen Knoevenagel condensation products (**5a**-**5o**) from various (hetero)aromatic aldehydes (Table 2). Those (hetero)aromatic aldehydes bearing electron-donating groups generally afforded good yields. Methoxy-substituted aldehydes (**5c**-**5e**) showed good reactivity. A methyl substituent (**5j**) also provided a good yield. In contrast, (hetero)aromatic aldehydes containing electron-withdrawing groups, such as nitro (**5h**), resulted in moderate yields. Chloro-substituted (hetero)aromatic aldehydes (**5f**-**g**) were also tolerated, producing the desired products in satisfactory yields. Moreover, (hetero)aromatic aldehydes, including pyrrole (**5l**), thiophene (**5m**), and furan (**5n**-**o**), were compatible with the reaction conditions and afforded products in yields of up to 98%. A similar trend has been reported in related condensation reactions, where electron-donating substituents provided higher yields than electron-withdrawing substituents [31]. The electronic properties of the substituents affect the reactivity of the aldehyde and the overall condensation process, while substituent position and steric size can further influence the formation and stability of the resulting 3-(benzylidene)indolin-2-one products [16]

**Fig. 4:**
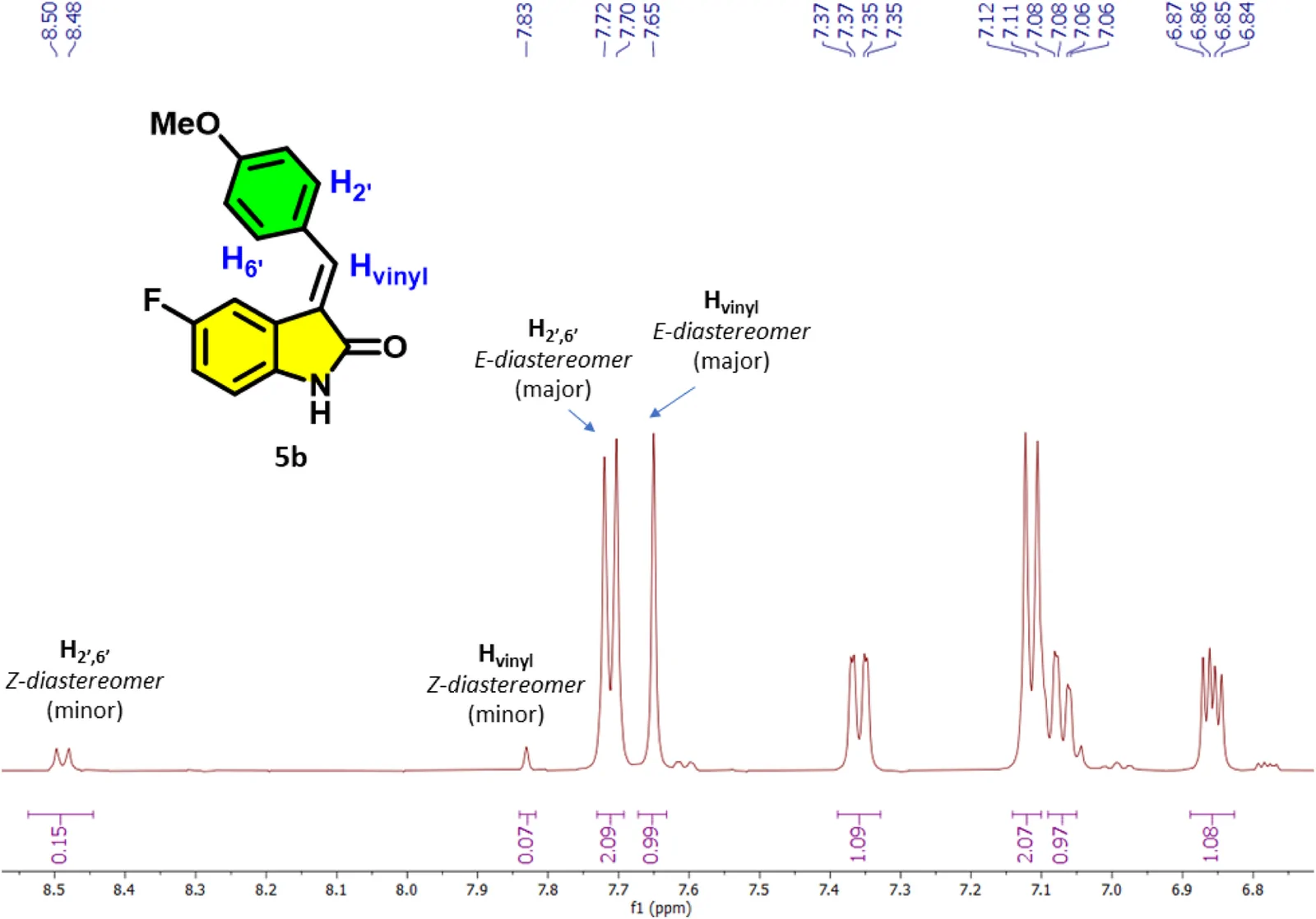
^1^H-NMR spectrum (aromatic region) of compound **5b** with ratio *E/Z*-isomer 93:7.

The synthesized 3-(benzylidene)indolin-2-one derivatives (**5a-5k**) were generally obtained with high stereoselectivity, predominantly affording the *E*-isomers (*E*= 86 to 100%). *E/Z* ratios were determined by integration of the corresponding 2′-H and/or 6′-H signals for 3-benzylidene derivatives, and the H-vinyl signal for the corresponding derivatives in the ¹H-NMR spectra [32,36]. For example, compound **5b** exhibited an *E/Z* ratio of 93:7 based on the integration of the diagnostic 2′,6′-H signals in the ¹H-NMR spectrum (Fig. 4). The *E*-isomer showed a signal at δ 7.71 ppm with an integral of 2.09, while the corresponding *Z*-isomer signal appeared at δ 8.49 ppm with an integral of 0.15. The *E/Z* ratio was calculated from the relative integral values, 2.09/(2.09 + 0.15) × 100 and 0.15/(2.09 + 0.15) × 100, giving 93% *E* and 7% *Z*, respectively. The corresponding ¹H NMR spectra and integrations for the other compounds are provided in the Supporting Information. The preference for the *E*-configuration can be attributed to its higher thermodynamic stability, as the arylidene substituent and the carbonyl moiety are positioned on opposite sides of the exocyclic C=C bond, thereby minimizing steric repulsion. Notably, the electrochemical protocol furnished the desired products within only 10 min while maintaining high *E*-selectivity, indicating that the stereochemical preference for the thermodynamically favored *E*-isomer was preserved under the electrochemical conditions [37]. In contrast, reactions involving pyrrole- and thiophene-derived aldehydes (**5l** and **5m**) furnished exclusively the *Z*-isomers. This stereochemical reversal is attributed to stabilizing intramolecular interactions in the *Z*-configuration, namely an N–H···O hydrogen bond between the pyrrole N–H and the carbonyl oxygen, and an electrostatic S···O interaction between the thiophene sulfur atom and the carbonyl oxygen, which favor the *Z*-isomer. Electrostatic repulsion between the furan oxygen atom and the carbonyl oxygen (compound **5n** and **5o**) disfavors the *Z*-configuration, resulting in preferential formation of the *E*-isomer [32].

## Method validation

### Scope validation

The developed methodology was successfully applied to a broad range of pyrrole-2-carbaldehyde and indolin-2-one derivatives under the optimized reaction conditions (Table 3). While most aromatic benzaldehydes (**3**) reached satisfactory conversion within 10 min, pyrrole-2-carbaldehyde (**7**) derivatives required an extended electrolysis time of 20 min under otherwise identical reaction conditions. Pyrrole-2-carbaldehyde derivative (**7**) was either commercially available and used as received or prepared according to previously reported procedures [34,38]. These results demonstrate the broad applicability and adaptability of the developed electrochemical methodology.

**Table 3:** Validation of the electrochemical Knoevenagel condensation method using structurally diverse substrates.


Entry	Compound	R_1_	R_2_	Yield (%)^a^
1	**8a**	H	NHCH_2_CH_2_NEt_2_	67^b^
2	**8b**	5-F	NHCH_2_CH_2_NEt_2_	95^b^
3	**8c**	5-Cl	NHCH_2_CH_2_NEt_2_	73^b^
4	**8d**	5-Br	NHCH_2_CH_2_NEt_2_	51^b^
5	**8e**	5-NO_2_	NHCH_2_CH_2_NEt_2_	60^b^
6	**8f**	6-Cl	NHCH_2_CH_2_NEt_2_	51^b^
7	**8g**	5-F	NHCH_2_CH_2_(morpholine)	55^b^
8	**8h**	5-Cl	NHCH_2_CH_2_(morpholine)	65^b^
9	**8i**	5-NO_2_	NHCH_2_CH_2_(morpholine)	57^b^
10	**8j**	6-Cl	NHCH_2_CH_2_(morpholine)	93^b^
11	**8k**	5-F	NHCH_2_CH_3_	82
12	**8l**	5-Cl	NHCH_2_CH_3_	58
13	**8m**	6-Cl	NHCH_2_CH_3_	85
14	**8n**	5-F	NH(CH_2_)_3_CH_3_	75
15	**8o**	5-Cl	NH(CH_2_)_3_CH_3_	59
16	**8p**	5-F	NHCH(CH_3_)_2_	80
17	**8q**	5-F	NHCH_2_(4-OMeC_6_H_4_)	75
18	**8r**	5-F	Morpholine	86
19	**8s**	H	OCH_2_CH_3_	79
20	**4**	5-F	OCH_2_CH_3_	94
21	**8t**	5-Cl	OCH_2_CH_3_	82
22	**8u**	5-Br	OCH_2_CH_3_	90
23	**8v**	5-NO_2_	OCH_2_CH_3_	82
24	**8w**	6-Cl	OCH_2_CH_3_	87

a Isolated yield, ^b^without addition NaOH

Pyrrolecarboxamide substrates containing basic groups, such as *N,N*-diethylaminoethyl substituents, underwent the reaction without the addition of NaOH, affording products **8a-j**. These results suggested that basic pyrrolecarbaldehydes generally gave higher yields than neutral substrates. In contrast, pyrrolecarboxamides without basic substituents required NaOH to produce condensation products with yields of 52–94%. These results indicated that the reaction tolerated various modifications on the pyrrole-2-carbaldehyde derivative.

Reaction conditions: indolin-2-one derivative **6** (0.15 mmol), pyrrolecarbaldehydes derivative **7** (0.10 mmol), H_2_O/EtOH (3:1, 4 mL), NaI (0.025 M), carbon as anode and cathode, constant current of 10 mA, 20 min.

**Fig. 5:**
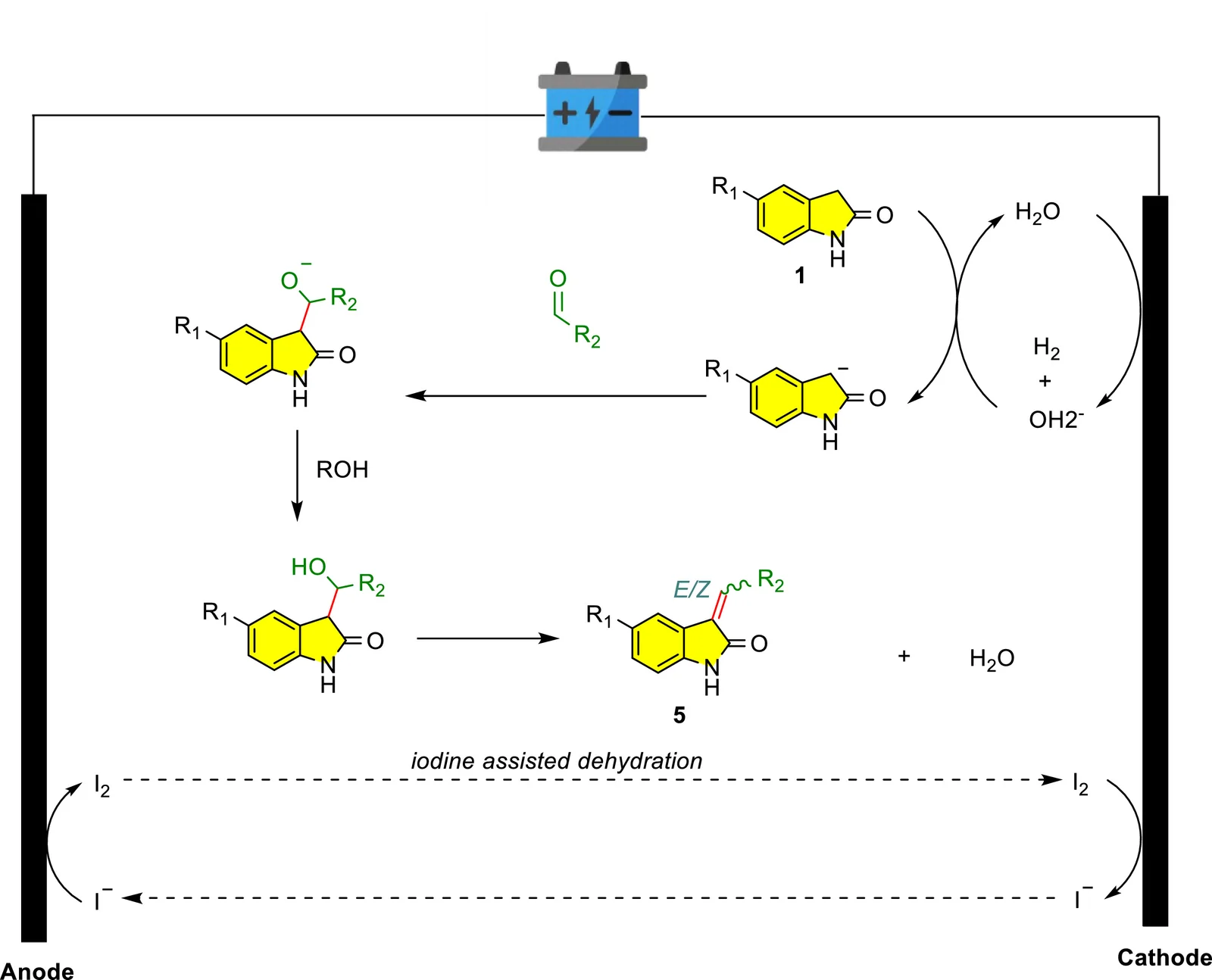
Proposed mechanism for the electrochemical condensation of indolin-2-one [34].

Electrochemical condensation likely proceeded via a base-assisted Knoevenagel-type pathway mediated by iodide [39]. Under electrolysis conditions, iodide was anodically oxidized to reactive iodine species (I₂/I⁺) (Fig. 5). These iodine species facilitated the dehydration step of the intermediate alcohol formed during condensation. Previous studies by Stavber and Jereb reported that low concentrations of iodine can promote the dehydration of alcohols under mild conditions without the need for elevated temperatures [40,41]. In the cathodic compartment, water was reduced to generate hydroxide ions and hydrogen gas. Hydrogen bubbles were observed at the cathode during electrolysis. The generated hydroxide ions acted as a base and promoted the deprotonation of compound **1** (indolin-2-one) to form the corresponding carbanionic intermediate. This nucleophilic species then attacked aldehyde, forming a *β*-hydroxy intermediate that subsequently underwent iodine-assisted dehydration to afford products **5** or **8**. Meanwhile, the iodine species generated at the anode were reduced to iodide at the cathode, thereby completing the iodide redox cycle. A similar mechanistic pathway was proposed by He et al. for iodide-mediated electrochemical aldol condensation reactions [34,42].

### Structural validation

**Fig. 6:**
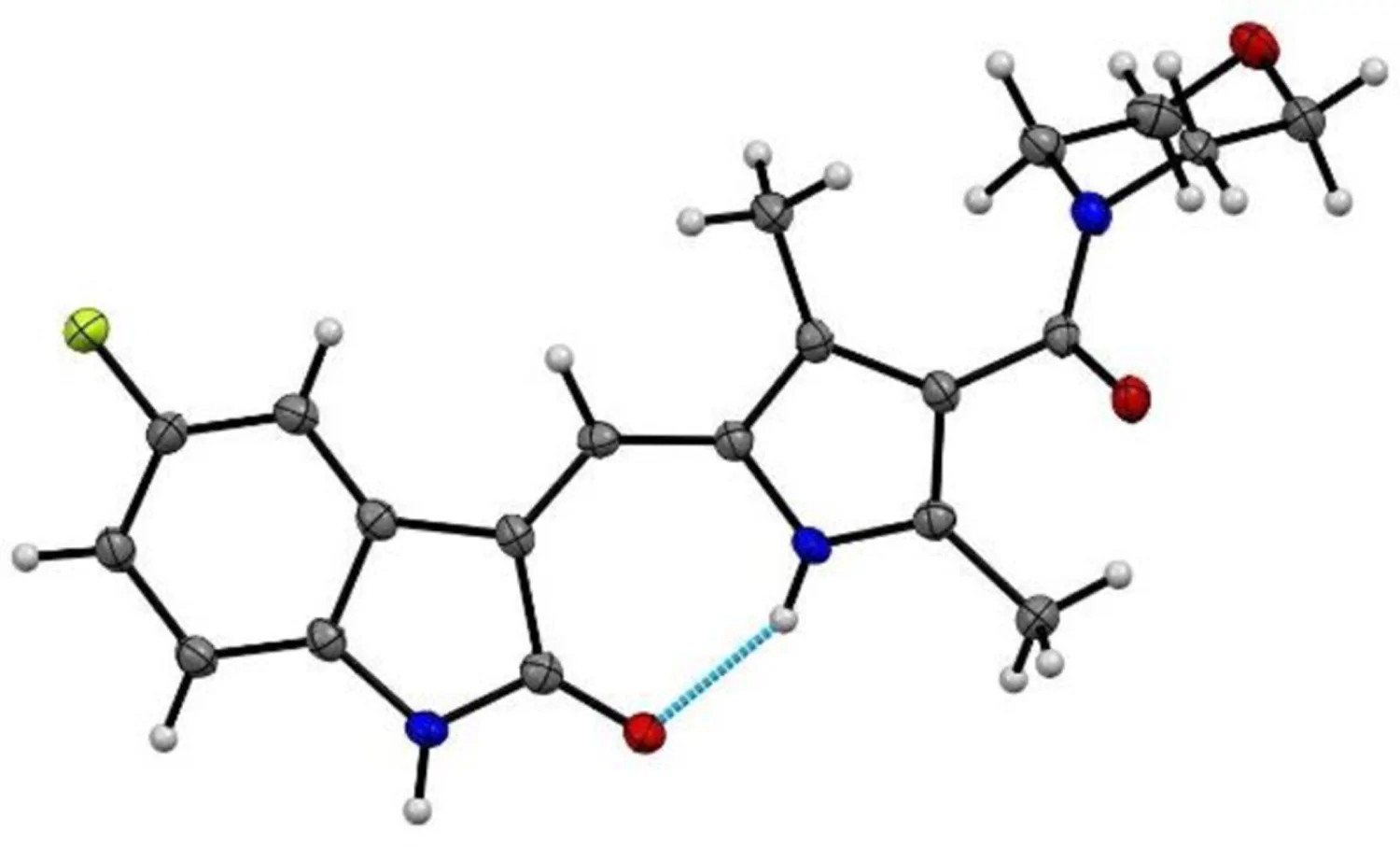
Crystal structure of compound **8r** (CCDC 2499402).

All the compounds were confirmed by melting point, ^1^H-NMR, ^13^C-NMR, FTIR, and HRMS analyses. The spectroscopic data were consistent with the proposed molecular structures and are provided in the Supporting Information. In addition, the molecular structure of compound **8r** was further confirmed by single-crystal X-ray diffraction analysis, providing definitive structural evidence for the electrochemical Knoevenagel condensation product (Fig. 6). The crystallographic data have been deposited with the Cambridge Crystallographic Data Centre (CCDC 2499402).

### Sustainability assessment (green metrics)

**Fig. 7:**
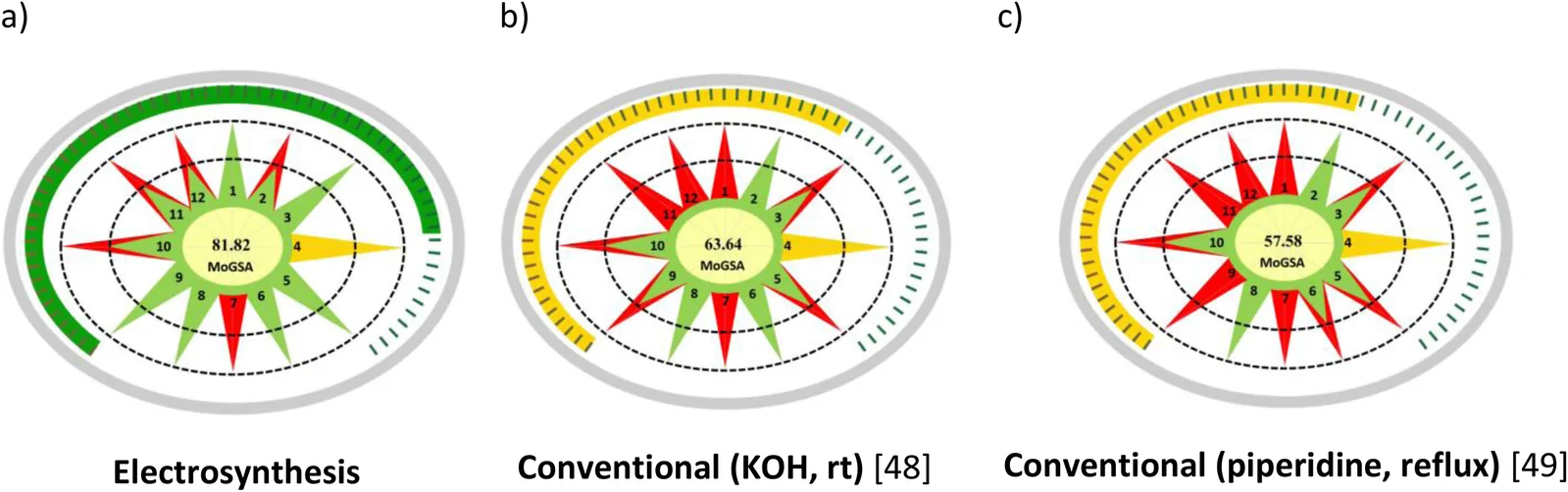
Green star score for the proposed electro-condensation protocol.

The sustainability of the developed electrochemical methodology was evaluated using several green chemistry metrics, including the Process Mass Intensity (PMI), energy intensity, time intensity, E-factor, EcoScale [43,44] (Table 4) and Green Star (Fig. 7). Atom economy (AE), molar efficiency (ME), process mass intensity (PMI), and E-factor were evaluated based on literature procedures [44] while energy intensity (EI) and time intensity (TI) have been calculated according to literature [45]. The total energy consumed during electrolysis (E) was calculated using E=V.I.t, where U is the cell voltage, I is the current, and t is the electrolysis time. For the conventional reaction, the total energy consumption (E) was calculated as E=P × t, assuming an effective heating power of 250 W. EcoScale assessment, including yield, reagents, price and availability, safety, technical setup, temperature/time, workup, and purification, was calculated according to the literature [43] and is available at https://ecoscale.cheminfo.org/. Green Star assessment was conducted using the software developed by Mansour et al [46], which evaluates the twelve principles of green chemistry.

**Table 4:** Green metrics for the developed electro-condensation.

Process	Comp.	Green metrics						
		AE (%)	ME (%)	PMI	Energy intensity (kWh. g^−1^)	Time intensity (min. g^−1^)	E-factor	EcoScale
Ideal values		100	100	1	0	0	0	100
Electrosynthesis (this work)	5a	93.00	26.48	1.97	2.44×10^−4^	293	0.95	70
	5b	93.73	11.11	4.44	5.05×10^−4^	606	3.39	48
	5f	93.82	28.15	1.80	1.99×10^−4^	239	0.75	67
	5l	92.69	36.30	1.46	1.84×10^−4^	221	0.42	80
	5m	93.16	34.81	1.49	1.78×10^−4^	214	0.45	81
	5n	93.08	45.00	1.49	1.86×10^−4^	224	0.44	78
Conventional synthesis (KOH, rt, 3 h) [47]	5a	93.00	9.54	3.25	n.d.	986	0.41	70
	5b	93.73	5.35	5.41	n.d.	1562	1.49	53
	5f	93.82	5.53	5.19	n.d.	1488	1.41	49
	5l	92.69	6.80	4.69	n.d.	1460	0.98	54
	5m	93.16	5.58	5.48	n.d.	1645	1.14	54
	5n	93.08	9.06	3.51	n.d.	1083	0.49	63
Conventional synthesis (piperidine, reflux, 3-5 h) [48]	5a	93.00	40.95	1.29	4860ᵃ	1166	0.25	76
	5l	92.69	40.49	1.31	5155^a^	1237	0.27	73
	5m	93.16	38.10	1.39	5096^a^	1223	0.34	73

ᵃ Calculated by assuming an effective heating power of 250 W, n.d.: not determined.

The method employs an ethanol/water solvent system, graphite electrodes, and an inexpensive iodide electrolyte under ambient conditions without external heating. Product isolation was achieved by simple filtration followed by recrystallization from ethanol, eliminating the need for chromatographic purification. The favorable values obtained for the evaluated green metrics demonstrate that the developed methodology provides an environmentally benign and resource-efficient approach for the synthesis of indolin-2-one derivatives.

## Limitations

The developed methodology was applicable to a broad range of indolin-2-one derivatives and heteroaromatic aldehydes. However, substrates containing free phenolic hydroxyl groups (e.g., 4-hydroxybenzaldehyde) or carboxylic acid groups (e.g., 5-formyl-2,4-dimethyl-1H-pyrrole-3-carboxylic acid) exhibited only trace product formation under the optimized reaction conditions. Consequently, the present protocol may not be suitable for aldehydes bearing acidic functional groups without further optimization

## Ethics statements

Not applicable.

## CRediT author statement

**Robby Gus Mahardika**: Writing – original draft, Resources, Investigation, Conceptualization. **Ade Danova**: Writing – review & editing, Supervision, Investigation, Conceptualization. **Warinthorn Chavasiri**: Writing – review & editing, Supervision. **Elvira Hermawati**: Writing – review & editing, Visualization, Data curation, Supervision. **Anita Alni**: Writing – review & editing, Supervision, Conceptualization.

Supplementary material

## Declaration of competing interest

The authors declare that they have no known competing financial interests or personal relationships that could have appeared to influence the work reported in this paper.

## Data Availability

Data will be made available on request.
