# Supplementary material for: Green Knoevenagel condensation of indolin-2-one derivatives using undivided electrochemical cell
Source: MethodsX. 2026 Sep 6;17:104145. doi: 10.1016/j.mex.2026.104145 (PMC13586819; doi:10.1016/j.mex.2026.104145)

Supporting Information

Green Knoevenagel Condensation of Indoline-2-one Derivatives Using Undivided Electrochemical Cell

Robby Gus Mahardika[1,2], Ade Danova[2], Warinthorn Chavasiri[3], Elvira Hermawati[2], and Anita Alni[2*]

[1]Doctoral Program of Chemistry, Faculty of Mathematics and Natural Sciences, Institut Teknologi Bandung, Jl. Ganesha No. 10, Bandung, West Java 40132, Indonesia

[2]Organic Chemistry Division, Faculty of Mathematics and Natural Sciences, Institut Teknologi Bandung, Jl. Ganesha No. 10, Bandung, West Java 40132, Indonesia

[3]Center of Excellence in Natural Products Chemistry, Department of Chemistry, Faculty of Science, Chulalongkorn University, Bangkok, Thailand

*E-mail: alni@itb.ac.id

1. General Information 2
2. Experimental Section..2
3. Single-Crystal X-ray Structure Determination 20
4. Green Metric Calculations...22
5. References 24
6. Spectra 25

1. General Information

Electrochemical synthesis was performed in undivided cells exposed to air using an Electrasyn® reactor. The progress of the reactions was monitored using thin-layer chromatography (TLC) on 0.25 mm silica gel plates (60F-254) and analyzed by observing fluorescence under a UV lamp at 254 nm. Reagents and solvents were sourced from Aldrich and Smart Lab Indonesia. The melting points of the compounds were determined using a Stuart Melting Point Apparatus. The synthesized products were characterized using an NMR spectrometer (DD2, 500 MHz, Agilent, Varian), spectrophotometer ATR-IR (Alpha II, Bruker), high-resolution mass spectrometry (HRMS) using an ESI-TOF mass spectrometer (LCT Premier XE, Waters), and Single Crystal X-ray Diffraction (SC-XRD) (D8 Venture, Bruker).

2. Experimental Section

Reaction was performed in undivided cells using IKA ElectraSyn 2.0 with graphite electrode.

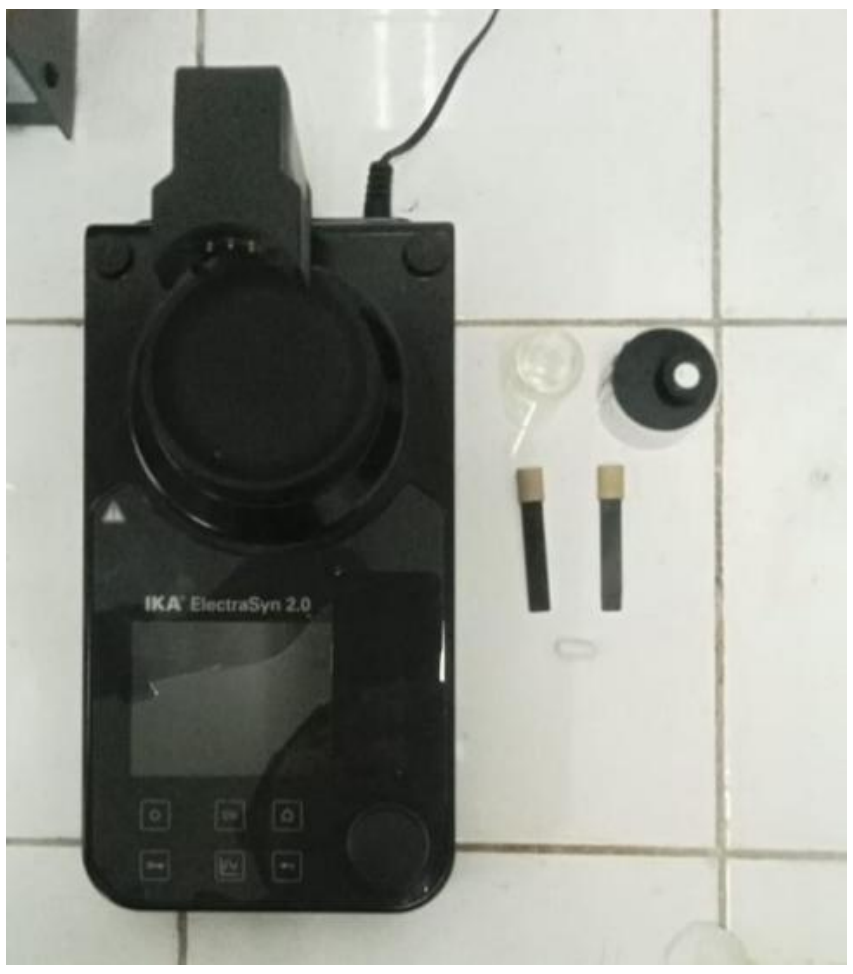

Figure S1. Electrochemical setup

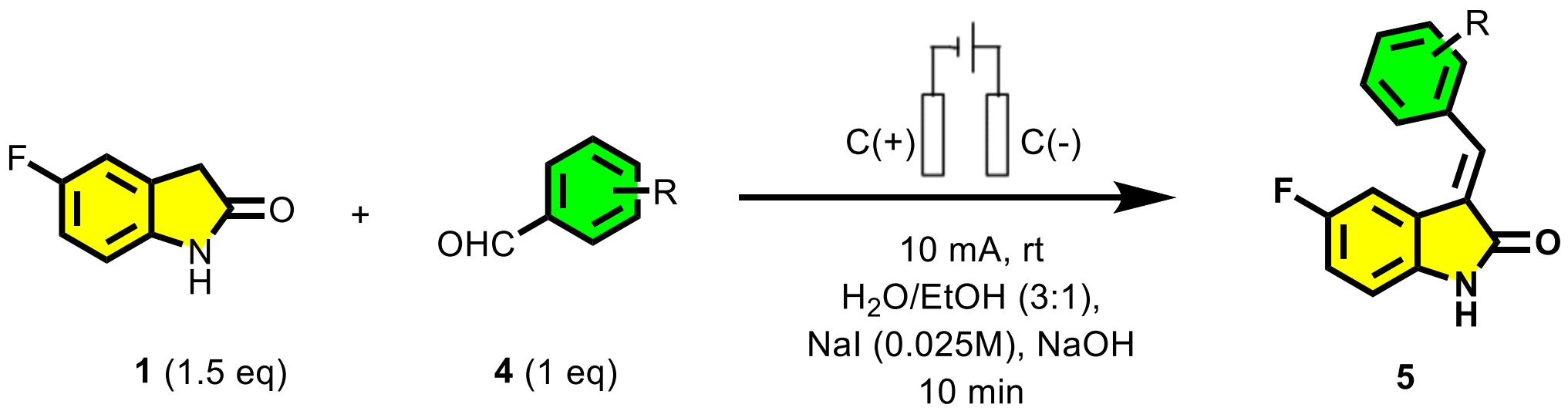

1 (1.5 eq) **4** (1 eq) **5**

The general procedure for electrocondensation involved dissolving compounds 1 (1.5 equiv.) and 3 (1 equiv.) in 4 mL of solvent. A base additive and carbon electrodes were added, and the reaction was conducted at room temperature under a constant current of 10 mA. After completion of the reaction, distilled water (5 mL) and a few drops of ethanol were added to the reaction mixture and recrystallized with ethanol to obtain pure products.

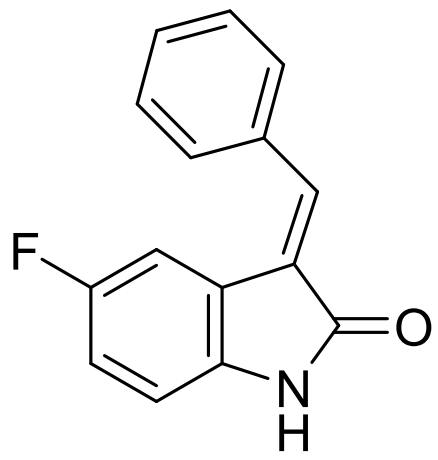

***(E)*-3-Benzylidene-5-fluoroindolin-2-one (5a)**. Orange-yellow solid; yield 71%; mp: 196 °C. IR (ATR, ν_{max}, cm^{-1}): 3164, 3026, 2879, 1620, 1574, 1446.

^{1}H NMR (500 MHz, DMSO-d_6) δ_H 10.66 (s, 1H, H-1$_{-oxindole}$), 7.72 – 7.68 (m, 3H), 7.55 (t, *J* = 7.3 Hz, 2H, H$_{-aryl}$), 7.51 (t, *J* = 7.2 Hz, 1H, H$_{-aryl}$), 7.19 (dd, *J* = 9.3, 2.6 Hz, 1H, H-4$_{-oxindole}$), 7.09 (td, *J* = 9.0, 2.8 Hz, 1H, H-6$_{-oxindole}$), 6.86 (dd, *J* = 8.5, 4.6 Hz, 1H, H-7$_{-oxindole}$).

^{13}C NMR (125 MHz, DMSO-d_6) δ_C 169.0 (C=O), δ 157.6 (d, J_{CF} = 235.1 Hz, C-5$_{-oxindole}$), 139.8 (C$_{-alkene}$), 138.0 (C-7a$_{-oxindole}$), 134.5 (C$_{-aryl}$), 130.5 (C$_{-aryl}$), 129.7 (C$_{-aryl}$), 129.4 (C$_{-aryl}$), 127.9 (C-3$_{-oxindole}$), 122.3 (d, J_{CF} = 8.6 Hz, C-3a$_{-oxindole}$), 117.0 (d, J_{CF} = 23.4 Hz, C-6$_{-oxindole}$), 111.3 (d, J_{CF} = 8.6 Hz, C-7$_{-oxindole}$), 109.8 (d, J_{CF} = 25.7 Hz, C-4$_{-oxindole}$).

^{19}F NMR (470 MHz, DMSO-d_6) δ_F -122.1.

HRMS (ESI-TOF) *m/z* $[M+H]^+$ calcd. for $C_{15}H_{10}FNO$: 240.0819, found: 240.0825.

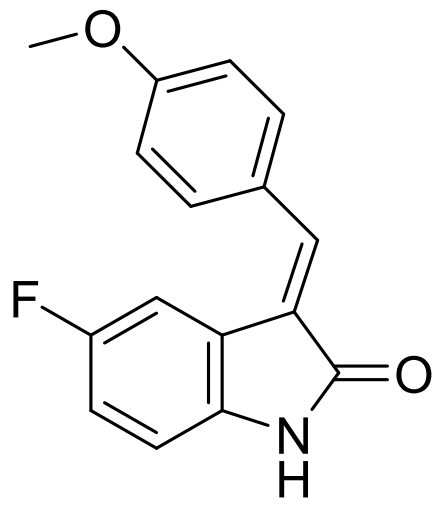

(*E*)-5-fluoro-3-(4-methoxybenzylidene)indolin-2-one (5b). Orange-yellow solid; yield 30%; mp: 172 °C. IR (ATR, ν_{max}, cm^{-1}): 3152, 3049, 2938, 2843, 1685, 1583, 1462.

^{1}H NMR (500 MHz, DMSO-d_6) δ_H 10.59 (s, 1H, H-1$_{-oxindole}$), 7.71 (d, *J* = 8.8 Hz, 2H, H$_{-aryl}$), 7.65 (s, 1H, H$_{-alkene}$), 7.36 (dd, *J* = 9.5, 2.8 Hz, 1H, H-4$_{-oxindole}$), 7.11 (d, *J* = 8.7 Hz, 2H, H$_{-aryl}$), 7.07 (dd, *J* = 9.0, 2.7 Hz, 1H, H-4$_{-oxindole}$), 6.86 (dd, *J* = 8.6, 4.6 Hz, 1H, H-7$_{-oxindole}$), 3.85 (s, 3H).

^{13}C NMR (125 MHz, DMSO-d_6) δ_C 169.3 (C=O), 161.3 (=C-O), δ 157.6 (d, J_{CF} = 234.6 Hz, C-5$_{-oxindole}$), 139.4 (C$_{-alkene}$), 138.2 (C-7a$_{-oxindole}$), 132.1 (2C, C$_{-aryl}$), 126.6 (C$_{-aryl}$), 125.7 (C$_{-aryl}$), 122.6 (C-3a$_{-oxindole}$), 116.4 (d, J_{CF} = 23.4 Hz, C-6$_{-oxindole}$), 114.9 (2C, C$_{-aryl}$), 111.1 (d, *J* = 8.6 Hz, C-7$_{-oxindole}$), 109.52 (d, *J* = 26.2 Hz, C-4$_{-oxindole}$), 55.9 (OCH_3)

^{19}F NMR (470 MHz, DMSO-d_6) δ_F -122.2
HRMS (ESI-TOF) *m/z* $[M+H]^+$ calcd. for $C_{16}H_{12}FNO_2$: 270.0925, found: 270.0920.

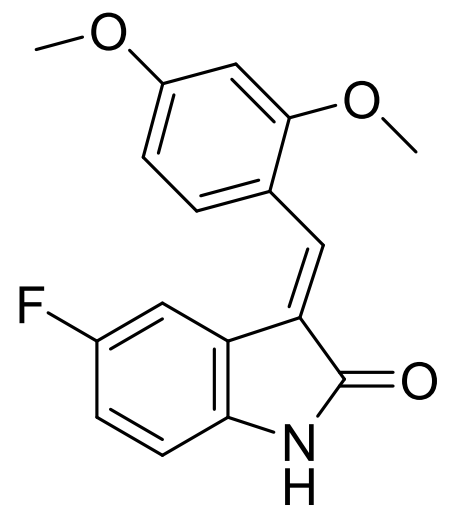

(*E*)-3-(2,4-dimethoxybenzylidene)-5-fluoroindolin-2-one (5c). Orange-yellow solid; yield 96%; mp: 210 °C. IR (ATR, ν_{max}, cm^{-1}): 3153, 3013, 2934, 2837, 1687, 1572, 1461.

^{1}H NMR (500 MHz, DMSO-d_6) δ_H 10.56 (s, 1H, H-1$_{\text{-oxindole}}$), 7.68 (s, 1H, H$_{\text{-alkene}}$), 7.66 (d, *J* = 9.2 Hz, 1H, H$_{\text{-aryl}}$), 7.22 (dd, *J* = 9.4, 2.6 Hz, 1H, H-4$_{\text{-oxindole}}$), 7.05 (td, *J* = 9.1, 2.7 Hz, 1H, H-6$_{\text{-oxindole}}$), 6.84 (dd, *J* = 8.6, 4.6 Hz, 1H, H-7$_{\text{-oxindole}}$), 6.72 – 6.70 (m, 2H, H$_{\text{-aryl}}$), 3.87 (s, 3H, OCH_3), 3.86 (s, 3H, OCH_3).

^{13}C NMR (125 MHz, DMSO-d_6) δ_C 169.4 (C=O), 163.5(=C-O), 160.0 (=C-O), 157.62 (d, J_{CF} = 234.6 Hz, C-5$_{\text{-oxindole}}$), 139.2 (C$_{\text{-alkene}}$), 133.8 (C$_{\text{-aryl}}$), 131.2 (C$_{\text{-aryl}}$), 125.35 (d, J_{CF} = 2.9 Hz, C-3$_{\text{-oxindole}}$), 122.85 (d, J_{CF} = 9.1 Hz, C-3a$_{\text{-oxindole}}$), 116.07 (d, J_{CF} = 23.4 Hz C-6$_{\text{-oxindole}}$), 115.4 (C$_{\text{-aryl}}$), 110.99 (d, J_{CF} = 8.6 Hz, C-7$_{\text{-oxindole}}$), 109.39 (d, J_{CF} = 25.7 Hz, C-6$_{\text{-oxindole}}$), 105.9, 99.1, 56.3 (OCH_3), 56.0 (OCH_3).

^{19}F NMR (470 MHz, DMSO-d_6) δ_F -122.4.
HRMS (ESI-TOF) *m/z* $[M+H]^+$ calcd. for $C_{16}H_{12}FNO_2$: 300.1030, found: 300.1028.

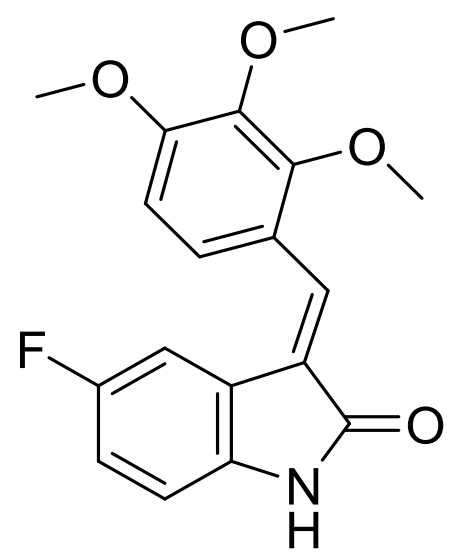

(*E*)-5-fluoro-3-(2,3,4-trimethoxybenzylidene)indolin-2-one (5d). Orange-yellow solid; yield 97%; mp: 190 °C. IR (ATR, ν_{max}, cm^{-1}): 3152, 3049, 2938, 2843, 1685, 1583, 1462.

^{1}H NMR (500 MHz, DMSO-d_6) δ 10.62 (s, 1H, H-1$_{\text{-oxindole}}$), 7.64 (s, 1H, H$_{\text{-alkene}}$), 7.48 (d, *J* = 8.7 Hz, 1H, H$_{\text{-aryl}}$), 7.24 (dd, *J* = 9.4, 2.8 Hz, 1H, H-4$_{\text{-oxindole}}$), 7.07 (td, *J* = 9.0, 2.7 Hz, 1H, H-6$_{\text{-oxindole}}$), 6.99 (d, *J* = 8.8 Hz, 1H, H$_{\text{-aryl}}$), 6.85 (dd, *J* = 8.6, 4.6 Hz, 1H, H-7$_{\text{-oxindole}}$), 3.89 (s, 3H, OCH_3), 3.83 (s, 3H, OCH_3), 3.80 (s, 3H, OCH_3).

^{13}C NMR (125 MHz, DMSO-d_6) δ 169.2 (C=O), 157.6 (d, J_{CF} = 235.1 Hz, C-5$_{\text{-oxindole}}$), 156.2 (=C-O), 153.0 (=C-O), 142.2 (=C-O), 139.4 (C$_{\text{-alkene}}$), 133.6 (C-7a$_{\text{-oxindole}}$), 126.6 (d, J_{CF} = 3.3 Hz, C-3$_{\text{-oxindole}}$), 125.1 (C$_{\text{-aryl}}$), 122.6 (d, *J* = 8.6 Hz, C-3a$_{\text{-oxindole}}$), 120.6 (C$_{\text{-aryl}}$), 116.5 (d, J_{CF} = 23.4 Hz, C-6$_{\text{-oxindole}}$), 111.1 (d, J_{CF} = 8.6 Hz, C-7$_{\text{-oxindole}}$), 109.7 (d, J_{CF} = 25.8 Hz, C-6$_{\text{-oxindole}}$), 108.3 (C$_{\text{-aryl}}$), 61.9 (OCH_3), 61.0 (OCH_3), 56.5 (OCH_3).

^{19}F NMR (470 MHz, DMSO-d_6) δ -121.8.

HRMS (ESI-TOF) *m/z* $[M+H]^+$ calcd. For $C_{18}H_{16}FNO_4$: 330.1136, found: 330.1134.

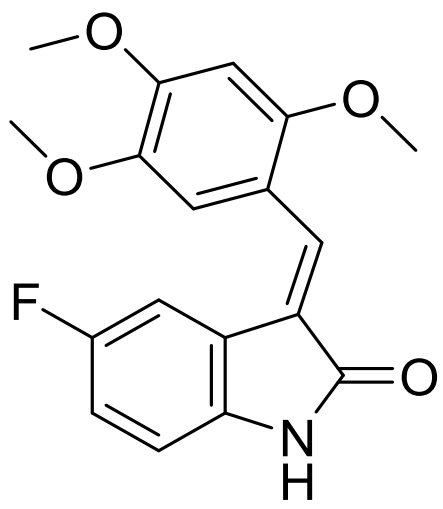

(*E*)-5-fluoro-3-(2,4,5-trimethoxybenzylidene)indolin-2-one (5e). Orange-yellow solid; yield 70%; mp: 248 °C. IR (ATR, ν_{max}, cm^{-1}): 3160, 3030, 2935, 2835, 1707, 1580, 1465.

^{1}H NMR (500 MHz, DMSO-*d$_6$*) δ_H 10.56 (s, 1H, H-1$_{\text{-oxindole}}$), 7.71 (s, 1H, H$_{\text{-alkene}}$), 7.30 (dd, *J* = 9.7, 2.7 Hz, 1H, H-4$_{\text{-oxindole}}$), 7.25 (s, 1H, H$_{\text{-aryl}}$), 7.05 (td, *J* = 8.9, 2.6 Hz, 1H, H-6$_{\text{-oxindole}}$), 6.86 – 6.82 (m, 2H), 3.90 (s, 3H, OCH_3), 3.88 (s, 3H, OCH_3), 3.71 (s, 3H, OCH_3).

^{13}C NMR (125 MHz, DMSO-*d$_6$*) δ_C 169.5 (C=O), 157.6 (d, J_{CF} = 235.1 Hz, C-5$_{\text{-oxindole}}$), 154.2 (=C-O), 153.0 (=C-O), 142.6 (=C-O), 139.2 (C$_{\text{-alkene}}$), 133.6 (C-7a$_{\text{-oxindole}}$), 125.0 (d, J_{CF} = 2.9 Hz, C-3$_{\text{-oxindole}}$), 122.9 (d, *J* = 9.1 Hz, C-3a$_{\text{-oxindole}}$), 115.9 (d, *J* = 23.4 Hz, C-6$_{\text{-oxindole}}$), 113.4 (C$_{\text{-aryl}}$), 111.0 (d, *J* = 8.1 Hz, C-7$_{\text{-oxindole}}$), 109.6 (d, *J* = 26.2 Hz, C-4$_{\text{-oxindole}}$), 98.1 (C$_{\text{-aryl}}$), 56.7, 56.6, 56.4.

^{19}F NMR (470 MHz, DMSO-*d$_6$*) δ_F -122.8.

HRMS (ESI-TOF) *m/z* $[M+H]^+$ calcd. For $C_{18}H_{16}FNO_4$: 330.1136, found: 330.1137.

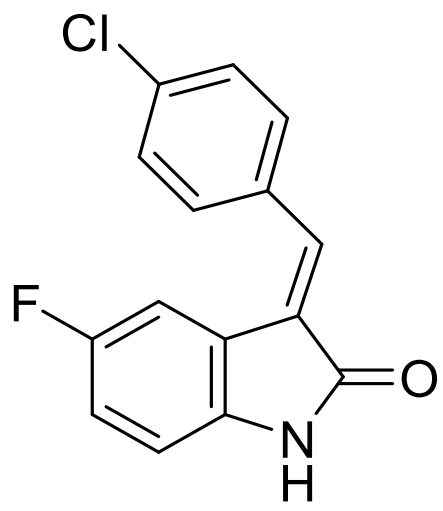

(*E*)-3-(4-chlorobenzylidene)-5-fluoroindolin-2-one (5f). Orange-yellow solid; yield 76%; mp: 242 °C. IR (ATR, ν_{max}, cm^{-1}): 3161, 3032, 2932, 2878, 1710, 1622, 1591, 1465.

^{1}H NMR (500 MHz, DMSO-*d$_6$*) δ_H 10.68 (s, 1H), 7.73 (d, *J* = 8.6 Hz, 2H), 7.66 (s, 1H), 7.61 (d, *J* = 8.6 Hz, 2H), 7.18 (dd, *J* = 9.3, 2.7 Hz, 1H), 7.10 (td, *J* = 8.9, 2.6 Hz, 1H), 6.86 (dd, *J* = 8.6, 4.5 Hz, 1H).

^{13}C NMR (125 MHz, DMSO-*d$_6$*) δ_C 168.9, 157.6 (d, *J* = 235.6 Hz), 139.9, 136.5, 135.0, 134.2, 133.4, 131.6, 129.5, 128.8, 128.3, 122.0 (d, *J* = 9.1 Hz), 117.2 (d, *J* = 23.8 Hz), 111.41 (d, *J* = 8.1 Hz), 110.1, 109.9.

^{19}F NMR (470 MHz, DMSO-*d$_6$*) δ_F -121.9.

HRMS (ESI-TOF) *m/z* $[M+H]^+$ calcd. for $C_{15}H_9ClFNO$ 274.0429, found: 274.0427.

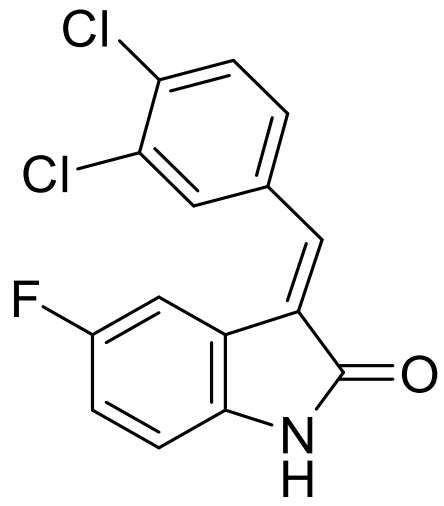

(*E*)-3-(3,4-dichlorobenzylidene)-5-fluoroindolin-2-one (5g). Orange-yellow solid; yield 58%; mp: 234 °C

^{1}H NMR (500 MHz, DMSO-d_6) δ 10.70 (s, 1H, H-1$_{\text{-oxindole}}$), 7.97 (d, J = 2.2 Hz, 1H, H$_{\text{-aryl}}$), 7.81 (d, J = 8.3 Hz, 1H, H$_{\text{-aryl}}$), 7.71 (dd, J = 8.3, 2.2 Hz, 1H, H$_{\text{-aryl}}$), 7.63 (s, 1H, H$_{\text{-alkene}}$), 7.16 – 7.11 (m, 2H, H$_{\text{-oxindole}}$), 6.87 (dd, J = 8.3, 4.5 Hz, 1H, H-7$_{\text{-oxindole}}$).

^{13}C NMR (125 MHz, DMSO-d_6) δ 168.7 (C=O), 157.62 (d, J_{CF} = 235.6 Hz, C-5$_{\text{-oxindole}}$), 140.1 (C$_{\text{-alkene}}$), 135.27 (C-7a$_{\text{-oximdole}}$),), 135.02 (C$_{\text{-aryl}}$), 133.51 (C$_{\text{-aryl}}$), 132.74 (C$_{\text{-aryl}}$), 132.16 (C$_{\text{-aryl}}$), 131.71 (C$_{\text{-aryl}}$), 131.57 (C$_{\text{-aryl}}$), 129.63 (C$_{\text{-oxindole}}$), 121.83 (d, J_{CF} = 8.6 Hz, C-3$_{\text{-oxindole}}$), 117.49 (d, J_{CF} = 23.8 Hz, C-6$_{\text{-oxindole}}$), 111.54 (d, J_{CF} = 8.6 Hz, C-7$_{\text{-oxindole}}$), 110.14 (d, J_{CF} = 25.7 Hz, C-4$_{\text{-oxindole}}$).

^{19}F NMR (470 MHz, dmso) δ -122.3.

HRMS (ESI-TOF) *m/z* $[M-H]^-$ calcd. for $C_{15}H_8Cl_2FNO$: 305.9894, found: 305.9891.

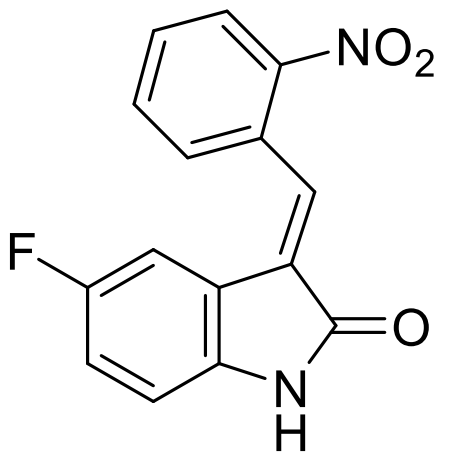

(*E*)-5-fluoro-3-(2-nitrobenzylidene)indolin-2-one (5h). Orange-yellow solid; yield 51%; mp: 238 °C. IR (ATR, ν_{max}, cm^{-1}): 3163, 3034, 2944, 2843, 1702, 1654, 1569, 1468.

^{1}H NMR (500 MHz, DMSO-d_6) δ_H 10.73 (s, 1H, H-1$_{\text{-oxindole}}$), 8.33 (d, J = 8.2 Hz, 1H, H$_{\text{-aryl}}$), 7.93 (s, 1H, H$_{\text{-alkene}}$), 7.91 (d, J = 7.6 Hz, 1H, H$_{\text{-aryl}}$), 7.85 – 7.76 (m, 2H, H$_{\text{-aryl}}$), 7.07 (td, J = 8.9, 2.7 Hz, 1H, H-4$_{\text{-oxindole}}$), 6.86 (dd, J = 8.5, 4.6 Hz, 1H, H-6$_{\text{-oxindole}}$), 6.51 (dd, J = 9.0, 2.8 Hz, 1H, H-7$_{\text{-oxindole}}$).

^{13}C NMR (125 MHz, DMSO-d_6) δ_C 168.4 (C=O), 157.54 (d, J_{CF} = 235.6 Hz, C-5$_{\text{-oxindole}}$), 147.5 (C$_{\text{-aryl}}$), 139.8 (d, J_{CF} = 1.9 Hz, C$_{\text{-alkene}}$), 135.1 (C$_{\text{-aryl}}$), 131.4 (C$_{\text{-aryl}}$), 131.3 (C$_{\text{-aryl}}$), 130.7 (C$_{\text{-aryl}}$), 128.5 (d, J = 2.9 Hz, C-3$_{\text{-oxindole}}$), 125.9 (C$_{\text{-aryl}}$), 122.0 (d, J_{CF} = 9.1 Hz, C-3a$_{\text{-oxindole}}$), 117.2 (d, J_{CF} = 23.8 Hz, C-6$_{\text{-oxindole}}$), 111.5 (d, J_{CF} = 8.1 Hz, C-4$_{\text{-oxindole}}$), 110.0 (d, J_{CF} = 25.7 Hz, C-4$_{\text{-oxindole}}$).

^{19}F NMR (470 MHz, DMSO-d_6) δ_F -122.1.

HRMS (ESI-TOF) *m/z* $[M+H]^+$ calcd. For $C_{15}H_9FN_2O_3$: 285.0670, found: 285.0665.

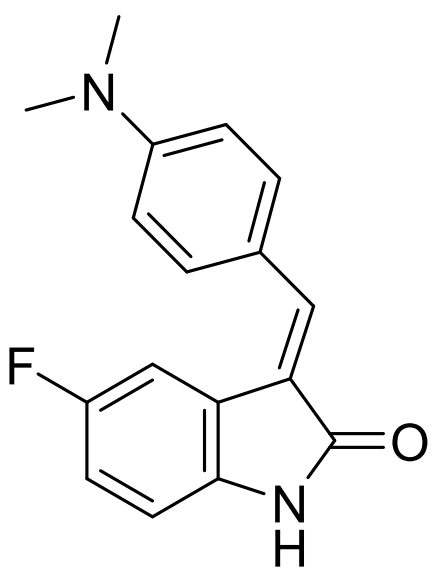

(*E*)-3-(4-(dimethylamino)benzylidene)-5-fluoroindolin-2-one (5i). Orange-red solid; yield 57%; mp: 232 °C. IR (ATR, ν_{max}, cm^{-1}): 3077, 2865, 1688, 1588, 1462.

^{1}H NMR (500 MHz, DMSO-d_6) δ_H 10.48 (s, 1H, H-1$_{-oxindole}$), 7.64 (d, *J* = 9.0 Hz, 2H, H$_{-aryl}$), 7.58 (s, 1H, H$_{-alkene}$), 7.50 (dd, *J* = 9.7, 2.7 Hz, 1H, H-4$_{-oxindole}$), 7.03 (td, *J* = 9.0, 2.6 Hz, 1H, H-6$_{-oxindole}$), 6.84 (dt, *J* = 8.6, 2.2 Hz, 3H), 3.04 (s, 6H, N(CH_3)$_2$).

^{13}C NMR (125 MHz, DMSO-d_6) δ_C 169.9 (C=O), 157.7 (d, J_{CF} = 234.1 Hz, C-5$_{-oxindole}$), 152.1 (C-N$_{-aryl}$), 139.5 (C$_{-alkene}$), 138.8 (C-7a$_{-oxindole}$), 132.6 (2C, C$_{-aryl}$), 123.3 (d, J_{CF} = 9.1 Hz, C-3a$_{-oxindole}$), 122.1 (d, J_{CF} = 2.9 Hz, C-3$_{-oxindole}$), 121.1 (C$_{-aryl}$), 115.2 (d, J_{CF} = 23.4 Hz, C-6$_{-oxindole}$), 112.0 (2C, C$_{-aryl}$), 110.7 (d, J_{CF} = 8.6 Hz, C-7$_{-oxindole}$), 109.0 (d, J_{CF} = 25.7 Hz, C-4$_{-oxindole}$), 40.1 (2C, N(CH_3)$_2$),

^{19}F NMR (470 MHz, DMSO-d_6) δ_F -122.5.

HRMS (ESI-TOF) *m/z* $[M+H]^+$ calcd. For $C_{17}H_{15}FN_2O$: 283.1241, found: 283.1246.

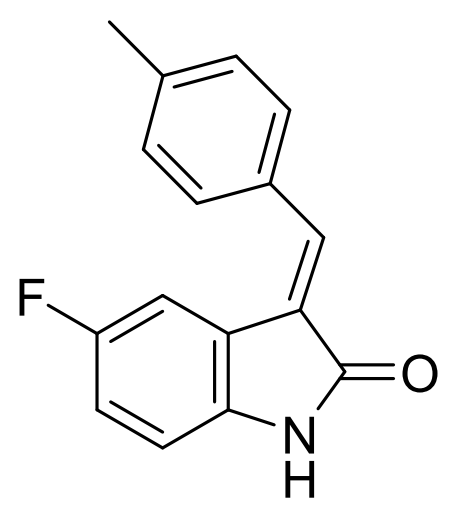

(*E*)-5-fluoro-3-(4-methylbenzylidene)indolin-2-one (5j). Orange-yellow solid; yield 94%; mp: 222 °C. IR (ATR, ν_{max}, cm^{-1}): 3155, 3062, 2878, 2838, 1691, 1511, 1468.

^{1}H NMR (500 MHz, DMSO-d_6) δ_H 10.62 (s, 1H, H-1$_{-oxindole}$), 7.67 (s, 1H, H$_{-alkene}$), 7.60 (d, *J* = 8.1 Hz, 2H, H$_{-aryl}$), 7.36 (d, *J* = 8.2 Hz, 2H, H$_{-aryl}$), 7.27 (dd, *J* = 9.4, 2.8 Hz, 1H, H-6$_{-oxindole}$), 7.08 (td, *J* = 9.0, 2.7 Hz, 1H, H-7$_{-oxindole}$), 6.86 (dd, *J* = 8.6, 4.7, 1H, H-4$_{-oxindole}$), 2.39 (s, 3H, CH_3).

^{13}C NMR (125 MHz, DMSO-d_6) δ_C 169.2, 157.6 (d, J_{CF} = 235.1 Hz), 140.7 (C$_{-aryl}$), 139.6 (C$_{-alkene}$), 138.2 (C-7a$_{-oxindole}$), 131.6 (C$_{-aryl}$), 129.9 (2C, C$_{-aryl}$), 129.9 (2C, C$_{-aryl}$), 127.1 (d, J_{CF} = 3.3 Hz, C-3$_{-oxindole}$), 122.4 (d, J_{CF} = 9.1 Hz, C-3a$_{-oxindole}$), 116.7 (d, J_{CF} = 23.4 Hz, C-6$_{-oxindole}$), 111.3 (d, J_{CF} = 8.6 Hz, C-7$_{-oxindole}$), 109.7 (d, J_{CF} = 25.8 Hz, C-4$_{-oxindole}$), 21.6 (CH_3).

19F NMR (470 MHz, DMSO-d_6) δ_F -122.2.

HRMS (ESI-TOF) *m/z* $[M+H]^+$ calcd. for $C_{16}H_{12}FNO$: 254.0976, found: 254.0980.

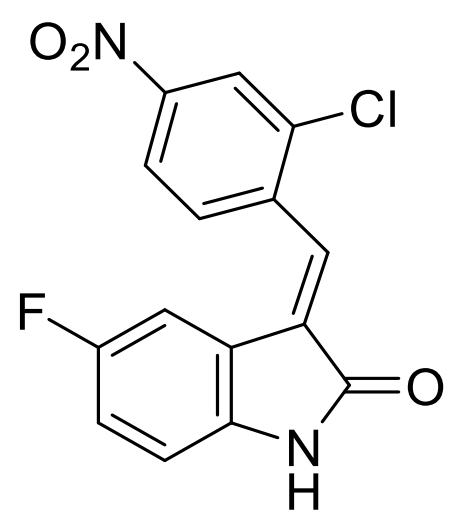

(E)-3-(2-chloro-4-nitrobenzylidene)-5-fluoroindolin-2-one (5k). Orange-yellow solid; yield 92%; mp: 258 °C. IR (ATR, ν_{max}, cm^{-1}): 3173, 3074, 3028, 2877, 1710, 1696, 1569, 1482.

^{1}H NMR (500 MHz, DMSO-d_6) δ_H 10.67 (s, 1H), 8.94 (d, *J* = 2.8 Hz, 1H, H-$_{aryl}$), 8.25 (dd, *J* = 8.8, 2.8 Hz, 1H, H-$_{aryl}$), 7.92 (s, 1H, H-$_{alkene}$), 7.85 (d, *J* = 8.8 Hz, 1H, H-$_{aryl}$), 7.76 (dd, *J* = 8.7, 2.7 Hz, 1H, H-4$_{-oxindole}$), 7.12 (td, *J* = 9.5, 2.6 Hz, H-6$_{-oxindole}$), 6.83 (dd, *J* = 8.4, 4.4 Hz, 1H, H-7$_{-oxindole}$).

^{13}C NMR (125 MHz, DMSO-d_6) δ_C 167.0, 159.4, 157.5, 146.0, 140.3, 138.7, 133.6, 131.3, 131.0, 130.9, 127.5, 125.8, 125.3, 124.9, 117.3, 117.2, 111.2, 111.1, 109.4, 109.2.

^{19}F NMR (470 MHz, DMSO-d_6) δ_F -122.0.

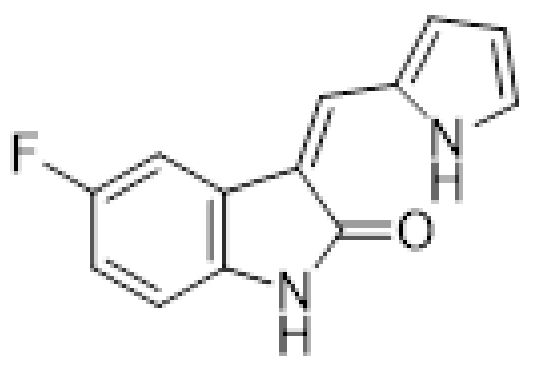

(*Z*)-3-((1H-pyrrol-2-yl)methylene)-5-fluoroindolin-2-one (5l). Orange-yellow solid; yield 98%; mp: 202 °C.

^{1}H NMR (500 MHz, DMSO-d_6) δ_H 13.37 (s, 1H, $H_{-pyrrole}$), 10.91 (s, 1H, H-$1_{-oxindole}$), 7.83 (s, 1H, $H_{-alkene}$), 7.54 (dd, J = 9.1, 2.6 Hz, 1H, H-$4_{-oxindole}$), 7.39 (q, J = 2.7 Hz, 1H, $H_{-pyrrole}$), 6.95 (ddd, J = 9.7, 8.4, 2.6 Hz, 1H, H-$7_{-oxindole}$), 6.86 – 6.82 (m, 2H), 6.37 (dd, J = 4.2, 2.0 Hz, 1H, $H_{-pyrrole}$).

^{13}C NMR (125 MHz, DMSO-d_6) δ_C 169.8 (C=O), 158.6 (d, J_{CF} = 234.6 Hz, C-$5_{-oxindole}$), 135.6 (C-$7a_{-oxindole}$), 129.9 ($C_{-alkene}$), 128.3 ($C_{-pyrrole}$), 127.2 (d, J_{CF} = 9.5 Hz, C-$3a_{-oxindole}$), 126.9 ($C_{-pyrrole}$), 121.5 ($C_{-pyrrole}$), 116.8 (d, J_{CF} = 3.3 Hz, C-$3_{-oxindole}$), 113.4 (d, J_{CF} = 24.3 Hz, C-$6_{-oxindole}$), 112.2 ($C_{-pyrrole}$), 110.6 (d, J_{CF} = 8.6 Hz, C-$7_{-oxindole}$), 106.3 (d, J_{CF} = 25.8 Hz, C-$4_{-oxindole}$)

^{19}F NMR (470 MHz, DMSO-d_6) δ_F -122.3.

HRMS (ESI-TOF) *m/z* $[M+H]^+$ calcd. for $C_{13}H_9FN_2O$: 229.0772, found: 229.0775.

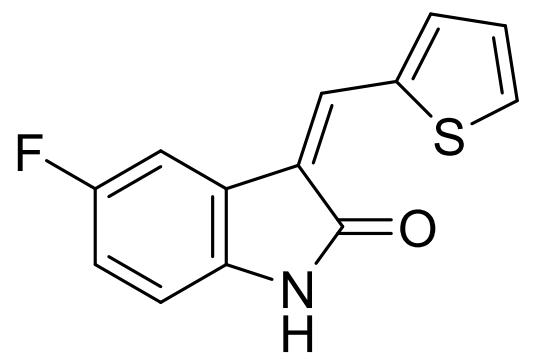

(*Z*)-5-fluoro-3-(thiophen-2-ylmethylene)indolin-2-one (**5m**). Orange-yellow solid; yield 94%; mp: 190 °C.

^{1}H NMR (500 MHz, DMSO-d_6) δ_H 10.65 (s, 1H, H-$1_{-oxindole}$), 8.03 (d, J = 5.1 Hz, 1H), 7.92 (dd, J = 9.5, 2.7 Hz, 1H), 7.88 – 7.84 (m, 2H), 7.33 (dd, J = 5.0, 3.7 Hz, 1H, H-$4_{-oxindole}$), 7.14 (td, J = 9.0, 2.7 Hz, 1H, H-$6_{-oxindole}$), 6.90 (dd, J = 8.6, 4.8 Hz, 1H, H-$7_{-oxindole}$).

^{13}C NMR (125 MHz, DMSO-d_6) δ_C 169.6 (C=O), 157.8 (d, J_{CF} = 234.6 Hz, C-$5_{-oxindole}$), 139.4 ($C_{-alkene}$), 137.4, 137.1 (C-$7a_{-oxindole}$), 133.2, 129.3, 129.2, 123.4 (d, J = 2.9 Hz, C-$3_{-oxindole}$), 122.0 (d, J = 8.6 Hz, C-$3a_{-oxindole}$), 116.5 (d, J = 23.8 Hz, C-$4_{-oxindole}$), 111.1 (d, J = 8.6 Hz, C-$7_{-oxindole}$), 110.6 (d, J = 26.2 Hz, C-$4_{-oxindole}$).

^{19}F NMR (470 MHz, DMSO-d_6) δ_F -122.6.

HRMS (ESI-TOF) *m/z* $[M+H]^+$ calcd. for $C_{13}H_8FNOS$: 246.0383, found: 246.0387.

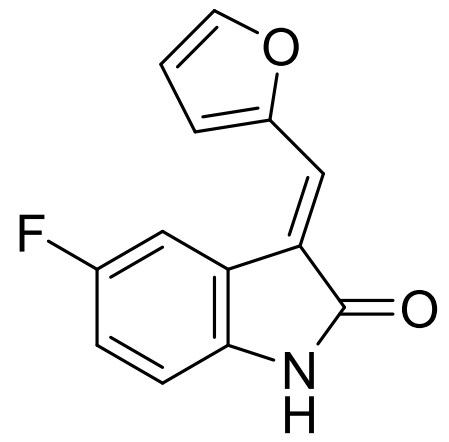

(*E*)-5-fluoro-3-(furan-2-ylmethylene)indolin-2-one (5n). Orange-yellow solid; yield 96%; mp: 212 °C.

^{1}H NMR (500 MHz, DMSO-*d6*) δ_H 10.59 (s, 1H), 8.25 (d, *J* = 2.1 Hz, 1H, H$_{-furan}$), 8.13 (dd, *J* = 9.8, 2.7 Hz, 1H, , H-4$_{-oxindole}$), 7.39 (s, 1H), 7.32 (d, *J* = 3.4 Hz, 1H, H$_{-furan}$), 7.10 (td, *J* = 9.0, 2.7 Hz, 1H, , H-6$_{-oxindole}$), 6.85 (dd, *J* = 8.5, 4.7 Hz, 1H, , H-7$_{-oxindole}$), 6.82 (dd, *J* = 3.5, 1.8 Hz, 1H, H$_{-furan}$).
^{13}C NMR (125 MHz, DMSO-*d6*) δ_C 169.7 (C=O), 158.1 (d, *J* = 234.1 Hz, C-5$_{-oxindole}$), 151.0 (C$_{-furan}$), 148.5 (C$_{-furan}$), 139.3 (C$_{-alkene}$), 122.7 (d, *J* = 9.5 Hz, C-3a$_{-oxindole}$), 122.4 (C-7a$_{-oxindole}$), 122.1 (d, *J* = 3.3 Hz, C-3$_{-oxindole}$), 121.0 (C$_{-furan}$), 116.3 (d, *J* = 23.8 Hz, C-6$_{-oxindole}$), 114.1 (C$_{-furan}$), 111.8 (d, *J* = 26.2 Hz, C-7$_{-oxindole}$), 110.7 (d, *J* = 8.6 Hz, C-4$_{-oxindole}$).
^{19}F NMR (470 MHz, DMSO-*d6*) δ_F -122.1.
HRMS (ESI-TOF) *m/z* $[M+H]^+$ calcd. for $C_{13}H_8FNO_2$: 230.0612, found: 230.0615.

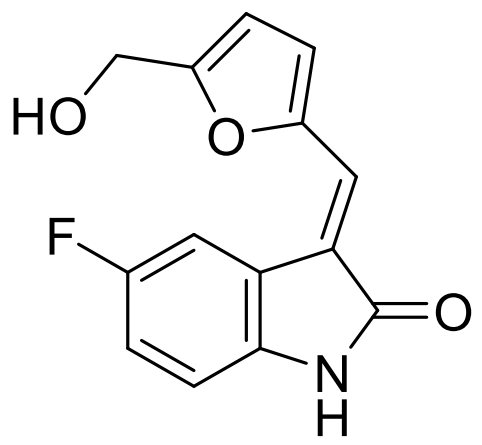

(*E*)-5-fluoro-3-((5-(hydroxymethyl)furan-2-yl)methylene)indolin-2-one (5o). Orange-yellow solid; yield 81%; mp: 262 °C. IR (ATR, ν_{max}, cm^{-1}): 3399, 3154, 3060, 2933, 2825, 1692, 1572, 1464.

^{1}H NMR (500 MHz, DMSO-*d6*) δ_H 10.56 (s, 1H, H-1$_{-oxindole}$), 8.19 (dd, *J* = 9.9, 2.8 Hz, 1H, H-4$_{-oxindole}$), 7.35 (s, 1H, H$_{-alkene}$), 7.25 (d, *J* = 3.4 Hz, 1H, H$_{-furan}$), 7.08 (td, *J* = 9.1, 2.7 Hz, 1H, H-6$_{-oxindole}$), 6.84 (dd, *J* = 8.5, 4.7 Hz, 1H, H-7$_{-oxindole}$), 6.64 (d, *J* = 3.4 Hz, 1H, H$_{-furan}$), 5.58 (t, *J* = 5.6 Hz, 1H, -OH), 4.61 (d, *J* = 5.5 Hz, 2H, CH_2).

^{13}C NMR (125 MHz, DMSO-*d6*) δ_C 169.8 (C=O), 160.9 (C$_{-furan}$), 158.17 (d, J_{CF} = 234.1 Hz, C-5$_{-oxindole}$), 150.2 (C$_{-furan}$), 139.2 (C$_{-alkene}$), 123.2 (C-7a$_{-oxindole}$), 122.8 (C-3a$_{-oxindole}$), 122.8 (C$_{-furan}$), 121.6 (d, J_{CF} = 2.9 Hz, C-3$_{-oxindole}$), 121.0 (C$_{-furan}$), 116.04 (d, *J* = 23.8 Hz, C-6$_{-oxindole}$), 112.2 (d, *J* = 26.2 Hz, C-4$_{-oxindole}$), 110.6 (d, *J* = 8.6 Hz, C-7$_{-oxindole}$) 56.6.

^{19}F NMR (470 MHz, DMSO-*d6*) δ_F -122.3.

HRMS (ESI-TOF) *m/z* $[M+H]^+$ calcd. for $C_{14}H_{10}FNO_3$: 260.0717, found: 260.0712.

Electrochemical synthesis of indolin-2-one derivatives bearing indoline-2-one-pyrrole scaffolds.

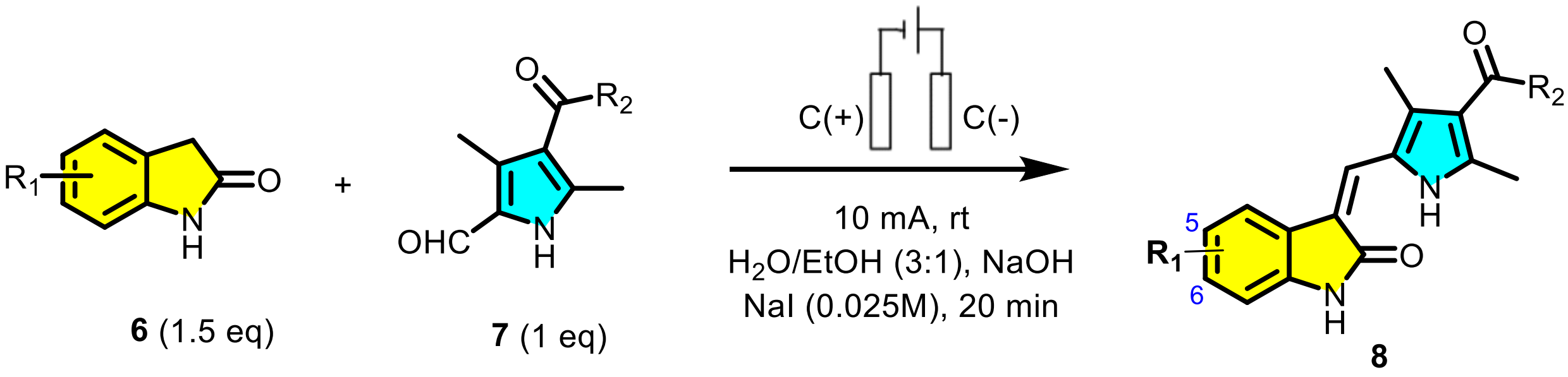

The general procedure for electro-condensation involved dissolving compounds 1 (1.5 equiv., 0.15 mmol) and aldehyde **7** (1 equiv., 0.10 mmol) in 4 mL of solvent EtOH/H_2O (1:3) and NaI (0.025 M). Carbon as anode and cathode were added, the reaction was conducted at room temperature under a constant current of 10 mA, 20 min. 20 µL of 1 M NaOH was added for synthesis compound **8k-x**. Compound **8a-j** without addition NaOH. After completion of the reaction, distilled water (5 mL) and a few drops of ethanol were added to the reaction mixture and recrystallized with ethanol to obtain pure products.

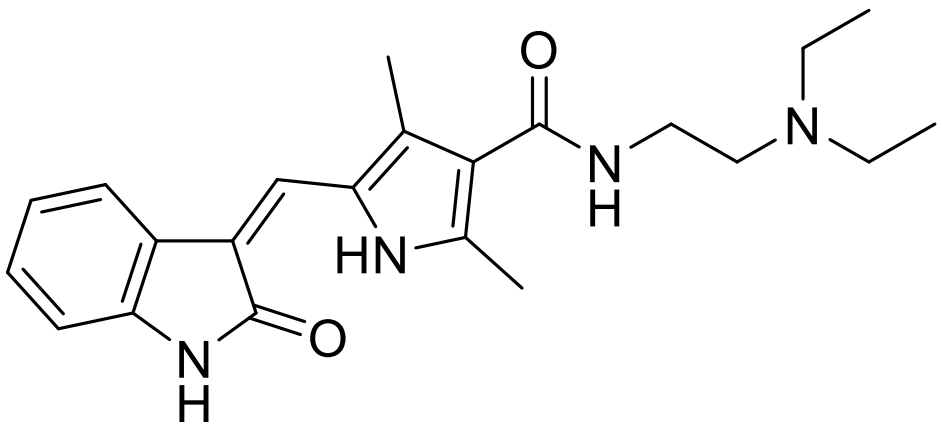

(*Z*)-*N*-(2-(diethylamino)ethyl)-2,4-dimethyl-5-((2-oxoindolin-3-ylidene)methyl)-1*H*-pyrrole-3-carboxamide (8a). Orange-yellow solid; yield 67%; mp: 228 °C.

^{1}H NMR (500 MHz, DMSO-*d*$_6$) δ_H 13.63 (s, 1H, H-1$_{\text{-pyrrole}}$), 10.90 (s, 1H, H-1$_{\text{-oxindole}}$), 7.77 (d, *J* = 7.6 Hz, 1H, H$_{\text{-oxindole}}$), 7.63 (s, 1H, H$_{\text{-alkene}}$), 7.41 (t, *J* = 5.8 Hz, 1H, H$_{\text{-oxindole}}$), 7.12 (t, *J* = 7.6 Hz, 1H, H$_{\text{-oxindole}}$), 6.99 (t, *J* = 7.4 Hz, 1H, H$_{\text{-oxindole}}$), 6.88 (d, *J* = 7.7 Hz, 1H, H$_{\text{-oxindole}}$), 3.27 (q, *J* = 6.1 Hz, 2H), 2.55 – 2.50 (m, 6H), 2.43 (s, 3H, $CH_{3\text{-pyrrole}}$), 2.40 (s, 3H, $CH_{3\text{-pyrrole}}$), 0.97 (t, *J* = 7.1 Hz, 6H, 2CH_3).

^{13}C NMR (125 MHz, DMSO-*d*$_6$) δ_C 169.9 (C=O), 165.1 (C=O), 138.9 (C$_{\text{-oxindole}}$), 136.2 (C$_{\text{-pyrrole}}$), 129.5 (C$_{\text{-pyrrole}}$), 126.9, 126.2 (C$_{\text{-pyrrole}}$), 126.0 (C$_{\text{-pyrrole}}$), 123.9, 121.5, 120.9, 119.1, 115.6, 109.9, 52.1, 47.0 (2C, 2CH_2), 37.4 (CH_2), 13.8 ($CH_{3\text{-pyrrole}}$), 12.4 (2C, 2CH_3), 11.1 ($CH_{3\text{-pyrrole}}$).
HRMS (ESI-TOF) *m/z* $[M+H]^+$ calcd. for $C_{22}H_{28}N_4O_2$: 381.2285, found: 381.2282.

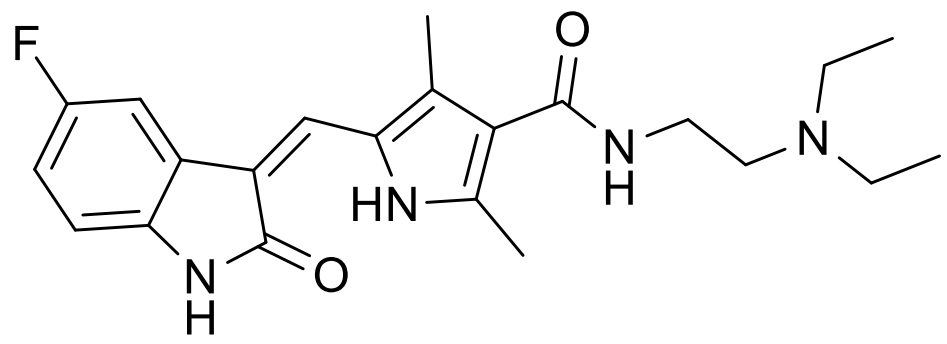

***(Z)*-*N*-(2-(diethylamino)ethyl)-5-((5-fluoro-2-oxoindolin-3-ylidene)methyl)-2,4-dimethyl-1*H*-pyrrole-3-carboxamide** (**8b**). a yellow solid product: 95% yield. Melting point : 223°C.

^{1}H NMR (500 MHz, DMSO-*d*$_6$) δ 13.68 (s, 1H, pyrrole-NH), 10.91 (s, 1H, oxindole-NH), 7.76 (dd, *J* = 9.4, 2.6 Hz, 1H), 7.71 (s, 1H, alkene-CH), 7.46 (t, *J* = 5.6 Hz, 1H), 6.92 (td, *J* = 8.8, 2.6 Hz, 1H), 6.83 (dd, *J* = 8.5, 4.5 Hz, 1H), 3,27 (q, *J* = 6.5 Hz, 2H), 2.53 (t, *J* = 7.1 Hz, 2H),

2.48 (m, 4H), 2.43 (s, 3H, pyrrole-CH_3), 2.41 (s, 3H, pyrrole-CH_3), 0.96 (t, J = 7.1 Hz, 6H, $N(CH_2C\mathit{H_3})_2$).

^{13}C NMR (125 MHz, DMSO-d_6) δ 170.0 (C=O), 165.0 (C=O), 158.7 (d, J_{CF} = 234.1 Hz, Ar-C), 137.1 ($C_{-pyrrole}$), 134.9 ($C_{-oxindole}$), 130.7 ($C_{-pyrrole}$), 127.6 (d, J_{CF} = 9.6 Hz, $C_{-oxindole}$), 126.2 ($C_{-pyrrole}$), 125.3 ($C_{-pyrrole}$), 121.2 ($C_{-pyrrole}$), 115.0 (d, J_{CF} = 2.7 Hz, $C_{-oxindole}$), 112.8 (d, J_{CF} = 23.9 Hz, $C_{-oxindole}$), 110.5 (d, J_{CF} = 8.5 Hz, $C_{-oxindole}$), 106.4 (d, J_{CF} = 25.4 Hz, $C_{-oxindole}$), 52.1 ($NHCH_2\mathit{C}H_2$), 46.9 ($N(\mathit{C}H_2CH_3)_2$), 37.4 ($NH\mathit{C}H_2CH_2$), 13.8 ($CH_{3\text{-pyrrole}}$), 12.3, 11.1 ($CH_{3\text{-pyrrole}}$).

^{19}F NMR (470 MHz, DMSO-d_6) δ 122.2.

HRMS (ESI-TOF): calculated for $C_{22}H_{27}FN_4O_2$ $[M-H]^-$: 397,2045; Found: 397,2046.

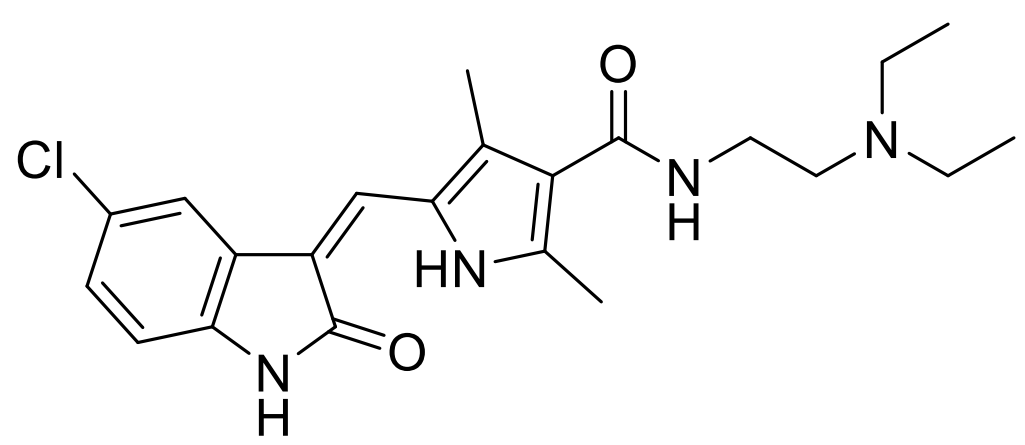

(*Z*)-5-((5-chloro-2-oxoindolin-3-ylidene)methyl)-*N*-(2-(diethylamino)ethyl)-2,4-dimethyl-1*H*-pyrrole-3-carboxamide (**8c**). Yellow solid; yield 73%; mp: 224 °C. IR (ATR, ν_{max}, cm^{-1}): 3147, 2966, 2869, 1675, 1556, 1466. 1255, 1027.

^{1}H NMR (500 MHz, DMSO-d_6) δ_H 13.63 (s, 1H, H-$1_{-pyrrole}$), 11.00 (s, H, H-$1_{-oxindole}$), 7.98 (d, J = 2.1 Hz, 1H, $H_{-oxindole}$), 7.76 (s, 1H, $H_{-alkene}$), 7.45 (t, J = 5.7 Hz, 1H, H_{-amide}), 7.12 (dd, J = 8.2, 2.1 Hz, 1H, $H_{-oxindole}$), 6.86 (d, J = 8.2 Hz, 1H, $H_{-oxindole}$), 3.27 (q, J = 6.1 Hz, 2H, CH_2), 2.52-2.54 (m, 6H, $3CH_2$), 2.43 (3H, $CH_{3\text{-pyrrole}}$), 2.42 (3H, $CH_{3\text{-pyrrole}}$) 0.97 (t, J = 7.1 Hz, 6H, $2CH_3$).

^{13}C NMR (125 MHz, DMSO-d_6) δ_C 169.8 (C=O), 164.9 (C=O), 137.3 (C-$2_{-pyrrole}$), 137.2 ($C_{-oxindole}$), 131.0 ($C_{-oxindole}$), 128.0 ($C_{-pyrrole}$), 126.4 ($C_{-oxindole}$), 126.0 ($C_{-pyrrole}$), 126.0 ($C_{-oxindole}$), 125.6 ($CH_{-alkene}$), 121.3 ($C_{-pyrrole}$), 119.1 ($C_{-oxindole}$), 114.2 ($C_{-oxindole}$), 111.1 ($C_{-oxindole}$), 52.1 (CH_2), 40.3 (2C, $2CH_2$), 37.4 (CH_2), 13.8 ($CH_{3\text{-pyrrole}}$), 12.4, 11.1 ($CH_{3\text{-pyrrole}}$).

HRMS (ESI-TOF) *m/z* $[M+H]^+$ calcd. for $C_{22}H_{27}ClN_4O_2$: 415.1895, found: 415.1891

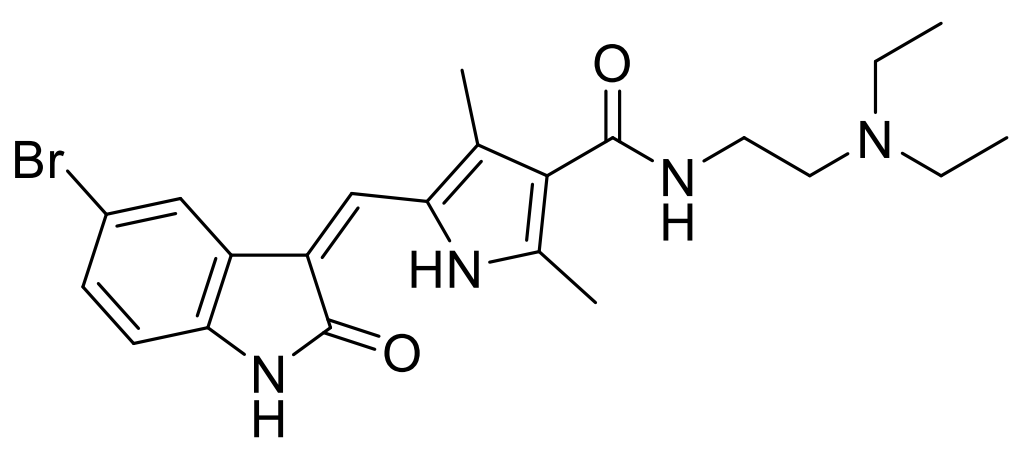

(*Z*)-5-((5-bromo-2-oxoindolin-3-ylidene)methyl)-*N*-(2-(diethylamino)ethyl)-2,4-dimethyl-1*H*-pyrrole-3-carboxamide (**8d**). Orange-yellow solid; yield 51%; mp: 242 °C.

^{1}H NMR (500 MHz, , DMSO-d_6) δ_H 13.63 (s, 1H, H-$1_{-pyrrole}$), 11.01 (s, 1H, H-$1_{-oxindole}$), 8.11 (d, J = 2.0 Hz, 1H, $H_{-oxindole}$), 7.77 (s, 1H, $H_{-alkene}$), 7.45 (t, J = 6.3 Hz, 1H), H_{-amide}), 7.25 (dd, J = 8.3, 2.0 Hz, 1H, $H_{-oxindole}$), 6.82 (d, J = 8.2 Hz, 1H, $H_{-oxindole}$), 3.27 (t, J = 6.6 Hz, 2H, CH_2),

2.50-2.53 (m, 6H, 3CH_2), 2.43 (s, 3H, $CH_{3\text{-pyrrole}}$), 2.42 (s, 3H, $CH_{3\text{-pyrrole}}$), 0.93 (t, *J* = 7.1 Hz, 6H, 2CH_3).

^{13}C NMR (125 MHz, dmso) δ 169.6 (C-2$_{\text{-oxindole}}$), 165.9 (C=O), 137.7 (C$_{\text{-pyrrole}}$), 137.3 (C-7a$_{\text{-oxindole}}$), 131.0 (C$_{\text{-oxindole}}$), 128.8 (C$_{\text{-oxindole}}$), 128.4 (C$_{\text{-pyrrole}}$), 126.4 (C$_{\text{-pyrrole}}$), 125.7 (C$_{\text{-alkene}}$), 121.8 (C$_{\text{-oxindole}}$), 121.3 (C$_{\text{-pyrrole}}$), 114.0 (C$_{\text{-oxindole}}$), 113.9 (C$_{\text{-oxindole}}$), 111.6 (C$_{\text{-oxindole}}$), 52.1 (NHCH_2*C*H_2N),, 47.0 (2C, N(*C*$H_2CH_3)_2$), 37.4 (CH_2), 13.8 ($CH_{3\text{-pyrrole}}$), 12.3 (2C, , N(CH_2*C*$H_3)_2$), 11.1 ($CH_{3\text{-pyrrole}}$).

HRMS (ESI-TOF) *m/z* $[M+H]^+$ calcd. for $C_{22}H_{27}BrN4O_2$: 459.1390, found: 459.1399.

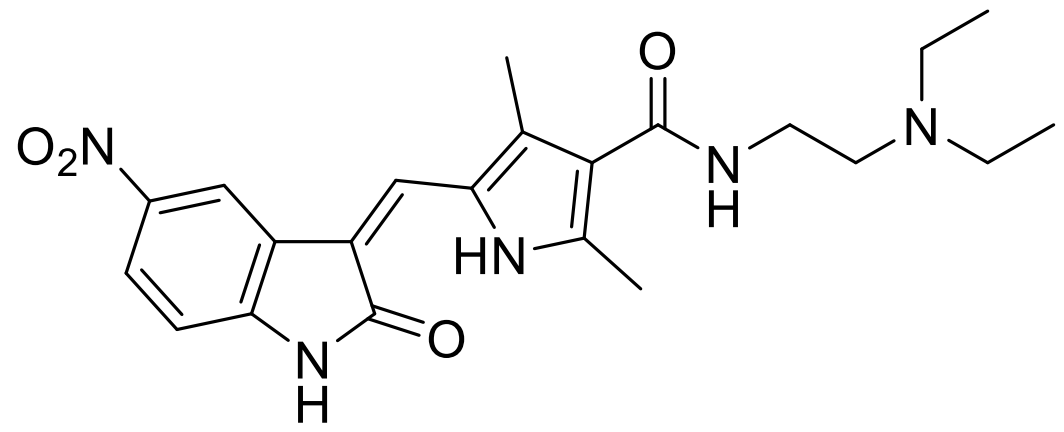

(*Z*)-*N*-(2-(diethylamino)ethyl)-2,4-dimethyl-5-((5-nitro-2-oxoindolin-3-ylidene)methyl)-1*H*-pyrrole-3-carboxamide (**8e**). Orange-yellow solid; yield 60%; mp: 252 °C. IR (ATR, ν_{max}, cm^{-1}): 3436, 3059, 2928, 2869, 1677, 1566, 1458. 1225, 1027.

^{1}H NMR (500 MHz, DMSO-*d$_6$*) δ_H 13.55 (s, 1H, H-1$_{\text{-pyrrole}}$), 11.49 (s, 1H, H-1$_{\text{-oxindole}}$), 8.84 (d, *J* = 2.4 Hz, 1H), 8.05 (dd, *J* = 8.6, 2.3 Hz, 1H), 8.01 (s, 1H, H$_{\text{-alkene}}$), 7.49 (t, *J* = 5.5 Hz, 1H, H$_{\text{-amide}}$), 7.05 (d, *J* = 8.6 Hz, 1H), 3.28 (d, *J* = 6.6 Hz, 2H, CH_2), 2.53 (dd, *J* = 13.3, 6.9 Hz, 6H, 3CH_2), 2.47 (s, 3H $CH_{3\text{-pyrrole}}$), 2.45 (s, 3H, $CH_{3\text{-pyrrole}}$), 0.97 (t, *J* = 7.1 Hz, 6H, 2CH_3).

^{13}C NMR (125 MHz, DMSO-*d$_6$*) δ_C 170.4, 164.8, 143.8, 142.7, 138.4, 132.5, 127.2, 126.9, 126.6, 122.8, 121.8, 114.9, 112.8, 109.7, 52.1, 47.0 (2C), 37.5, 13.8, 12.3 (2C), 11.1.

HRMS (ESI-TOF) *m/z* $[M+H]^+$ calcd. for $C_{22}H_{27}N_5O_4$: 426.2136, found: 426.2134.

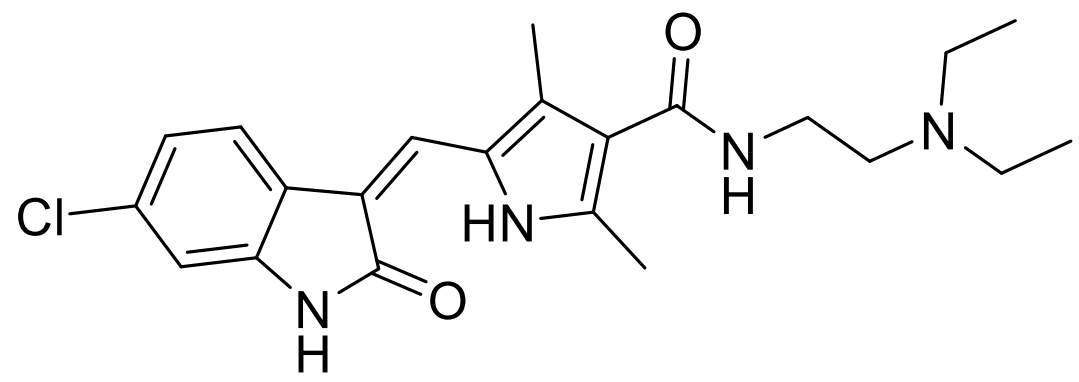

(*Z*)-5-((6-chloro-2-oxoindolin-3-ylidene)methyl)-*N*-(2-(diethylamino)ethyl)-2,4-dimethyl-1*H*-pyrrole-3-carboxamide (**8f**). Orange-yellow solid; yield 51%; mp: 260 °C.

^{1}H NMR (500 MHz, DMSO-*d$_6$*) δ_H 13.53 (s, 1H, H-1$_{\text{-pyrrole}}$), 11.03 (s, 1H, H-1$_{\text{-oxindole}}$), 7.82 (d, *J* = 8.2 Hz, 1H, H$_{\text{-oxindole}}$), 7.68 (s, 1H, H$_{\text{-alkene}}$), 7.44 (t, *J* = 6.0 Hz, 1H, H$_{\text{-amide}}$), 7.03 (d, *J* = 8.1 Hz, 1H, H$_{\text{-oxindole}}$), 6.88 (s, 1H, H$_{\text{-oxindole}}$), 3.27 (q, *J* = 6.5 Hz, 2H, CH_2), 2.56 – 2.51 (m, 4H, 2CH_2), 2.49 – 2.46 (m, 2H, CH_2), 2.43 (s, 3H, $CH_{3\text{-pyrrole}}$), 2.41 (s, 3H, $CH_{3\text{-pyrrole}}$), 0.97 (t, *J* = 7.1 Hz, 6H, 2CH_3).

^{13}C NMR (125 MHz, DMSO-*d$_6$*) δ_C 169.9 (C=O), 164.9 (C=O), 139.8 (C$_{\text{-oxindole}}$), 136.9 (C$_{\text{-pyrrole}}$), 130.7 (C$_{\text{-oxindole}}$), 130.5 (C$_{\text{-oxindole}}$), 126.3 (C$_{\text{-pyrrole}}$), 125.0 (CH$_{\text{-alkene}}$), 124.8 (C$_{\text{-pyrrole}}$), 121.3 (C$_{\text{-oxindole}}$), 121.2 (C$_{\text{-pyrrole}}$), 120.5 (C$_{\text{-oxindole}}$), 114.2 (C$_{\text{-oxindole}}$), 109.8 (C$_{\text{-oxindole}}$), 52.1 (CH_2), 47.0 (2C, CH_2), 39.5, 37.4 (CH_2), 13.8 ($CH_{3\text{-pyrrole}}$), 12.4 (2C, CH_3), 11.1 ($CH_{3\text{-pyrrole}}$).

HRMS (ESI-TOF) *m/z* $[M+H]^+$ calcd. for $C_{22}H_{27}ClN_4O_2$: 415.1895, found: 415.1899.

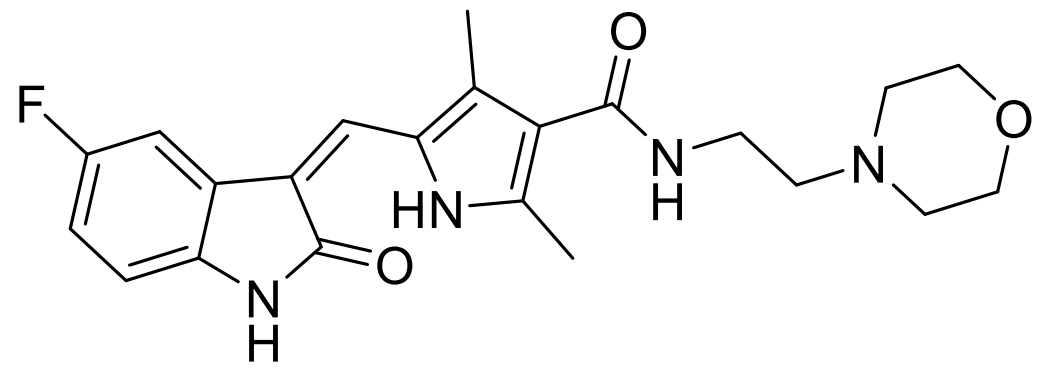

(*Z*)-5-((5-fluoro-2-oxoindolin-3-ylidene)methyl)-2,4-dimethyl-*N*-(2-morpholinoethyl)-1*H*-pyrrole-3-carboxamide (**8g**). Orange-yellow solid; yield 55%; mp: 280 °C

^{1}H NMR (500 MHz, DMSO-d_6) δ_H 13.67 (s, 1H, H-1$_{\text{-pyrrole}}$), 10.90 (s, 1H, H-1$_{\text{-oxindole}}$), 7.76 (dd, *J* = 9.4, 2.5 Hz, 1H, H$_{\text{-oxindole}}$), 7.71 (s, 1H, H$_{\text{-alkene}}$), 7.50 (t, *J* = 5.6 Hz, 1H, H$_{\text{-amide}}$), 6.92 (td, *J* = 9.0, 2.6 Hz, 1H, H$_{\text{-oxindole}}$), 6.84 (dd, *J* = 8.4, 4.5 Hz, 1H, H$_{\text{-oxindole}}$), 3.57 (t, *J* = 4.6 Hz, 4H, 2CH$_{2\text{-morpholine}}$), 3.34 – 3.30 (m, 2H, 2CH$_2$), 2.46 – 2.41 (m, 12H, 2CH$_{3\text{-pyrrole}}$, 2CH$_{2\text{-morpholine}}$, CH$_2$).

^{13}C NMR (125 MHz, DMSO-d_6) δ_C 170.0 (C=O), 165.0 (C=O), 158.7 (d, J_{CF} = 234.1 Hz, C-5$_{\text{-oxindole}}$), 137.0 (C$_{\text{-pyrrole}}$), 135.0 (C-7$_{\text{-oxindole}}$), 130.7 (C$_{\text{-pyrrole}}$), 127.6 (d, J_{CF} = 9.5 Hz, C-3a$_{\text{-oxindole}}$), 126.3 (C$_{\text{-pyrrole}}$), 125.4 (CH$_{\text{-alkene}}$), 121.3 (C$_{\text{-pyrrole}}$), 115.0 (d, J_{CF} = 3.3 Hz, C-3$_{\text{-oxindole}}$), 112.8 (d, J_{CF} = 24.3 Hz, C-6$_{\text{-oxindole}}$), 110.5 (d, J_{CF} = 8.6 Hz, C-7$_{\text{-oxindole}}$), 106.4 (d, J_{CF} = 25.7 Hz, C-4$_{\text{-oxindole}}$), 66.7 (2C, 2CH$_2$), 57.7, 53.6 (2C, 2CH$_3$), 36.2 (CH$_2$), 13.7 (CH$_{3\text{-pyrrole}}$), 11.0 (CH$_{3\text{-pyrrole}}$).

^{19}F NMR (470 MHz, DMSO-d_6) δ_F -122.5.

HRMS (ESI-TOF) *m/z* [M+H]$^+$ calcd. for $C_{22}H_{25}FN_4O_3$: 413.1983, found:413.1987.

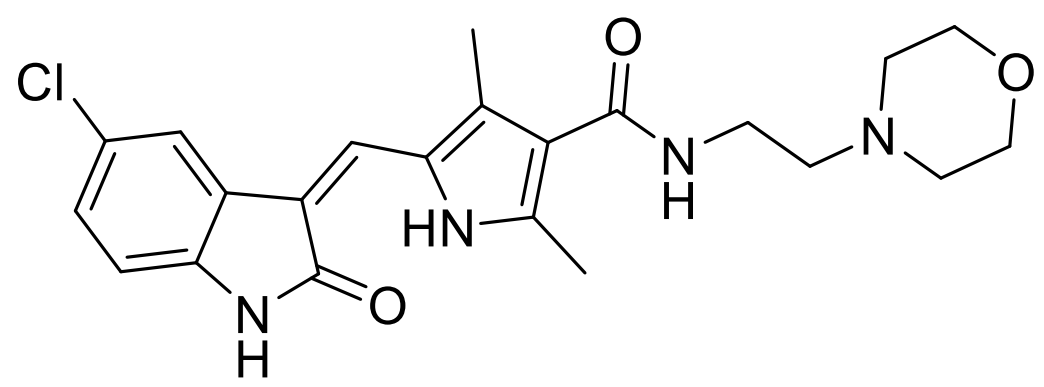

(*Z*)-5-((5-chloro-2-oxoindolin-3-ylidene)methyl)-2,4-dimethyl-*N*-(2-morpholinoethyl)-1*H*-pyrrole-3-carboxamide (8h). Orange-yellow solid; yield 65%; mp: 268 °C. IR (ATR, ν_{max}, cm^{-1}): 3288, 3148, 2941, 2861, 1667, 1553, 1476. 1239, 1034.

^{1}H NMR (500 MHz, DMSO-d_6) δ_H 13.63 (s, 1H, H-1$_{\text{-pyrrole}}$), 11.00 (s, 1H, H-1$_{\text{-oxindole}}$), 7.98 (d, *J* = 2.2 Hz, H$_{\text{-oxindole}}$), 7.77 (s, 1H, H$_{\text{-alkene}}$), 7.51 (t, *J* = 5.6 Hz, 1H, NH$_{\text{-amide}}$), 7.13 (dd, *J* = 8.6, 2.3 Hz, 1H, H$_{\text{-oxindole}}$), 6.87 (d, *J* = 8.3 Hz, 1H, H$_{\text{-oxindole}}$), 3.58 (t, *J* = 4.4 Hz, 4H, 2CH$_{2\text{-morpholine}}$), 3.50 (s, 2H, CH$_2$), 2.44 (s, 3H, CH$_{3\text{-pyrrole}}$), 2.46-2.43 (m, 12H, 2CH$_{3\text{-pyrrole}}$, 2CH$_{2\text{-morpholine}}$, CH$_2$).

^{13}C NMR (125 MHz, DMSO-d_6) δ_C 169.8 (C=O), 164.9 (C=O), 137.3 (C-2$_{\text{-pyrrole}}$), 137.2 (C$_{\text{-oxindole}}$), 131.0 (C$_{\text{-oxindole}}$), 128.0 (C$_{\text{-pyrrole}}$), 126.4 (C$_{\text{-oxindole}}$), 126.1 (C$_{\text{-pyrrole}}$), 126.0 (C$_{\text{-oxindole}}$), 125.6 (C$_{\text{-alkene}}$), 121.4 (C$_{\text{-pyrrole}}$), 119.1 (C$_{\text{-oxindole}}$), 114.2 (C$_{\text{-oxindole}}$), 111.1 (C$_{\text{-oxindole}}$), 66.7 (2C, 2CH$_2$), 57.7 (CH$_2$), 53.6 (2C, 2CH$_2$), 36.2 (CH$_2$), 13.7 (CH$_{3\text{-pyrrole}}$), 11.0, (CH$_{3\text{-pyrrole}}$).

HRMS (ESI-TOF) *m/z* [M+H]$^+$ calcd. for $C_{22}H_{25}ClN_4O_3$: 429.1688, found: 429.1682.

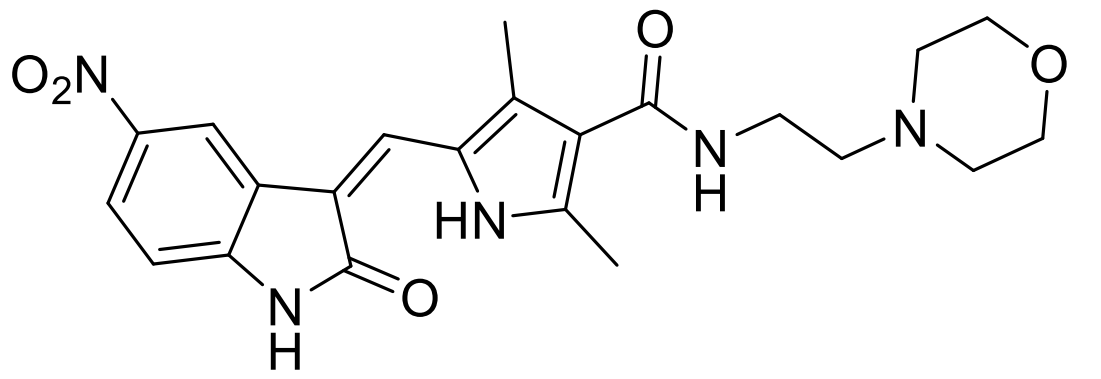

(*Z*)-2,4-dimethyl-*N*-(2-morpholinoethyl)-5-((5-nitro-2-oxoindolin-3-ylidene)methyl)-1*H*-pyrrole-3-carboxamide (8i). Orange-yellow solid; yield 57%; mp: 318 °C.

^{1}H NMR (500 MHz, DMSO-*d$_6$*) δ_{H} 13.55 (s, 1H, H$_{\text{-pyrrole}}$), 11.55 (s, 1H, H$_{\text{-pyrrole}}$), 8.87 (d, *J* = 2.3 Hz, 1H, H$_{\text{-oxindole}}$), 8.06 (dd, *J* = 8.6, 2.3 Hz, 1H, H$_{\text{-oxindole}}$), 8.04 (s, 1H, H$_{\text{-alkene}}$), 7.58 (s, 1H, H$_{\text{-amide}}$), 7.04 (d, *J* = 8.5 Hz, 1H, H$_{\text{-oxindole}}$), 3.58-3.54 (m, 4H, 2CH$_{\text{2-morpholine}}$), 3.38 (s, 2H, CH$_2$), 2.46-2.42 (m, 12H, 2CH$_{\text{3-pyrrole}}$, 2CH$_{\text{2-morpholine}}$, CH$_2$).

^{13}C NMR (125 MHz, DMSO-*d$_6$*) δ_{C} 170.4 (C=O), 164.7 (C=O), 143.7 (C$_{\text{-oxindole}}$), 142.7(C$_{\text{-oxindole}}$), 138.4 (C$_{\text{-pyrrole}}$), 132.6 (C$_{\text{-oxindole}}$), 127.3 (C$_{\text{-pyrrole}}$), 126.9 (C$_{\text{-pyrrole}}$), 126.7 (C$_{\text{-alkene}}$), 122.8 (C$_{\text{-oxindole}}$), 122.0 (C$_{\text{-pyrrole}}$), 115.0 (C$_{\text{-oxindole}}$), 112.8 (C$_{\text{-oxindole}}$), 109.7 (C$_{\text{-oxindole}}$), 66.7 (2C, CH$_{\text{2-morpholine}}$), 57.7 (CH$_2$), 53.6 (2C, CH$_{\text{2-morpholine}}$), 36.3 (CH$_2$), 13.8 (CH$_{\text{3-pyrrole}}$), 11.1 (CH$_{\text{3-pyrrole}}$).

HRMS (ESI-TOF) *m/z* [M+H]$^+$ calcd. for $C_{22}H_{25}N_5O_5$: 440.1928, found: 440.1924.

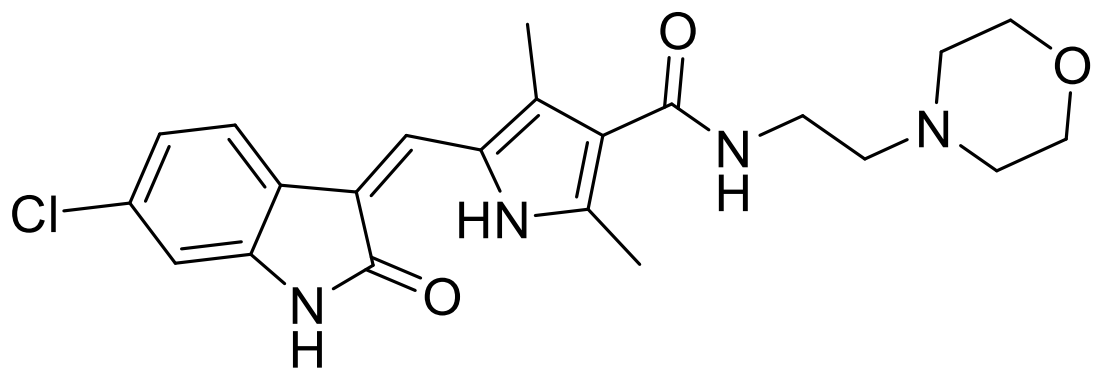

(*Z*)-5-((6-chloro-2-oxoindolin-3-ylidene)methyl)-2,4-dimethyl-*N*-(2-morpholinoethyl)-1*H*-pyrrole-3-carboxamide (8j). Orange-yellow solid; yield 93%; mp: 290 °C.

^{1}H NMR (500 MHz, DMSO-*d$_6$*) δ_{H} 13.51 (s, 1H, H$_{\text{-pyrrole}}$), 11.04 (s, 1H, H$_{\text{-oxindole}}$), 7.80 (d, *J* = 8.2 Hz, 1H, H$_{\text{-oxindole}}$), 7.67 (s, 1H, H$_{\text{-alkene}}$), 7,54 (t, *J* = 5,1, 1H, H$_{\text{-amide}}$), 7.03 (dd, *J* = 8.2, 2.1 Hz, 1H, H$_{\text{-oxindole}}$), 6.90 (d, *J* = 2.1 Hz, 1H, H$_{\text{-oxindole}}$), 3.57 (t, *J* = 4.6 Hz, 4H, 2CH$_{\text{2-morpholine}}$), 3.34 (d, *J* = 5.2 Hz, 2H, CH$_2$), 2.43 – 2.40 (m, 12H, 2CH$_{\text{3-pyrrole}}$, 2CH$_{\text{2-morpholine}}$, & CH$_2$).

^{13}C NMR (125 MHz, DMSO-*d$_6$*) δ_{C} 169.9 (C=O), 164.9 (C=O), 139.8 (C$_{\text{-oxindole}}$), 136.9 (C$_{\text{-pyrrole}}$), 130.7 (C$_{\text{-oxindole}}$), 130.5 (C$_{\text{-oxindole}}$), 126.3 (C$_{\text{-pyrrole}}$), 125.0 (C$_{\text{-alkene}}$), 124.8 (C$_{\text{-pyrrole}}$), 121.3 (C$_{\text{-pyrrole}}$), 120.5 (C$_{\text{-oxindole}}$), 114.2 (C$_{\text{-oxindole}}$), 109.8 (C$_{\text{-oxindole}}$), 66.7 (2C, CH$_2$), 57.7 (CH$_2$), 53.6 (2C, CH$_2$), 36.2 (CH$_2$), 13.7 (CH$_{\text{3-pyrrole}}$), 11.0 (CH$_{\text{3-pyrrole}}$).

HRMS (ESI-TOF) *m/z* [M+H]$^+$ calcd. for $C_{22}H_{25}ClN_4O_3$: 429.1682, found: 429.1693.

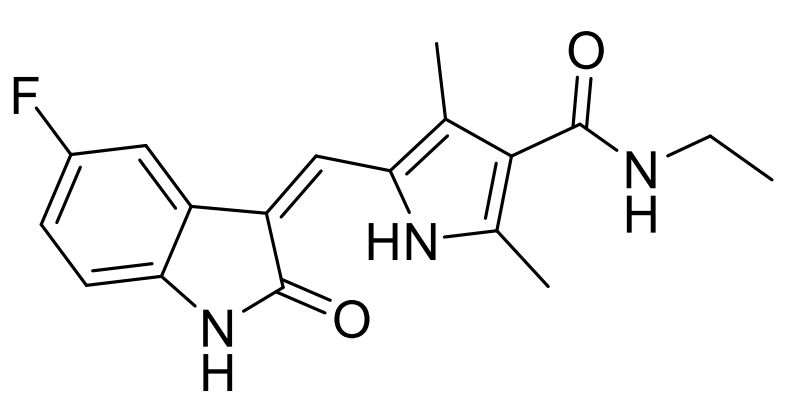

(Z)-*N*-ethyl-5-((5-fluoro-2-oxoindolin-3-ylidene)methyl)-2,4-dimethyl-1*H*-pyrrole-3-carboxamide (**8k**). Orange solid; yield 82%; mp: 298 °C.

^{1}H NMR (500 MHz, DMSO-*d$_6$*) δ 13.66 (s, 1H, H-1$_{\text{-pyrrole}}$), 10.87 (s, 1H, H-1$_{\text{-oxindole}}$), 7.74 (dd, *J* = 9.4, 2.6 Hz, 1H, H$_{\text{-oxindole}}$), 7.70 (s, 1H, H$_{\text{-alkene}}$), 7.62 (t, *J* = 5.7 Hz, 1H, N*H*CH$_2$,CH$_3$), 6.91

(ddd, J = 9.5, 8.4, 2.6 Hz, 1H, $H_{-oxindole}$), 6.84 (dd, J = 8.5, 4.5 Hz, 1H, $H_{-oxindole}$), 3.27 – 3.22 (m, 2H, $NHCH_2$,CH_3), 2.42 (s, 3H, $CH_{3\text{-pyrrole}}$), 2.40 (s, 3H, $CH_{3\text{-pyrrole}}$), 1.11 (t, J = 7.2 Hz, 3H, $NHCH_2$,CH_3).

^{13}C NMR (125 MHz, DMSO-d_6) δ 170.0 (C=O), 164.9 (C=O), 158.2 (d, J_{CF} = 234.6 Hz), 136.8, 135.0, 130.7, 127.2 (d, J_{CF} = 9.5 Hz), 126.2, 125.3, 121.5, 114.9 (d, J_{CF} = 2.5 Hz), 112.3 (d, J_{CF} = 24.2 Hz), 110.0 (d, J_{CF} = 8.6 Hz), 105.9 (d, J = 25.6 Hz), 34.0 ($NHCH_2$,CH_3), 15.5 ($NHCH_2$,CH_3), 13.7 ($CH_{3\text{-pyrrole}}$), 10.9 ($CH_{3\text{-pyrrole}}$).

^{19}F NMR (470 MHz, DMSO-d_6) δ_F -122.2.

HRMS (ESI-TOF) *m/z* $[M-H]^-$ calcd. for $C_{18}H_{18}FN_3O_2$: 326.1310, found: 326.1309.

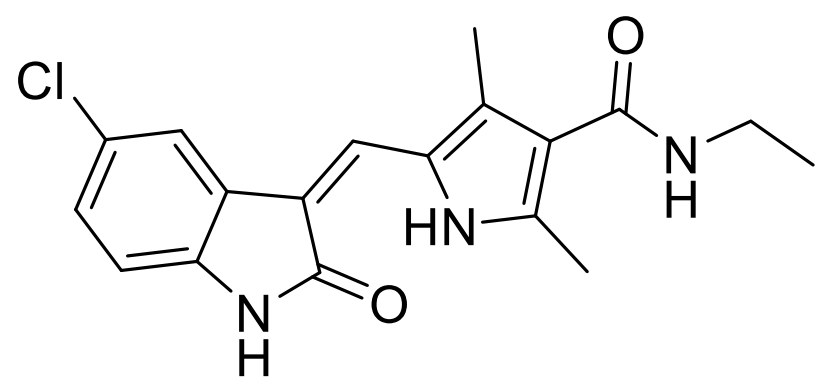

(*Z*)-5-((5-chloro-2-oxoindolin-3-ylidene)methyl)-*N*-ethyl-2,4-dimethyl-1*H*-pyrrole-3-carboxamide (8l). Orange-yellow solid; yield 58%; mp: 294 °C.

^{1}H NMR (500 MHz, DMSO-d_6) δ_H 13.63 (s, 1H, H-$1_{-pyrrole}$), 10.99 (s, 1H, H-$1_{-oxindole}$), 7.98 (d, J = 2.4 Hz, 1H, $H_{-oxindole}$), 7.77 (s, 1H, $H_{-alkene}$), 7.65 (t, J = 5.4 Hz, 1H, H_{-amide}), 7.13 (dd, J = 8.1, 2.0, 1H, $H_{-oxindole}$), 6.87 (d, J = 8.1 Hz, 1H, $H_{-oxindole}$), 3.25 – 3.22 (m, 2H, CH_2), 2.42 (s, 3H, $CH_{3\text{-pyrrole}}$), 2.41 (s, 3H, $CH_{3\text{-pyrrole}}$), 1.11 (t, J = 7.2 Hz, 3H, CH_3).

^{13}C NMR (125 MHz, DMSO-d_6) δ_C 169.8 (C=O), 165.0 (C=O), 137.3, 134.8, 131.1, 128.0, 126.3, 126.1, 126.0, 125.6, 121.9, 119.0, 114.1, 111.2, 34.1 (CH_2), 15.5(CH_3), 13.7 ($CH_{3\text{-pyrrole}}$), 10.9 ($CH_{3\text{-pyrrole}}$).

HRMS (ESI-TOF) *m/z* $[M+H]^+$ calcd. for $C_{18}H_{18}ClN_3O_2$: 344.1160, found: 334.1169.

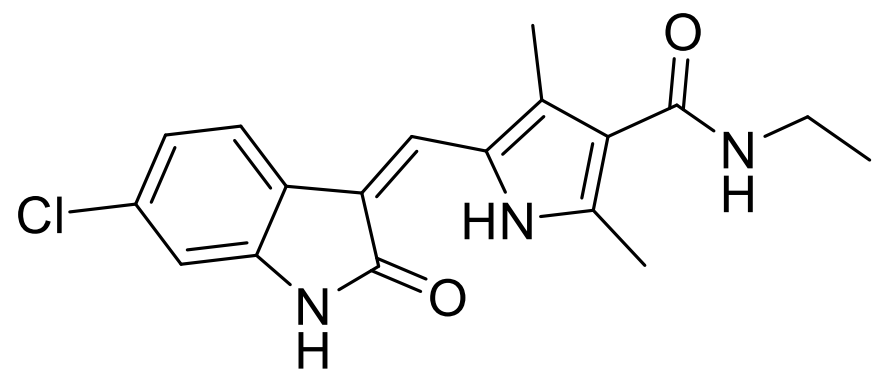

(*Z*)-5-((6-chloro-2-oxoindolin-3-ylidene)methyl)-*N*-ethyl-2,4-dimethyl-1*H*-pyrrole-3-carboxamide (8m). Orange-yellow solid; yield 85%; mp: 298 °C.

^{1}H NMR (500 MHz, DMSO-d_6) δ_H 13.52 (s, 1H, H-$1_{-pyrrole}$), 11.03 (s, 1H, $H_{-oxindole}$), 7.82 (d, J = 8.2 Hz, 1H, $H_{-oxindole}$), 7.68 (s, 1H, $H_{-alkene}$), 7.64 (t, J = 5.7 Hz, 1H, H_{-amide}), 7.03 (dd, J = 8.1, 2.0 Hz, 1H, $H_{-oxindole}$), 6.88 (d, J = 2.2 Hz, 1H, $H_{-oxindole}$), 3.23 (q, J = 6.8 Hz, 2H, CH_2), 2.42 (s, 3H, $CH_{3\text{-pyrrole}}$), 2.39 (s, 3H, $CH_{3\text{-pyrrole}}$), 1.11 (t, J = 7.2 Hz, 3H, CH_3).

^{13}C NMR (125 MHz, DMSO-d_6) δ_C 169.9 (C=O), 164.9 (C=O), 139.8 ($C_{-oxindole}$), 136.7 ($C_{-pyrrole}$), 130.7 ($C_{-oxindole}$), 130.5 ($C_{-oxindole}$), 126.3 ($C_{-pyrrole}$), 125.0 ($C_{-alkene}$), 124.8 ($C_{-pyrrole}$), 121.5 ($C_{-oxindole}$), 121.2 ($C_{-pyrrole}$), 120.4 ($C_{-oxindole}$), 114.1 ($C_{-oxindole}$), 109.8 ($C_{-oxindole}$), 34.0 (CH_2), 15.5 (CH_3), 13.7 ($CH_{3\text{-pyrrole}}$), 11.0 ($CH_{3\text{-pyrrole}}$).

HRMS (ESI-TOF) *m/z* $[M+H]^+$ calcd. for $C_{18}H_{18}ClN_3O_2$: 344.1160, found: 344.1157.

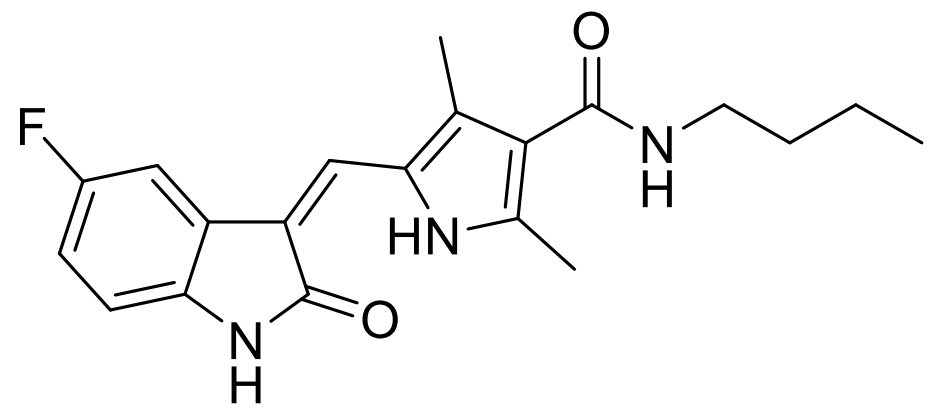

(*Z*)-*N*-butyl-5-((5-fluoro-2-oxoindolin-3-ylidene)methyl)-2,4-dimethyl-1*H*-pyrrole-3-carboxamide (8n). Orange-yellow solid; yield 75%; mp: 286 °C.
^{1}H NMR (500 MHz, DMSO-d_6) δ_H 13.66 (s, 1H, H-1$_{\text{-pyrrole}}$), 10.89 (s, 1H, H-1$_{\text{-oxindole}}$), 7.74 (dd, *J* = 9.4, 2.6 Hz, 1H), 7.71 (s, 1H, H$_{\text{-alkene}}$), 7.63 (t, *J* = 5.3 Hz, 1H, NH$_{\text{-amide}}$), 6.92 (t, *J* = 9.0 Hz, 1H, , H-6$_{\text{-oxindole}}$), 6.84 (dd, *J* = 8.3, 4.4 Hz, 1H, , H-7$_{\text{-oxindole}}$), 3.21 (q, *J* = 7.1 Hz, 2H, CH_2), 2.42 (s, 3H, CH$_{3\text{-pyrrole}}$), 2.40 (s, 3H, CH$_{3\text{-pyrrole}}$), 1.49 (p, *J* = 7.0 Hz, 2H, CH_2), 1.35 (dt, *J* = 13.9, 7.1 Hz, 2H, CH_2), 0.91 (t, *J* = 7.4 Hz, 3H, CH_3).

^{13}C NMR (125 MHz, DMSO-d_6) δ_C 170.0 (C-2$_{\text{-oxindole}}$), 165.1 (C=O), 158.7 (d, J_{CF} = 234.2 Hz, C-5$_{\text{-oxindole}}$), 136.7 (C-2$_{\text{-pyrrole}}$), 134.9 (C-7a$_{\text{-oxindole}}$), 130.7 (C-4$_{\text{-pyrrole}}$), 127.6 (d, J_{CF} = 9.5 Hz, C-3a$_{\text{-oxindole}}$), 126.2 (C-5$_{\text{-pyrrole}}$), 125.3 (C$_{\text{-alkene}}$), 121.7 (C-3$_{\text{-pyrrole}}$), 114.9 (d, J_{CF} = 2.9 Hz, C-3$_{\text{-oxindole}}$), 112.8 (d, J_{CF} = 24.2 Hz, C-6$_{\text{-oxindole}}$), 110.5 (d, J_{CF} = 8.6 Hz, C-7$_{\text{-oxindole}}$), 106.3 (d, J_{CF} = 25.2 Hz, C-4$_{\text{-oxindole}}$), 38.8 (NH*C*H$_2$CH$_2$CH$_2$CH$_3$), 31.9 (NHCH$_2$*C*H$_2$CH$_2$CH$_3$), 20.2 (NHCH$_2$CH$_2$*C*H$_2$CH$_3$), 14.2 (NHCH$_2$CH$_2$CH$_2$*C*H$_3$), 13.7 (CH$_{3\text{-pyrrole}}$), 10.9(CH$_{3\text{-pyrrole}}$).

^{19}F NMR (470 MHz, DMSO-d_6) δ -122.6.

HRMS (ESI-TOF) *m/z* $[M-H]^-$ calcd. for $C_{20}H_{22}FN_3O_2$: 354.1623, found: 354.1626.

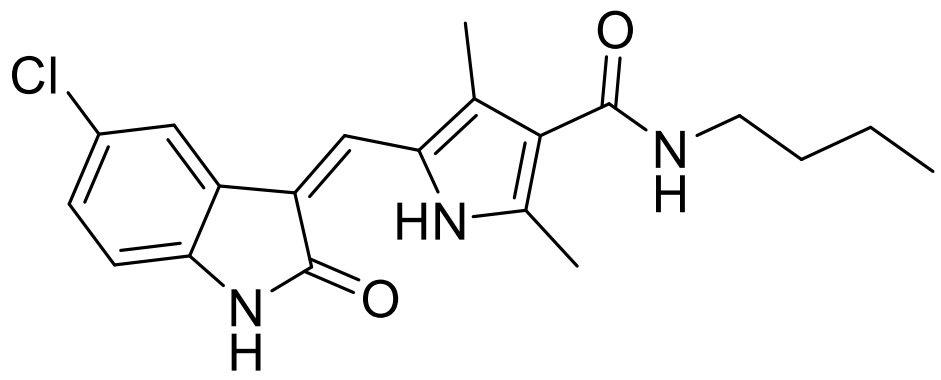

(*Z*)-*N*-butyl-5-((5-chloro-2-oxoindolin-3-ylidene)methyl)-2,4-dimethyl-1*H*-pyrrole-3-carboxamide (**8o**). Orange-yellow solid; yield 59%; mp: 286 °C. IR (ATR, ν_{max}, cm^{-1}): 3142, 2927, 2857, 1673, 1559, 1464. 1256.

^{1}H NMR (500 MHz, DMSO-d_6) δ_H 13.62 (s, 1H, H-1$_{\text{-pyrrole}}$), 10.99 (s, 1H, H-1$_{\text{-oxindole}}$), 7.98 (d, *J* = 2.1 Hz, 1H, H$_{\text{-oxindole}}$), 7.76 (s, 1H, H$_{\text{-alkene}}$), 7.64 (t, *J* = 5.7 Hz, 1H, H$_{\text{-amide}}$), 7.13 (dd, *J* = 8.5, 2.0 Hz, 1H, H$_{\text{-oxindole}}$), 6.87 (d, *J* = 8.2 Hz, 1H, H$_{\text{-oxindole}}$), 3.21 (q, *J* = 6.7 Hz, 2H, CH_2), 2.42 (s, 3H, CH$_{3\text{-pyrrole}}$), 2.40 (s, 3H, CH$_{3\text{-pyrrole}}$), 1.49 (p, *J* = 7.3 Hz, 2H, CH_2), 1.34 (q, *J* = 7.3 Hz, 2H, CH_2), 0.91 (t, *J* = 7.3 Hz, 3H, CH_3).

^{13}C NMR (125 MHz, DMSO-d_6) δ_C 169.8 (C=O), 165.0 (C=O), 137.3 (C$_{\text{-pyrrole}}$), 136.9 (C$_{\text{-oxindole}}$), 131.0 (C$_{\text{-oxindole}}$), 128.0 (C$_{\text{-pyrrole}}$), 126.3 (C$_{\text{-oxindole}}$), 126.0 (C$_{\text{-pyrrole}}$), 126.0 (C$_{\text{-oxindole}}$), 125.6 (C$_{\text{-alkene}}$), 121.8 (C$_{\text{-pyrrole}}$), 119.1 (C$_{\text{-oxindole}}$), 114.1 (C$_{\text{-oxindole}}$), 111.1 (C$_{\text{-oxindole}}$), 38.8 (CH_2), 31.9 (CH_2), 20.2 (CH_2), 14.2 (CH_3), 13.7 (CH_3), 10.9 (CH_3),

HRMS (ESI-TOF) *m/z* $[M+H]^+$ calcd. for $C_{20}H_{22}ClN_3O_2$: 372.1473, found: 372.1467.

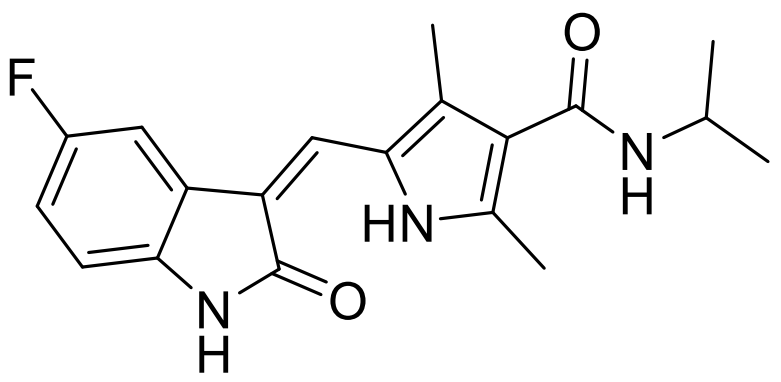

(*Z*)-5-((5-fluoro-2-oxoindolin-3-ylidene)methyl)-*N*-isopropyl-2,4-dimethyl-1*H*-pyrrole-3-carboxamide (**8p**). Yellow solid; yield 80%; mp: 282 °C.

^{1}H NMR (500 MHz, DMSO-*d₆*) δ_H 13.65 (s, 1H, H-1$_{\text{-pyrrole}}$)), 10.89 (s, 1H, H-1$_{\text{-oxindole}}$), 7.75 (dd, *J* = 9.4, 2.6 Hz, 1H, H-4$_{\text{-oxindole}}$), 7.70 (s, 1H, H$_{\text{-alkene}}$), 7.52 (d, *J* = 7.9 Hz, 1H, H$_{\text{-amide}}$), 6.92 (td, *J* = 9.0, 2.6 Hz, 1H, H-6$_{\text{-oxindole}}$), 6.84 (dd, *J* = 8.4, 4.6 Hz, 1H, H-7$_{\text{-oxindole}}$), 4.04 (dq, *J* = 13.2, 6.6 Hz, 1H, CH), 2.41 (s, 3H, CH$_{3\text{-pyrrole}}$), 2.39 (s, 3H, CH$_{3\text{-pyrrole}}$), 1.14 (d, *J* = 6.6 Hz, 6H, 2CH$_3$).

^{13}C NMR (125 MHz, DMSO-d_6) δ_C 170.0 (C=O), 164.3 (C=O), 158.7 (d, *J* = 234.1 Hz, C-5$_{\text{-oxindole}}$), 136.7 (C$_{\text{-pyrrole}}$), 134.9 (C-7a$_{\text{-oxindole}}$), 130.8 (C$_{\text{-pyrrole}}$), 127.6 (d, *J* = 10.0 Hz, C-3a$_{\text{-oxindole}}$), 126.2 (C-5$_{\text{-pyrrole}}$), 125.4 (CH$_{\text{-alkene}}$), 121.9 (C$_{\text{-pyrrole}}$), 114.8 (d, *J* = 2.9 Hz, C-3$_{\text{-oxindole}}$), 112.8 (d, *J* = 23.8 Hz, C-6$_{\text{-oxindole}}$), 110.5 (d, *J* = 8.6 Hz, C-7$_{\text{-oxindole}}$), 106.4 (d, *J* = 25.3 Hz, C-4$_{\text{-oxindole}}$), 40.9 (CH), 23.0 (2C, 2CH$_3$), 13.6 (CH$_{3\text{-pyrrole}}$), 10.8 (CH$_{3\text{-pyrrole}}$).

^{19}F NMR (470 MHz, DMSO-d_6) δ_F -122.6.

HRMS (ESI-TOF) *m/z* $[M+H]^+$ calcd. for $C_{19}H_{20}FN_3O_2$: 342.1612, found: 342.1616.

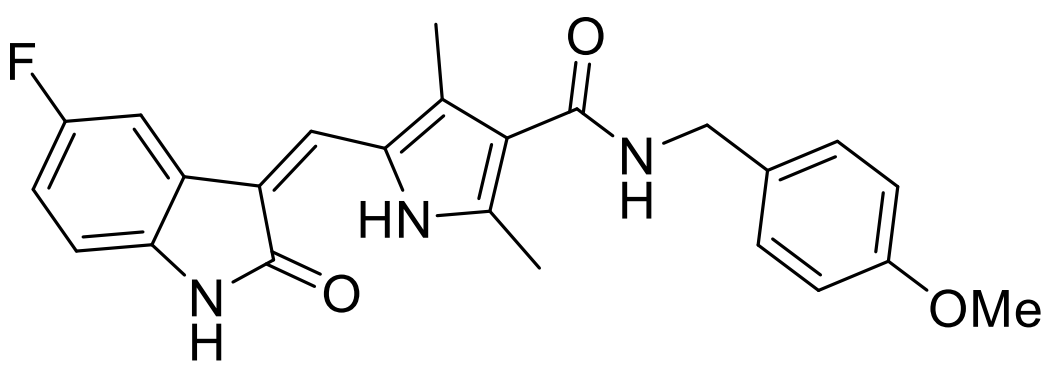

(*Z*)-5-((5-fluoro-2-oxoindolin-3-ylidene)methyl)-*N*-(4-methoxybenzyl)-2,4-dimethyl-1*H*-pyrrole-3-carboxamide (**8q**). Yellow solid; yield 75%; mp: 308 °C.

^{1}H NMR (500 MHz, DMSO-d_6) δ_H 13.68 (s, 1H), 10.89 (s, 1H), 8.11 (t, *J* = 6.0 Hz, 1H), 7.75 (dd, *J* = 9.3, 2.5 Hz, 1H), 7.71 (s, 1H), 7.28 – 7.24 (m, 2H), 6.95 – 6.89 (m, 3H), 6.84 (dd, *J* = 8.4, 4.5 Hz, 1H), 4.37 (d, *J* = 6.0 Hz, 2H), 3.73 (s, 3H), 2.43 (s, 3H), 2.41 (s, 3H).

^{13}C NMR (125 MHz, DMSO-d_6) δ_C 170.0, 165.1, 158.7 (d, *J* = 234.0 Hz), 158.6, 137.0, 135.0, 132.4, 130.8, 129.0 (2C), 127.6 (d, *J* = 9.4 Hz), 126.3, 125.4, 121.2, 115.1, 114.1 (2C), 112.8 (d, *J* = 24.6 Hz), 110.5 (d, J_{CF} = 8.7 Hz), 106.4 (d, J_{CF} = 25.5 Hz), 55.5 (Ar-O*C*H$_3$), 42.2 (NH*C*H$_2$Ar), 13.8 (CH$_{3\text{-pyrrole}}$), 11.0 (CH$_{3\text{-pyrrole}}$).

^{19}F NMR (470 MHz, DMSO-d_6) δ_F -122.2.

HRMS (ESI-TOF) m/z $[M+H]^+$ calcd. for $C_{24}H_{22}FN_3O_2$: 402.1623, found: 402.1633.

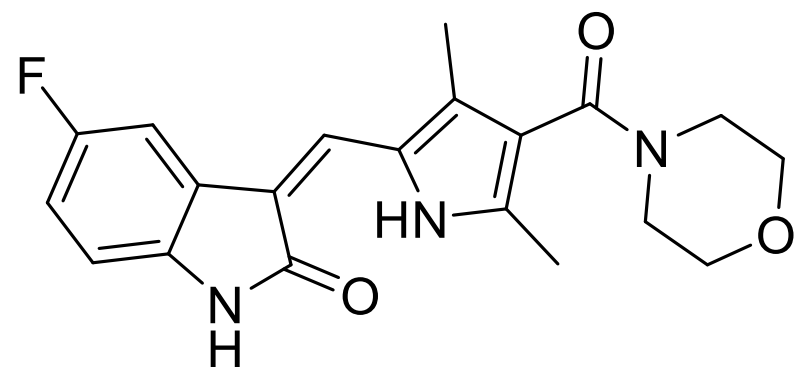

(*Z*)-3-{[3,5-Dimethyl-4-(morpholine-4-carbonyl)-1*H*-pyrrol-2-yl]methylene}-5-fluoroindolin-2-one (8r). Yellow solid; yield 86%; mp: 262 °C.

^{1}H NMR (500 MHz, DMSO-d_6) δ_H 13.63 (s, 1H, H-1$_{\text{-pyrrole}}$), 10.82 (s, 1H, H-1$_{\text{-oxindole}}$), 7.73 (d, *J* = 9.2 Hz, 1H, H-4$_{\text{-oxindole}}$, $^3J_{HF}$ = 9.2 Hz), 7.69 (s, 1H, CH$_{\text{-alkene}}$), 6.92 (t, *J* = 9.2 Hz, 1H, H-6$_{\text{-oxindole}}$, $^3J_{HF}$ = 9.2 Hz), 6.84 (dd, *J* = 8.7, 4.5 Hz, 1H, H-7$_{\text{-oxindole}}$, $^4J_{HF}$ = 4.5 Hz), 3.57-3.48 (m, 8H, 4CH$_{2\text{-morpholine}}$), 2.30 (s, 3H, CH$_{3\text{-pyrrole}}$), 2.27 (s, 3H, CH$_{3\text{-pyrrole}}$).

^{13}C NMR (125 MHz, DMSO-d_6) δ_C 170.0 (C-2$_{\text{-oxindole}}$), 165.9 (C=O), 158.7 (d, J_{CF} = 234.2 Hz, C-5$_{\text{-oxindole}}$), 135.0 (C-2$_{\text{-pyrrole}}$), 134.9 (C-7a$_{\text{-oxindole}}$), 129.7 (C-4$_{\text{-pyrrole}}$), 127.6 (C-3a$_{\text{-oxindole}}$), 126.5 (C-5$_{\text{-pyrrole}}$), 125.2 (C$_{\text{-alkene}}$), 120.3 (C-3$_{\text{-pyrrole}}$), 115.1 (C-3$_{\text{-oxindole}}$), 112.9 (d, J_{CF} = 24.2 Hz, C-6$_{\text{-oxindole}}$), 110.5 (d, J_{CF} = 8.6 Hz, C-7$_{\text{-oxindole}}$), 106.4 (d, J_{CF} = 25.6 Hz, C-4$_{\text{-oxindole}}$), 66.9 (C-2$_{\text{-morpholine}}$), 66.9 (C-6$_{\text{-morpholine}}$), 40.2 (C-5$_{\text{-morpholine}}$), 40.0 (C-3$_{\text{-morpholine}}$), 12.9 (CH$_{3\text{-pyrrole}}$), 10.5 (CH$_{3\text{-pyrrole}}$).

^{19}F NMR (470 MHz, DMSO-d_6) δ_F -122.5 (td, *J* = 9.5, 4.6 Hz).

HRMS (ESI-TOF) *m/z* $[M+H]^+$ calcd. for $C_{20}H_{20}FN_3O_3$: 368.1416, found: 368.1409.

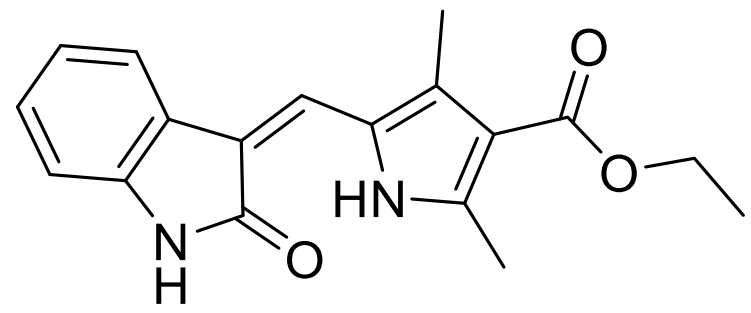

Ethyl (Z)-2,4-dimethyl-5-((2-oxoindolin-3-ylidene)methyl)-1H-pyrrole-3-carboxylate (8s). Yellow solid; yield 79%; mp: 244 °C.

^{1}H NMR (500 MHz, DMSO-d_6) δ_H 13.90 (s, 1H), 10.98 (s, 1H), 7.81 (d, *J* = 7.3 Hz, 1H), 7.68 (s, 1H), 7.18 – 7.11 (m, 1H), 7.00 (td, *J* = 7.6, 1.1 Hz, 1H), 6.88 (d, *J* = 7.6 Hz, 1H), 4.20 (q, *J* = 7.2 Hz, 2H), 2.54 (s, 3H, CH$_{3\text{-pyrrole}}$), 2.46 (s, 3H, CH$_{3\text{-pyrrole}}$), 1.30 (t, *J* = 7.1 Hz, 3H).
^{13}C NMR (125 MHz, DMSO-d_6) δ_C 170.0, 164.9, 140.6, 139.1, 132.3, 127.4, 126.5, 125.7 123.7, 121.7, 119.5, 117.1, 113.8, 110.0, 59.6 (OCH_2), 15.0 (CH_3), 14.8 (CH$_{3\text{-pyrrole}}$), 11.9 (CH$_{3\text{-pyrrole}}$).
HRMS (ESI-TOF) *m/z* $[M-H]^-$ calcd. for $C_{18}H_{18}N_2O_3$: 309.1245, found: 309.1249.

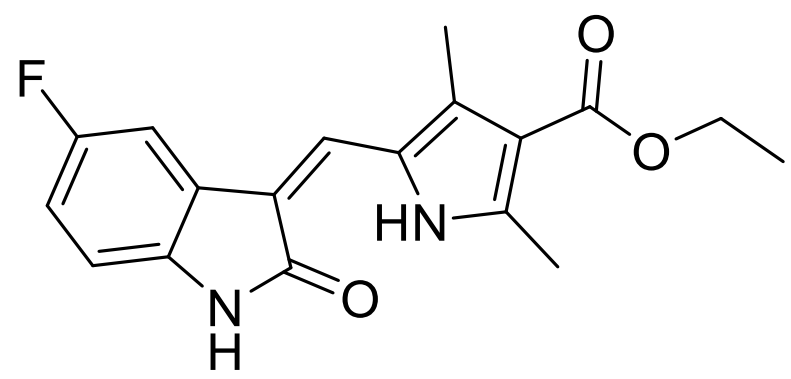

Ethyl (*Z*)-5-((5-fluoro-2-oxoindolin-3-ylidene)methyl)-2,4-dimethyl-1*H*-pyrrole-3-carboxylate (4). Yellow solid; yield 94 %; melting point: 282 °C.

^{1}H NMR (500 MHz, DMSO-d_6) δ_H 13.90 (s, 1H), 10.96 (s, 1H), 7.78 (dd, *J* = 9.4, 2.6 Hz, 1H), 7.72 (s, 1H), 6.93 (ddd, *J* = 9.5, 8.4, 2.6 Hz, 1H), 6.83 (dd, *J* = 8.4, 4.5 Hz, 1H), 4.19 (q, *J* = 7.1 Hz, 2H), 2.52 (s, 3H, $CH_{3\text{-pyrrole}}$), 2.49 (s, 3H, $CH_{3\text{-pyrrole}}$), 1.29 (t, *J* = 7.1 Hz, 3H).

^{13}C NMR (125 MHz, DMSO-d_6) δ_C 170.0, 164.7, 158.7 (d, J_{CF} = 234.6 Hz), 141.2, 135.2, 133.4, 127.4 (d, *J* = 9.5 Hz), 126.6, 125.2, 116.51 (d, *J* = 3.3 Hz), 114.0, 113.3 (d, *J* = 24.3 Hz), 110.6 (d, *J* = 8.6 Hz), 106.8 (d, *J* = 25.3 Hz), 59.6, 15.0 (CH_3), 14.7 ($CH_{3\text{-pyrrole}}$), 11.9 ($CH_{3\text{-pyrrole}}$).

^{19}F NMR (470 MHz, DMSO-d_6) δ_F -122.3.

HRMS (ESI-TOF) *m/z* $[M+H]^+$ calcd. for $C_{18}H_{17}FN_2O_3$: 329.1296, found: 329.1289.

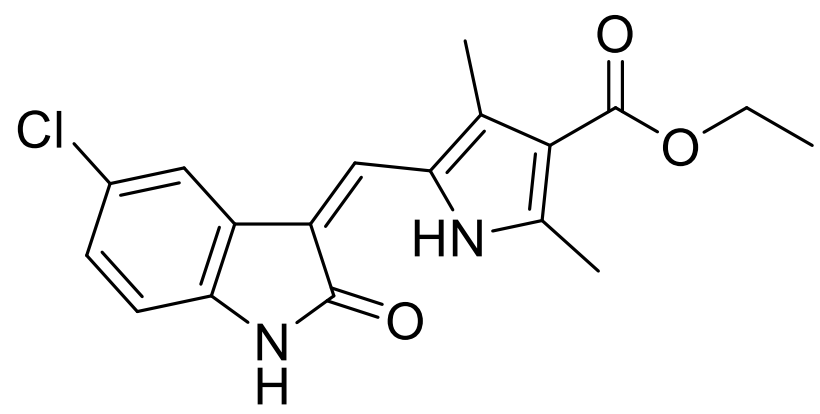

Ethyl (*Z*)-5-((5-chloro-2-oxoindolin-3-ylidene)methyl)-2,4-dimethyl-1*H*-pyrrole-3-carboxylate (**8t**). Orange-yellow solid; yield 82%; mp: 272 °C. IR (ATR, ν_{max}, cm^{-1}): 3267, 2982, 2902, 1681, 1574, 1471. 1252, 1066.

^{1}H NMR (500 MHz, DMSO-d_6) δ_H 13.87 (s, 1H, $H_{\text{-pyrrole}}$), 11.06 (s, 1H, H-$1_{\text{-oxindole}}$), 8.01 (d, *J* = 2.1 Hz, 1H, $H_{\text{-oxindole}}$), 7.80 (s, 1H, $H_{\text{-alkene}}$), 7.14 (dd, *J* = 8.2, 2.0 Hz, 1H, $H_{\text{-oxindole}}$), 6.86 (d, *J* = 8.2 Hz, 1H, $H_{\text{-oxindole}}$), 4.20 (q, *J* = 7.1 Hz, 2H, OCH_2), 2.53 (s, 3H, $CH_{3\text{-pyrrole}}$), 2.50 (s, 3H, $CH_{3\text{-pyrrole}}$), 1.30 (t, *J* = 7.1 Hz, 3H, CH_3).

^{13}C NMR (125 MHz, DMSO-d_6) δ_C 169.8 (C=O), 164.7 (C=O), 141.3 ($C_{\text{-pyrrole}}$), 137.6 ($C_{\text{-oxindole}}$), 133.8 ($C_{\text{-alkene}}$), 127.7 ($C_{\text{-oxindole}}$), 126.7 ($C_{\text{-oxindole}}$), 126.5 ($C_{\text{-pyrrole}}$), 126.2 ($C_{\text{-oxindole}}$), 125.5 ($CH_{\text{-pyrrole}}$), 119.5 ($C_{\text{-oxindole}}$), 115.8 ($C_{\text{-oxindole}}$), 114.1 ($C_{\text{-pyrrole}}$), 111.2 ($C_{\text{-oxindole}}$), 59.7 (CH_2), 15.0 (CH_3), 14.7 ($CH_{3\text{-pyrrole}}$), 11.9 ($CH_{3\text{-pyrrole}}$).

HRMS (ESI-TOF) *m/z* $[M+H]^+$ calcd. for $C_{18}H_{17}ClN_2O_3$: 345.1000, found: 345.0996.

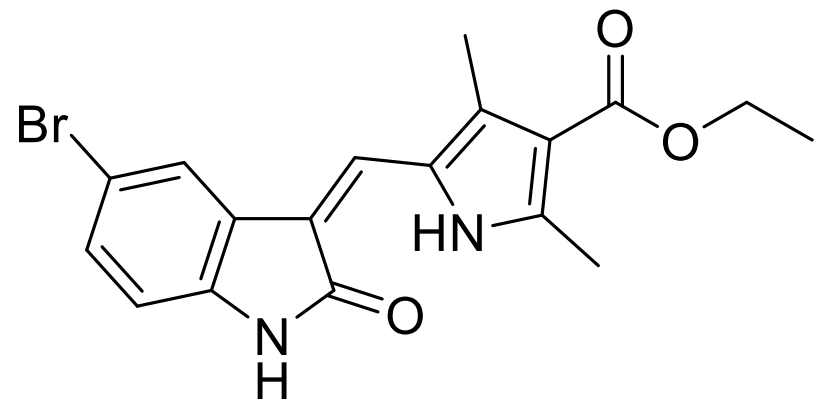

Ethyl (*Z*)-5-((5-bromo-2-oxoindolin-3-ylidene)methyl)-2,4-dimethyl-1*H*-pyrrole-3-carboxylate (8u). Orange-yellow solid; yield 90%; mp: 270 °C. IR (ATR, ν_{max}, cm^{-1}): 3248, 2975, 2902, 2867, 1684, 1576, 1467. 1252, 1055.

^{1}H NMR (500 MHz, DMSO-d_6) δ_H 13.87 (s, 1H, $H_{\text{-pyrrole}}$), 11.08 (s, 1H, H-$1_{\text{-oxindole}}$), 8.14 (d, *J* = 2.1 Hz, 1H, $H_{\text{-oxindole}}$), 7.81 (s, 1H, $H_{\text{-alkene}}$), 7.27 (dd, *J* = 8.2, 2.0 Hz, 1H, $H_{\text{-oxindole}}$), 6.82 (d, *J* = 8.2 Hz, 1H, $H_{\text{-oxindole}}$), 4.21 (q, *J* = 7.1 Hz, 2H, CH_2), 2.53 (s, 3H, $CH_{3\text{-pyrrole}}$), 2.51 (s, 3H, $CH_{3\text{-pyrrole}}$), 1.30 (t, *J* = 7.1 Hz, 3H, CH_3).

^{13}C NMR (125 MHz, DMSO-d_6) δ_C 169.7 (C=O), 164.7 (C=O), 141.4 ($C_{\text{-pyrrole}}$), 138.0 ($C_{\text{-oxindole}}$), 133.8 ($C_{\text{-alkene}}$), 129.3 ($C_{\text{-oxindole}}$), 128.2 ($C_{\text{-oxindole}}$), 126.7 ($C_{\text{-pyrrole}}$), 125.5 ($CH_{\text{-pyrrole}}$),

122.2 (C-oxindole), 115.6 (C-oxindole), 114.1 (C-pyrrole), 114.0 (C-oxindole), 111.7 (C-oxindole), 59.7 (OCH_2), 15.0 (CH_3), 14.7 ($CH_{3\text{-pyrrole}}$), 11.9 ($CH_{3\text{-pyrrole}}$).

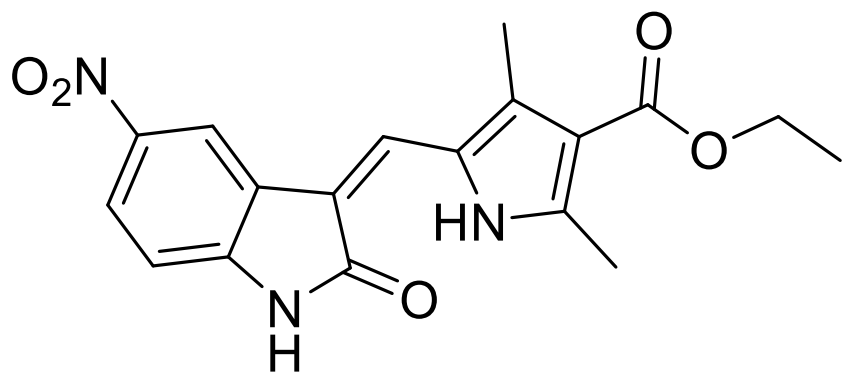

Ethyl (*Z*)-2,4-dimethyl-5-((5-nitro-2-oxoindolin-3-ylidene)methyl)-1*H*-pyrrole-3-carboxylate (8v). Orange-yellow solid; yield 82%; mp: 292 °C.

^{1}H NMR (500 MHz, DMSO-d_6) δ_H 13.73 (s, 1H, H-1-pyrrole), 11.58 (s, 1H, H-1-oxindole), 8.86 (d, *J* = 2.2 Hz, 1H, H-oxindole), 8.06 (dd, *J* = 8.4, 2.3 Hz, 1H, H-oxindole), 8.03 (s, 1H, H-alkene), 7.03 (d, *J* = 8.6 Hz, 1H, H-oxindole), 4.21 (q, *J* = 6.9 Hz, 2H, OCH_2), 2.54 (s, 3H, $CH_{3\text{-pyrrole}}$), 2.53 (s, 3H, $CH_{3\text{-pyrrole}}$), 1.30 (t, *J* = 7.1 Hz, 3H, CH_3).

^{13}C NMR (125 MHz, DMSO-d_6) δ_C 170.4 (C=O), 164.6 (C=O), 144.1 (C-pyrrole), 142.8 (C-oxindole), 142.2 (C-oxindole), 135.3, 127.1, 126.9, 126.6 (C-pyrrole), 123.2 (C-oxindole), 115.4 (C-oxindole), 114.5, 114.4, 109.8 (C-oxindole), 59.7 (OCH_2), 15.1 ($CH_{3\text{-pyrrole}}$), 14.7 (CH_3), 12.0 ($CH_{3\text{-pyrrole}}$).

HRMS (ESI-TOF) *m/z* $[M-H]^-$ calcd. for $C_{18}H_{17}N_3O_5$: 354.1095, found: 354.1099.

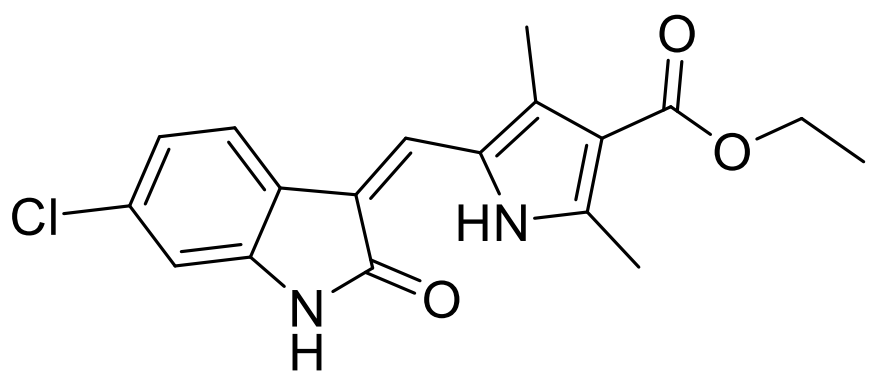

Ethyl (*Z*)-5-((6-chloro-2-oxoindolin-3-ylidene)methyl)-2,4-dimethyl-1*H*-pyrrole-3-carboxylate (**8w**). Yellow solid; yield 87%; mp: 248 °C.
^{1}H NMR (500 MHz, DMSO-d_6) δ_H 13.76 (s, 1H, H-pyrrole), 11.09 (s, 1H, H-1-oxindole), 7.84 (d, *J* = 8.2 Hz, 1H, H-oxindole), 7.71 (s, 1H, H-alkene), 7.04 (dd, *J* = 8.0, 2.0 Hz, 1H, H-oxindole), 6.88 (d, *J* = 2.0 Hz, 1H, H-oxindole), 4.21 (q, *J* = 7.0 Hz, 2H, OCH_2), 2.53 (s, 3H, $CH_{3\text{-pyrrole}}$), 2.49 (s, 3H, $CH_{3\text{-pyrrole}}$), 1.29 (t, *J* = 7.2 Hz, 3H, CH_3).

^{13}C NMR (125 MHz, DMSO-d_6) δ_C 170.0 (C=O), 164.7 (C=O), 141.1 (C-pyrrole), 140.1 (C-oxindole), 133.2 (C-alkene), 131.2 (C-oxindole), 126.6, 124.7, 124.6, 121.4 (C-oxindole), 120.8 (C-oxindole), 115.7 (C-oxindole), 114.0 (C-pyrrole), 109.9 (C-oxindole), 59.6 (CH_2), 15.0 ($CH_{3\text{-pyrrole}}$), 14.7 (CH_3), 11.9 ($CH_{3\text{-pyrrole}}$).

HRMS (ESI-TOF) *m/z* $[M+H]^+$ calcd. for $C_{18}H_{17}ClN_2O_3$: 345.1000, found: 345.1002.

3. Single-Crystal X-ray Structure Determination

Single-crystal X-ray diffraction analysis was carried out to confirm the molecular structure of compound **8r**. A suitable yellow block crystal was measured on a Bruker D8 Venture diffractometer using Mo*Kα* radiation (λ = 0.71073 Å) at 100(2) K. The crystal was determined to be monoclinic, space group Cc, and the structure was refined to final values of R_1 = 0.0307 and wR_2 = 0.0835 for reflections with $I \geq 2\sigma(I)$. The ORTEP representation is shown in Figure S2, and detailed crystallographic data are provided in Table S1. The crystallographic data have been deposited with the Cambridge Crystallographic Data Centre (CCDC 2499402).

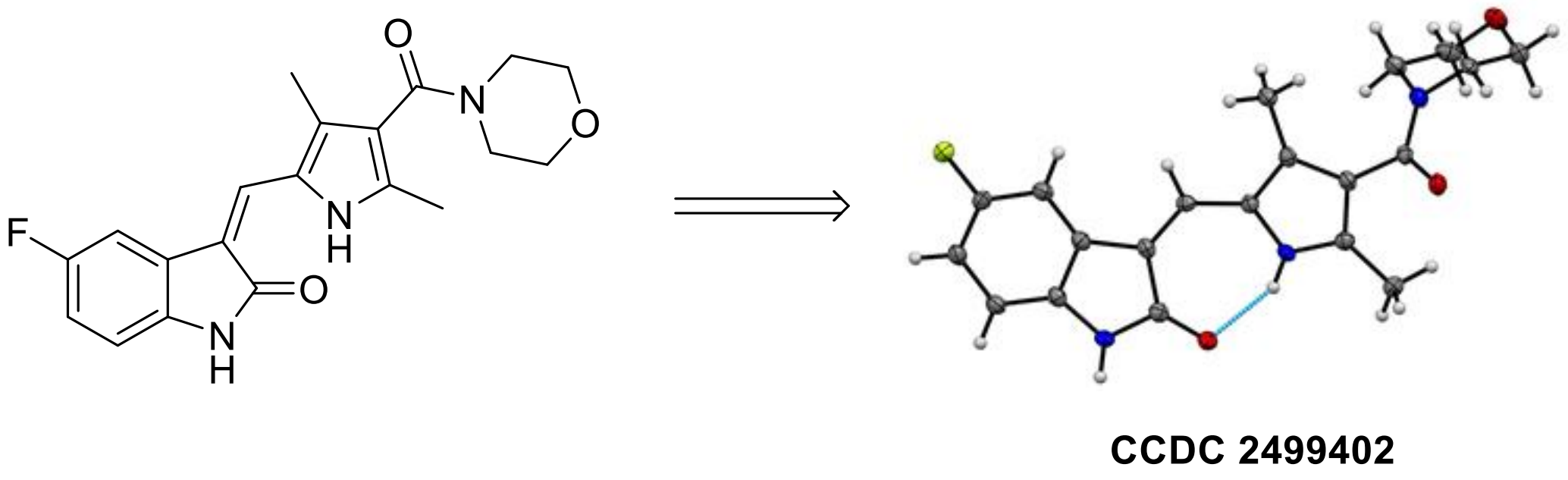

Figure S2. ORTEP structure of compound **8r.**

Table S1. Crystal data and structure refinement for compound **8r.**

**Parameter**	**Value**
CCDC number	2499402
Empirical formula	$C_{20}H_{20}FN_3O_3$
Formula weight	369.39
Temperature (K)	100(2)
Crystal system	Monoclinic
Space group	Cc (9)
a (Å)	12.960(2)
b (Å)	12.426(2)
c (Å)	11.5398(17)
β (°)	114.032(8)
Volume (Å^3)	1697.2(5)
Z	4
Density (g cm^{-3})	1.446
μ (mm^{-1})	0.106
F(000)	776
Radiation	Mo$K\alpha$ (λ = 0.71073 Å)
Reflections collected	30291
Independent reflections	5828
R_{int}	0.1563
Completeness (%)	99.9
Goodness-of-fit on F^2	0.617
R_1 [$I \geq 2\sigma(I)$]	0.0307
wR_2 [$I \geq 2\sigma(I)$]	0.0835
R_1 (all data)	0.0358
wR_2 (all data)	0.0864
Largest diff. peak/hole (eÅ^{-3})	0.19 / −0.19
Flack parameter	−0.2(4)

4. Green Metric Calculations

Atom economy (AE), molar efficiency (ME), process mass intensity (PMI), and E-factor were evaluated based on literature procedures[1] while energy intensity (EI) and time intensity (TI) have been calculated according to literature[2] using the following equations:

$$AE = \frac{number\ of\ atoms\ in\ the\ final\ products}{number\ of\ atoms\ in\ the\ reagents} x\ 100$$

$$ME = \frac{moles\ of\ products}{moles\ of\ reagents + mole\ of\ catalyst} x\ 100$$

$$PMI = \frac{total\ mass\ of\ process\ steps}{mass\ of\ product}$$

$$E - factor = \frac{mass\ of\ total\ waste}{mass\ of\ product}$$

$$Energy\ intensity = \frac{Total\ Energy}{Mass\ of\ final\ product}$$

$$Time\ intensity = \frac{Total\ Time}{Mass\ of\ final\ product}$$

The total energy consumed during electrolysis (E) was calculated using E=V.I.t, where U is the cell voltage, I is the current, and t is the electrolysis time. For the conventional reaction, the total energy consumption (E) was calculated as E=P×t, assuming an effective heating power of 250 W.

EcoScale assessment, including yield, reagents, price and availability, safety, technical setup, temperature/time, workup, and purification, was calculated according to the literature[3] and is available at https://ecoscale.cheminfo.org/. An example of the EcoScale calculation is shown in Figure S3. Green Star assessment was conducted using the software developed by Mansour et al. [4], which evaluates the twelve principles of green chemistry.

(a)

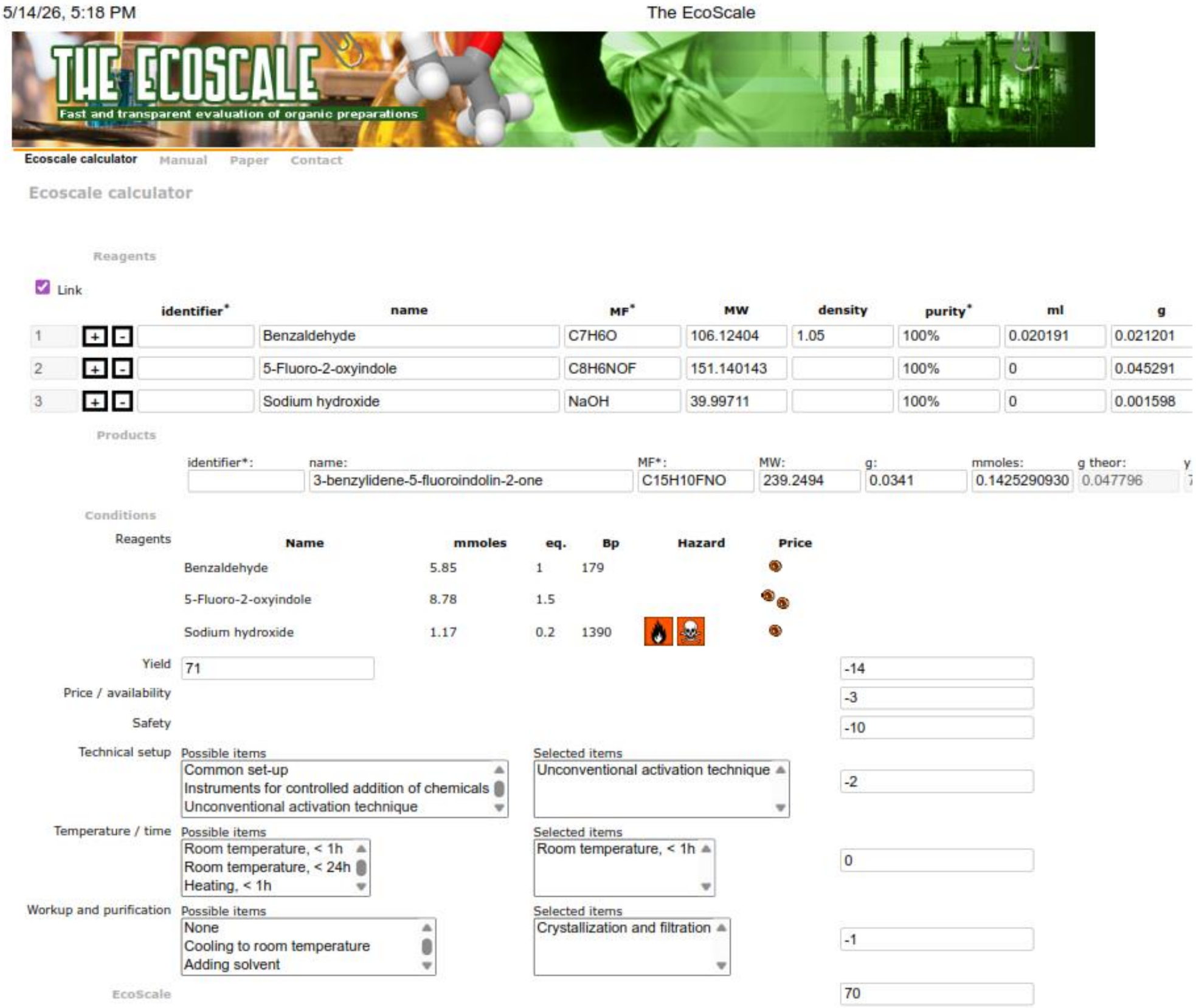

(b)

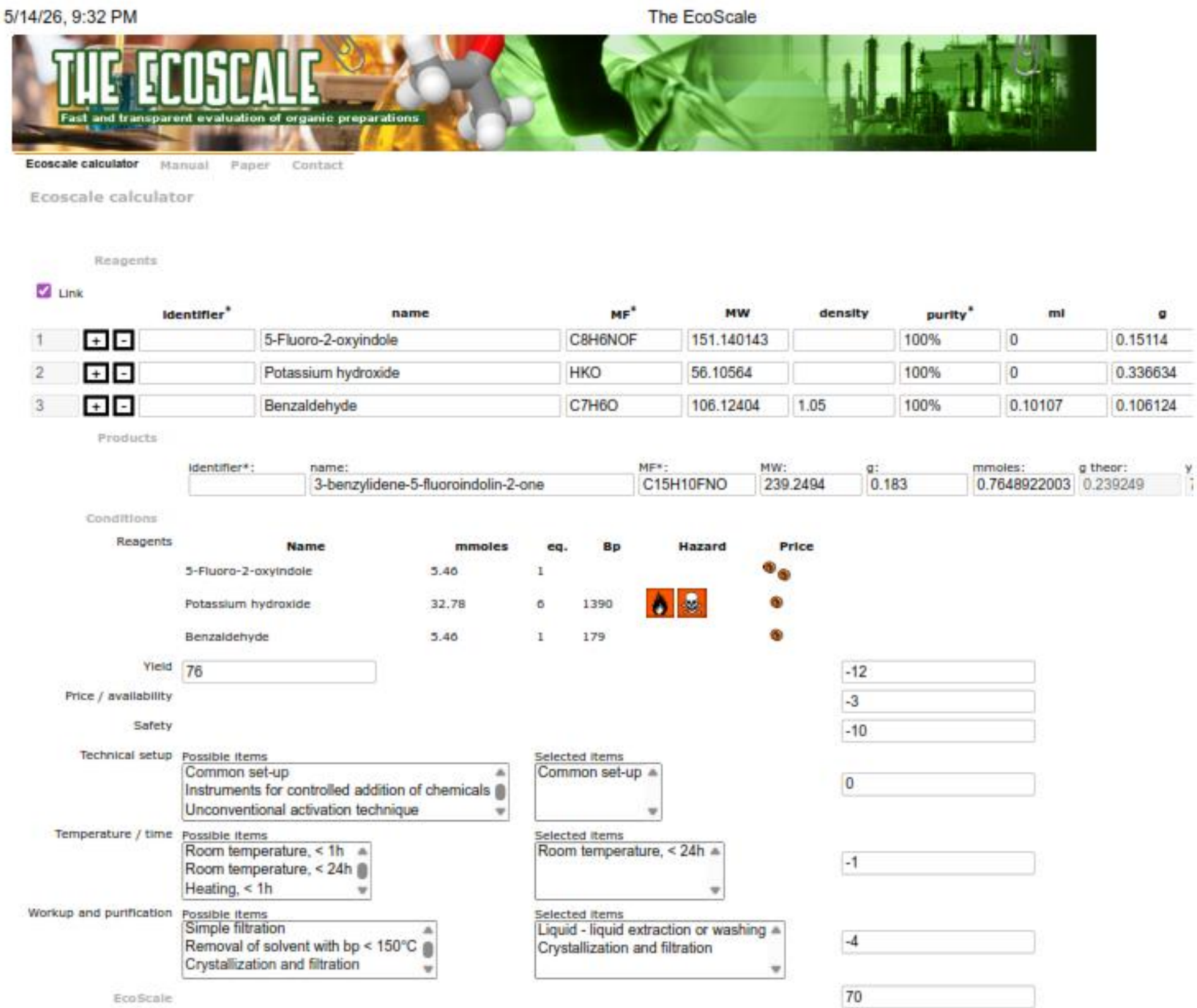

Figure S3. (a) EcoScale calculation for the electro-condensation method; (b) EcoScale calculation for the conventional method.

5. References

[1] L. Tedesco, F. Verdini, E.C. Gaudino, S. Tabasso, I. Arduino, D. Lembo, M. Donalisio, G. Cravotto, M. Manzoli, Sustainable route to antiviral furano-chalcones via microwave-assisted solvent-free synthesis with recyclable MgO, Green Chem. 28 (2026) 5334–5346. https://doi.org/10.1039/D5GC06471F.

[2] A.L. Kerek, L. Sens, M.C.M. Sandri, B.C. Fiorin, Green synthesis of Azachalcones: an evaluation based on chemical greenness metrics, Green Chemistry Letters and Reviews 18 (2025) 2581930. https://doi.org/10.1080/17518253.2025.2581930.

[3] K.V. Aken, L. Strekowski, L. Patiny, EcoScale, a semi-quantitative tool to select an organic preparation based on economical and ecological parameters, Beilstein J. Org. Chem. 2 (2006) 3. https://doi.org/10.1186/1860-5397-2-3.

[4] F.R. Mansour, M.A.E. Hassab, T.A. Majrashi, W.M. Eldehna, A Modified Green Star Area (MoGSA) and software to assess greenness of reactions in the chemistry laboratories, PLOS ONE 19 (2024) e0314421. https://doi.org/10.1371/journal.pone.0314421.

6. Spectra

Spectrum ^{1}H NMR (500 MHz, DMSO-*d6*) of compound **5a** (*E*/*Z* = 100:0)

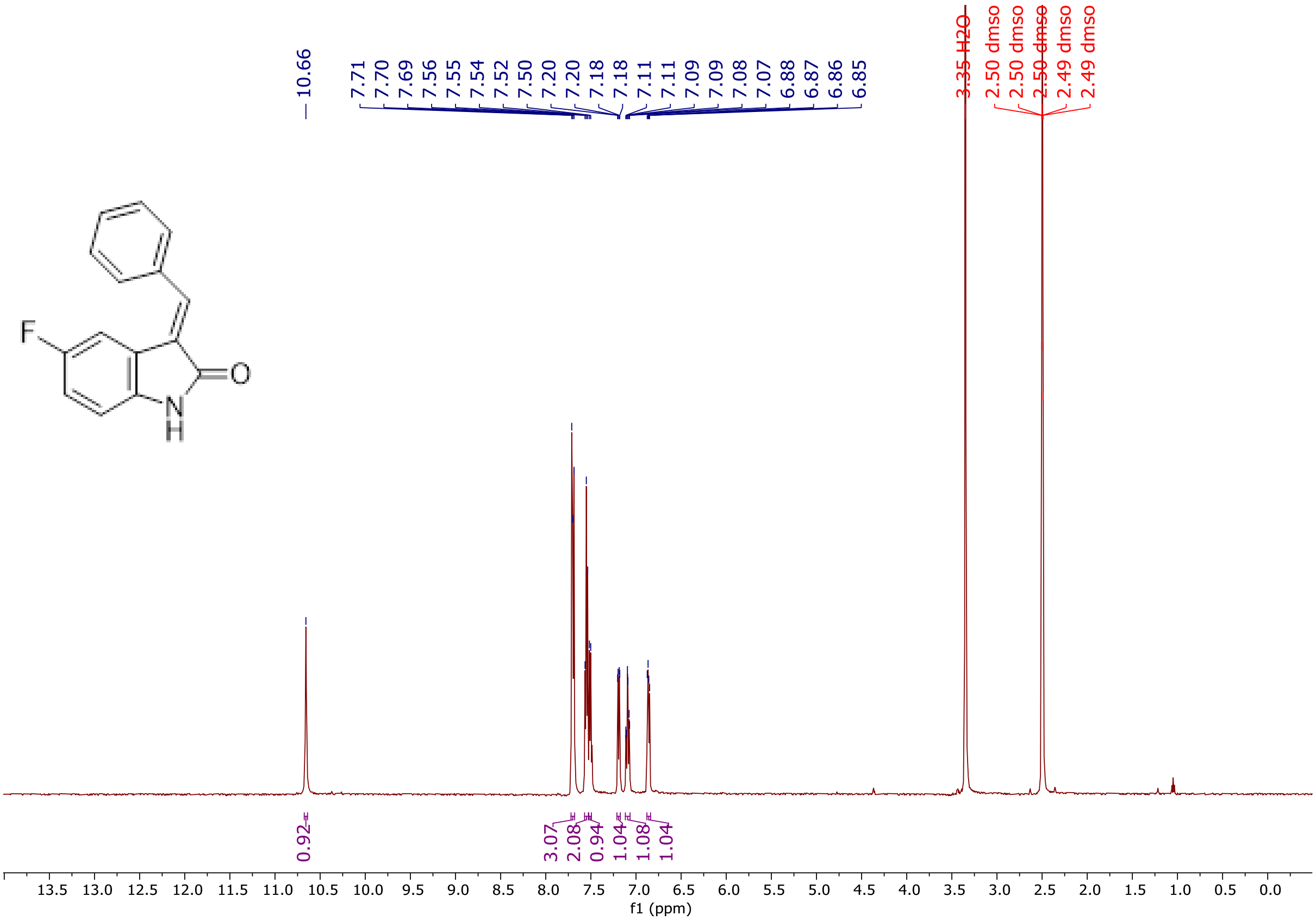

Spectrum ^{13}C NMR (125 MHz, DMSO-*d6*) of compound **5a**

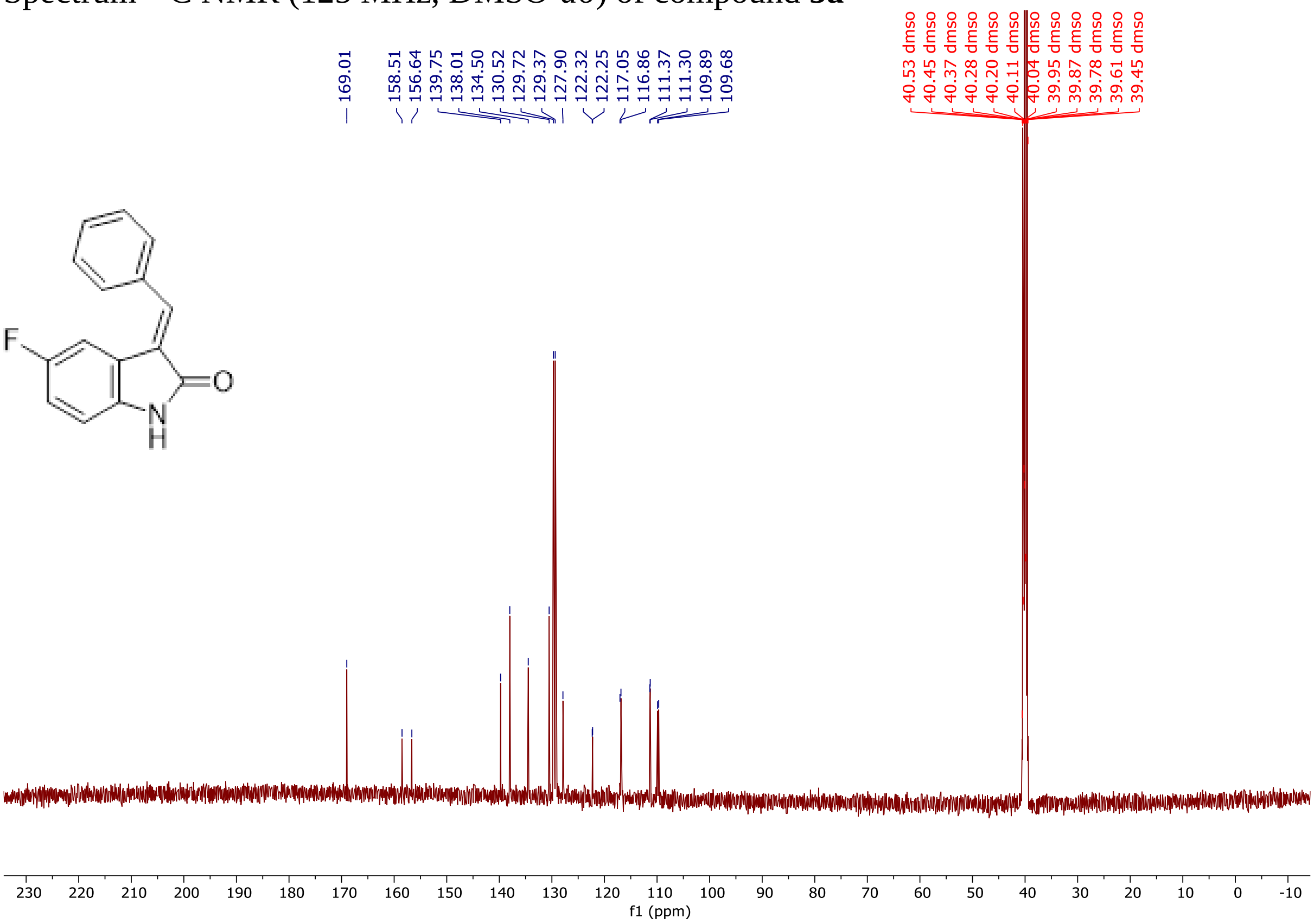

Spectrum ^{19}F NMR (470 MHz, DMSO-d_6) of compound **5a**

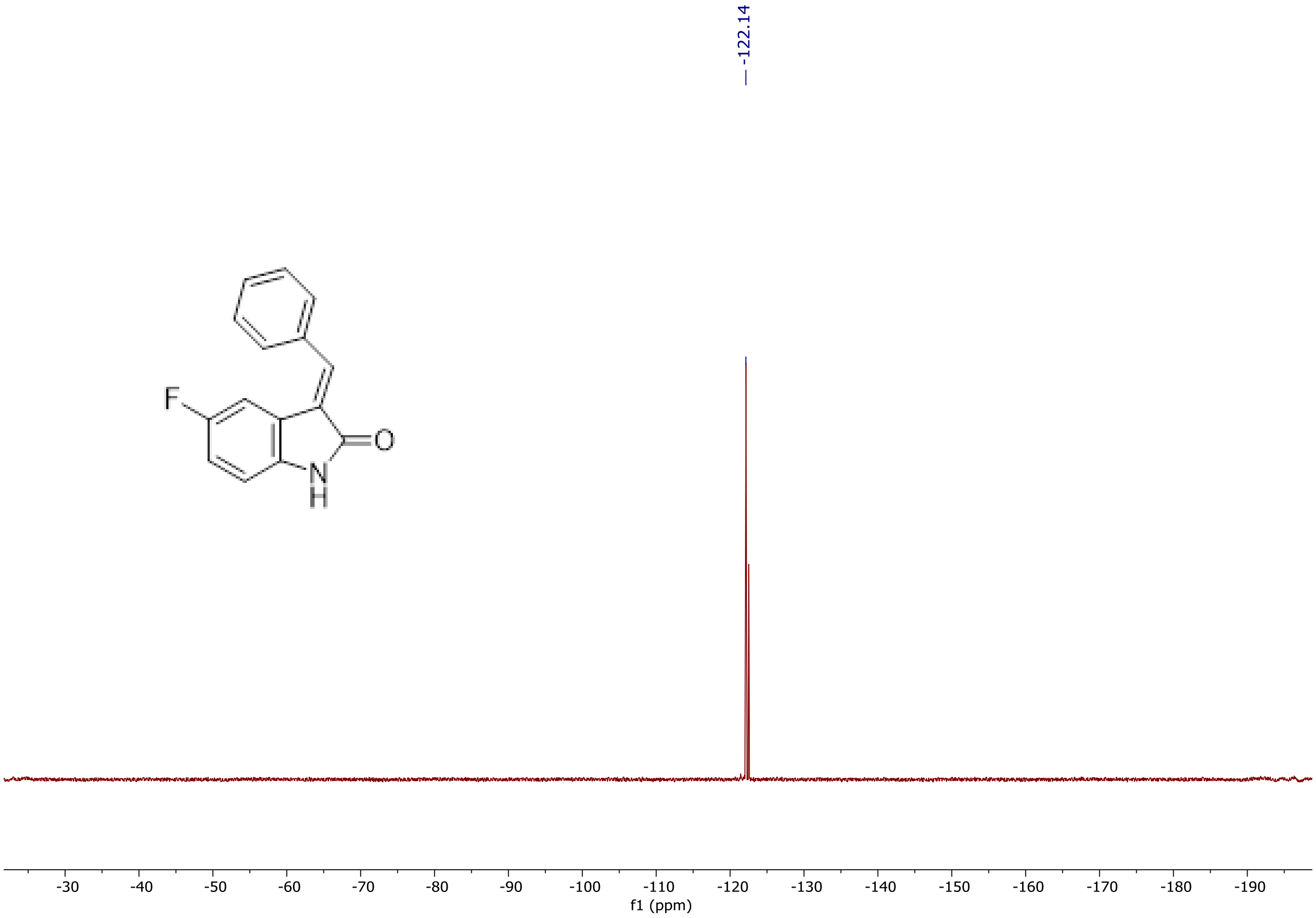

HRMS spectrum of compound **5a**

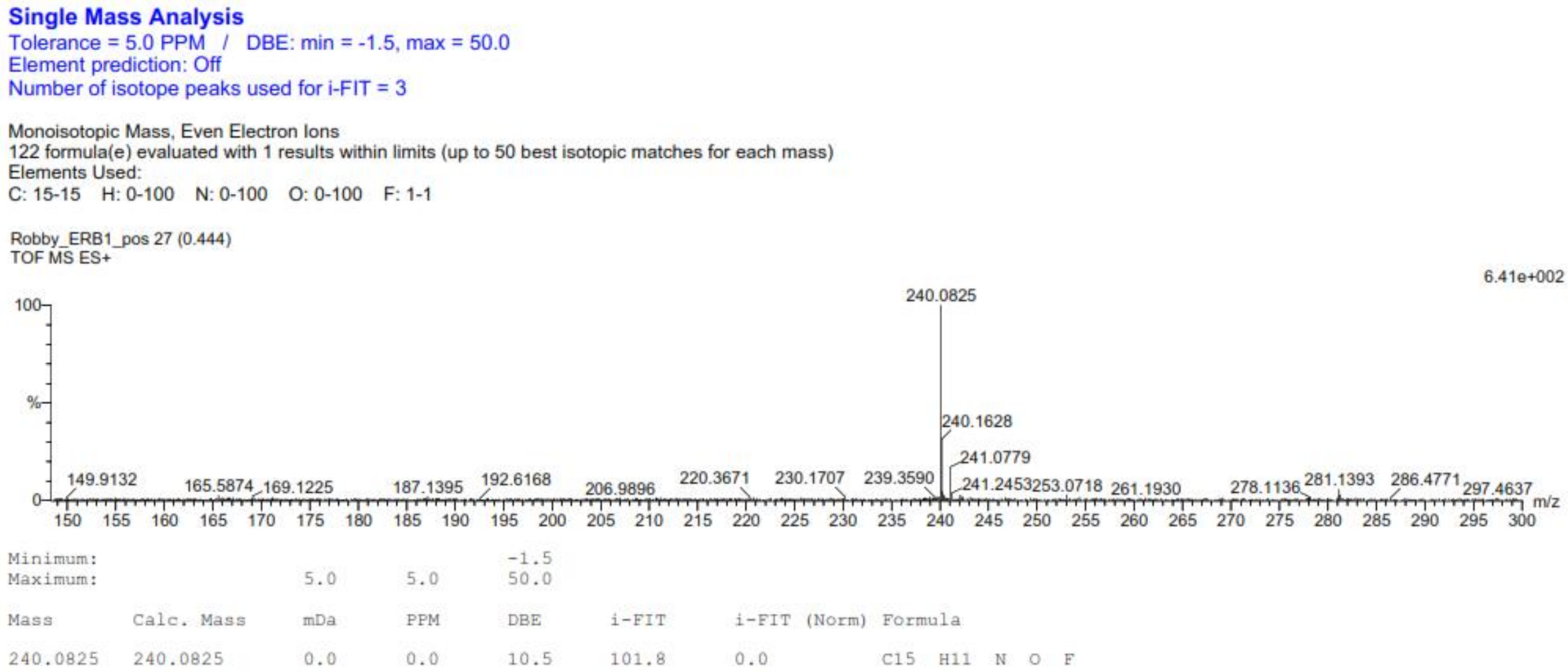

Spectrum ^{1}H NMR (500 MHz, DMSO-*d6*) of compound **5b** (*E/Z* = 93:7)

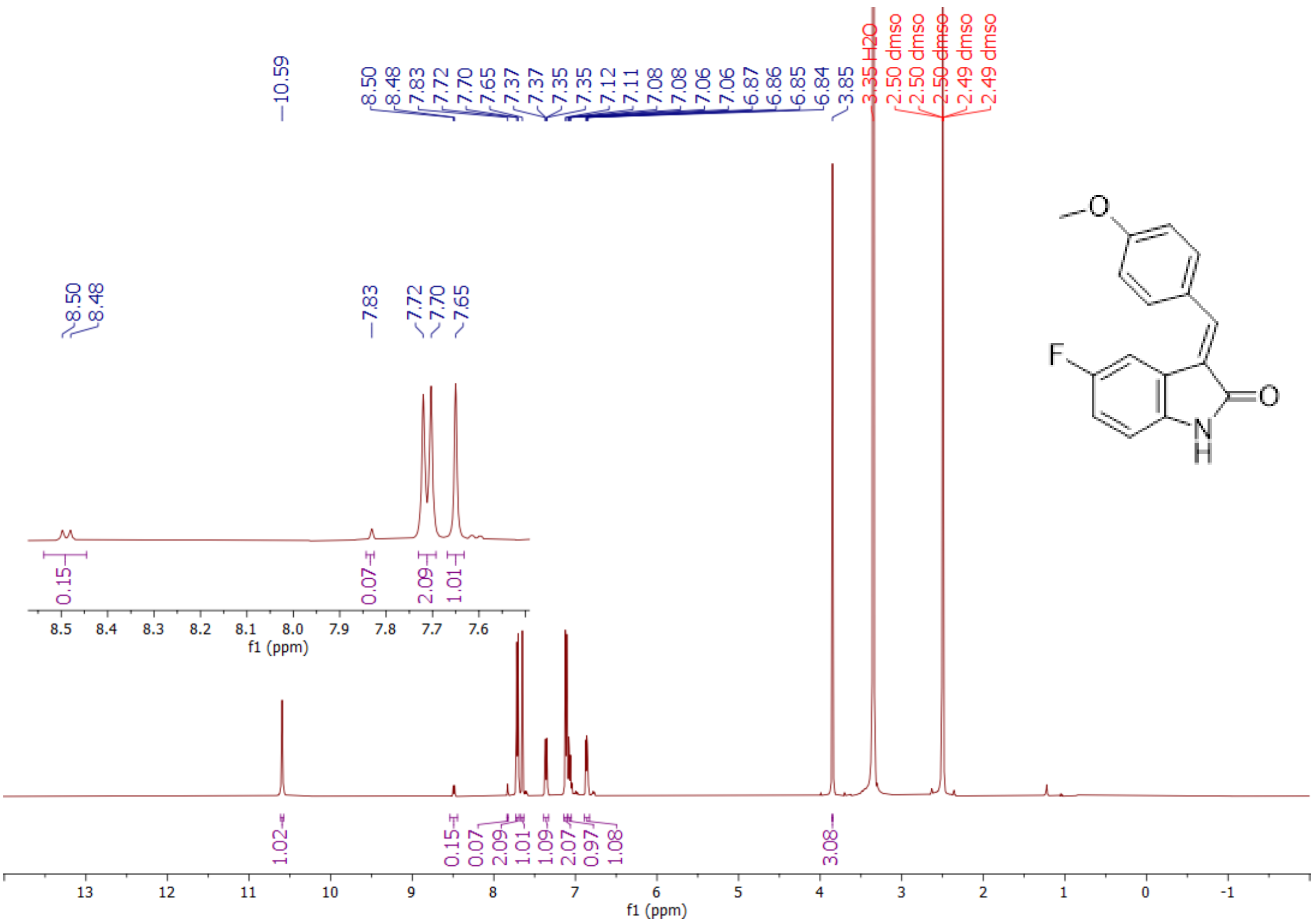

Spectrum ^{13}C NMR (125 MHz, DMSO-*d6*) of compound **5b**

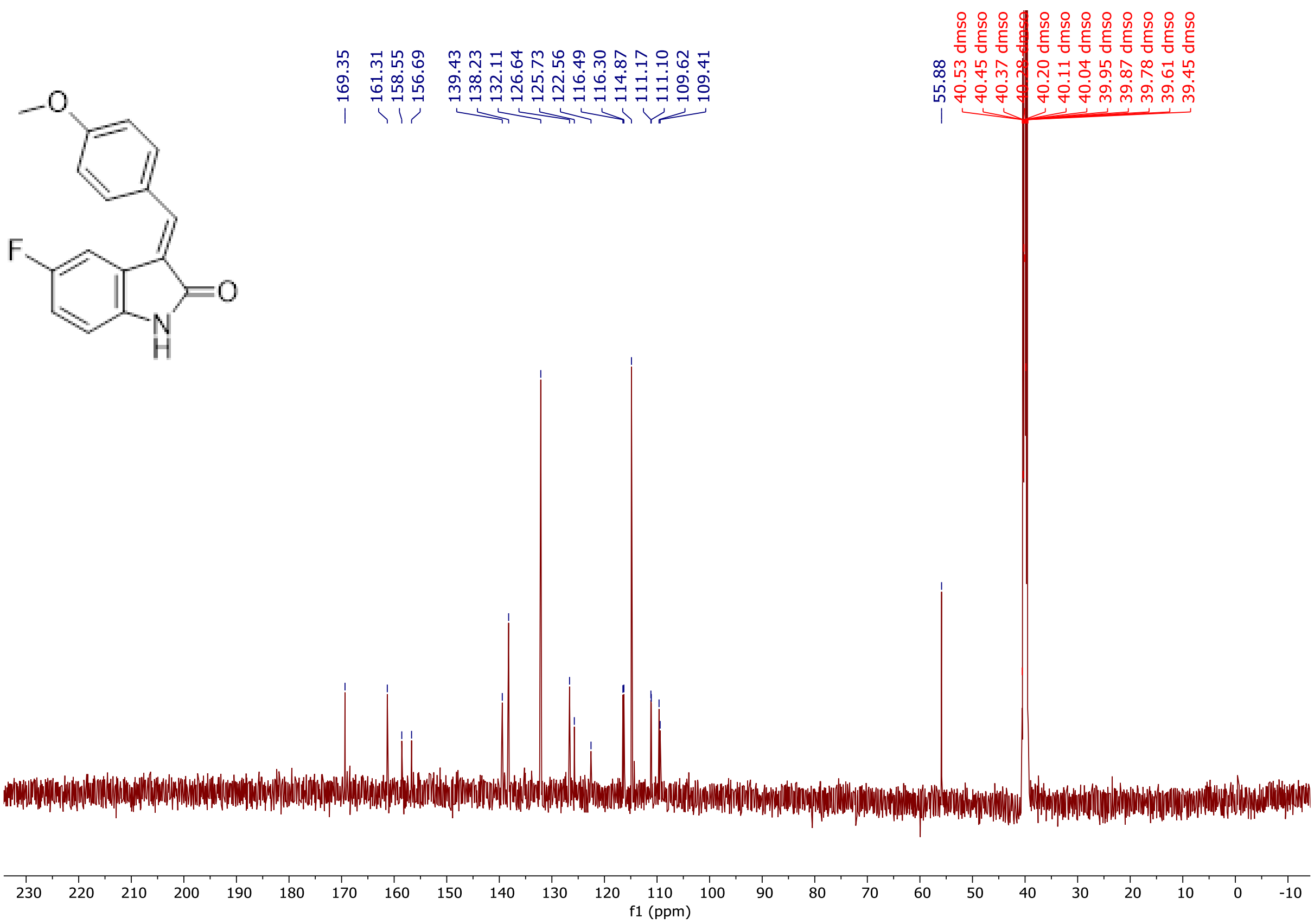

Spectrum ^{19}F NMR (470 MHz, DMSO-d_6) of compound **5b**

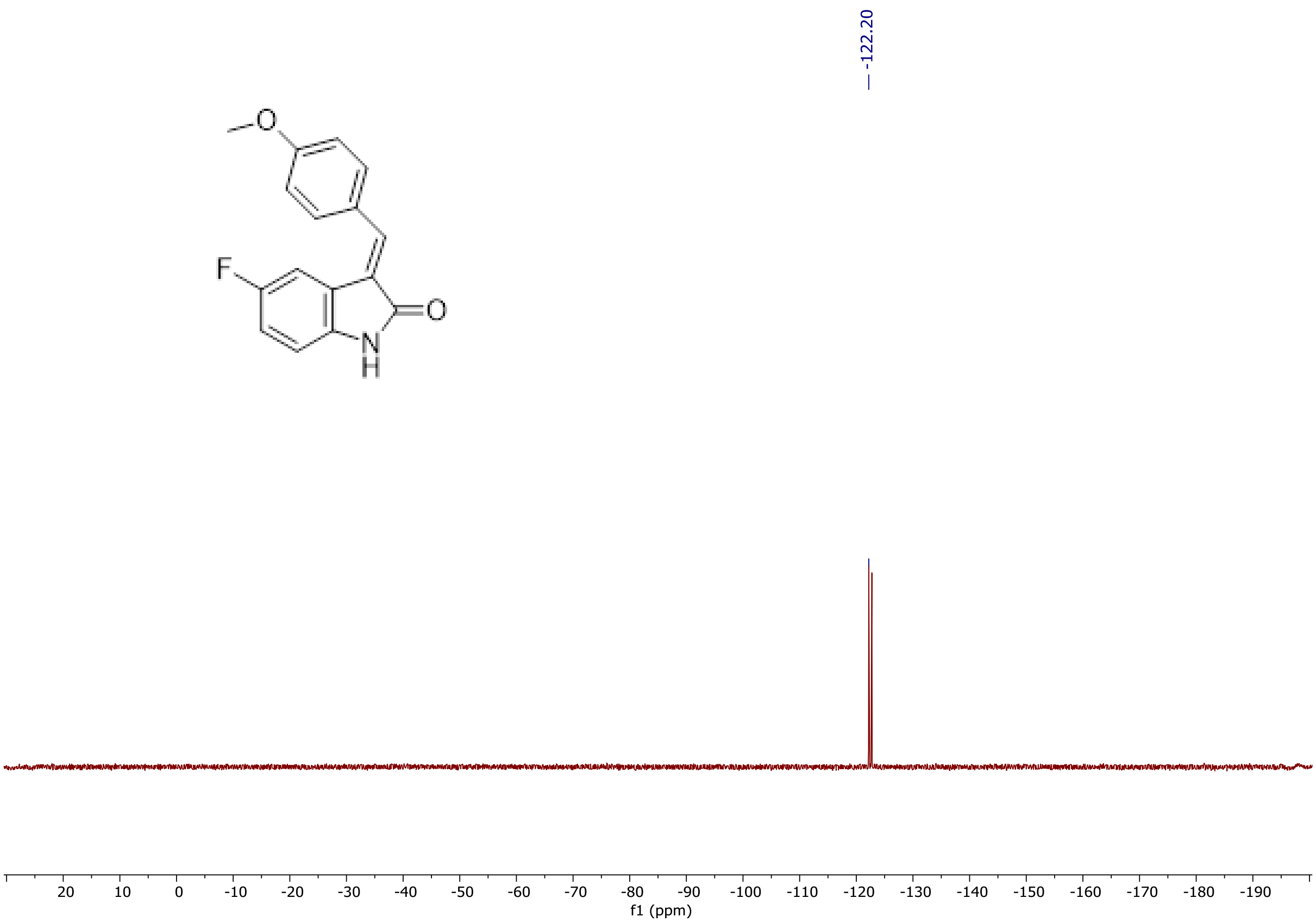

HRMS spectrum of compound **5b**

Single Mass Analysis
Tolerance = 5.0 PPM / DBE: min = -1.5, max = 50.0
Element prediction: Off
Number of isotope peaks used for i-FIT = 3

Monoisotopic Mass, Even Electron Ions
155 formula(e) evaluated with 1 results within limits (up to 50 best isotopic matches for each mass)
Elements Used:
C: 16-16 H: 0-100 N: 0-100 O: 0-100 F: 1-1

Robby_ERB2_pos 28 (0.461)
TOF MS ES+

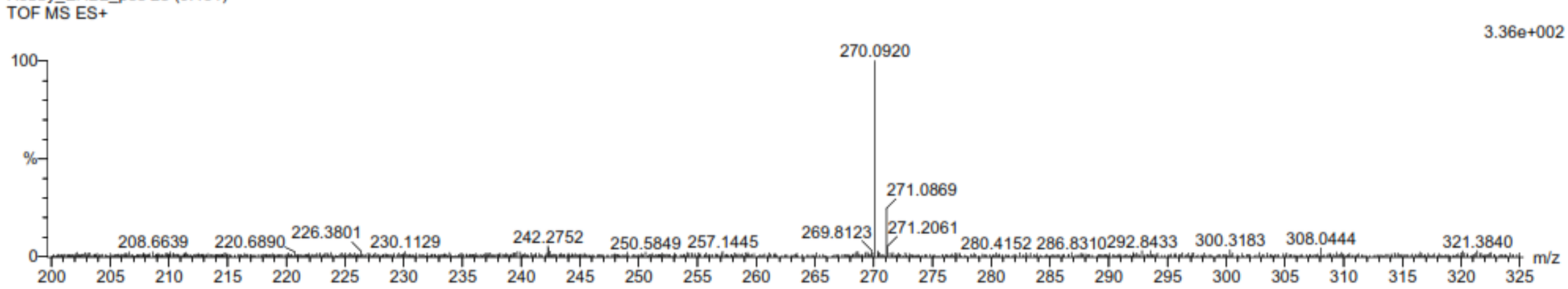

Minimum:				-1.5			
Maximum:		5.0	5.0	50.0			
Mass	Calc. Mass	mDa	PPM	DBE	i-FIT	i-FIT (Norm)	Formula
270.0920	270.0930	-1.0	-3.7	10.5	61.8	0.0	C16 H13 N O2 F

Spectrum ^{1}H NMR (500 MHz, DMSO-*d6*) of compound **5c** (*E/Z* = 100:0)

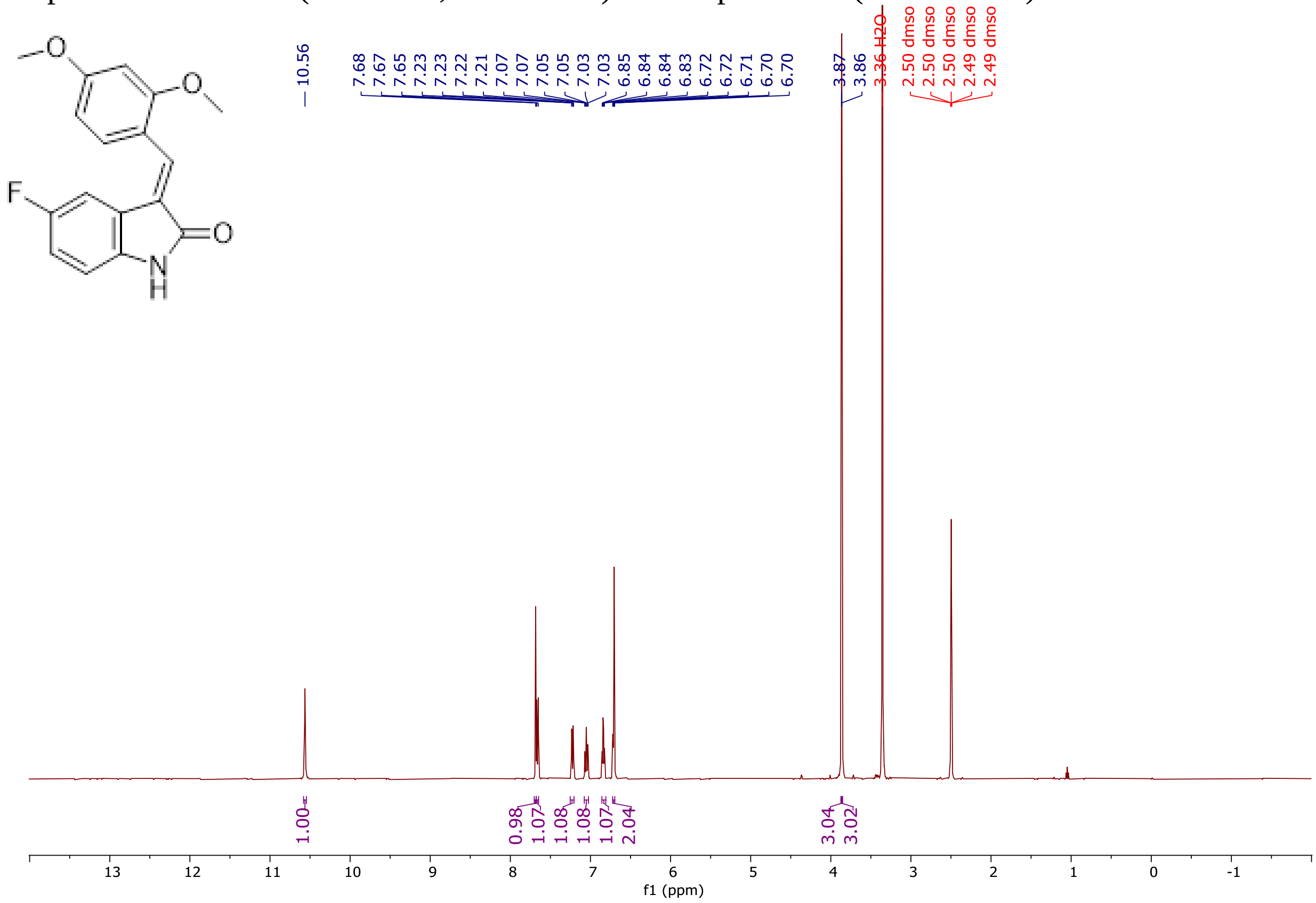

Spectrum ^{13}C NMR (125 MHz, DMSO-*d6*) of compound **5c**

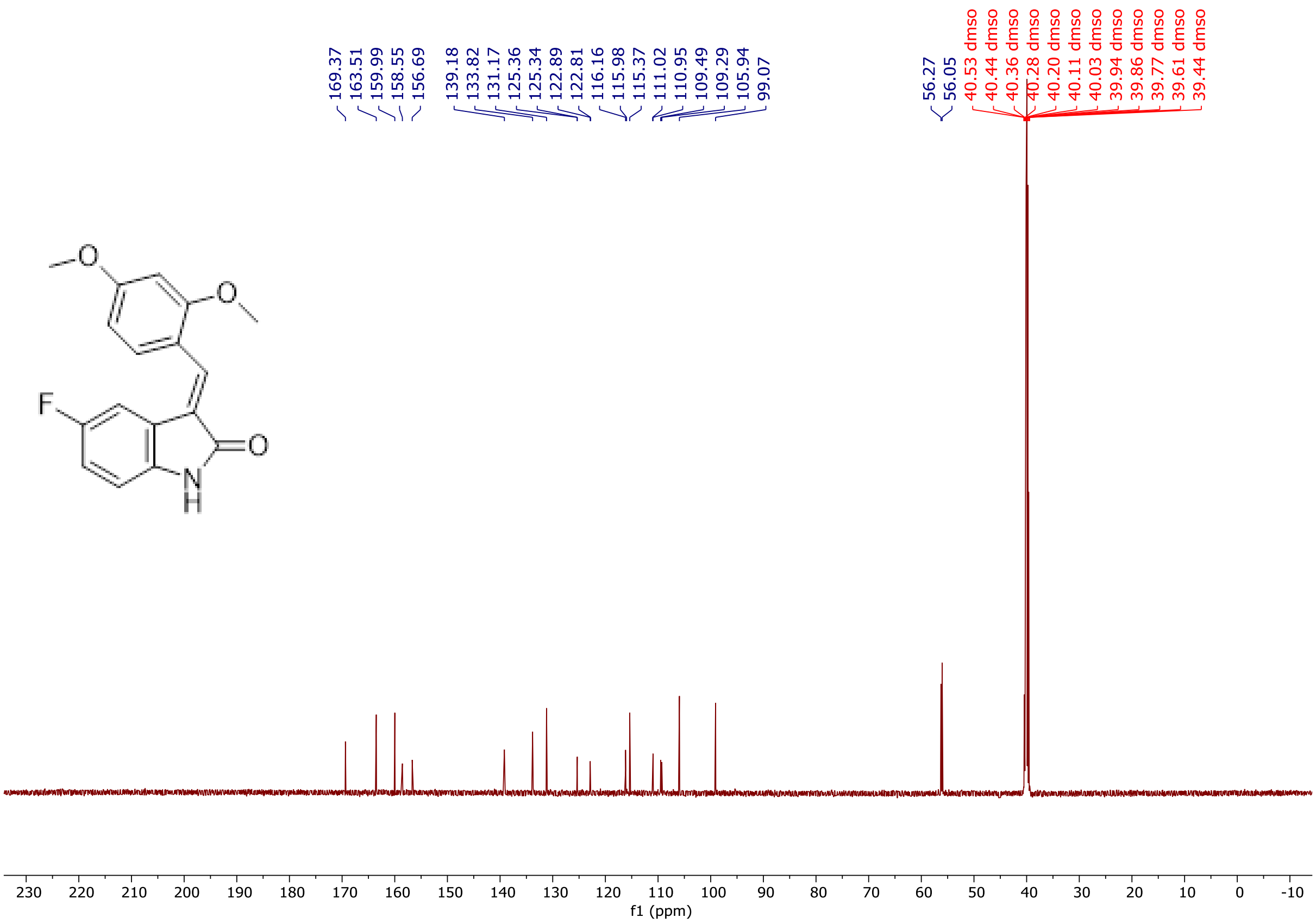

Spectrum ^{19}F NMR (470 MHz, DMSO-d_6) of compound **5c**

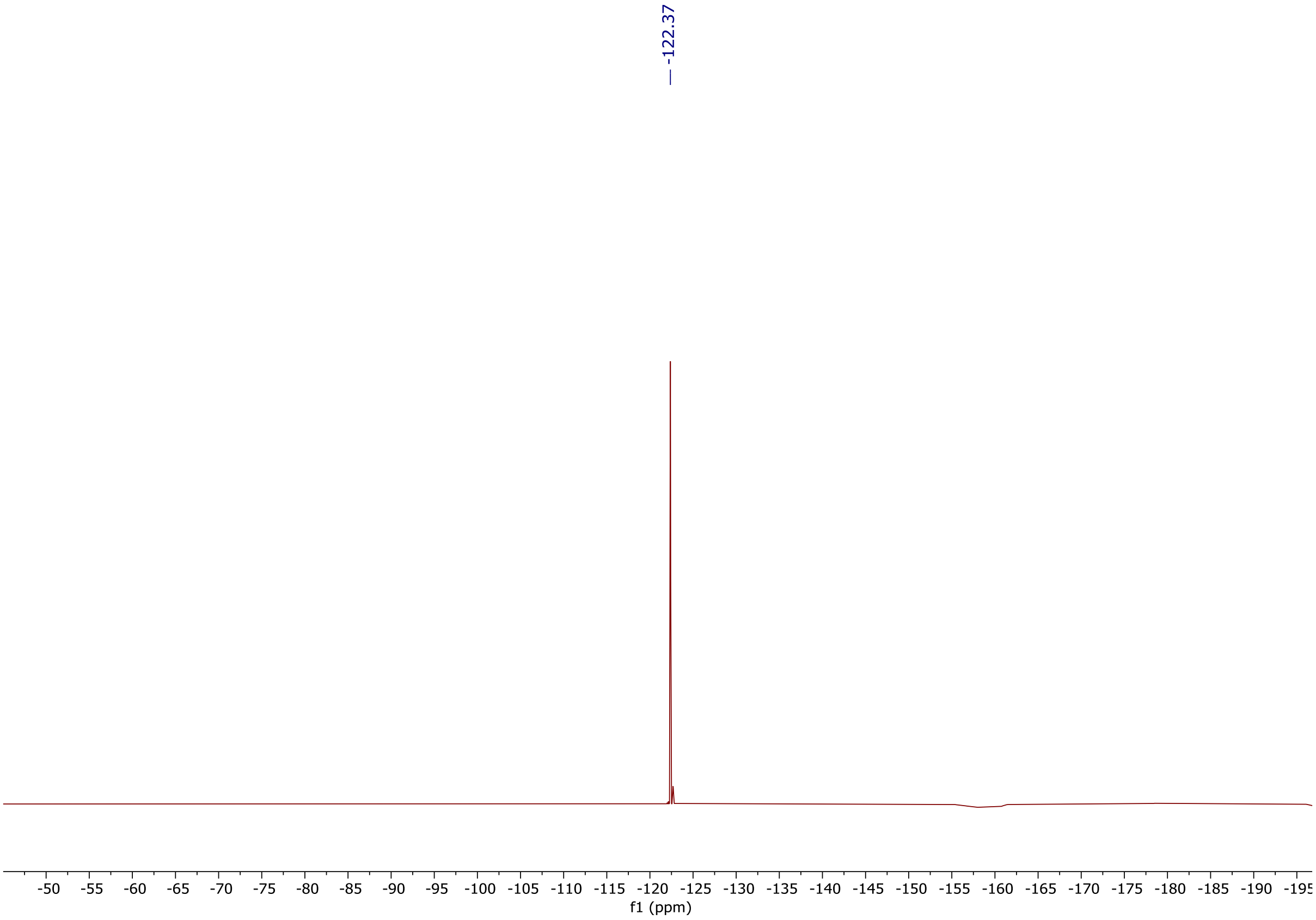

HRMS spectrum of compound **5c**

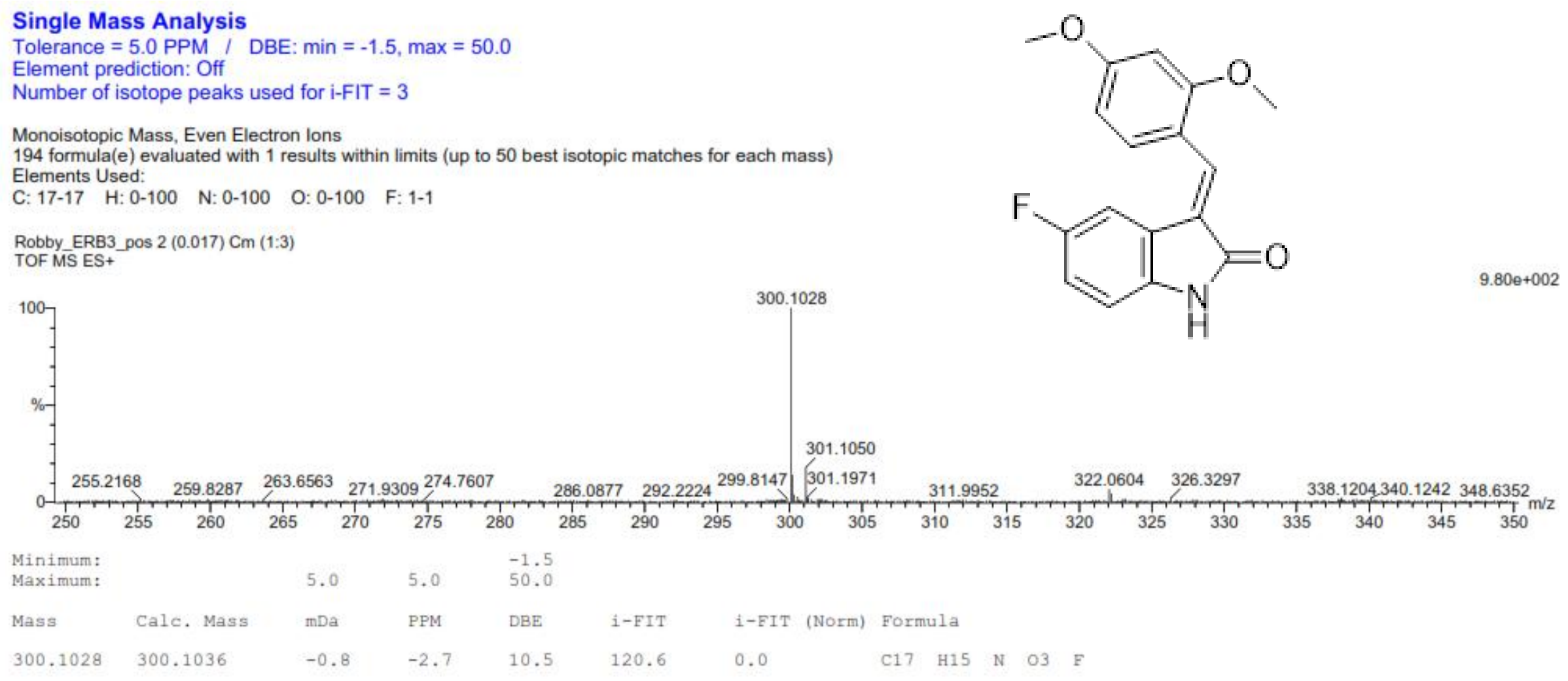

Spectrum ^{1}H NMR (500 MHz, DMSO-*d6*) of compound **5d** (*E/Z* = 100:0)

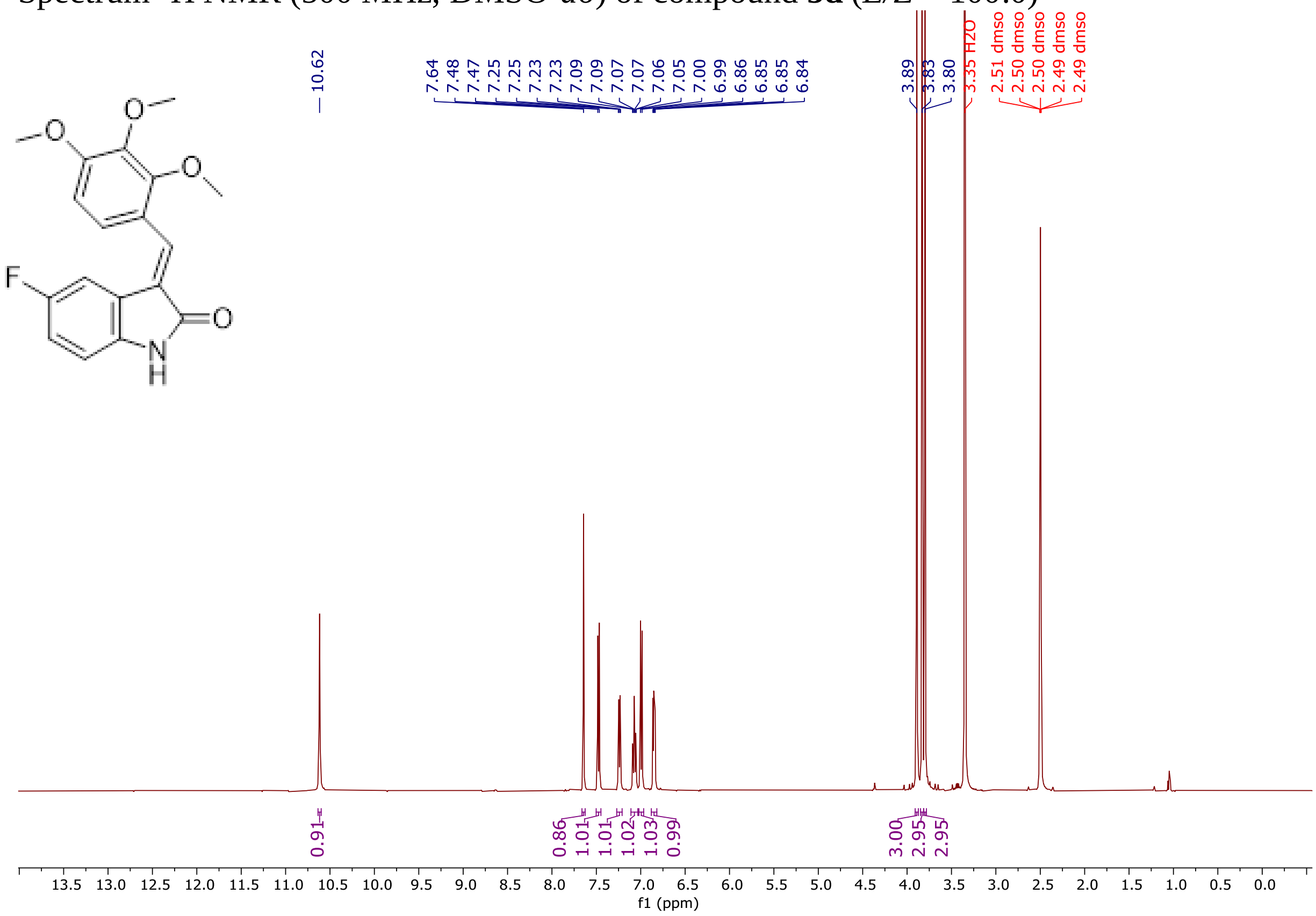

Spectrum ^{13}C NMR (125 MHz, DMSO-*d6*) of compound **5d**

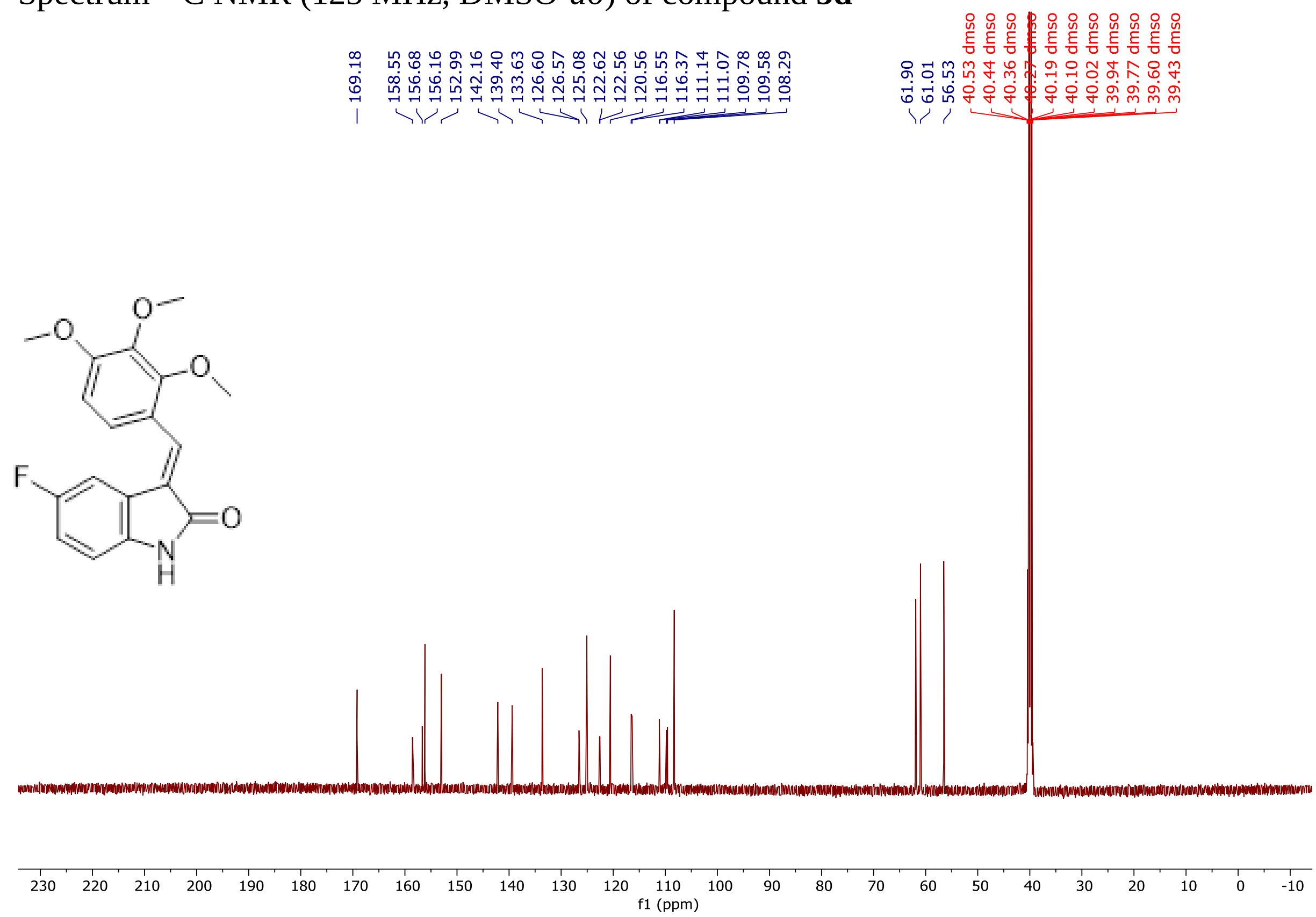

Spectrum ^{19}F NMR (470 MHz, DMSO-d_6) of compound **5d**

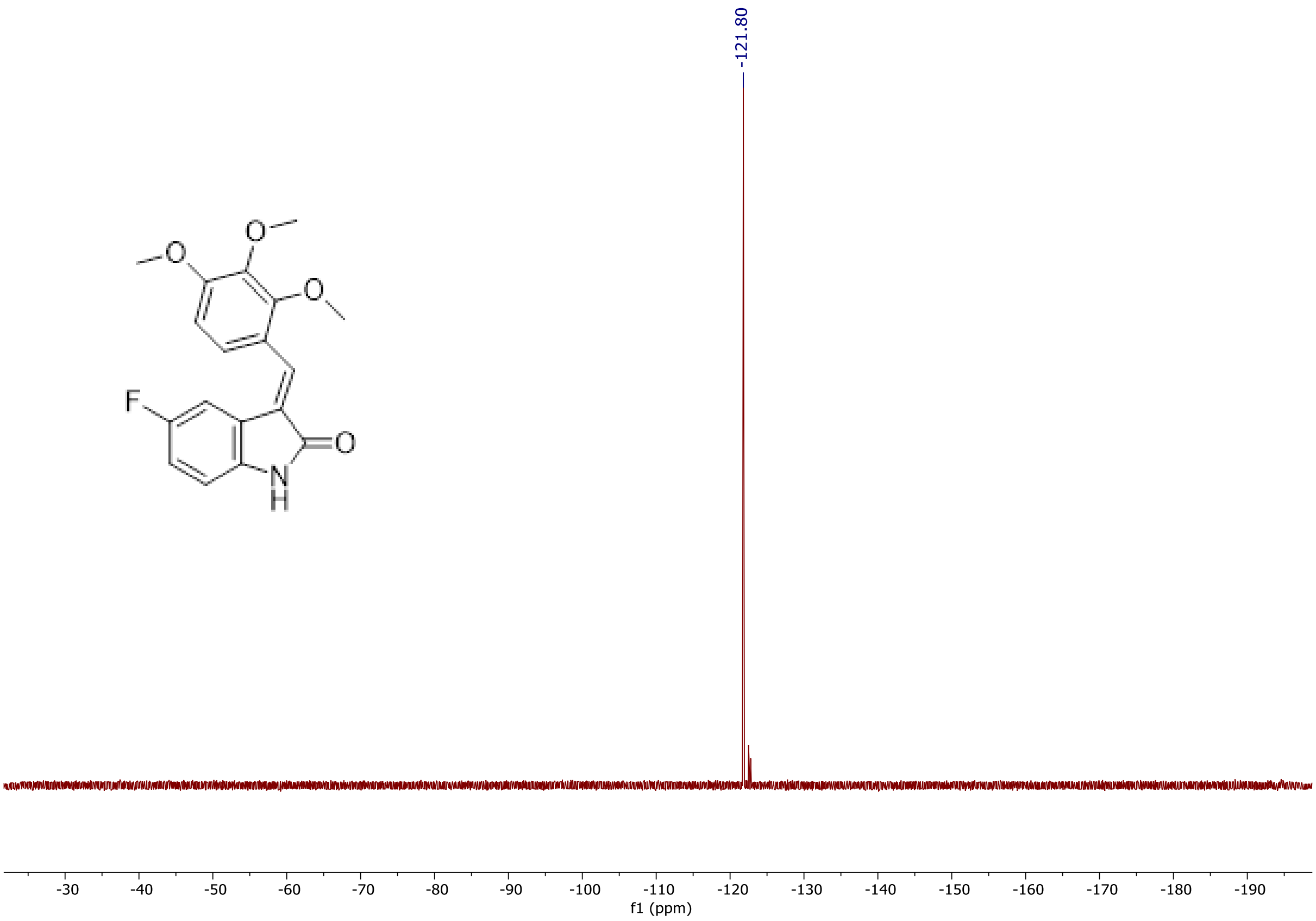

HRMS spectrum of compound **5d**

Single Mass Analysis
Tolerance = 5.0 PPM / DBE: min = -1.5, max = 50.0
Element prediction: Off
Number of isotope peaks used for i-FIT = 3

Monoisotopic Mass, Even Electron Ions
235 formula(e) evaluated with 1 results within limits (up to 50 best isotopic matches for each mass)
Elements Used:
C: 18-18 H: 0-100 N: 0-100 O: 0-100 F: 1-1

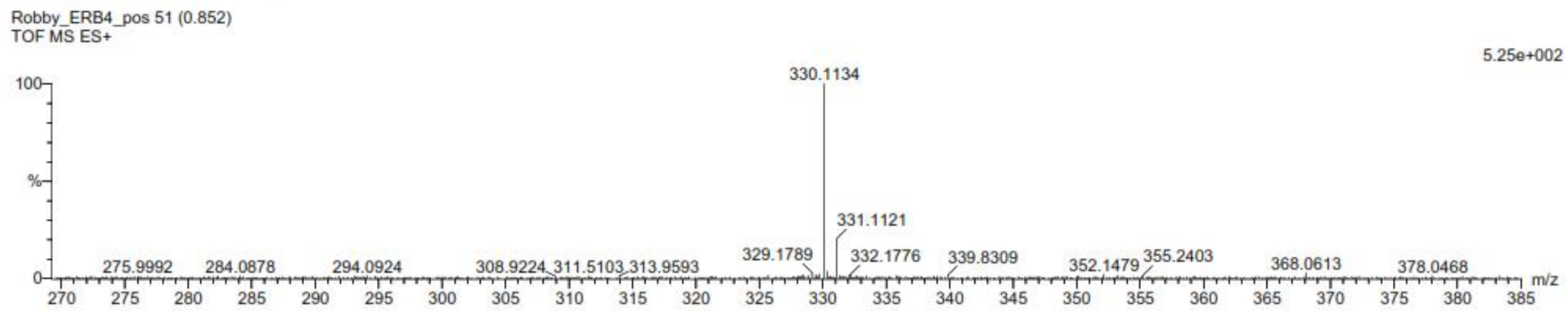

Minimum:				-1.5			
Maximum:		5.0	5.0	50.0			
Mass	Calc. Mass	mDa	PPM	DBE	i-FIT	i-FIT (Norm)	Formula
330.1134	330.1142	-0.8	-2.4	10.5	65.3	0.0	C18 H17 N O4 F

Spectrum ^{1}H NMR (500 MHz, DMSO-*d6*) of compound **5e** (*E*/*Z* = 98:2)

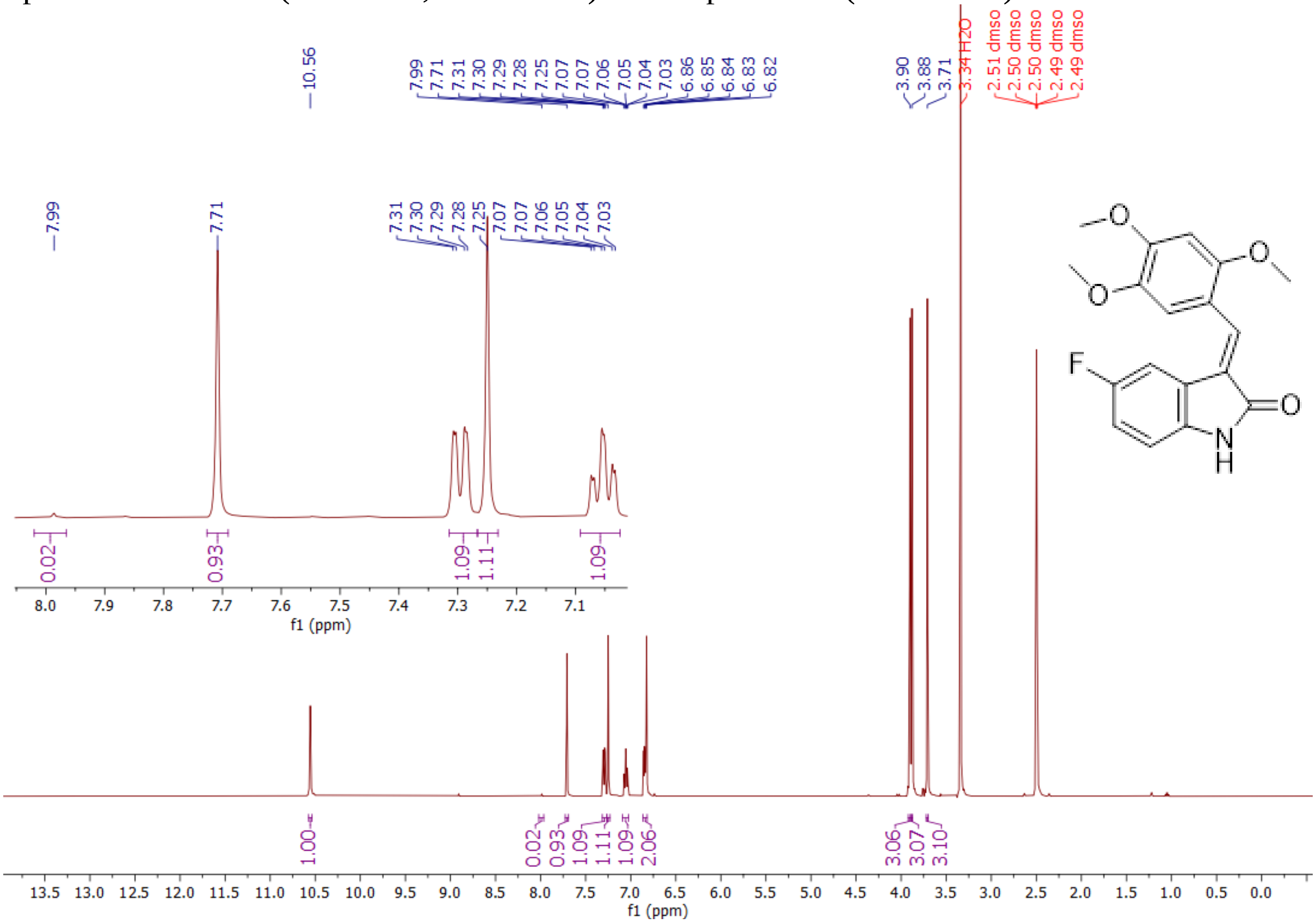

Spectrum ^{13}C NMR (125 MHz, DMSO-*d6*) of compound **5e**

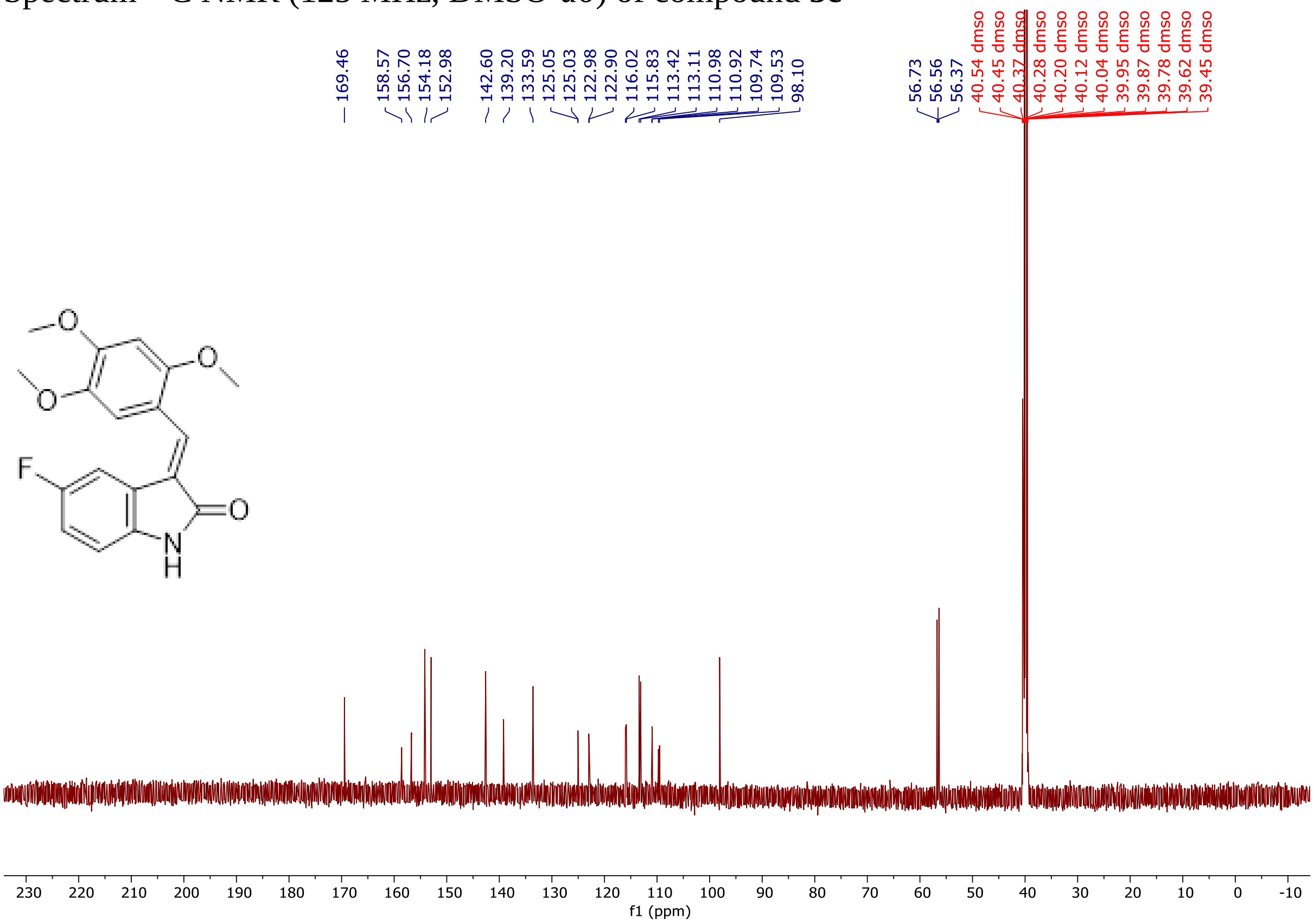

Spectrum ^{19}F NMR (470 MHz, DMSO-d_6) of compound **5e**

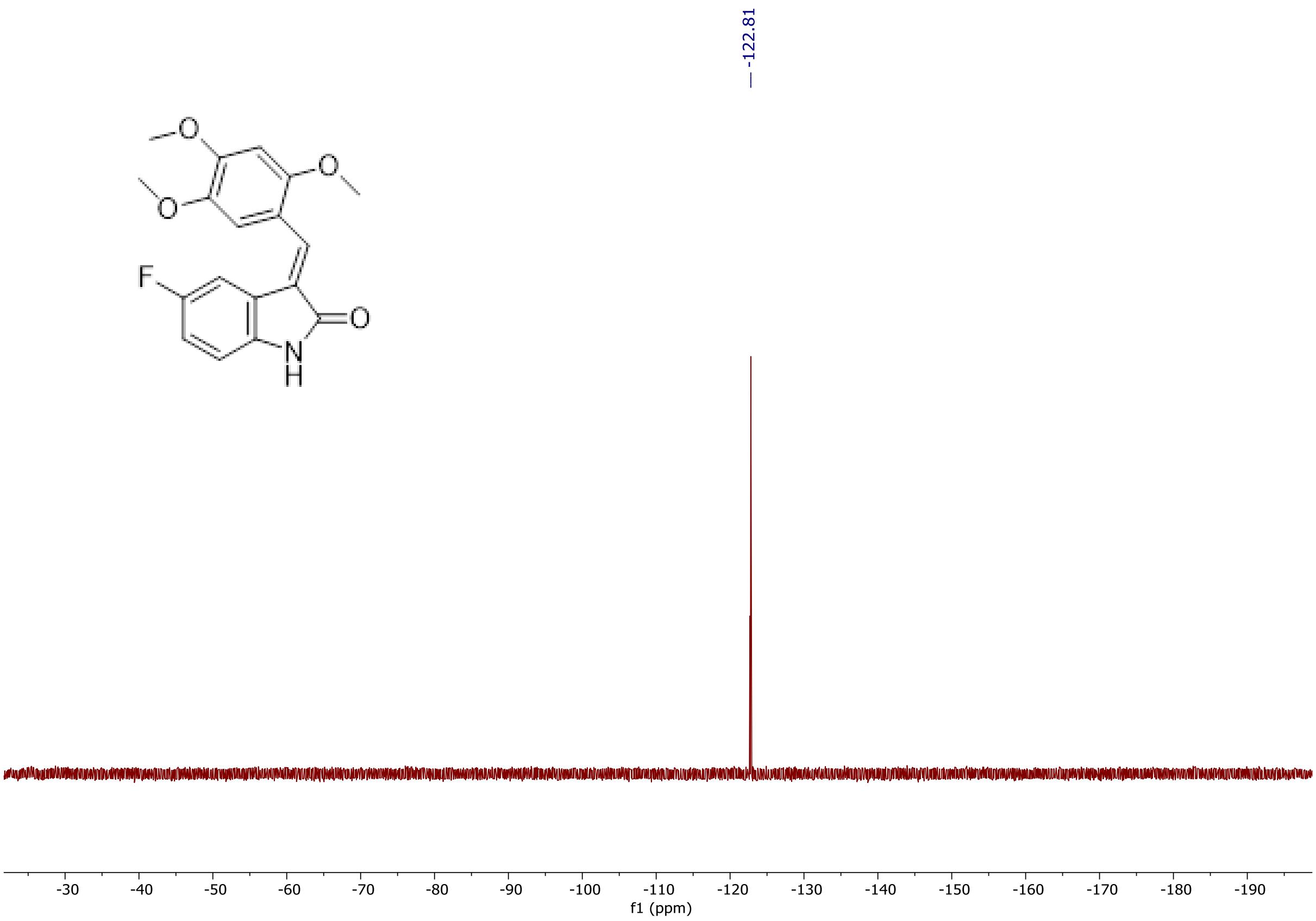

HRMS spectrum of compound **5e**

Single Mass Analysis
Tolerance = 5.0 PPM / DBE: min = -1.5, max = 50.0
Element prediction: Off
Number of isotope peaks used for i-FIT = 3

Monoisotopic Mass, Even Electron Ions
235 formula(e) evaluated with 1 results within limits (up to 50 best isotopic matches for each mass)
Elements Used:
C: 18-18 H: 0-100 N: 0-100 O: 0-100 F: 1-1

Robby_ERB5_pos 9 (0.153)
TOF MS ES+

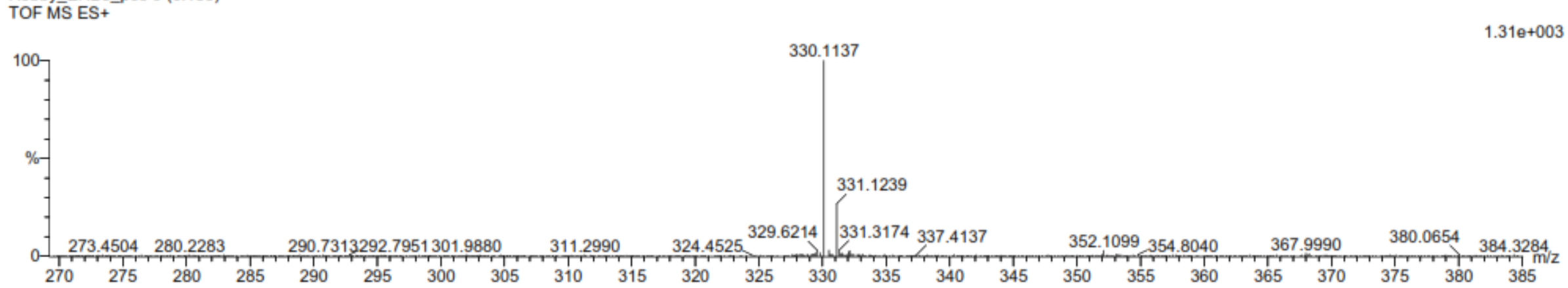

	Mass	Calc. Mass	mDa	PPM	DBE	i-FIT	i-FIT (Norm)	Formula
Minimum:					-1.5			
Maximum:			5.0	5.0	50.0			
	330.1137	330.1142	-0.5	-1.5	10.5	85.5	0.0	C18 H17 N O4 F

Spectrum ^{1}H NMR (500 MHz, DMSO-*d6*) of compound **5f** (*E/Z* = 93:7)

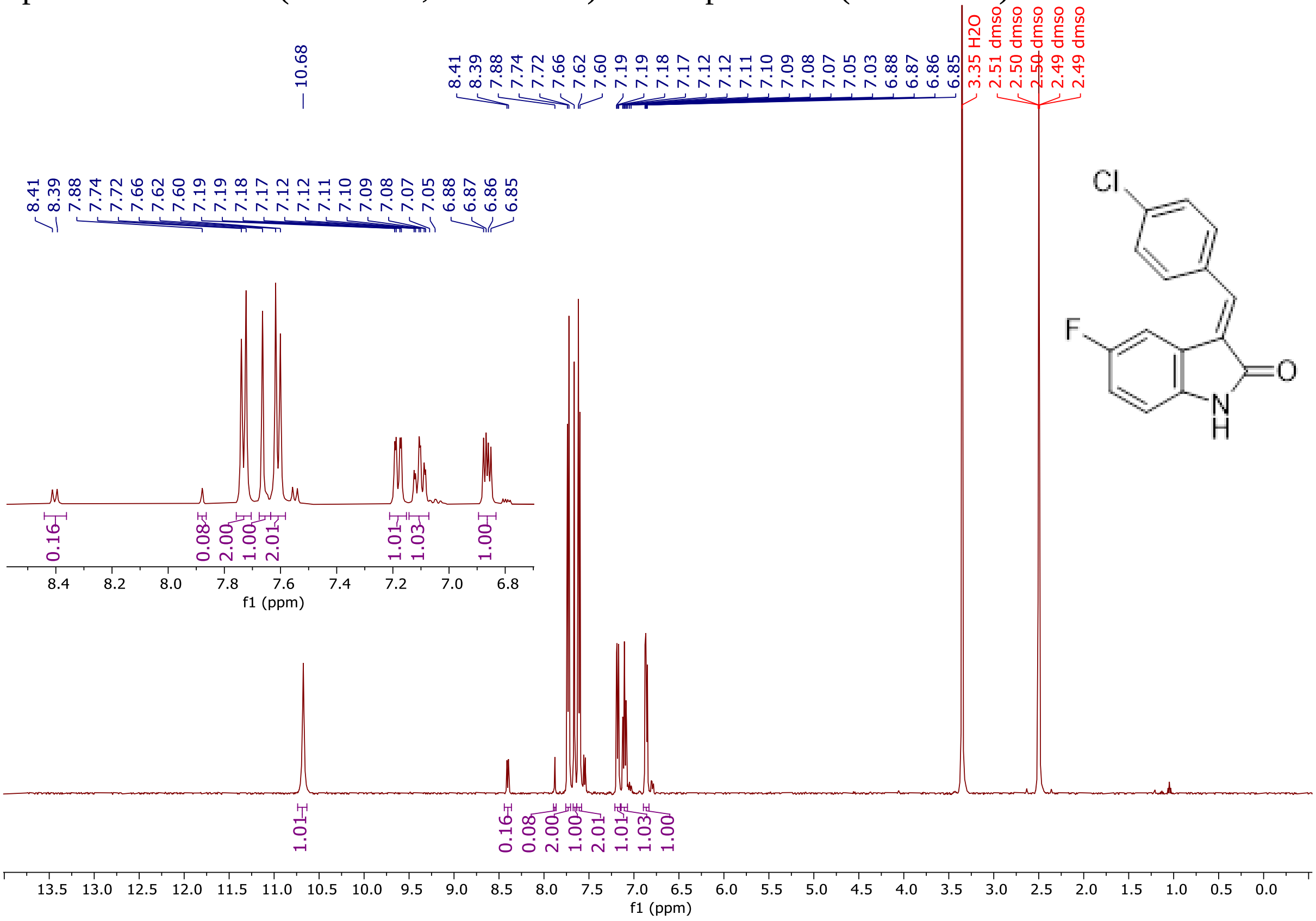

Spectrum ^{13}C NMR (125 MHz, DMSO-*d6*) of compound **5f**

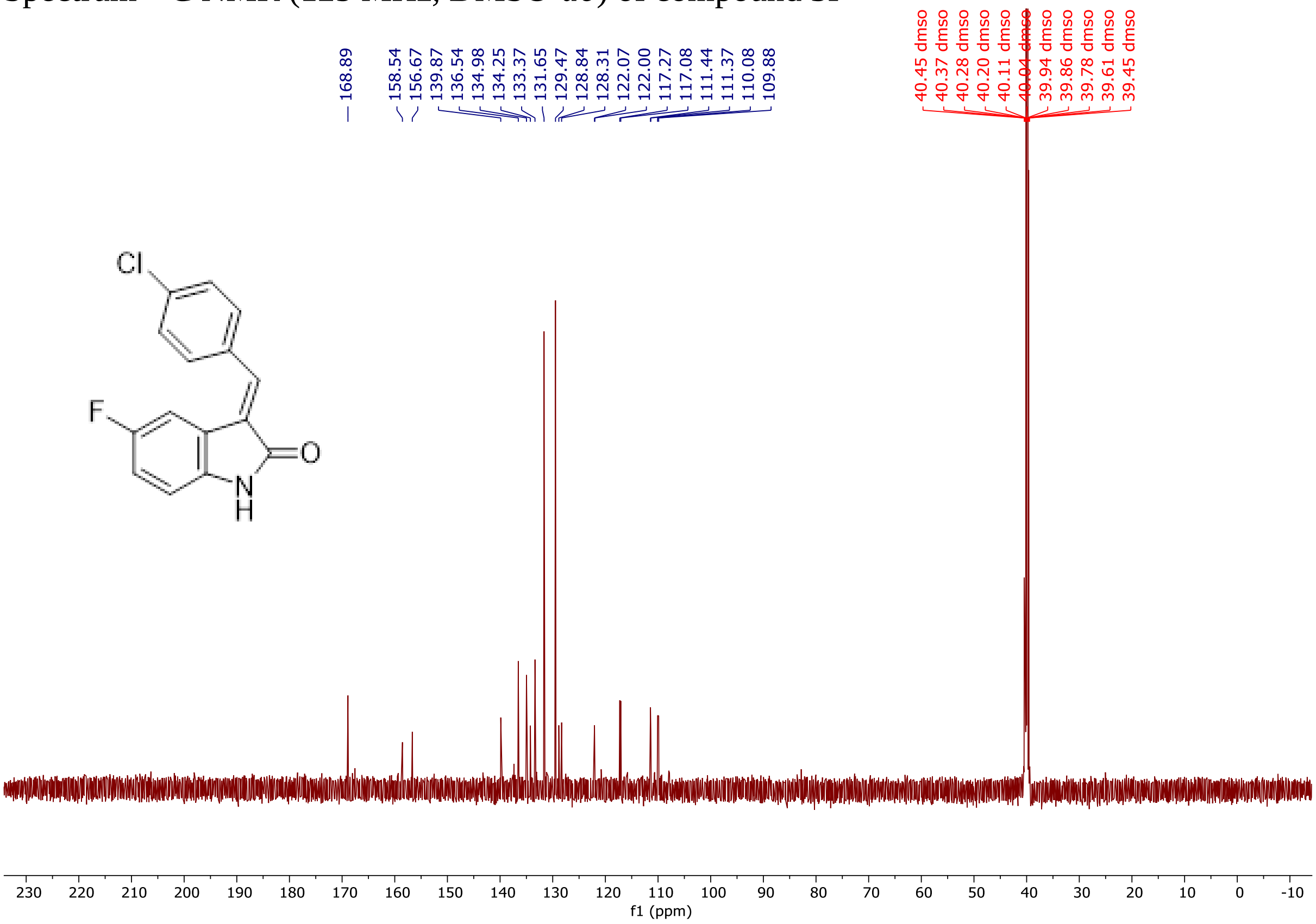

Spectrum ^{19}F NMR (470 MHz, DMSO-d_6) of compound **5f**

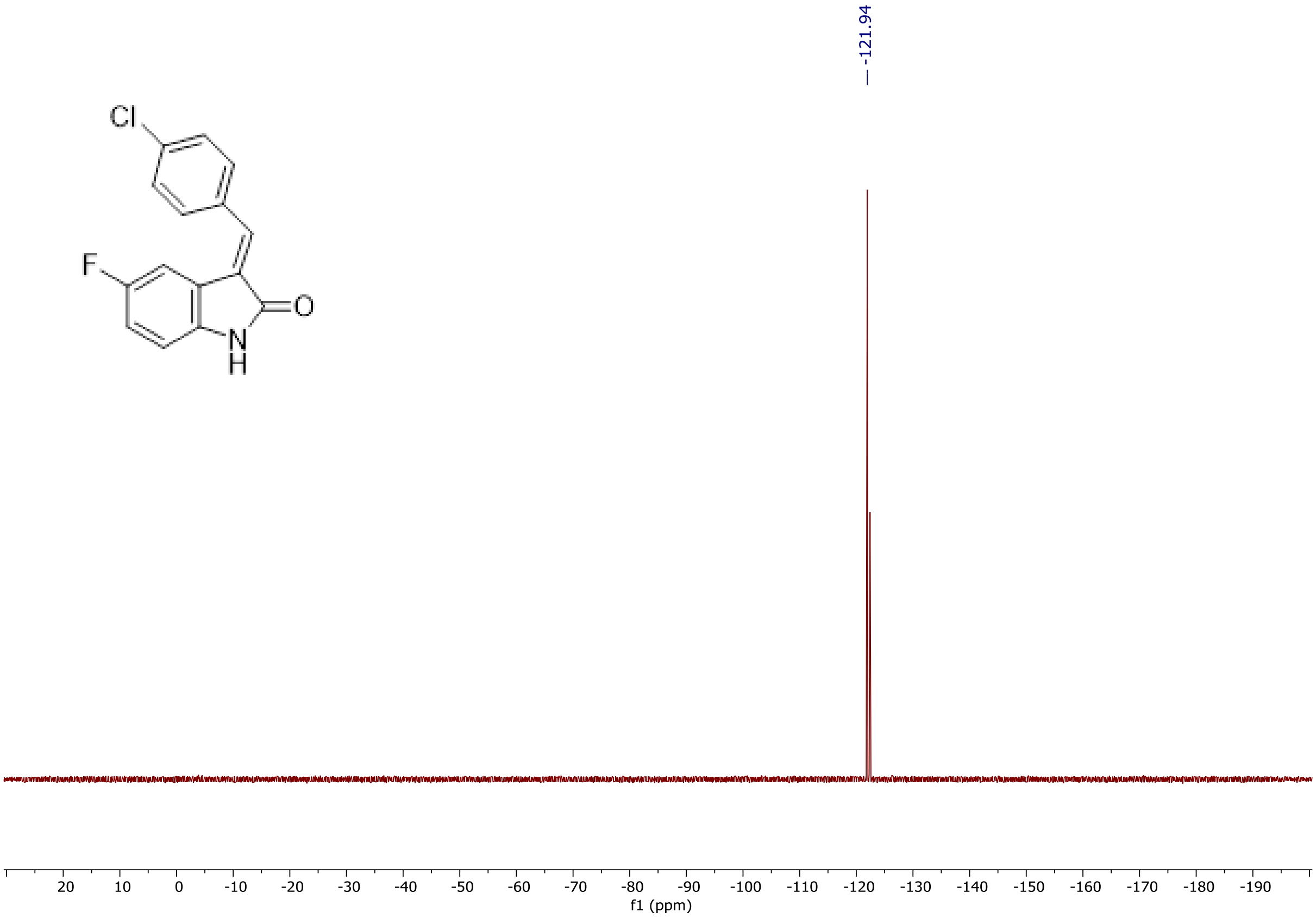

HRMS spectrum of compound **5f**

Single Mass Analysis
Tolerance = 5.0 PPM / DBE: min = -1.5, max = 50.0
Element prediction: Off
Number of isotope peaks used for i-FIT = 3

Monoisotopic Mass, Even Electron Ions
121 formula(e) evaluated with 1 results within limits (up to 50 best isotopic matches for each mass)
Elements Used:
C: 15-15 H: 0-100 N: 0-100 O: 0-100 Cl: 1-1 F: 1-1

Robby_ERB6_pos 91 (1.536)
TOF MS ES+

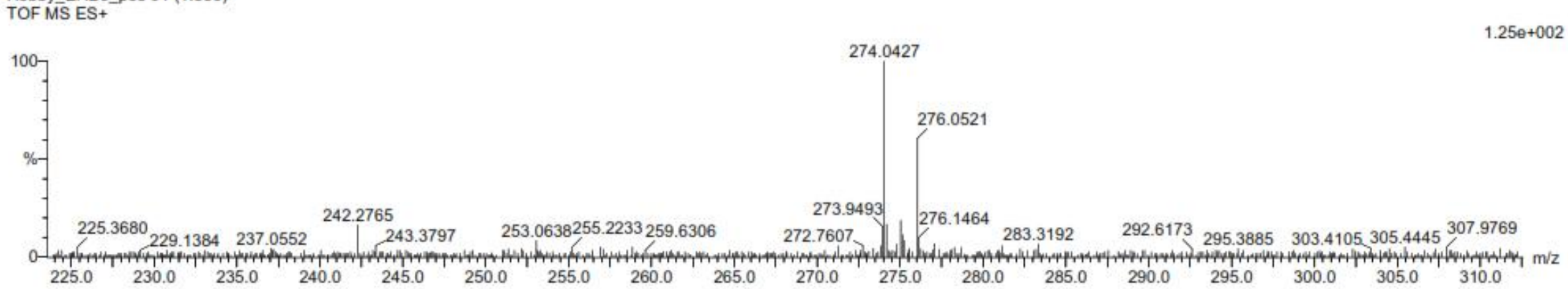

Mass	Calc. Mass	mDa	PPM	DBE	i-FIT	i-FIT (Norm)	Formula
Minimum:				-1.5			
Maximum:		5.0	5.0	50.0			
274.0427	274.0435	-0.8	-2.9	10.5	73.2	0.0	C15 H10 N O Cl F

Spectrum ^{1}H NMR (500 MHz, DMSO-*d6*) of compound **5g** (*E*/*Z* = 97:3)

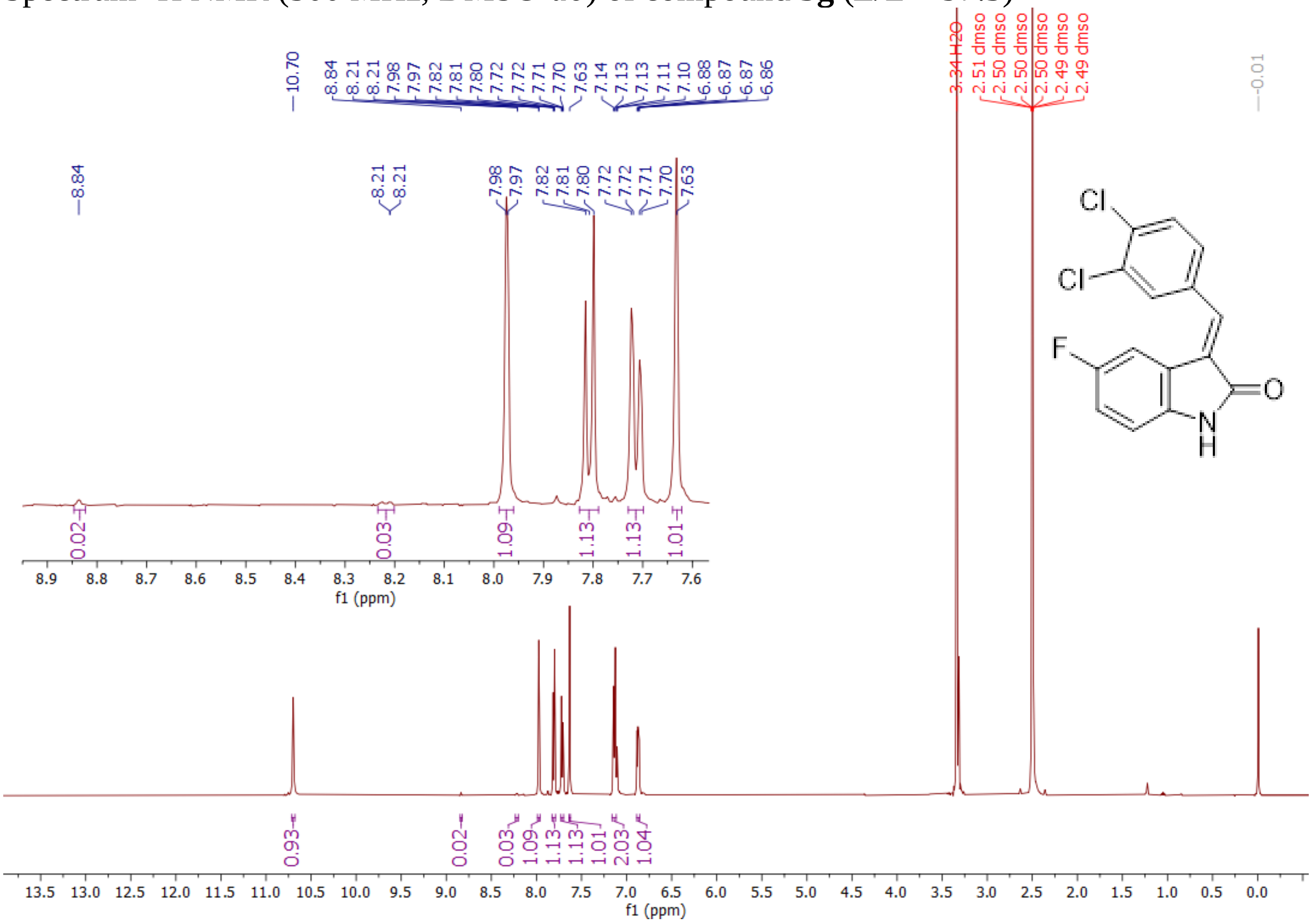

Spectrum ^{13}C NMR (125 MHz, DMSO-*d6*) of compound **5g**

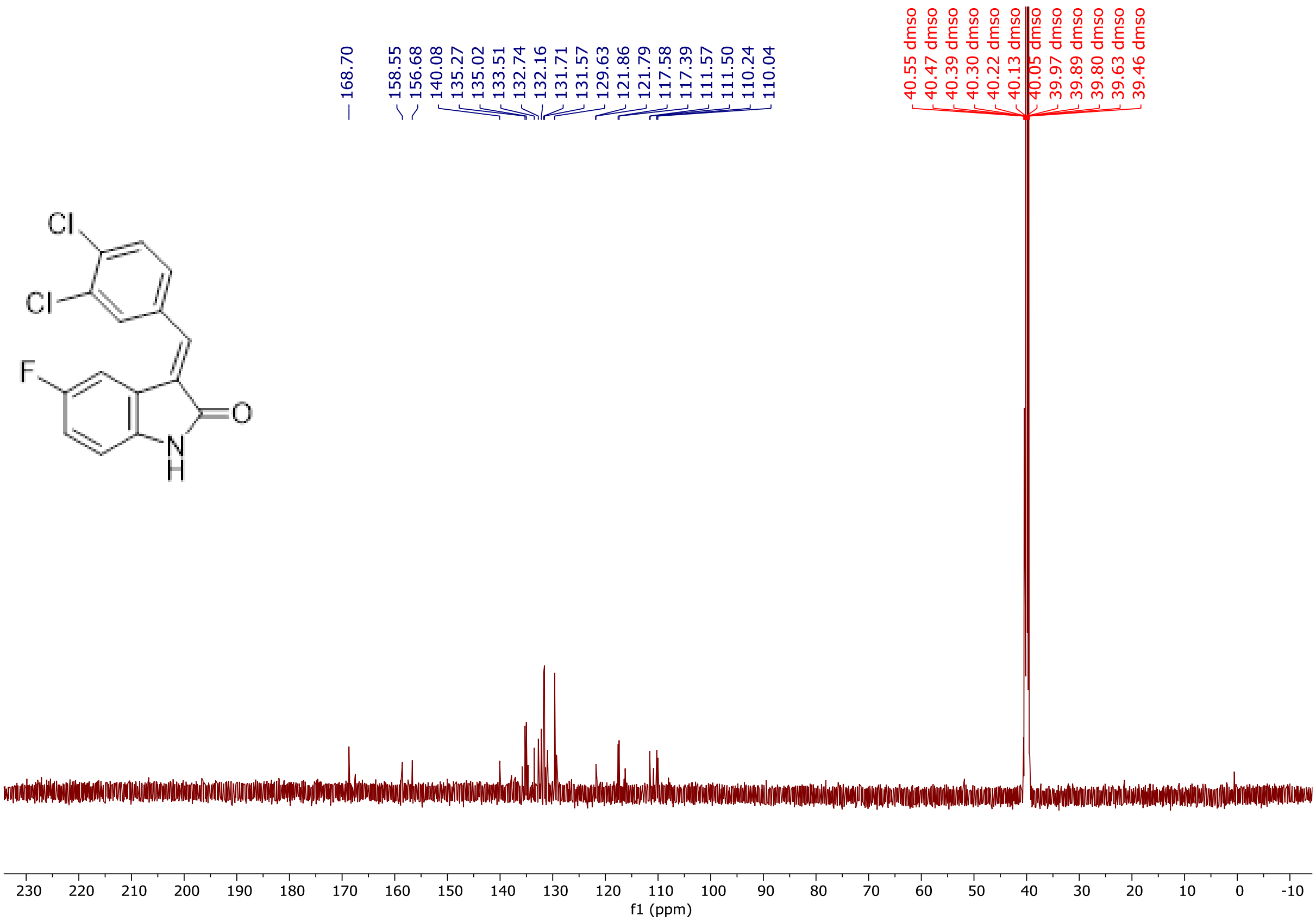

Spectrum ^{19}F NMR (470 MHz, DMSO-d_6) of compound **5g**

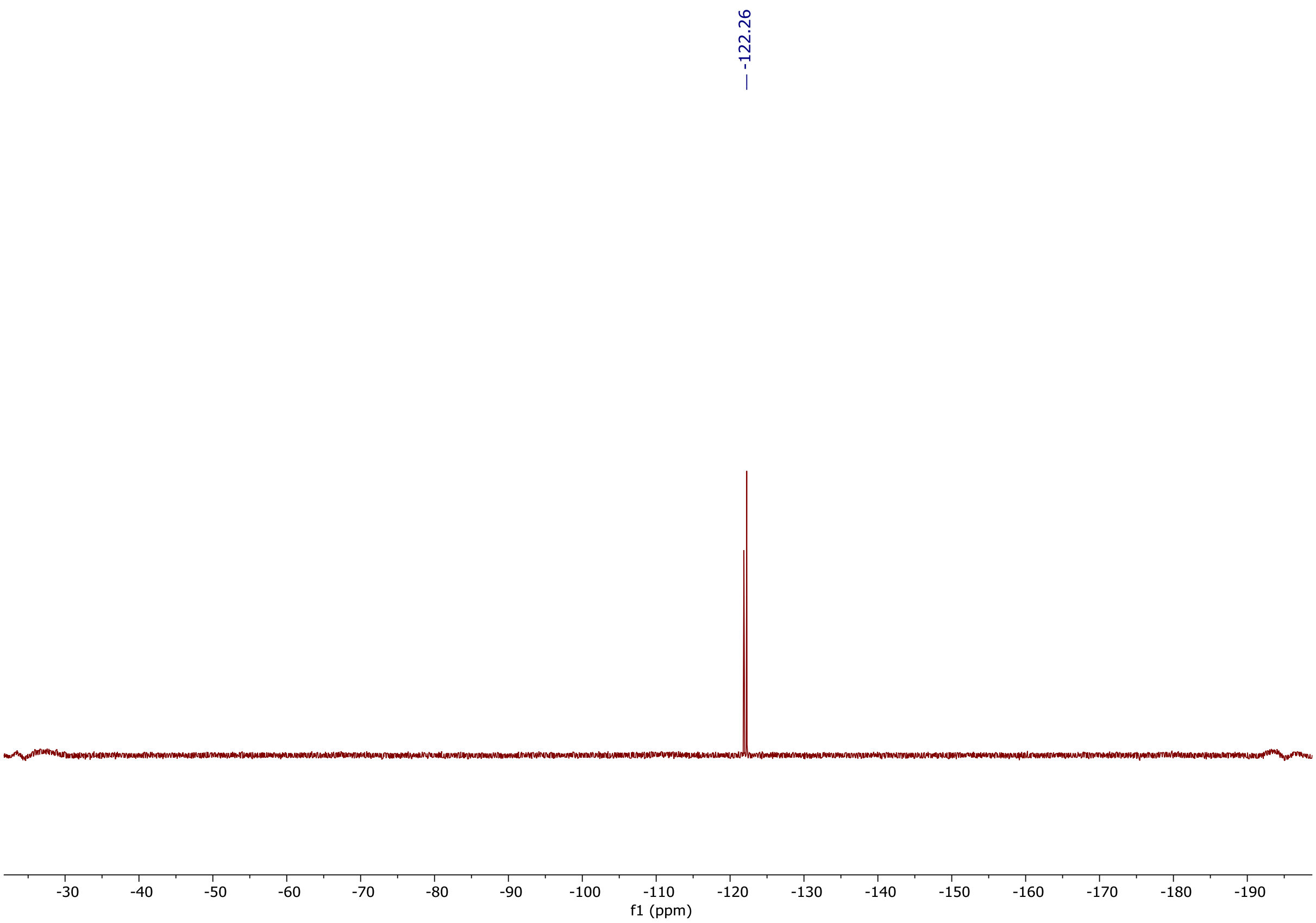

HRMS spectrum of compound **5g**

Single Mass Analysis
Tolerance = 5.0 PPM / DBE: min = -1.5, max = 50.0
Element prediction: Off
Number of isotope peaks used for i-FIT = 3

Monoisotopic Mass, Even Electron Ions
119 formula(e) evaluated with 1 results within limits (up to 50 best isotopic matches for each mass)
Elements Used:
C: 15-15 H: 0-100 N: 0-100 O: 0-100 Cl: 2-2 F: 1-1

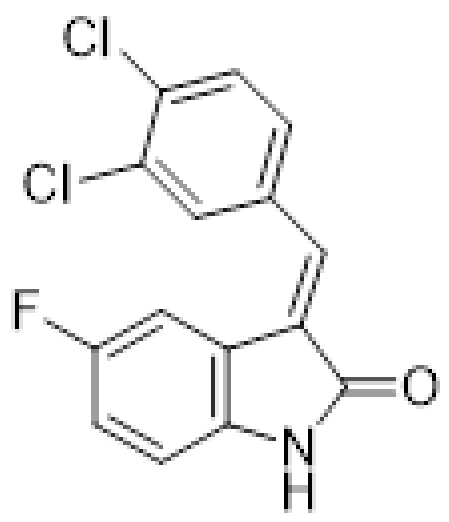

Robby_ERB8_neg 8 (0.119) Cm (4:8)
TOF MS ES-

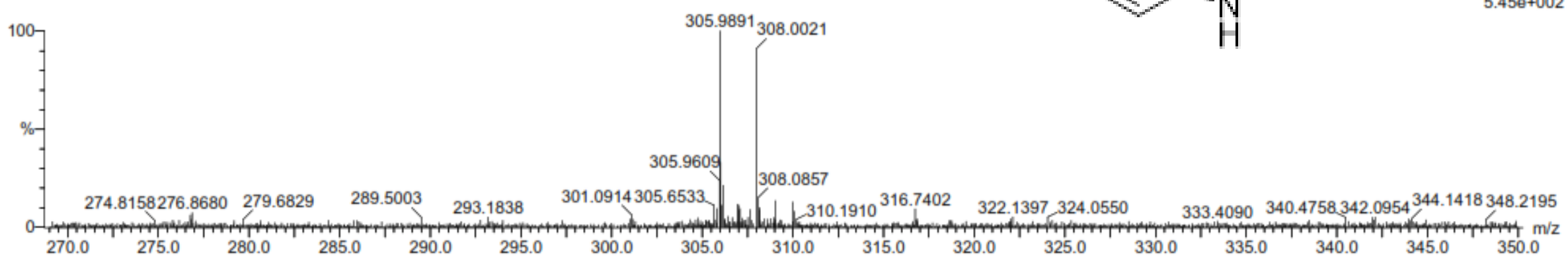

Mass	Calc. Mass	mDa	PPM	DBE	i-FIT	i-FIT (Norm)	Formula
Minimum:				-1.5			
Maximum:		5.0	5.0	50.0			
305.9891	305.9889	0.2	0.7	11.5	258.8	0.0	C15 H7 N O Cl2 F

Spectrum ^{1}H NMR (500 MHz, DMSO-*d6*) of compound **5h** (*E/Z* = 100:0)

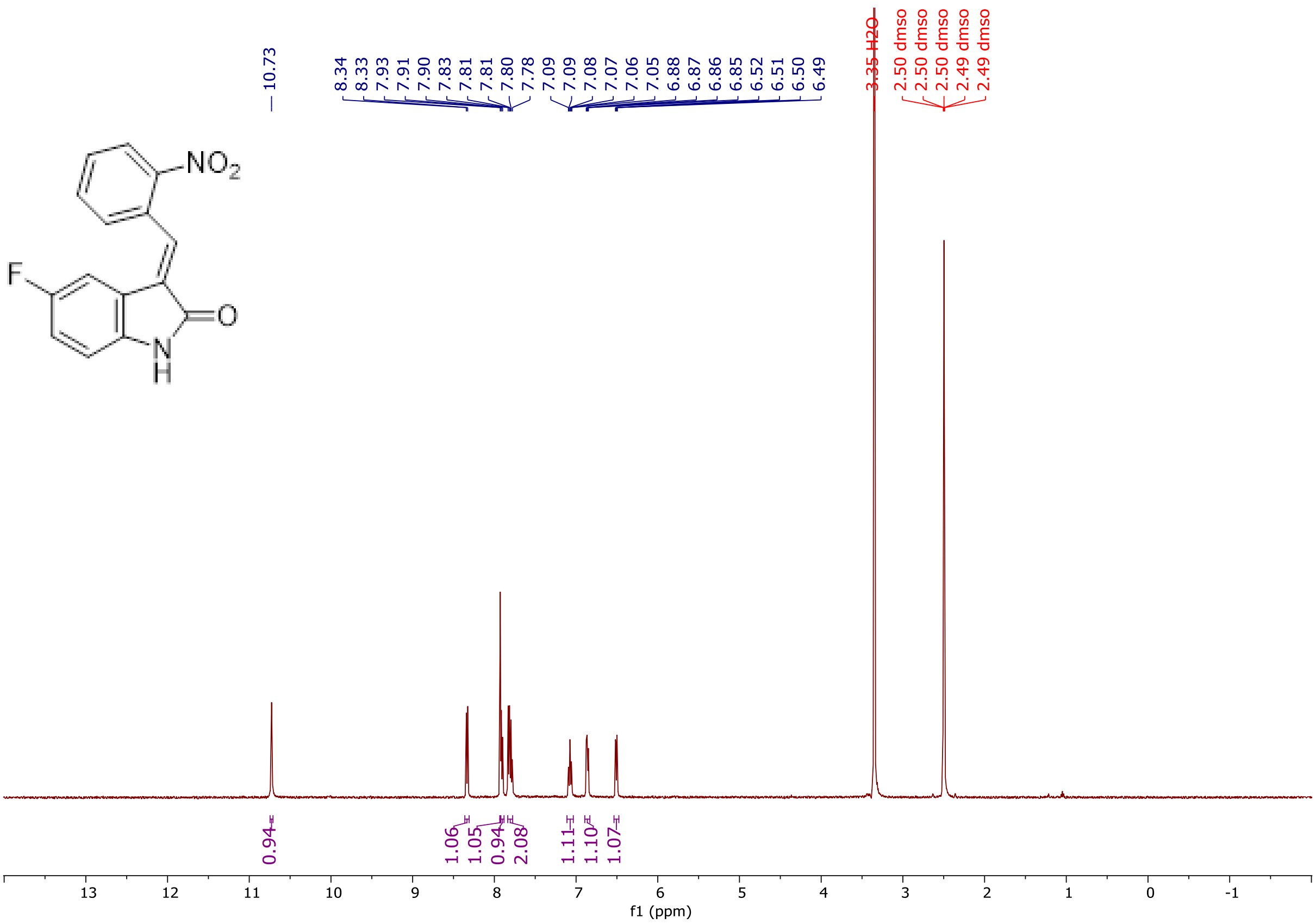

Spectrum ^{13}C NMR (125 MHz, DMSO-*d6*) of compound **5h**

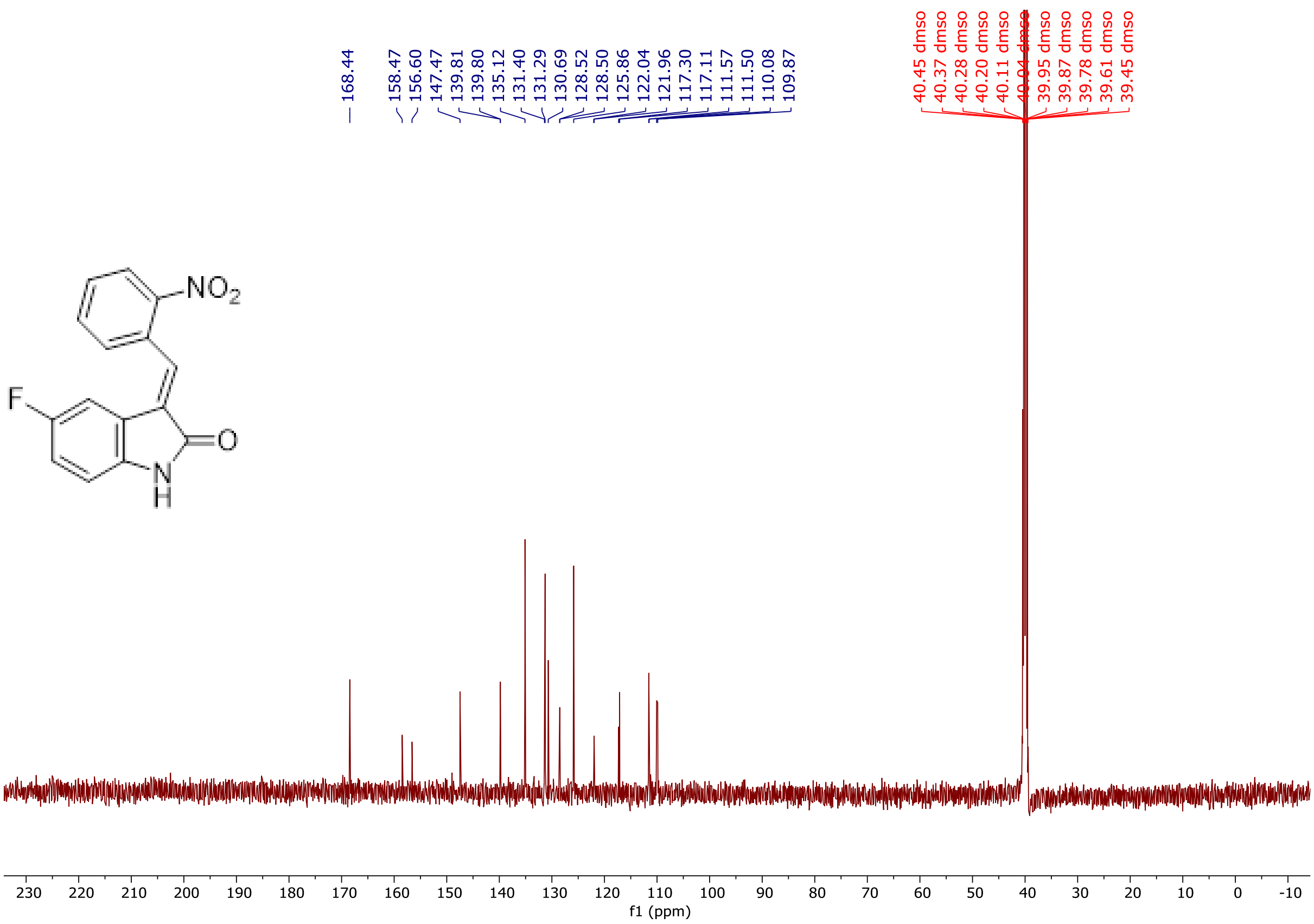

Spectrum ^{19}F NMR (470 MHz, DMSO-d_6) of compound **5h**

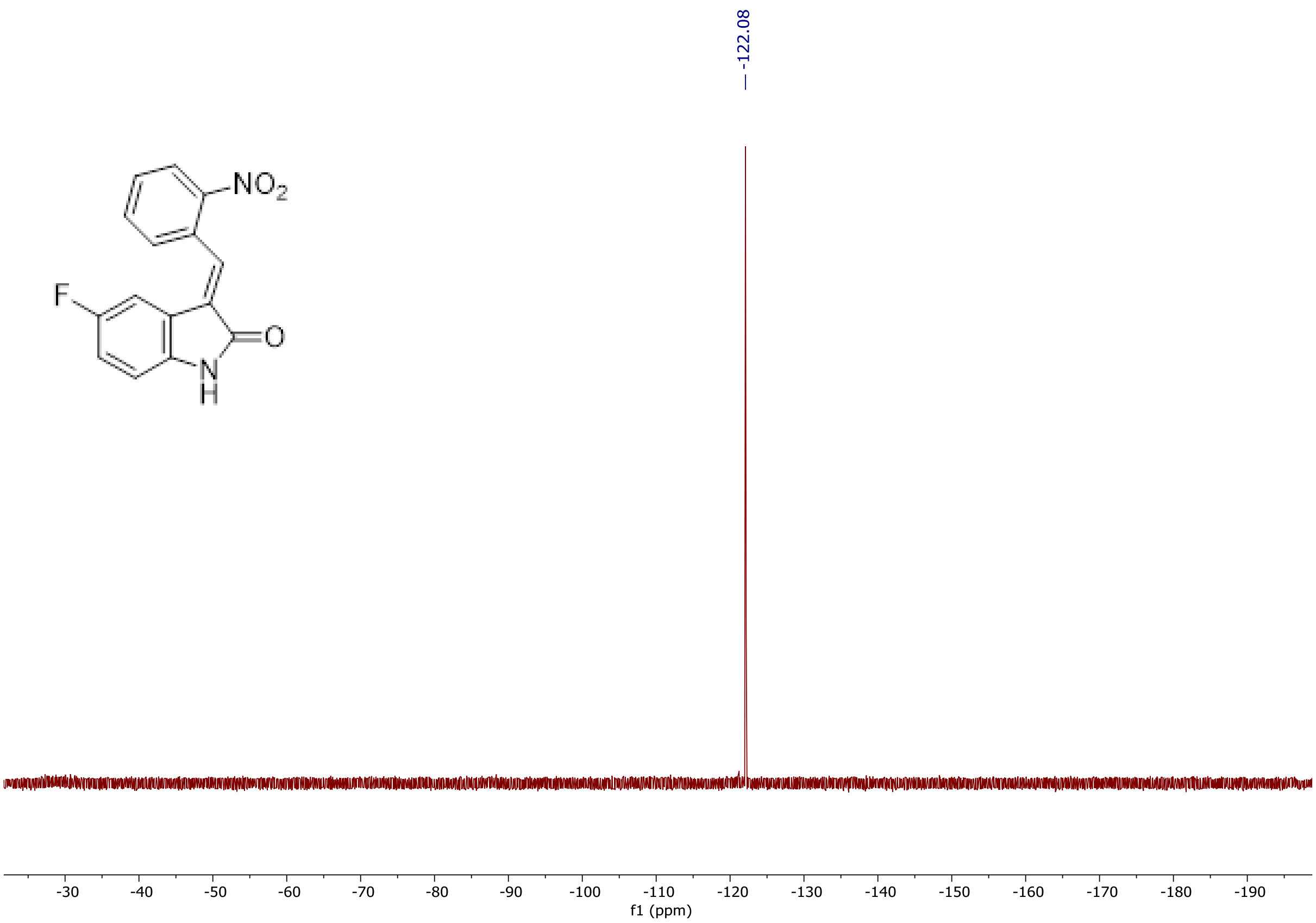

HRMS spectrum of compound **5h**

Single Mass Analysis
Tolerance = 5.0 PPM / DBE: min = -1.5, max = 50.0
Element prediction: Off
Number of isotope peaks used for i-FIT = 3

Monoisotopic Mass, Even Electron Ions
174 formula(e) evaluated with 1 results within limits (up to 50 best isotopic matches for each mass)
Elements Used:
C: 15-15 H: 0-100 N: 0-100 O: 0-100 F: 1-1

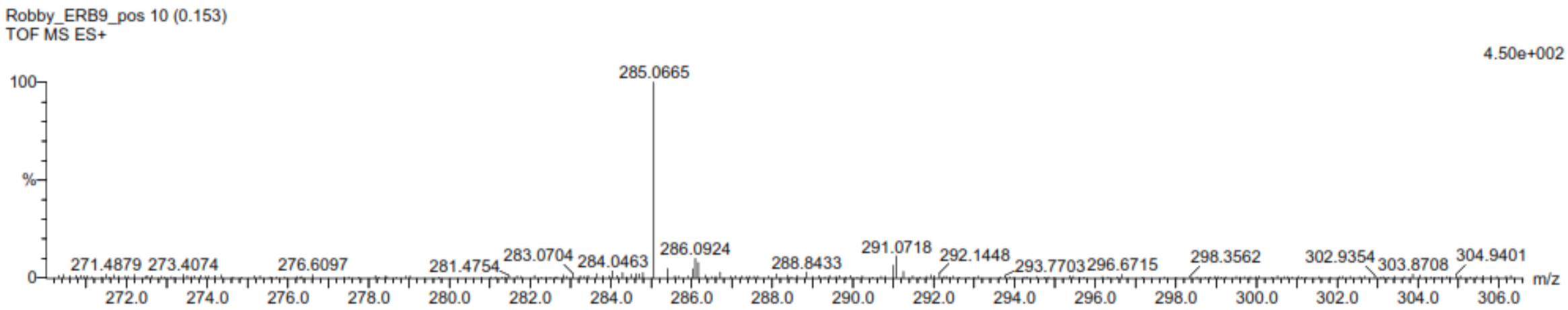

Minimum:				-1.5			
Maximum:		5.0	5.0	50.0			
Mass	Calc. Mass	mDa	PPM	DBE	i-FIT	i-FIT (Norm)	Formula
285.0665	285.0675	-1.0	-3.5	11.5	73.4	0.0	C15 H10 N2 O3 F

Spectrum ^{1}H NMR (500 MHz, DMSO-*d6*) of compound **5i** (*E/Z* = 96:4)

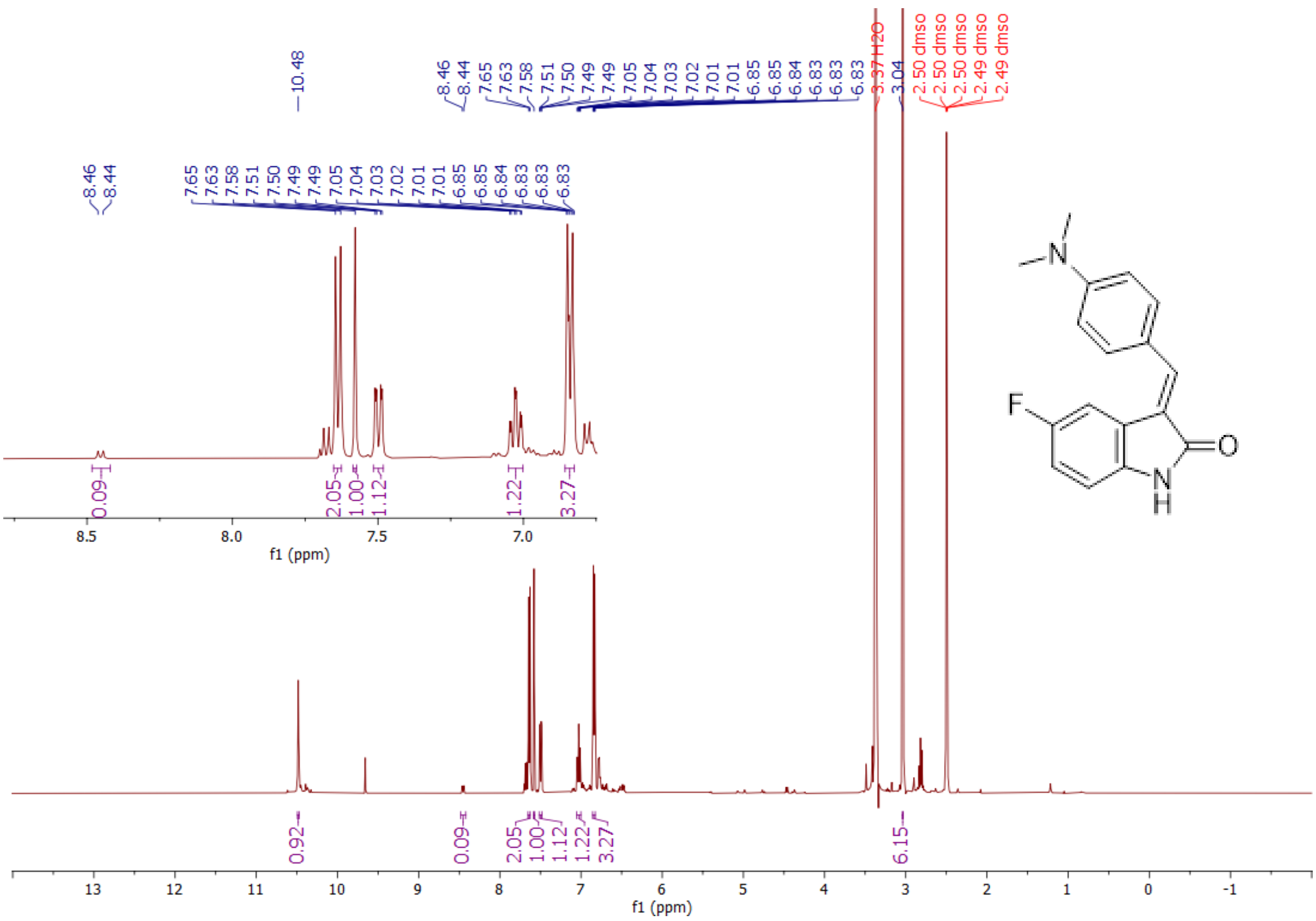

Spectrum ^{13}C NMR (125 MHz, DMSO-*d6*) of compound **5i**

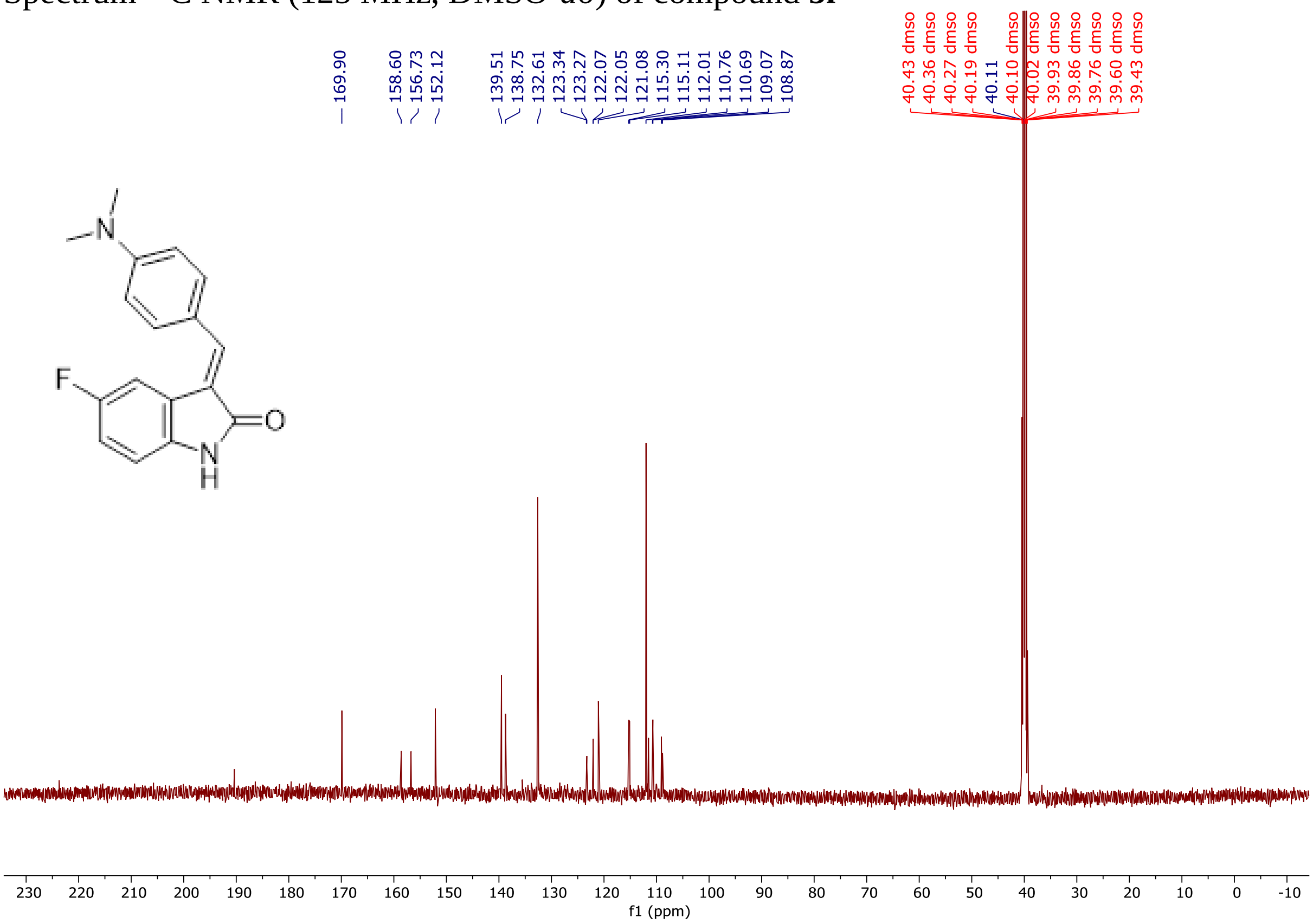

Spectrum ^{19}F NMR (470 MHz, DMSO-d_6) of compound **5i**

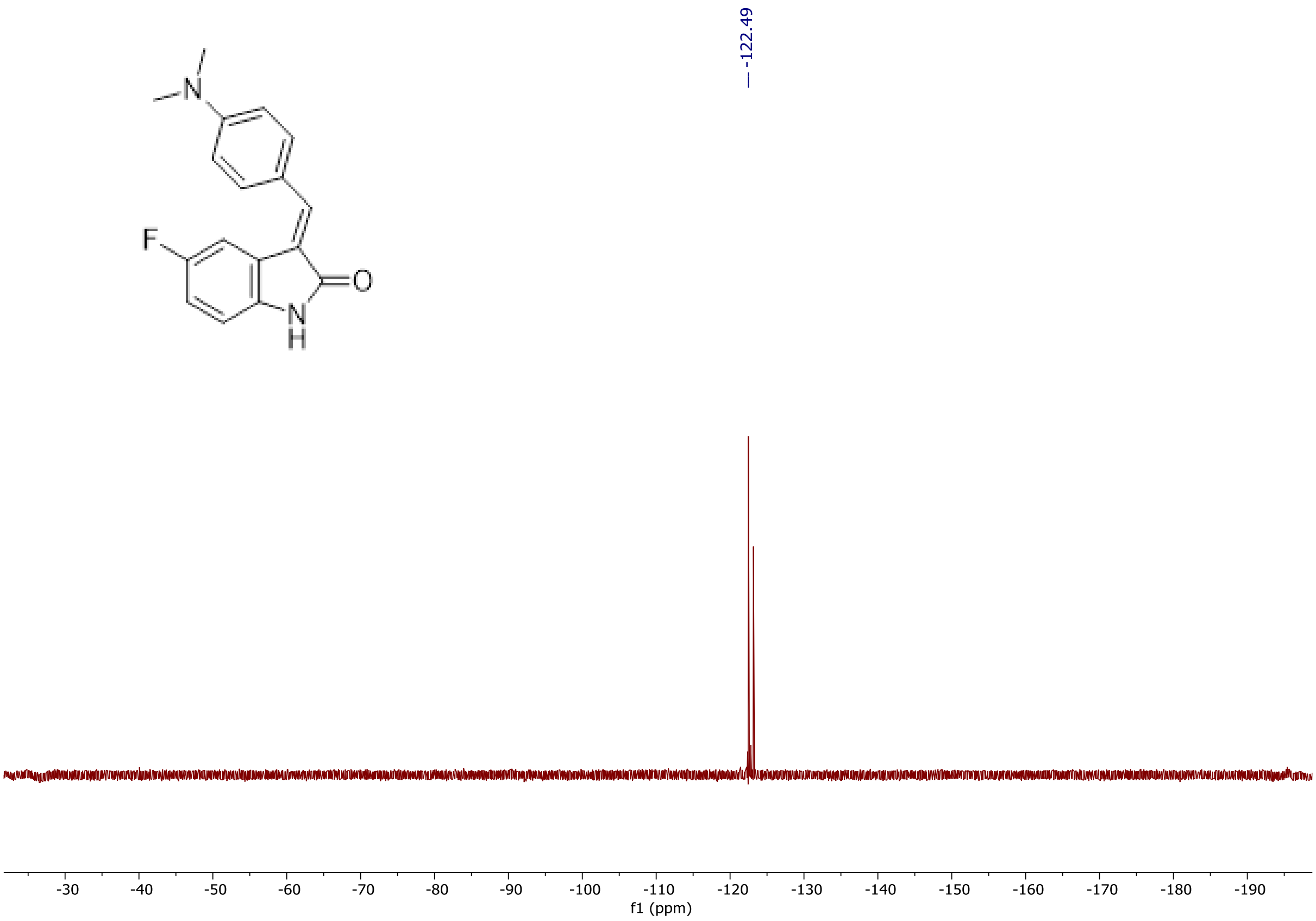

HRMS spectrum of compound **5i**

Single Mass Analysis
Tolerance = 5.0 PPM / DBE: min = -1.5, max = 50.0
Element prediction: Off
Number of isotope peaks used for i-FIT = 3

Monoisotopic Mass, Even Electron Ions
172 formula(e) evaluated with 1 results within limits (up to 50 best isotopic matches for each mass)
Elements Used:
C: 17-17 H: 0-100 N: 0-100 O: 0-100 F: 1-1

Robby_ERB11_pos 46 (0.768)
TOF MS ES+

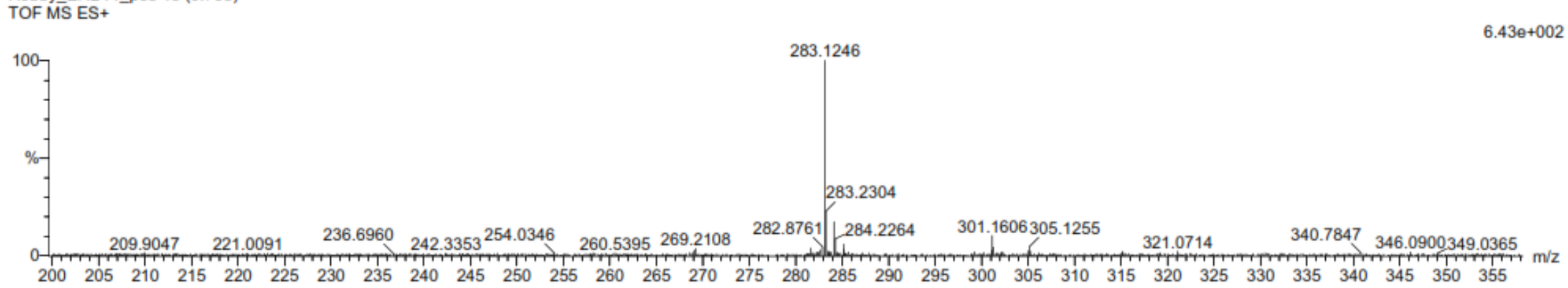

Mass	Calc. Mass	mDa	PPM	DBE	i-FIT	i-FIT (Norm)	Formula
Minimum:				-1.5			
Maximum:		5.0	5.0	50.0			
283.1246	283.1247	-0.1	-0.4	10.5	101.4	0.0	C17 H16 N2 O F

Spectrum ^{1}H NMR (500 MHz, DMSO-*d6*) of compound **5j** (*E*/*Z* = 97:3)

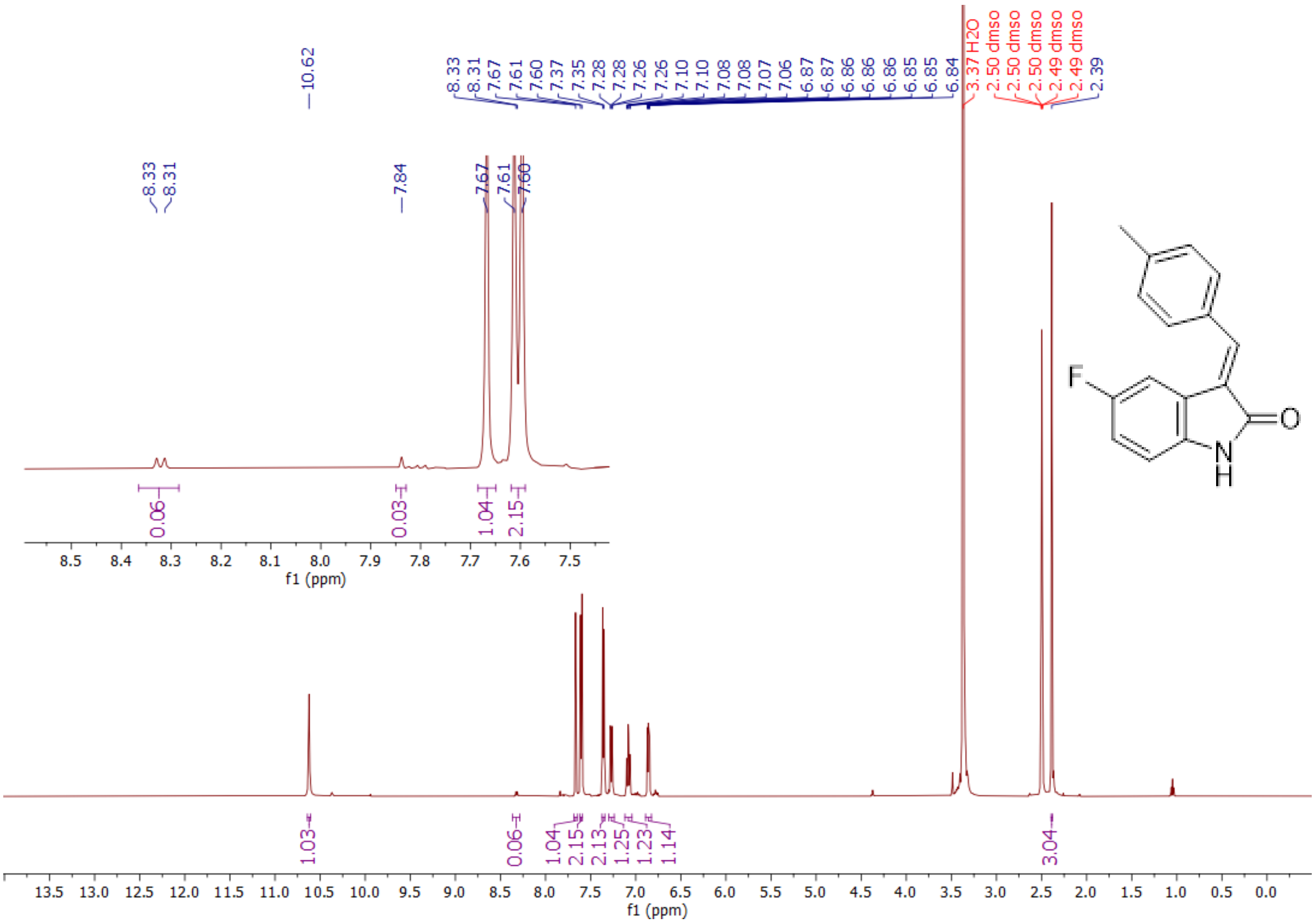

Spectrum ^{13}C NMR (125 MHz, DMSO-*d6*) of compound **5j**

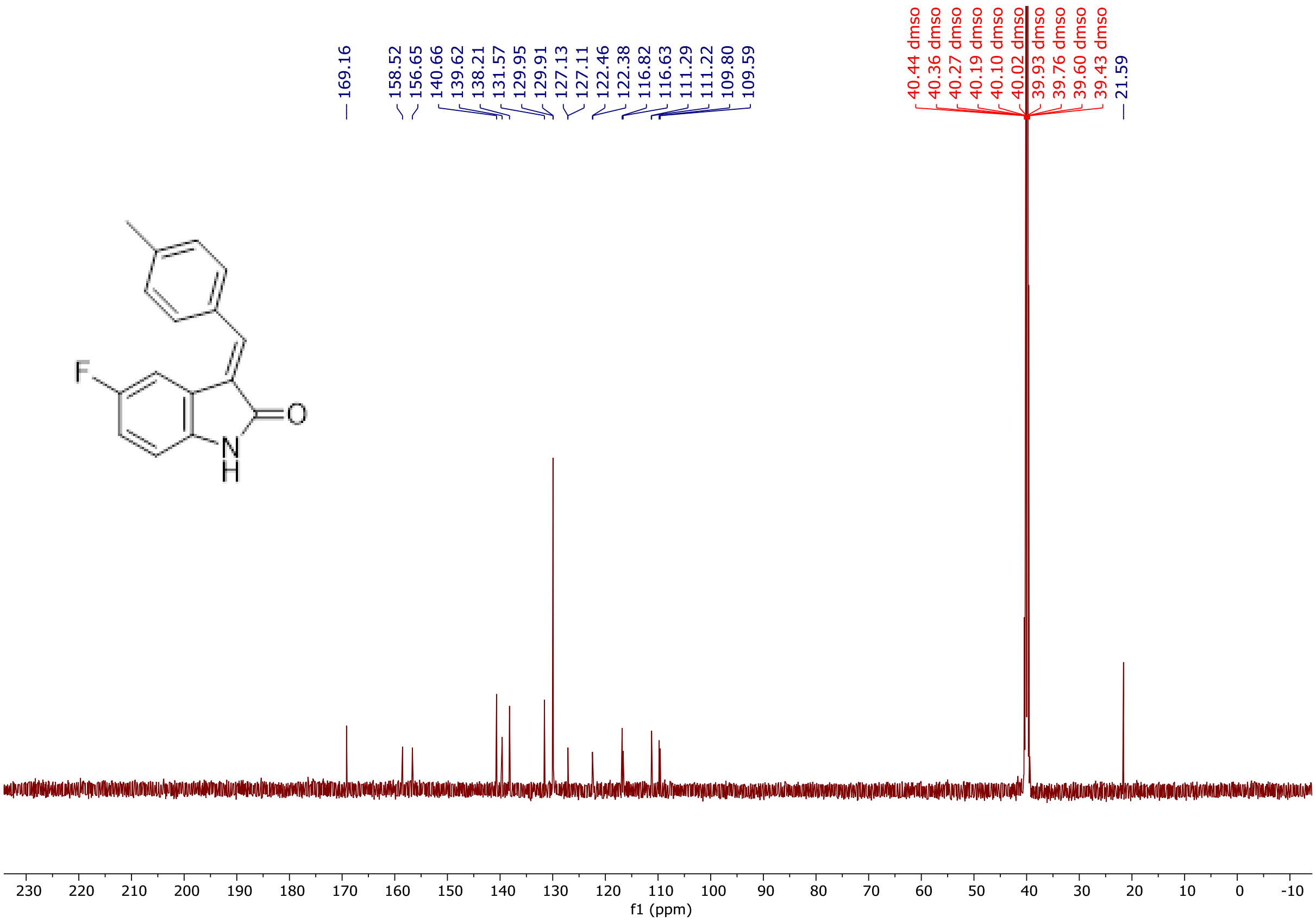

Spectrum ^{19}F NMR (470 MHz, DMSO-d_6) of compound **5j**

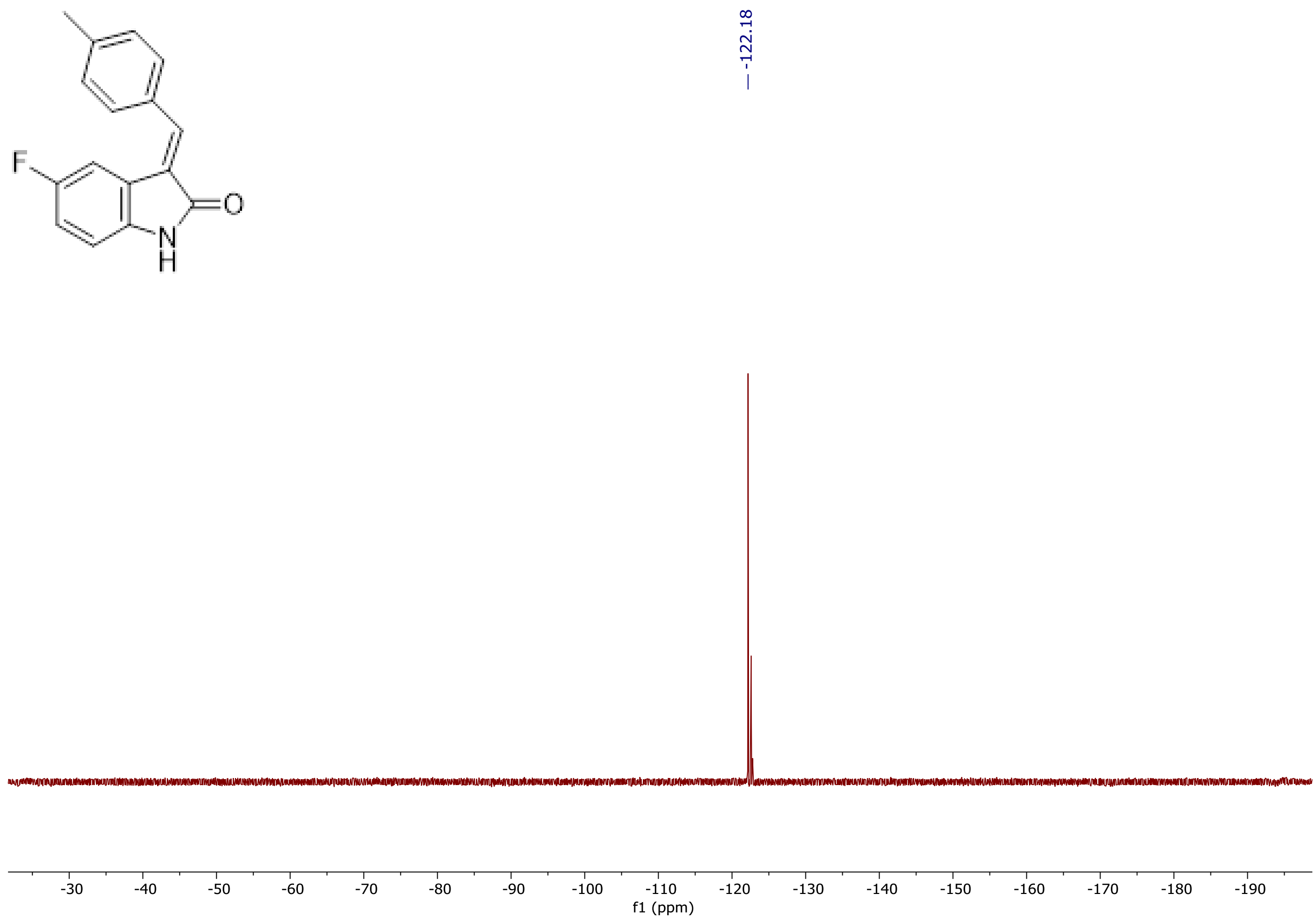

HRMS spectrum of compound **5j**

Single Mass Analysis
Tolerance = 5.0 PPM / DBE: min = -1.5, max = 50.0
Element prediction: Off
Number of isotope peaks used for i-FIT = 3

Monoisotopic Mass, Even Electron Ions
137 formula(e) evaluated with 1 results within limits (up to 50 best isotopic matches for each mass)
Elements Used:
C: 16-16 H: 0-100 N: 0-100 O: 0-100 F: 1-1

Robby_ERB13_pos 33 (0.546)
TOF MS ES+

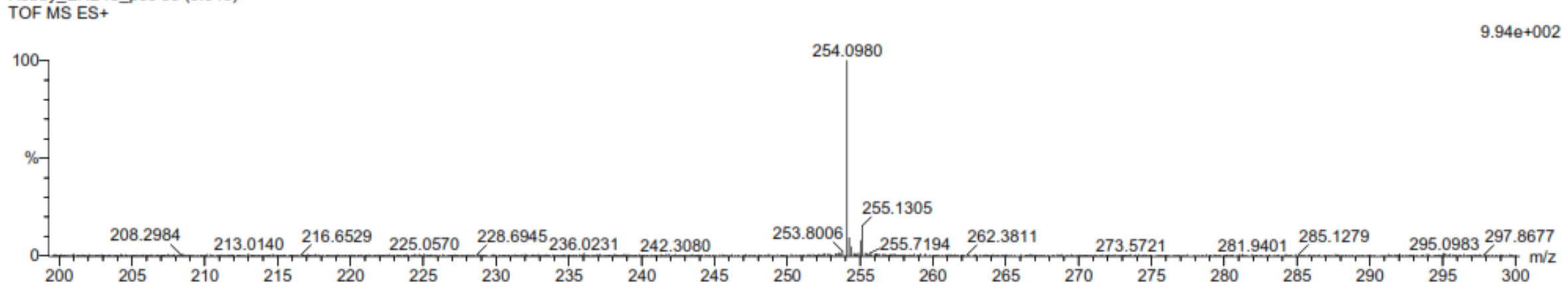

Minimum:				-1.5			
Maximum:		5.0	5.0	50.0			
Mass	Calc. Mass	mDa	PPM	DBE	i-FIT	i-FIT (Norm)	Formula
254.0980	254.0981	-0.1	-0.4	10.5	108.1	0.0	C16 H13 N O F

Spectrum ^{1}H NMR (500 MHz, DMSO-*d6*) of compound **5k** (*E/Z* = 86:14)

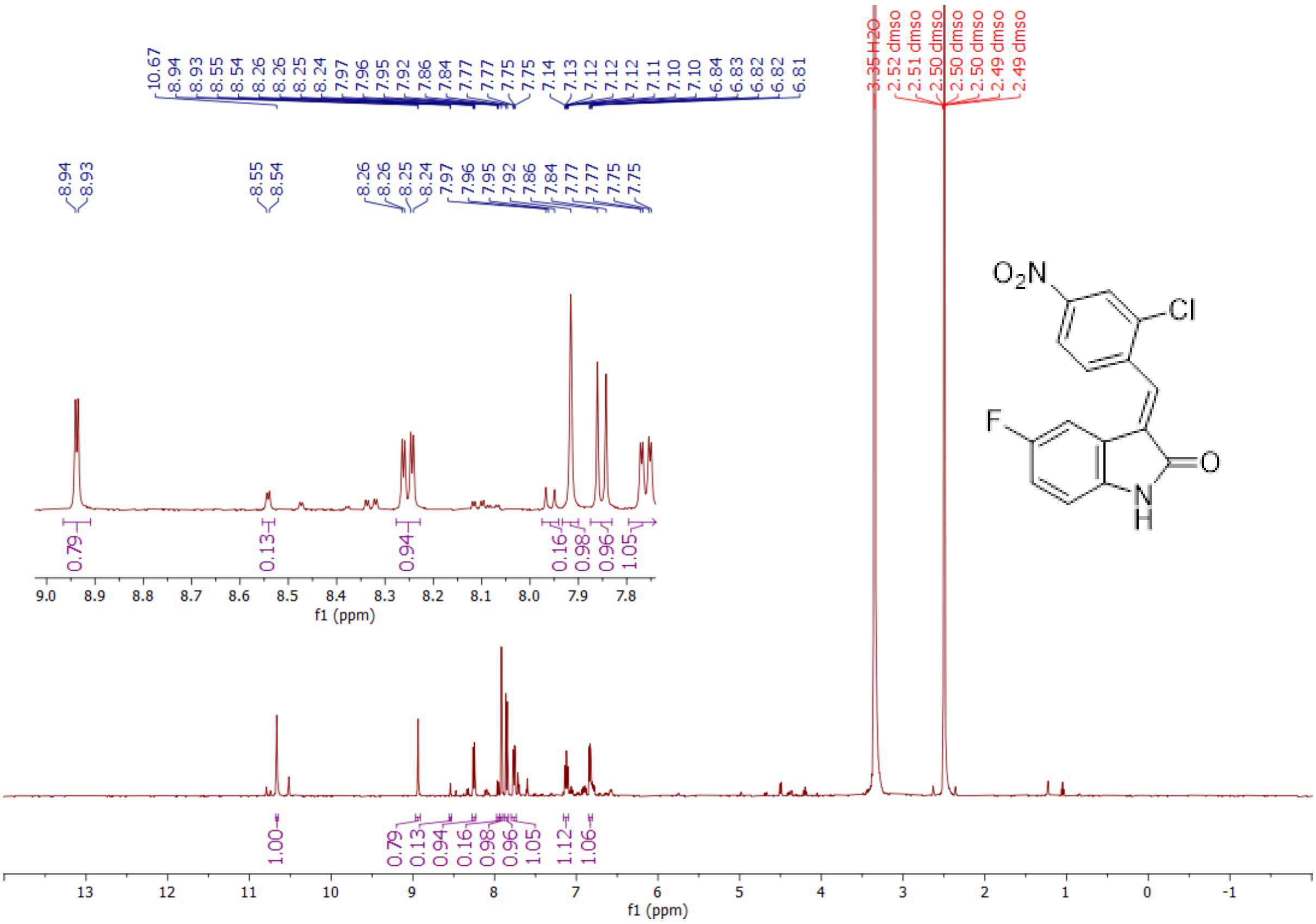

Spectrum ^{13}C NMR (125 MHz, DMSO-*d6*) of compound **5k**

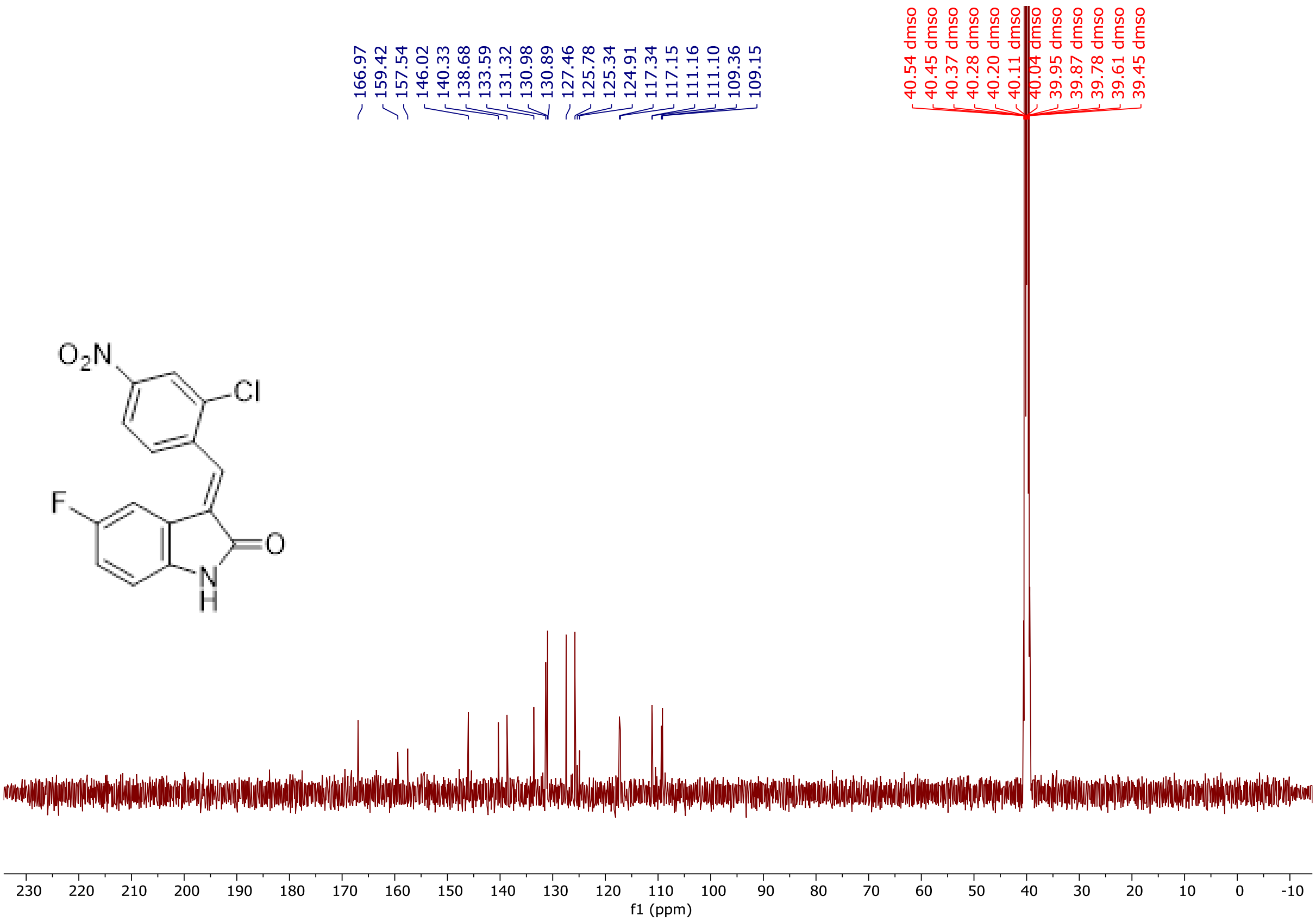

Spectrum ^{19}F NMR (470 MHz, DMSO-d_6) of compound **5k**

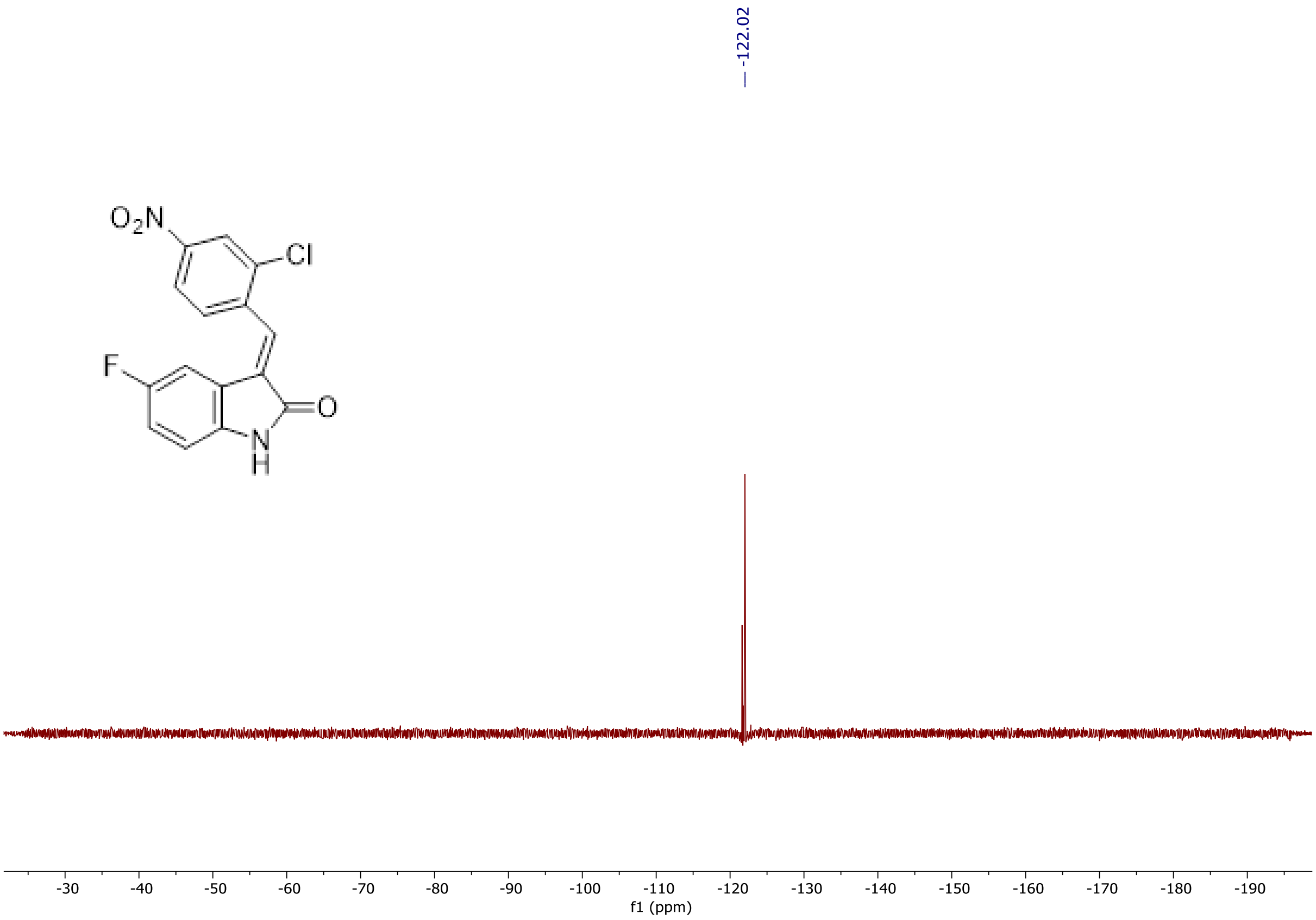

Spectrum ^{1}H NMR (500 MHz, DMSO-*d6*) of compound **5l** (*E/Z* = 100:0)

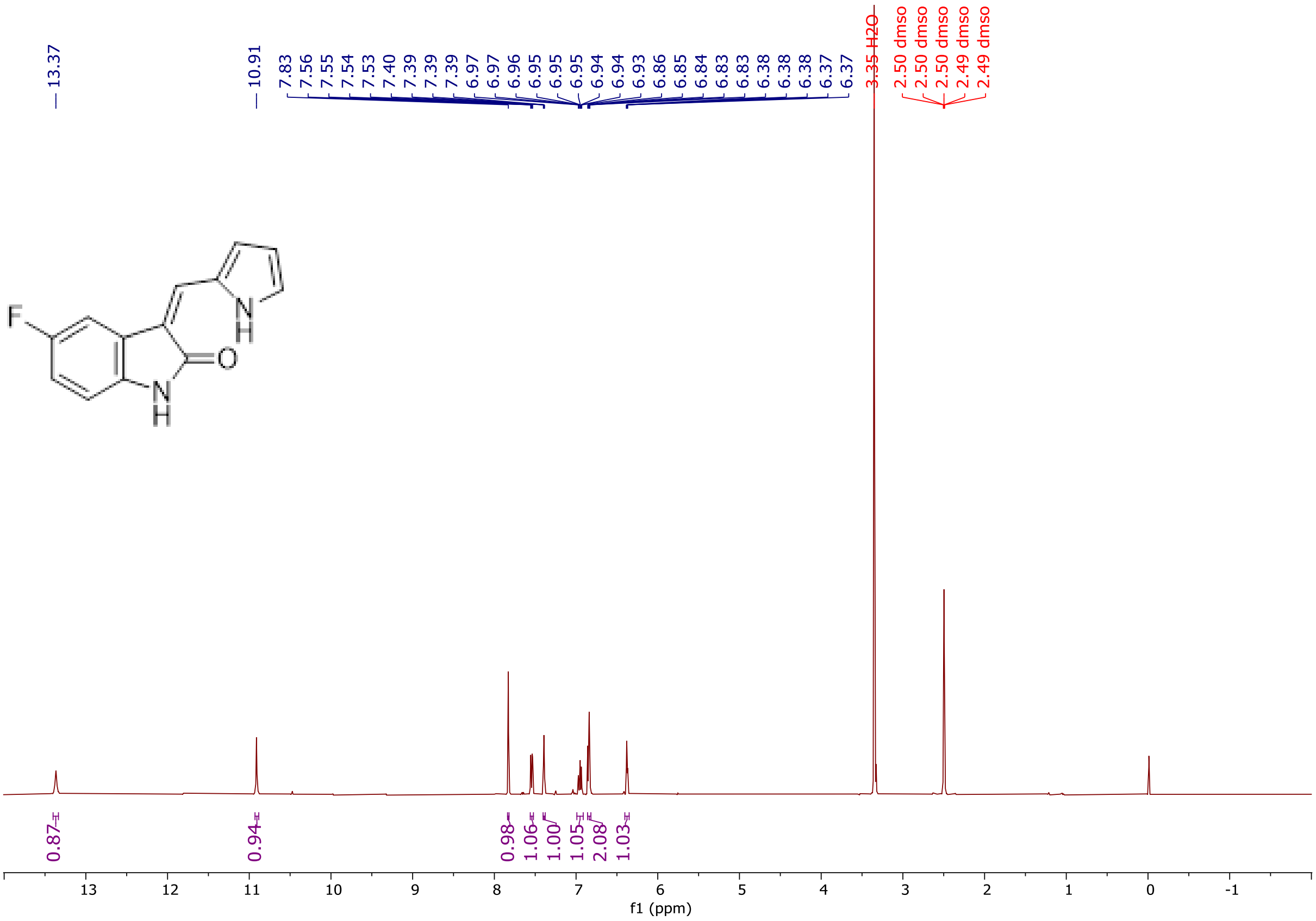

Spectrum ^{13}C NMR (125 MHz, DMSO-*d6*) of compound **5l**

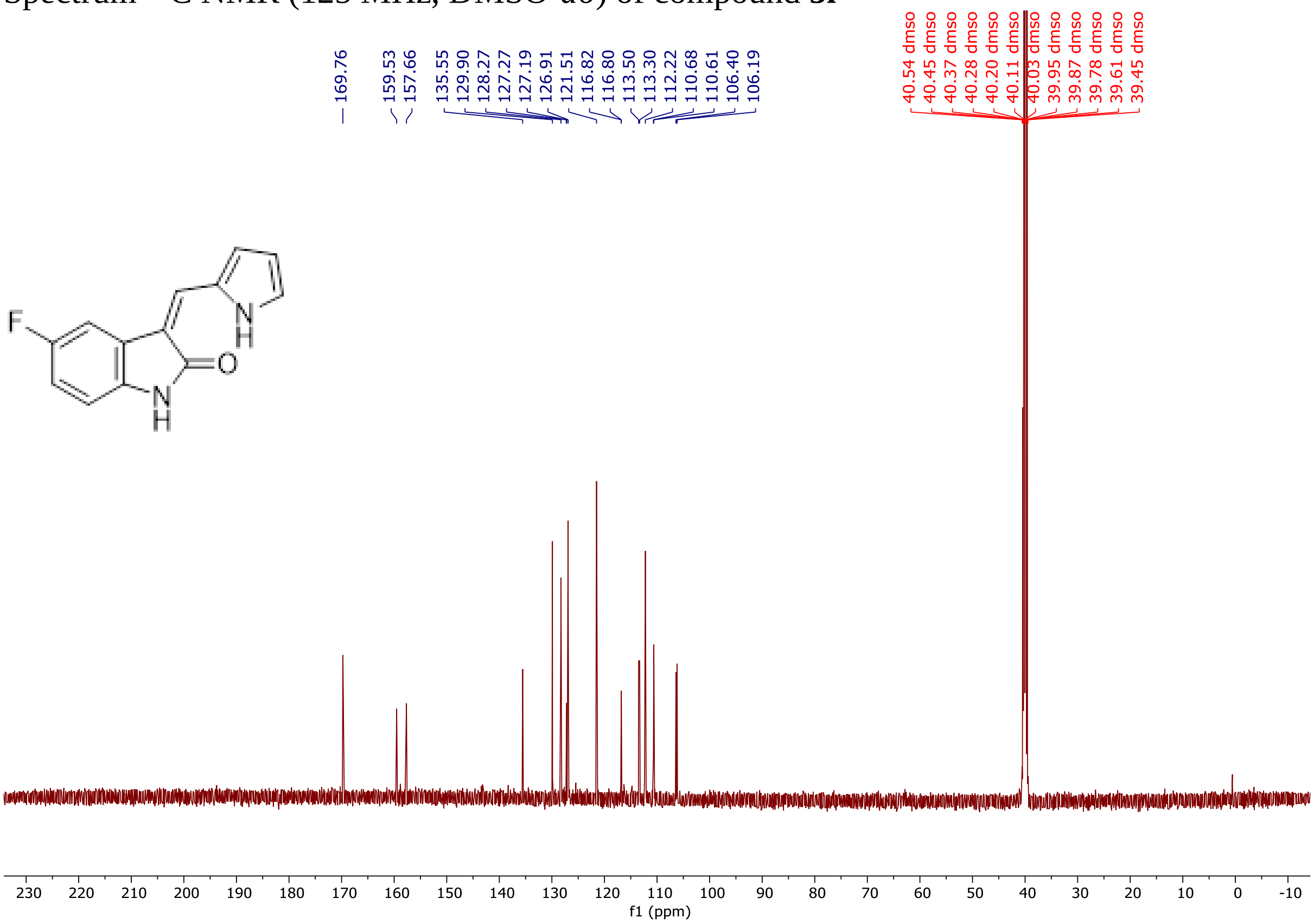

Spectrum ^{19}F NMR (470 MHz, DMSO-d_6) of compound **5l**

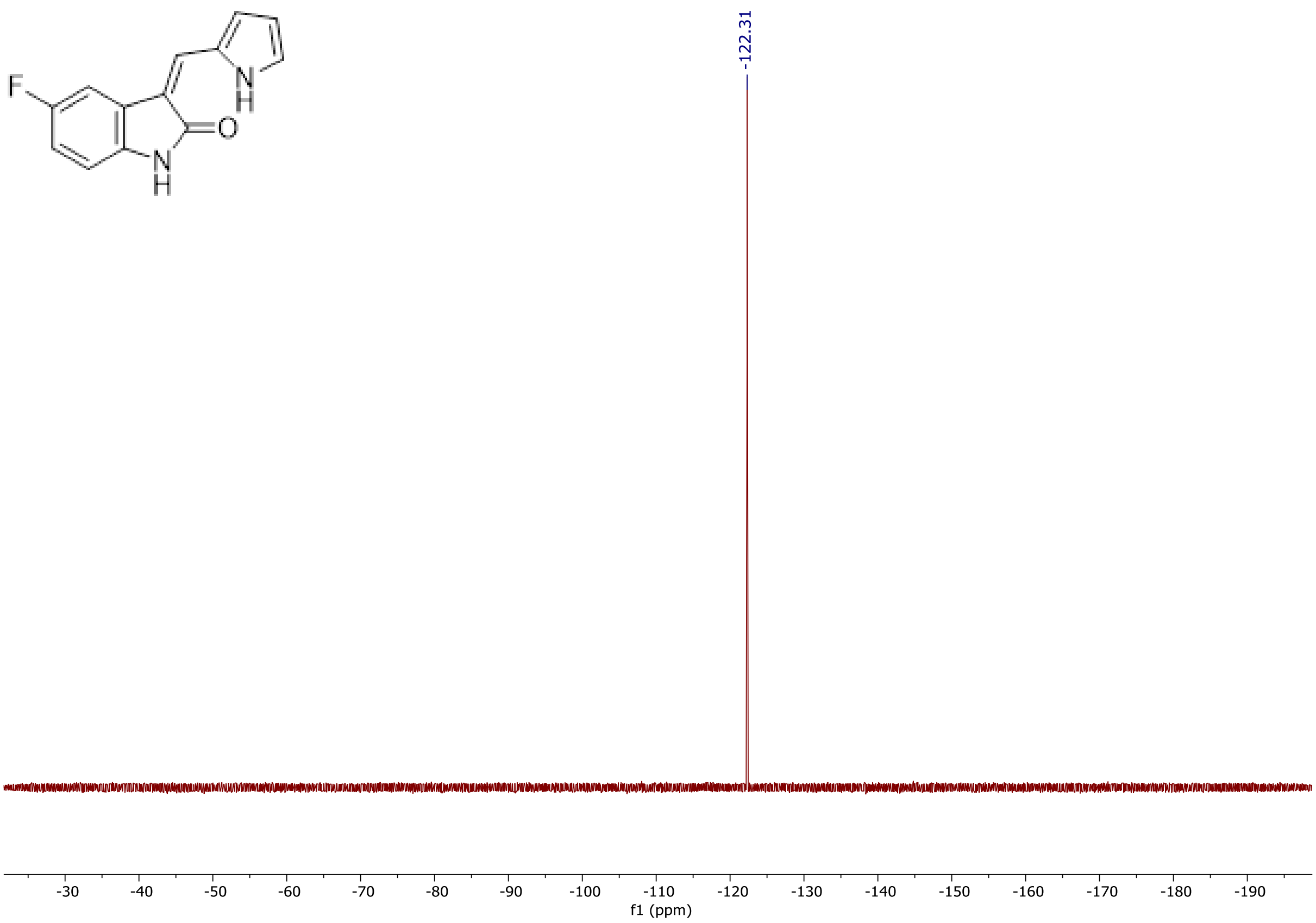

HRMS spectrum of compound **5l**

Single Mass Analysis
Tolerance = 5.0 PPM / DBE: min = -1.5, max = 50.0
Element prediction: Off
Number of isotope peaks used for i-FIT = 3

Monoisotopic Mass, Even Electron Ions
111 formula(e) evaluated with 1 results within limits (up to 50 best isotopic matches for each mass)
Elements Used:
C: 13-13 H: 0-100 N: 0-100 O: 0-100 F: 1-1

Robby_ERB18_pos 16 (0.273)
TOF MS ES+

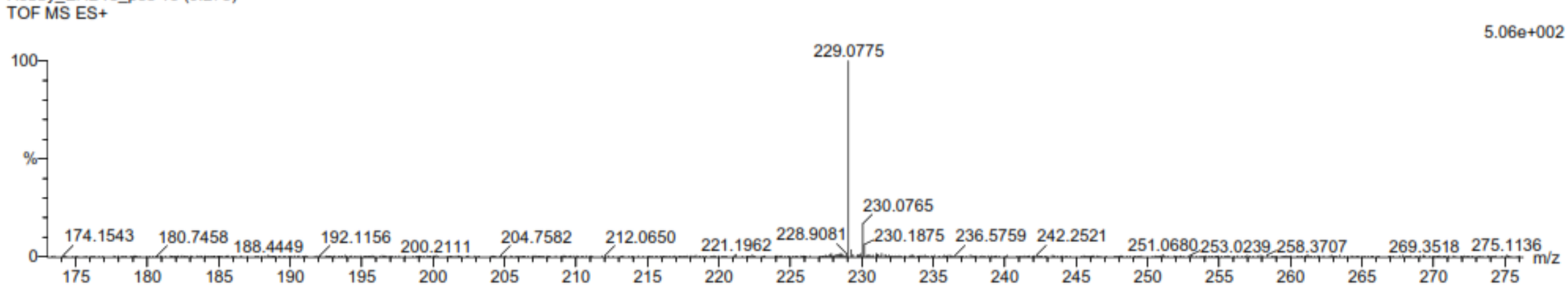

Minimum:				-1.5			
Maximum:		5.0	5.0	50.0			
Mass	Calc. Mass	mDa	PPM	DBE	i-FIT	i-FIT (Norm)	Formula
229.0775	229.0777	-0.2	-0.9	9.5	66.7	0.0	C13 H10 N2 O F

Spectrum ^{1}H NMR (500 MHz, DMSO-*d6*) of compound **5m** (*E/Z* = 100:0)

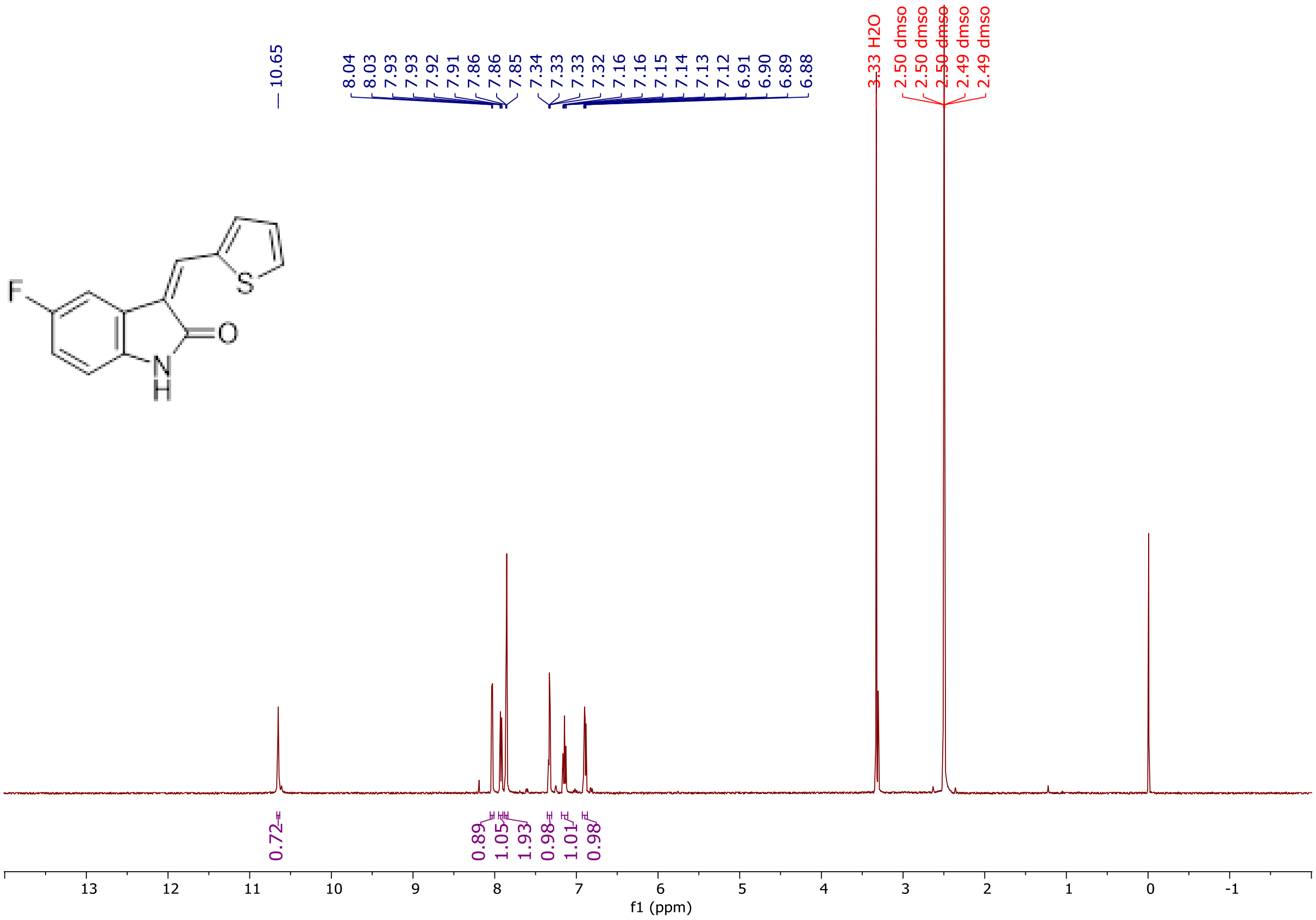

Spectrum ^{13}C NMR (125 MHz, DMSO-*d6*) of compound **5m**

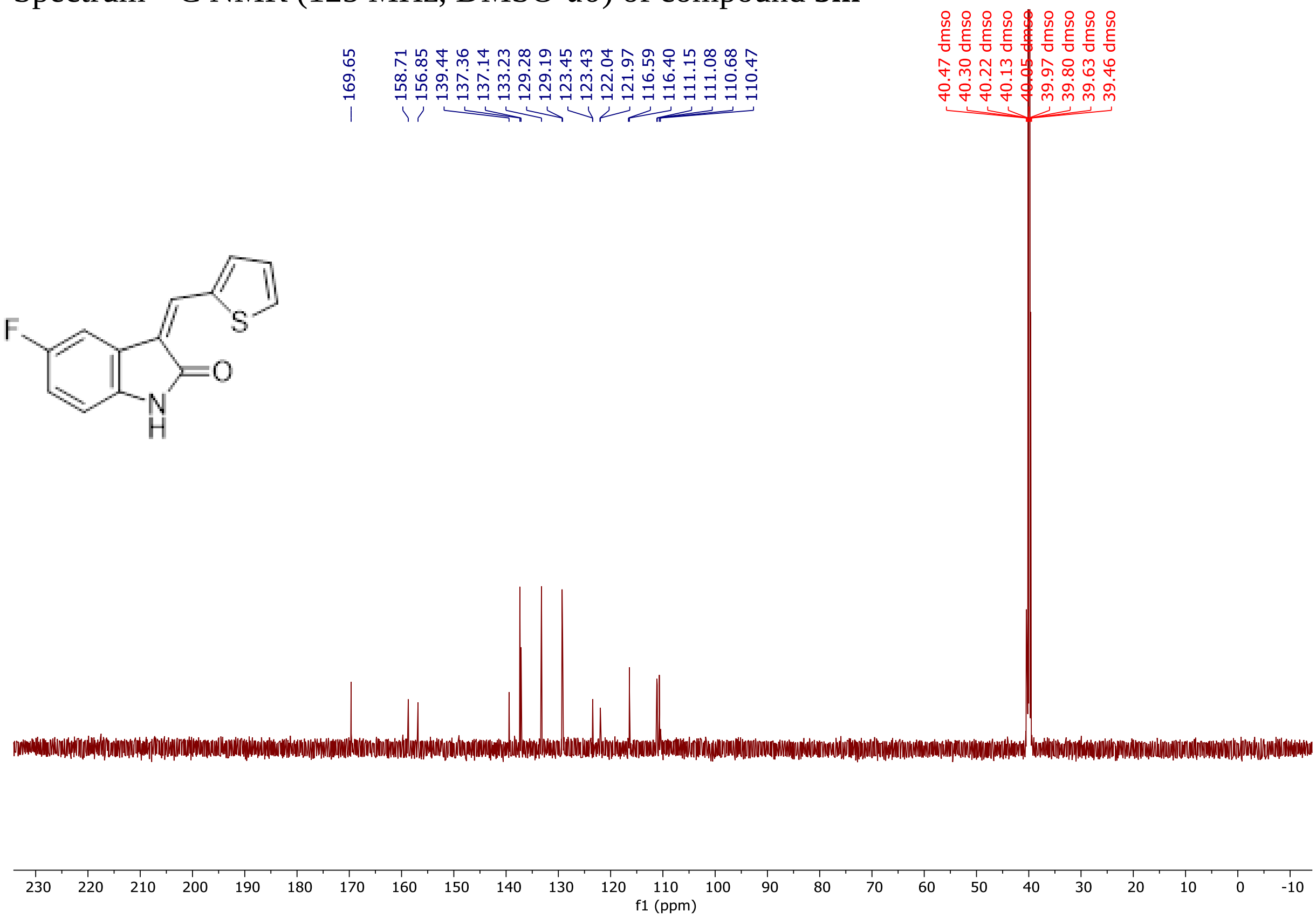

Spectrum ^{19}F NMR (470 MHz, DMSO-d_6) of compound **5m**

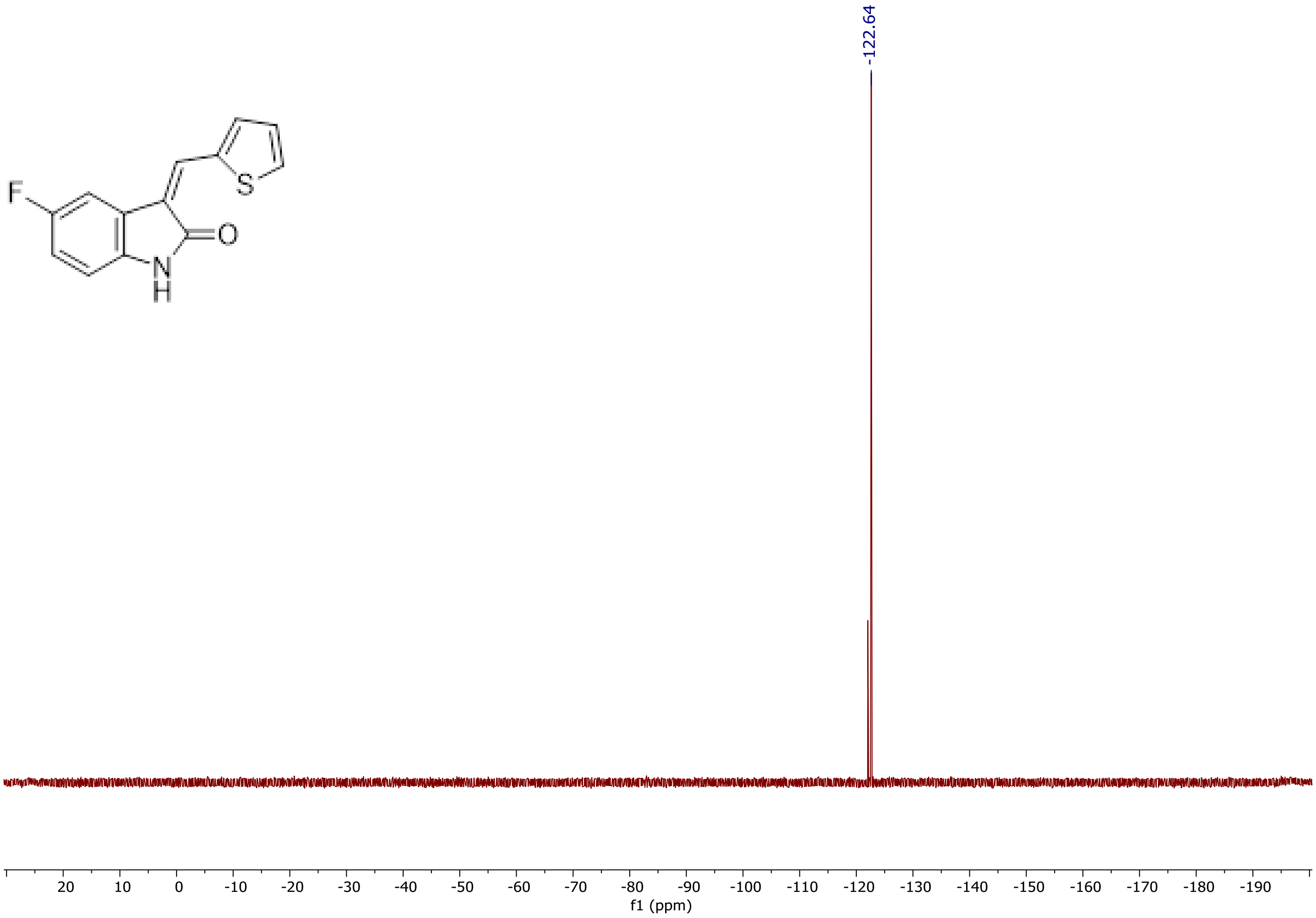

HRMS spectrum of compound **5m**

Single Mass Analysis
Tolerance = 5.0 PPM / DBE: min = -1.5, max = 50.0
Element prediction: Off
Number of isotope peaks used for i-FIT = 3

Monoisotopic Mass, Even Electron Ions
96 formula(e) evaluated with 1 results within limits (up to 50 best isotopic matches for each mass)
Elements Used:
C: 13-13 H: 0-100 N: 0-100 O: 0-100 S: 1-1 F: 1-1

Robby_ERB19_pos 22 (0.358)
TOF MS ES+

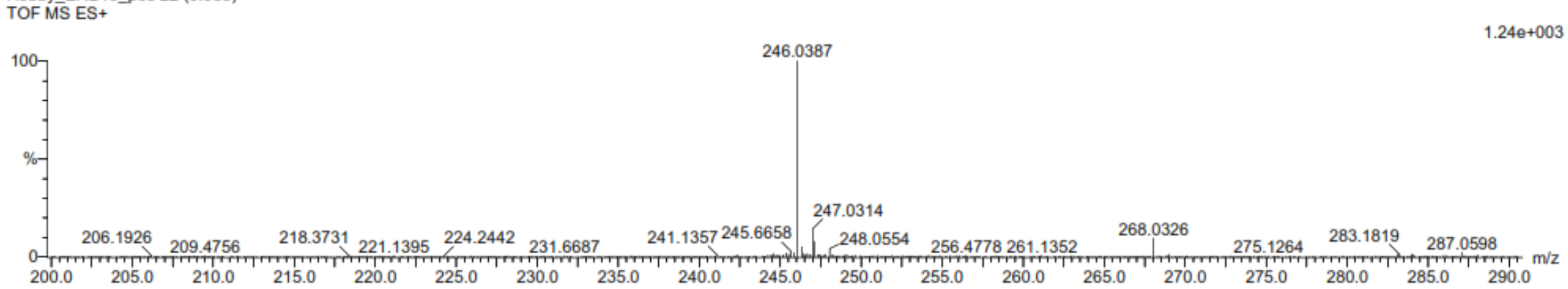

Minimum:				-1.5			
Maximum:		5.0	5.0	50.0			
Mass	Calc. Mass	mDa	PPM	DBE	i-FIT	i-FIT (Norm)	Formula
246.0387	246.0389	-0.2	-0.8	9.5	105.2	0.0	C13 H9 N O S F

Spectrum ^{1}H NMR (500 MHz, DMSO-*d6*) of compound **5n** (*E/Z* = 97:3)

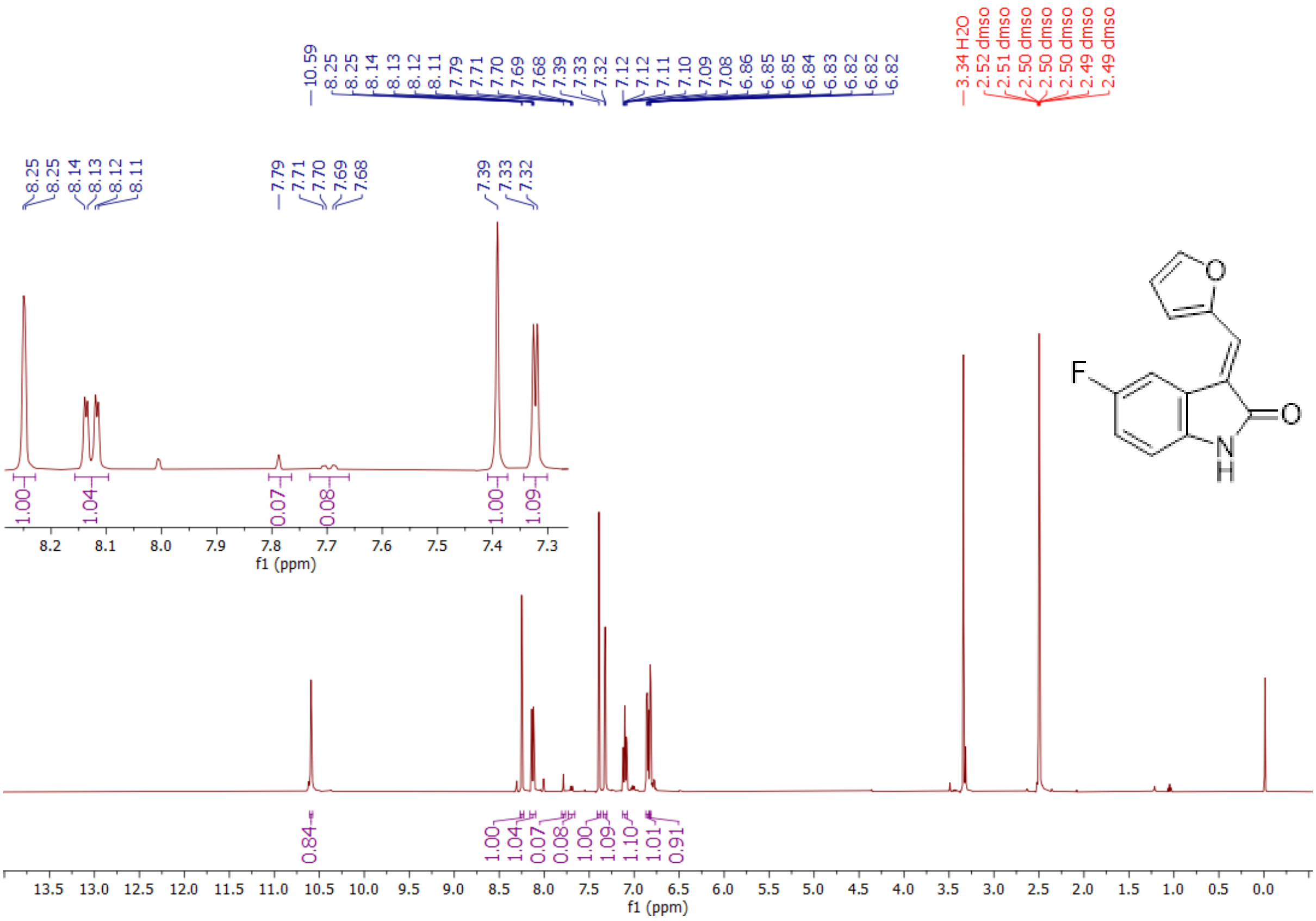

Spectrum ^{13}C NMR (125 MHz, DMSO-*d6*) of compound **5n**

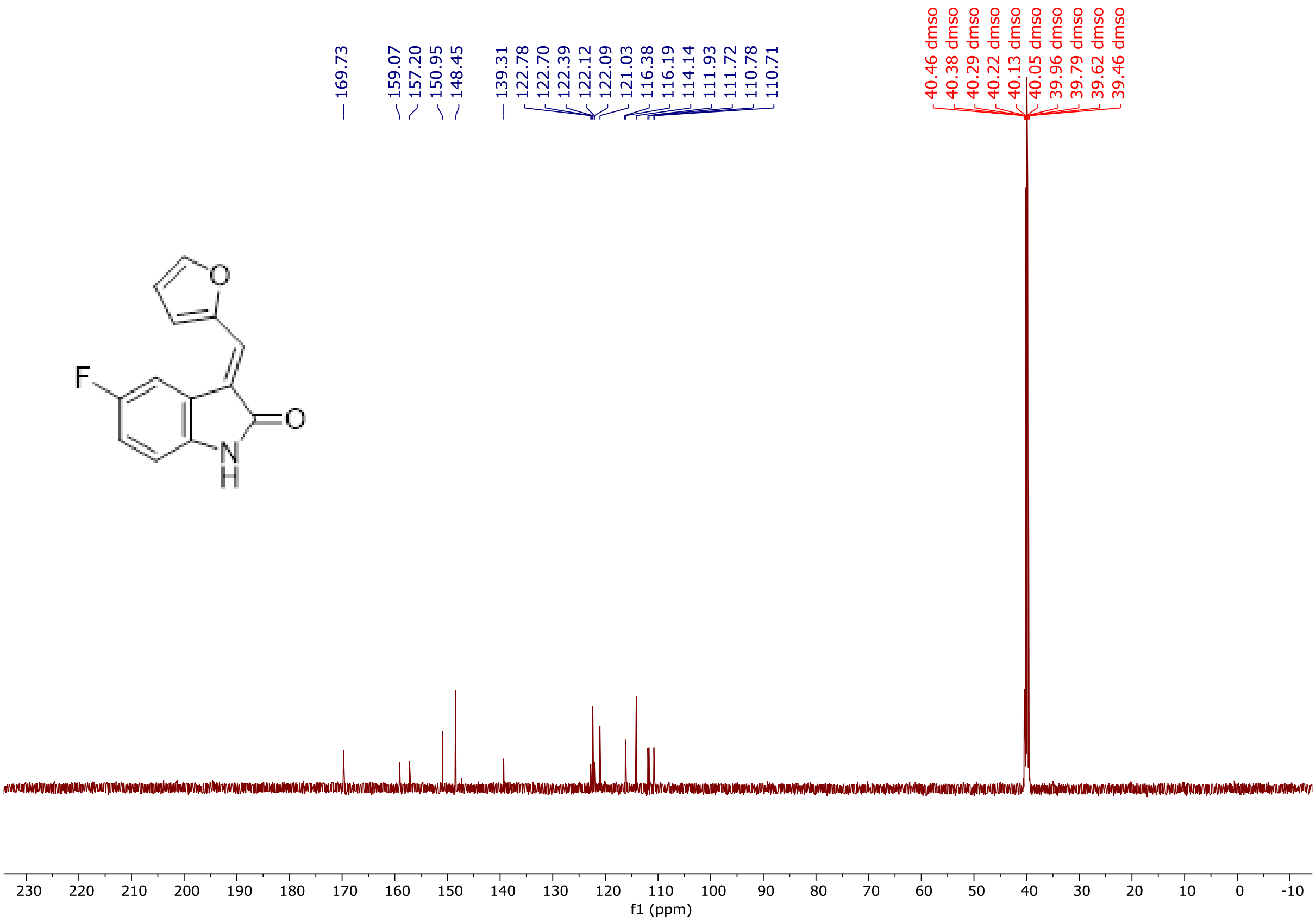

Spectrum ^{19}F NMR (470 MHz, DMSO-d_6) of compound **5n**

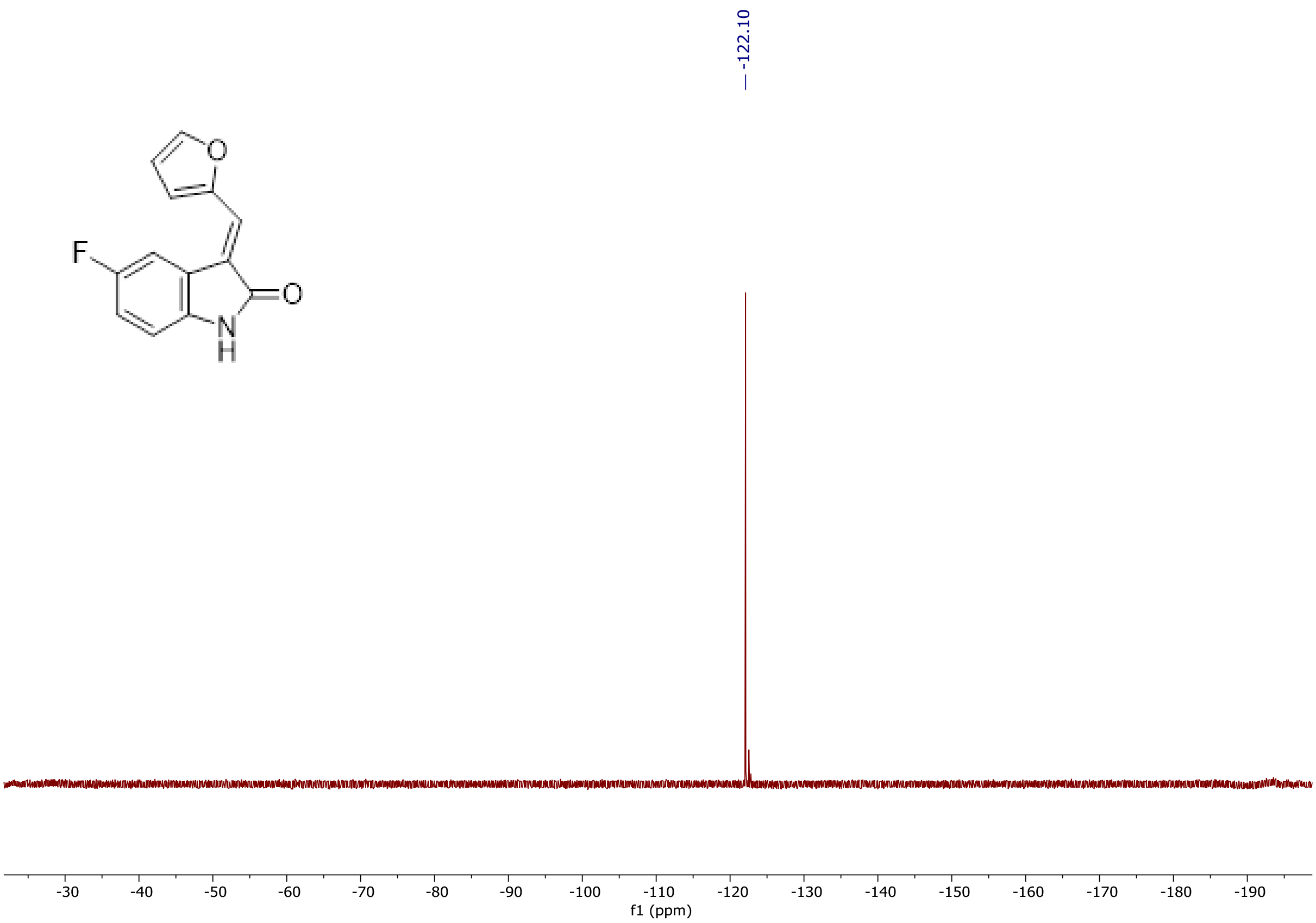

HRMS spectrum of compound **5n**

Monoisotopic Mass, Even Electron Ions
112 formula(e) evaluated with 1 results within limits (up to 50 best isotopic matches for each mass)
Elements Used:
C: 13-13 H: 0-100 N: 0-100 O: 0-100 F: 1-1

Robby_ERB21_pos 77 (1.297)
TOF MS ES+

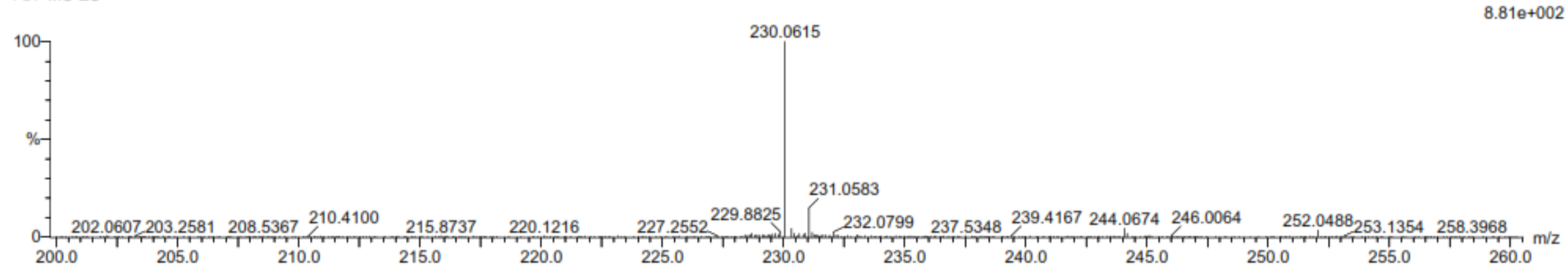

Minimum:				-1.5			
Maximum:		5.0	5.0	50.0			
Mass	Calc. Mass	mDa	PPM	DBE	i-FIT	i-FIT (Norm)	Formula
230.0615	230.0617	-0.2	-0.9	9.5	97.2	0.0	C13 H9 N O2 F

Spectrum ^{1}H NMR (500 MHz, DMSO-*d6*) of compound **5o** (*E/Z* = 97:3)

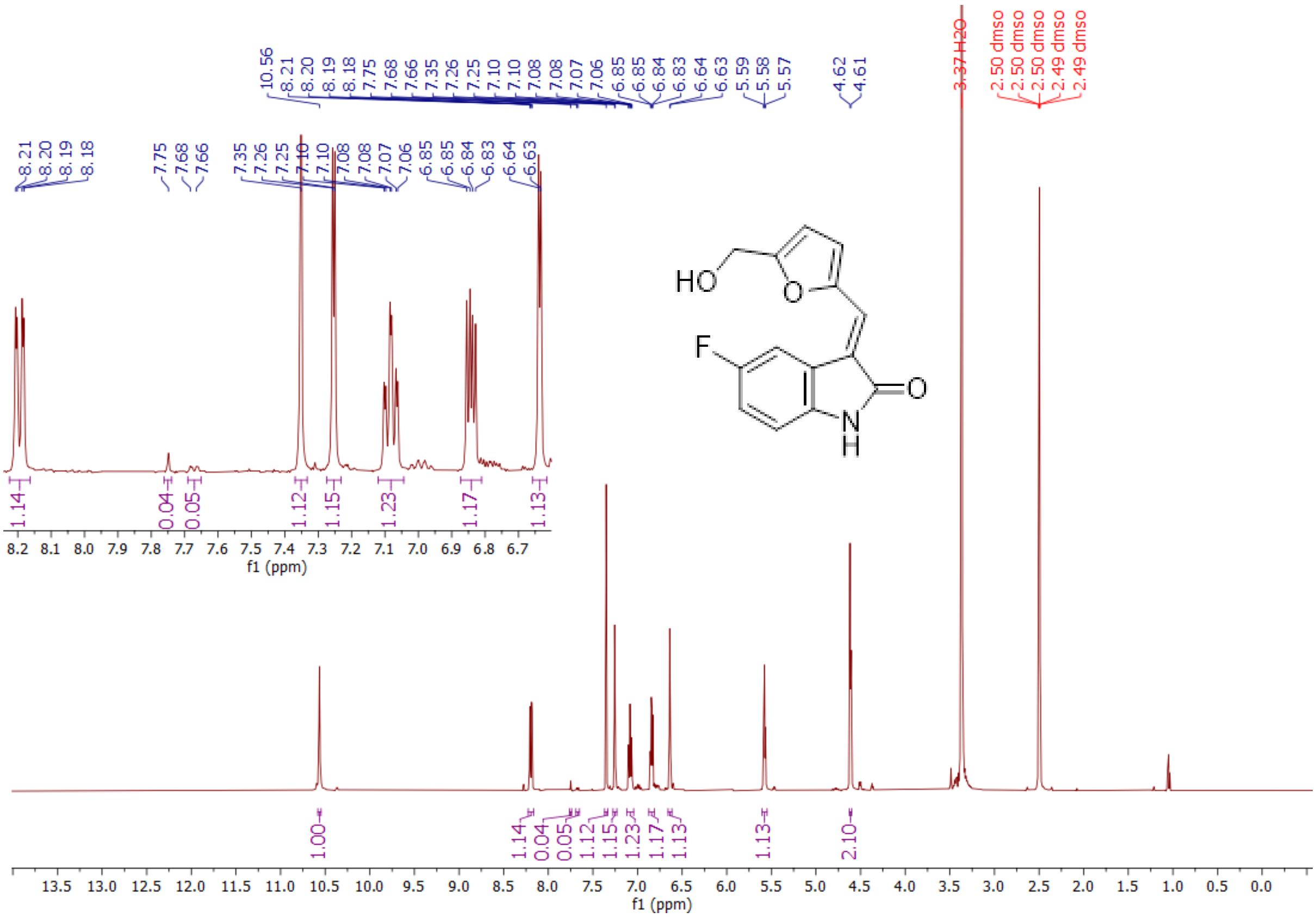

Spectrum ^{13}C NMR (125 MHz, DMSO-*d6*) of compound **5o**

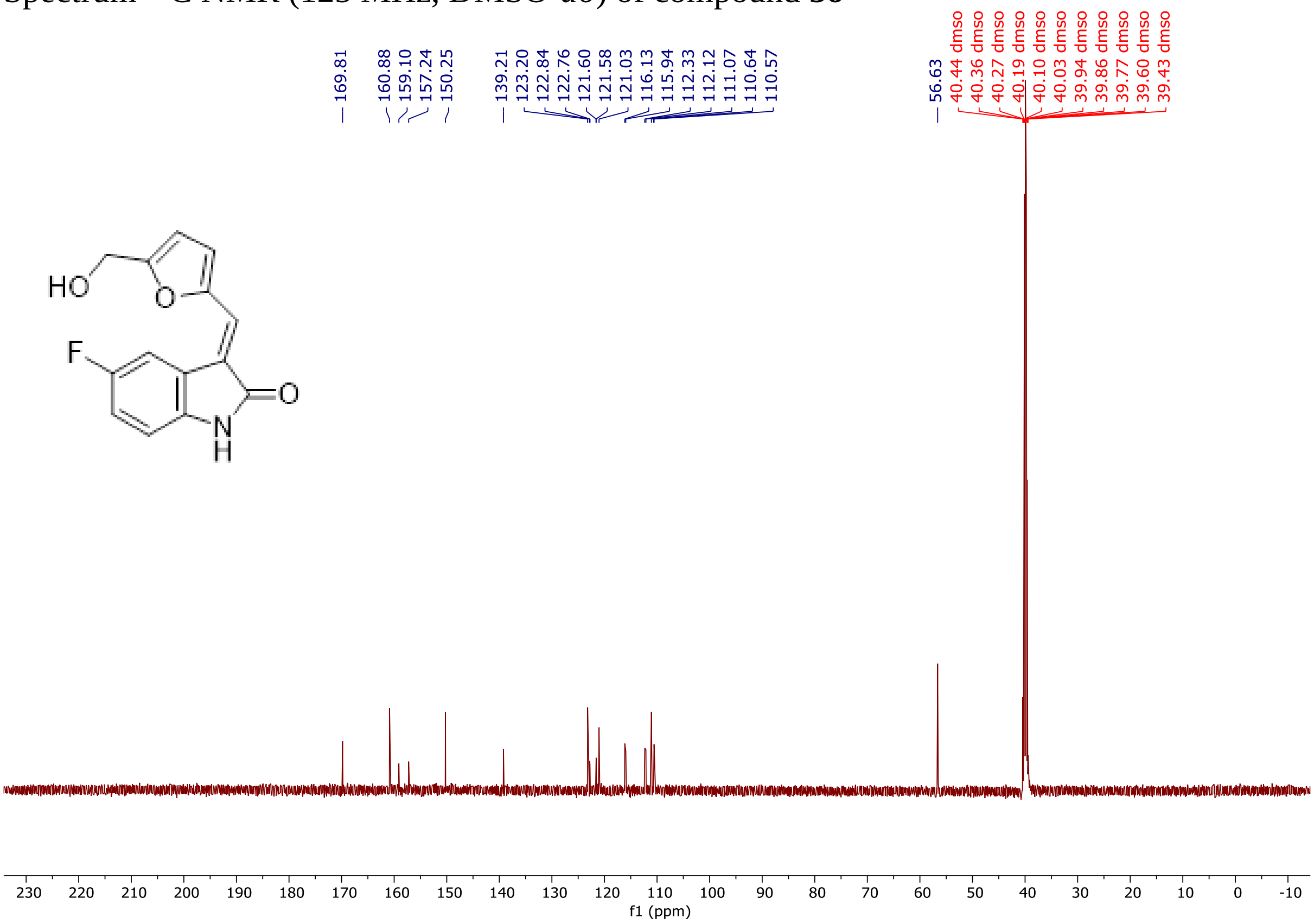

Spectrum ^{19}F NMR (470 MHz, DMSO-d_6) of compound **5o**

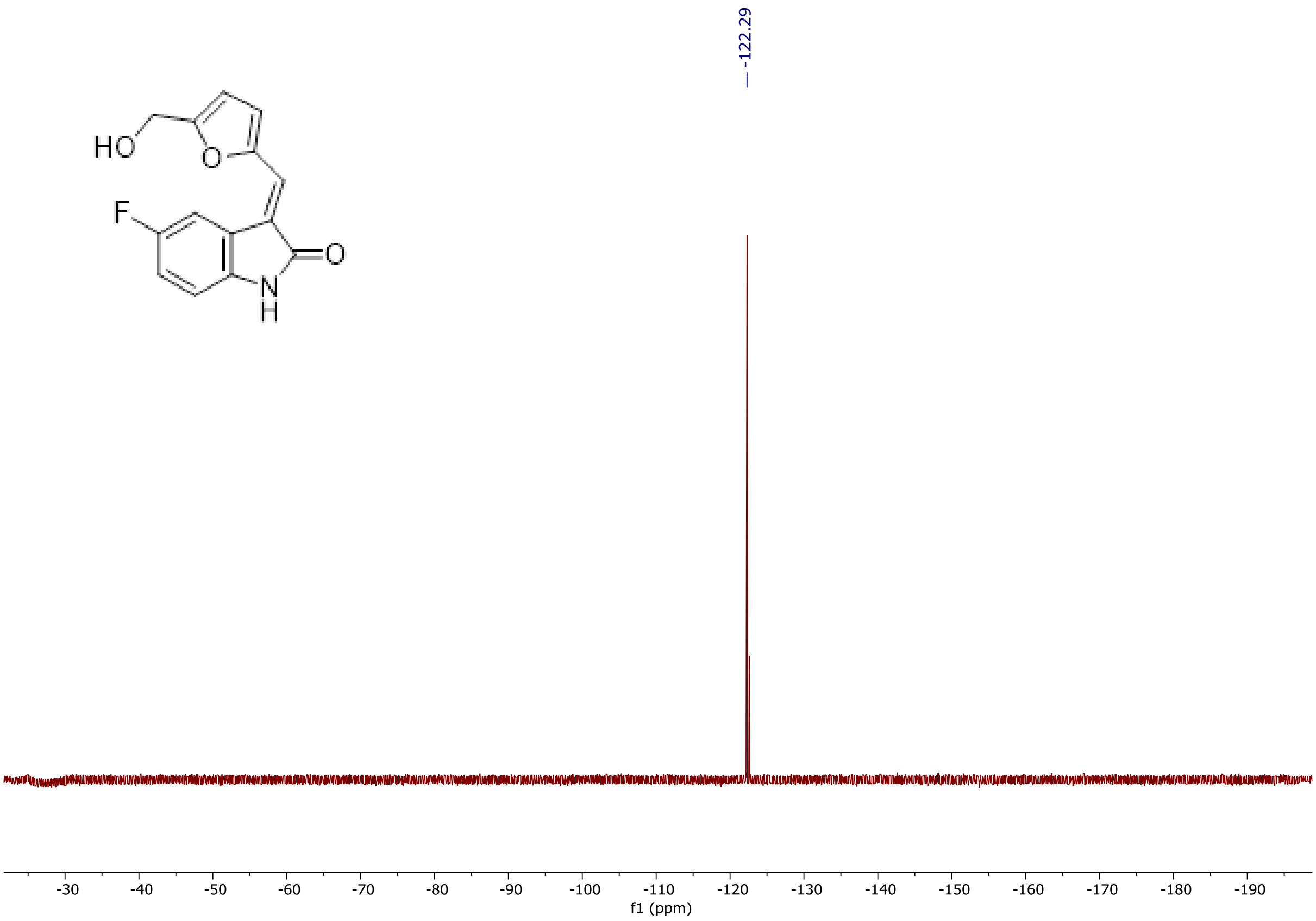

HRMS spectrum of compound **5o**

Single Mass Analysis
Tolerance = 5.0 PPM / DBE: min = -1.5, max = 50.0
Element prediction: Off
Number of isotope peaks used for i-FIT = 3

Monoisotopic Mass, Even Electron Ions
145 formula(e) evaluated with 1 results within limits (up to 50 best isotopic matches for each mass)
Elements Used:
C: 14-14 H: 0-100 N: 0-100 O: 0-100 F: 1-1

Robby_ERB16_pos 13 (0.222)
TOF MS ES+

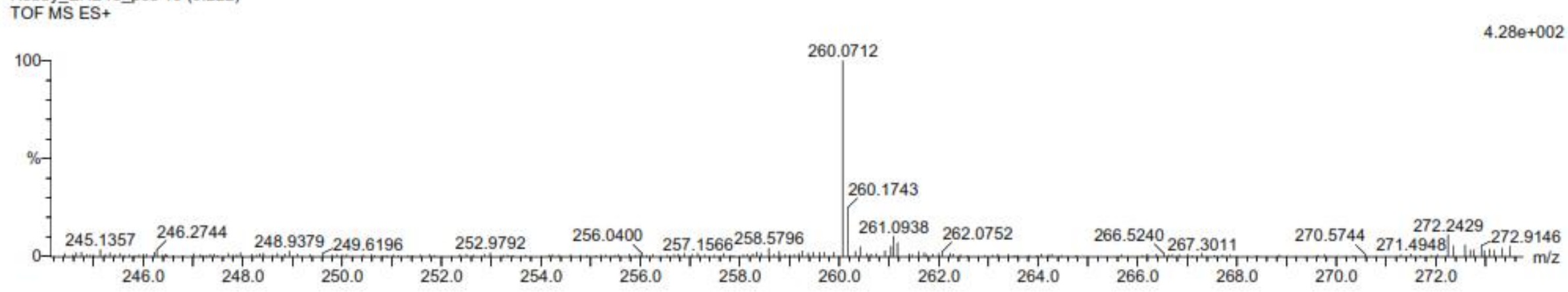

Minimum:				-1.5			
Maximum:		5.0	5.0	50.0			
Mass	Calc. Mass	mDa	PPM	DBE	i-FIT	i-FIT (Norm)	Formula
260.0712	260.0723	-1.1	-4.2	9.5	98.0	0.0	C14 H11 N O3 F

Spectrum ^{1}H NMR (500 MHz, DMSO-*d6*) of compound **8a**

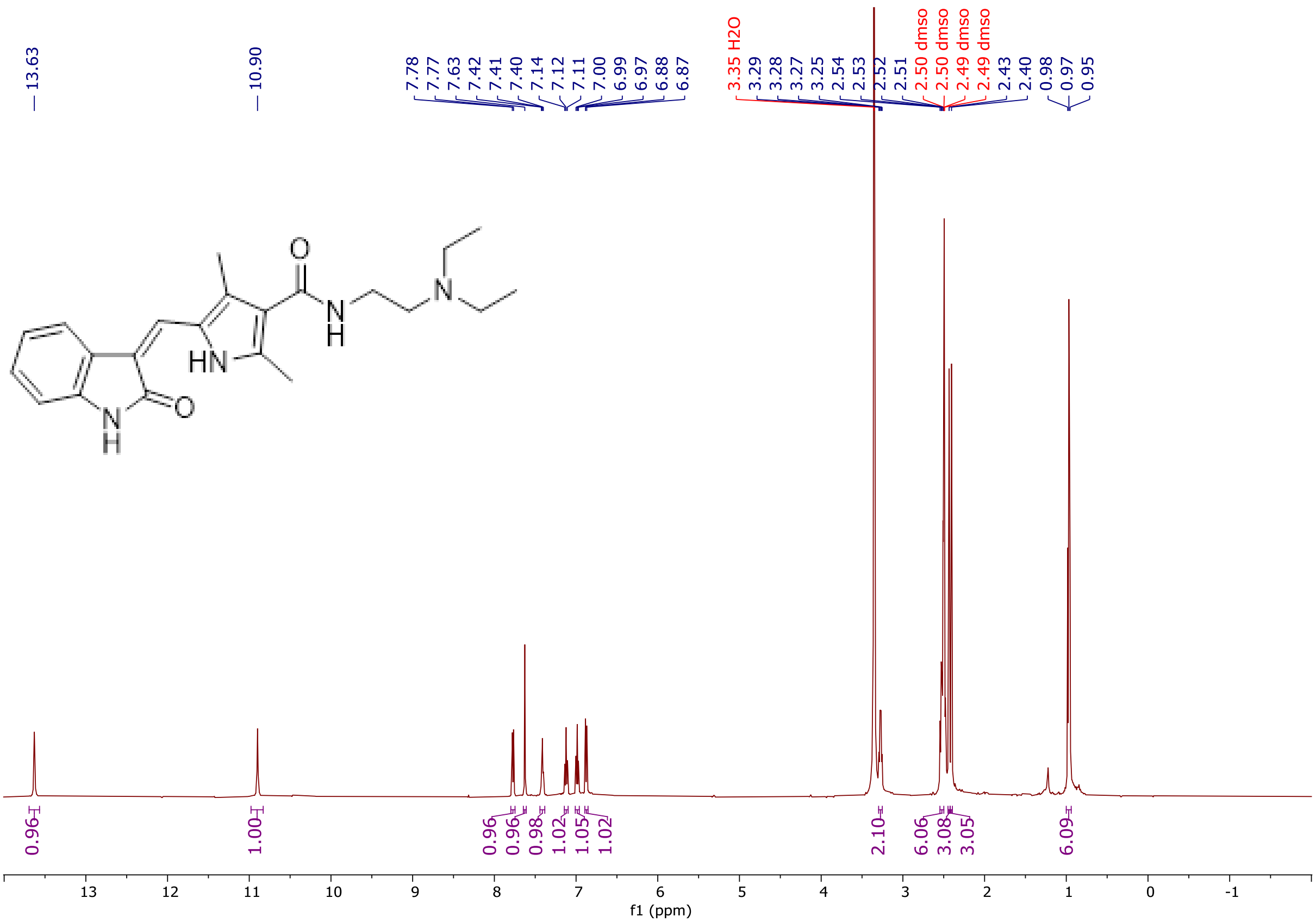

Spectrum ^{13}C NMR (125 MHz, DMSO-*d6*) of compound **8a**

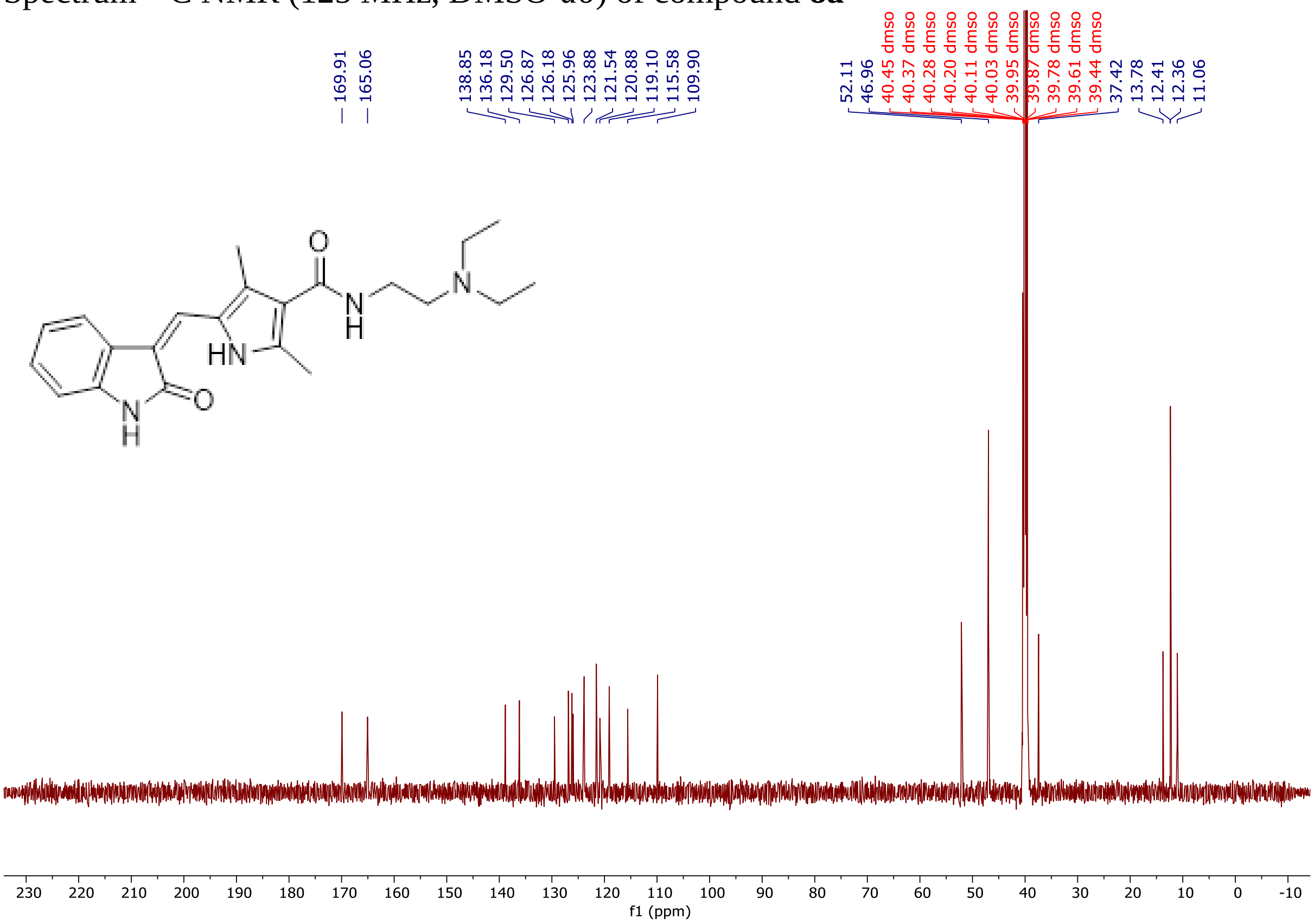

HRMS spectrum of compound **8a**

Single Mass Analysis
Tolerance = 5.0 PPM / DBE: min = -1.5, max = 50.0
Element prediction: Off
Number of isotope peaks used for i-FIT = 3

Monoisotopic Mass, Even Electron Ions
348 formula(e) evaluated with 1 results within limits (up to 50 best isotopic matches for each mass)
Elements Used:
C: 22-22 H: 0-100 N: 0-100 O: 0-100
Robby_ERIS_pos 8 (0.120) Cm (6:10)
TOF MS ES+

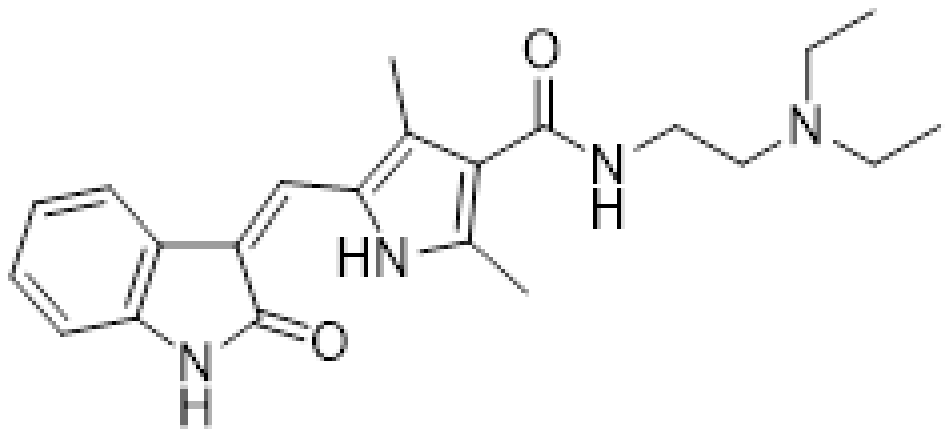

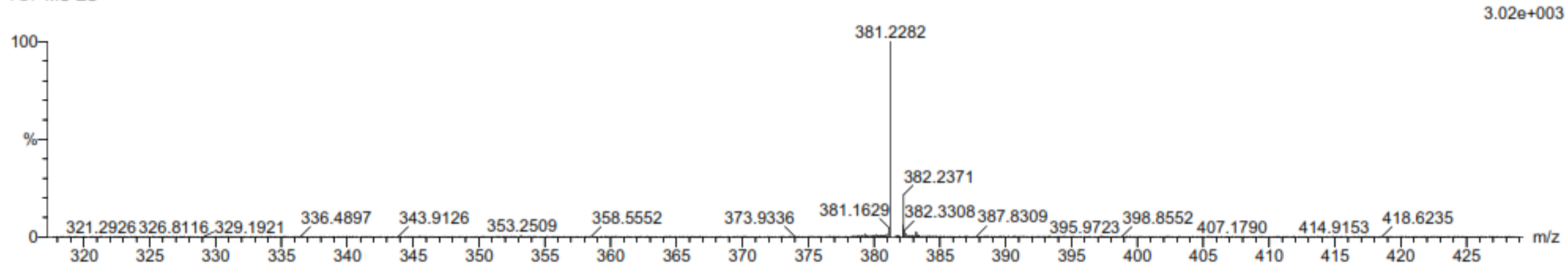

Minimum:				-1.5			
Maximum:		5.0	5.0	50.0			
Mass	Calc. Mass	mDa	PPM	DBE	i-FIT	i-FIT (Norm)	Formula
381.2282	381.2291	-0.9	-2.4	10.5	175.1	0.0	C22 H29 N4 O2

Spectrum ^{1}H NMR (500 MHz, DMSO-*d6*) of compound **8b**

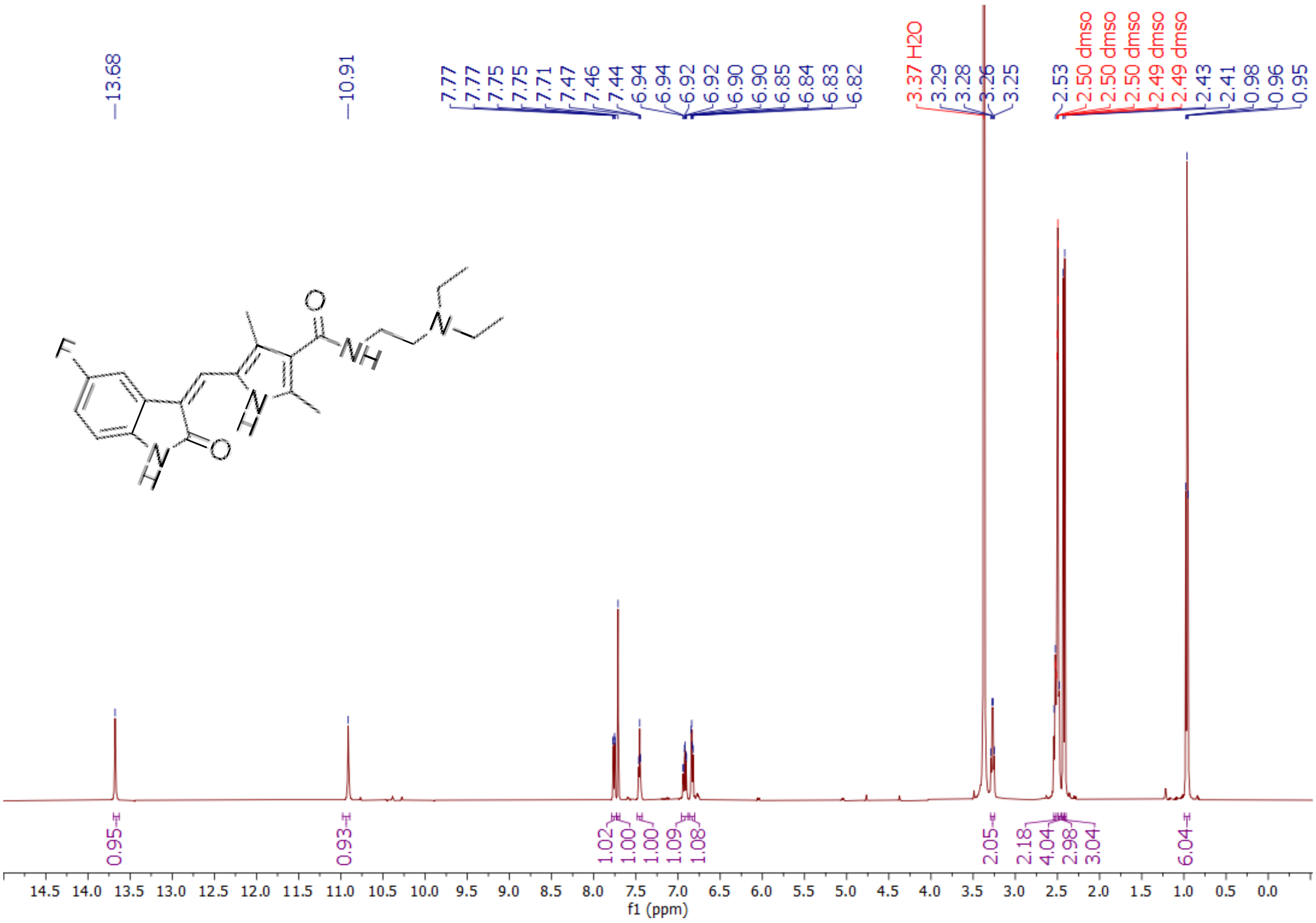

Spectrum ^{13}C NMR (125 MHz, DMSO-*d6*) of compound **8b**

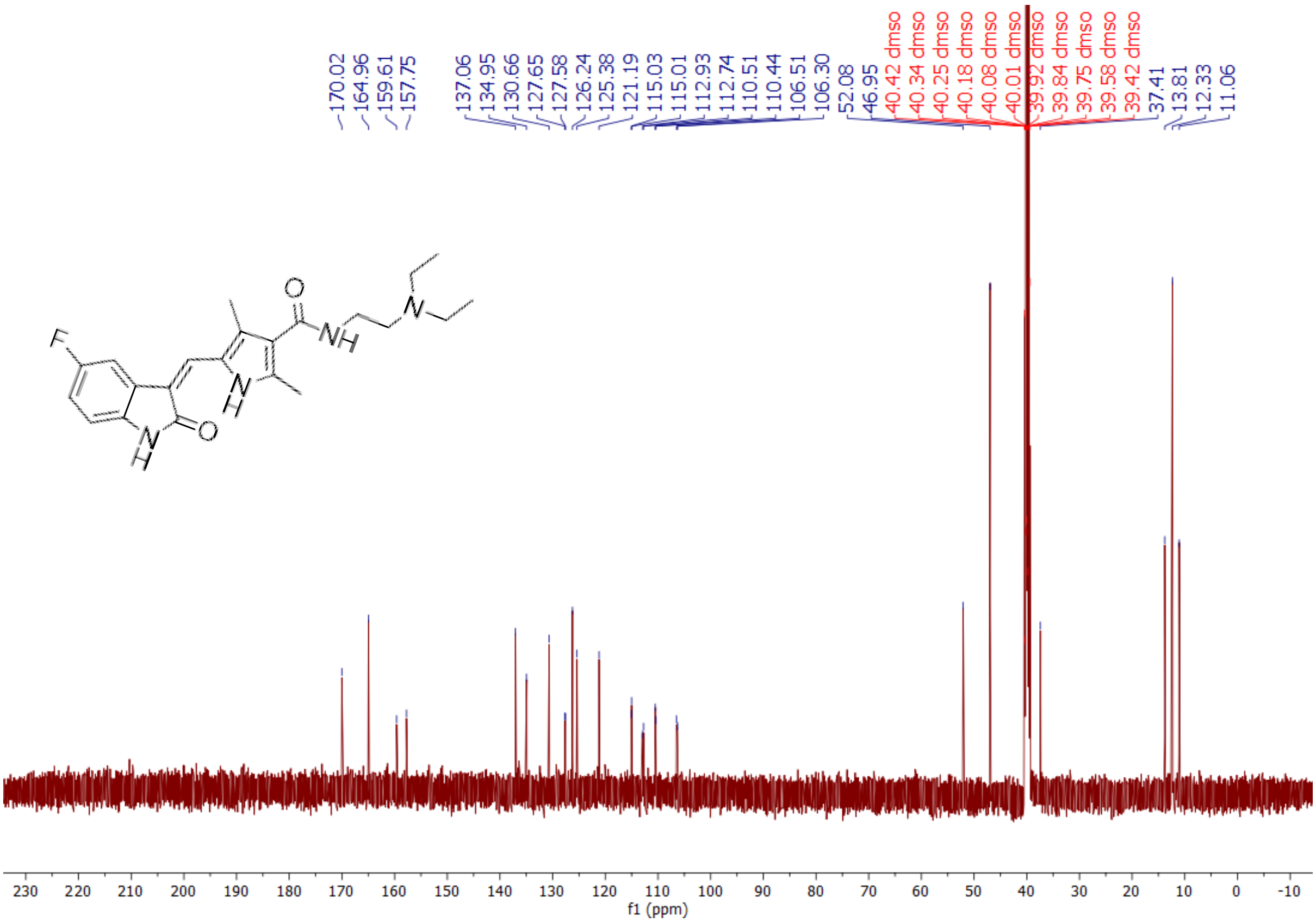

Spectrum ^{19}F NMR (470 MHz, DMSO-d_6) of compound **8b**

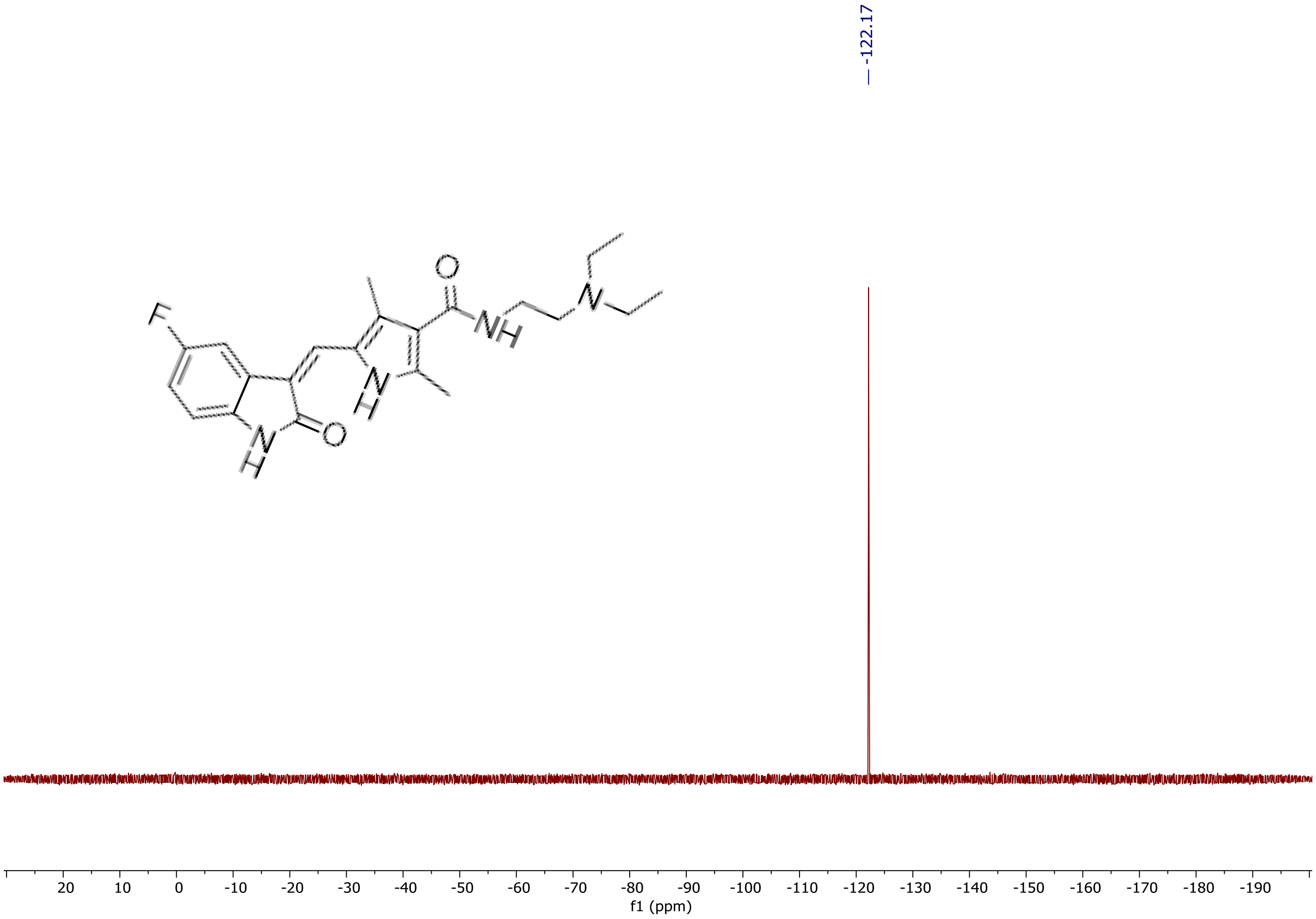

HRMS spectrum of compound **8b**

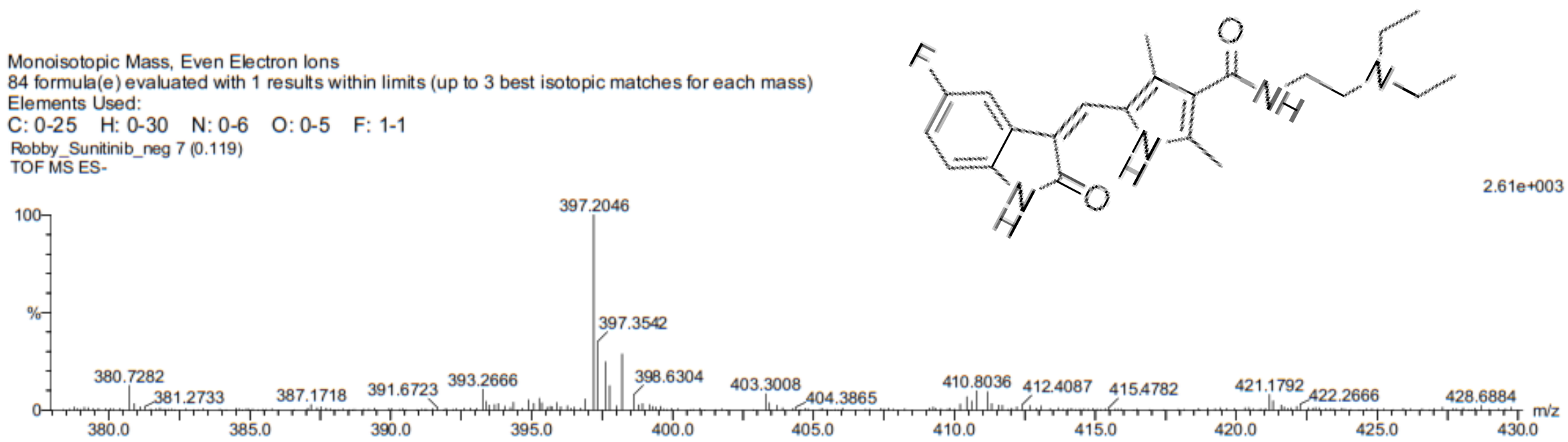

Spectrum ^{1}H NMR (500 MHz, DMSO-*d6*) of compound **8c**

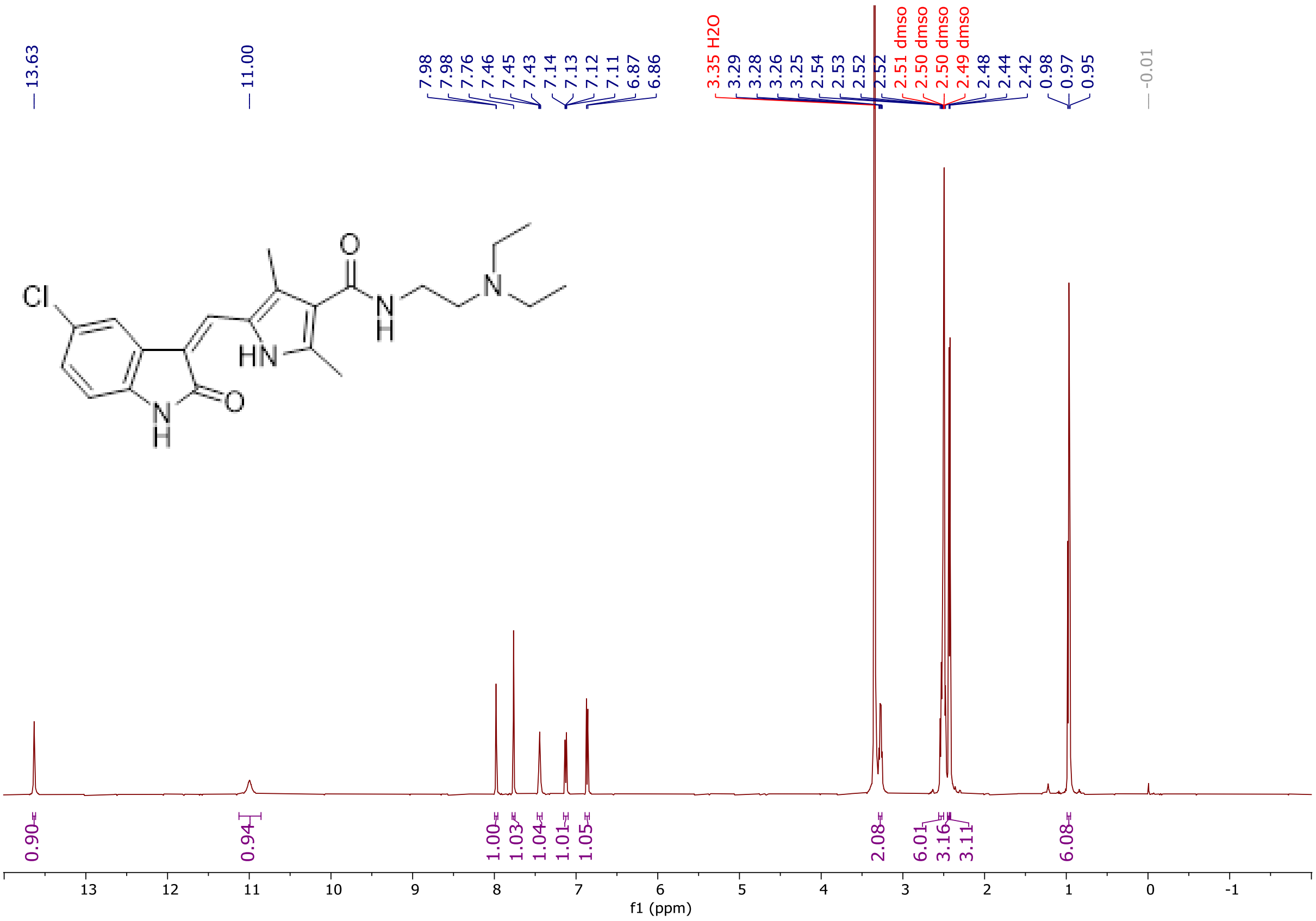

Spectrum ^{13}C NMR (125 MHz, DMSO-*d6*) of compound **8c**

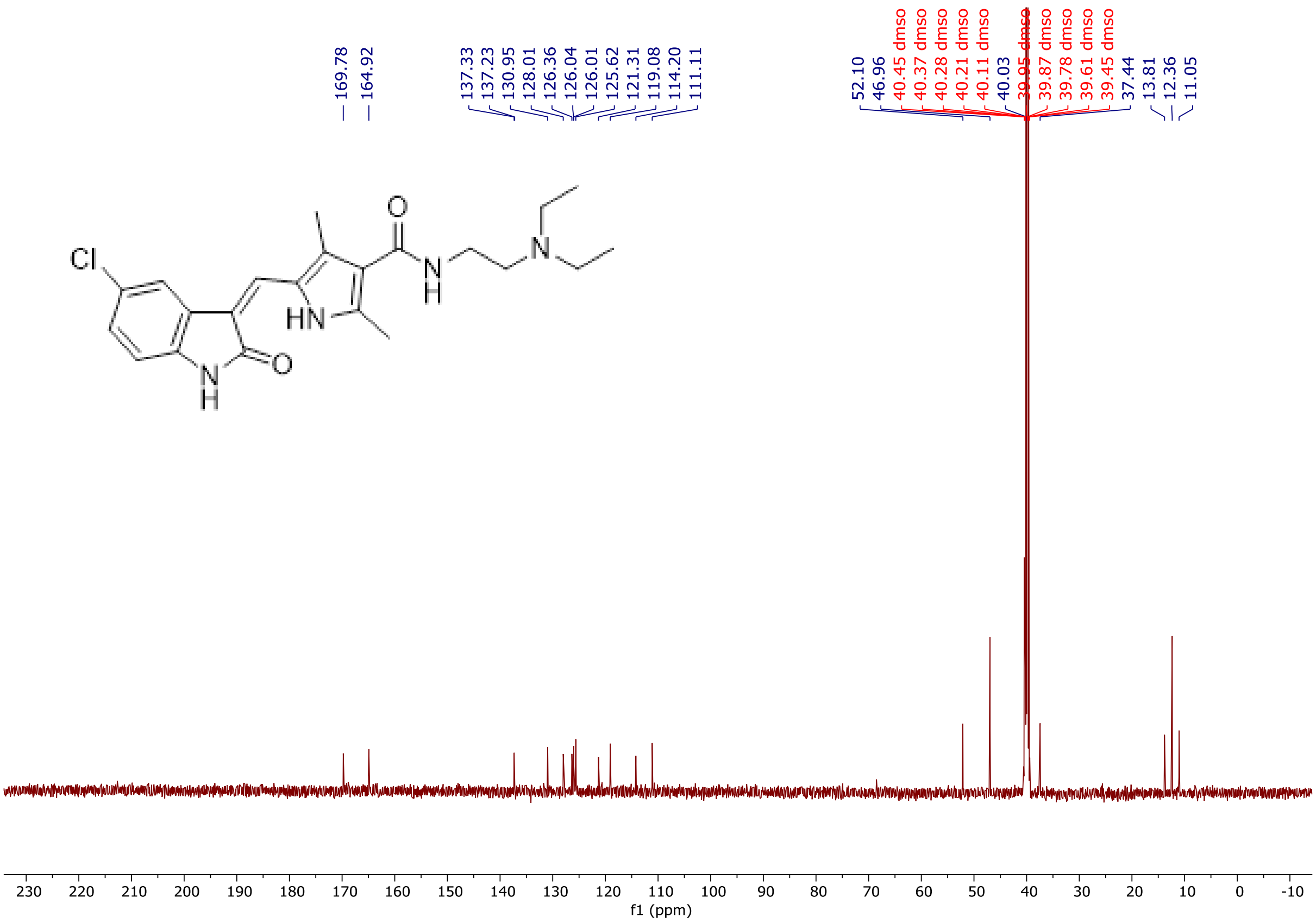

HRMS spectrum of compound **8c**

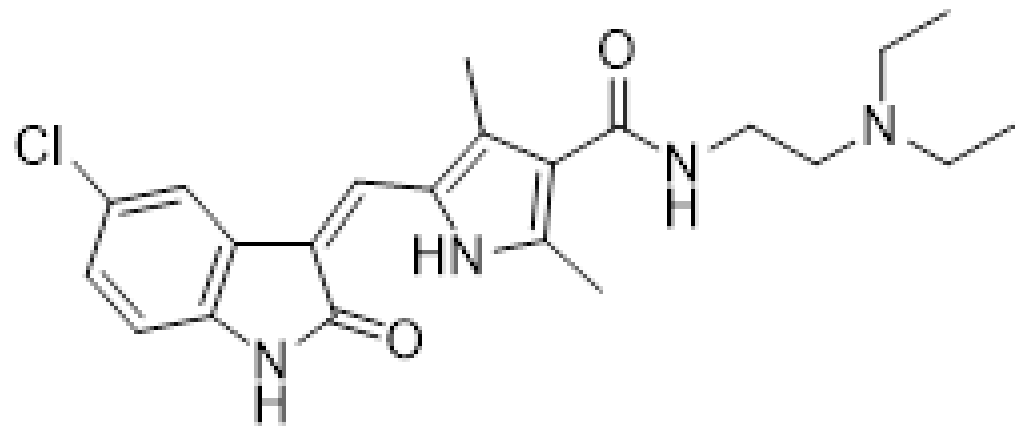

Single Mass Analysis
Tolerance = 5.0 PPM / DBE: min = -1.5, max = 50.0
Element prediction: Off
Number of isotope peaks used for i-FIT = 3

Monoisotopic Mass, Even Electron Ions
347 formula(e) evaluated with 1 results within limits (up to 50 best isotopic matches for each mass)
Elements Used:
C: 22-22 H: 0-100 N: 0-100 O: 0-100 Cl: 1-1
Robby_RGC1_pos 89 (1.501)
TOF MS ES+

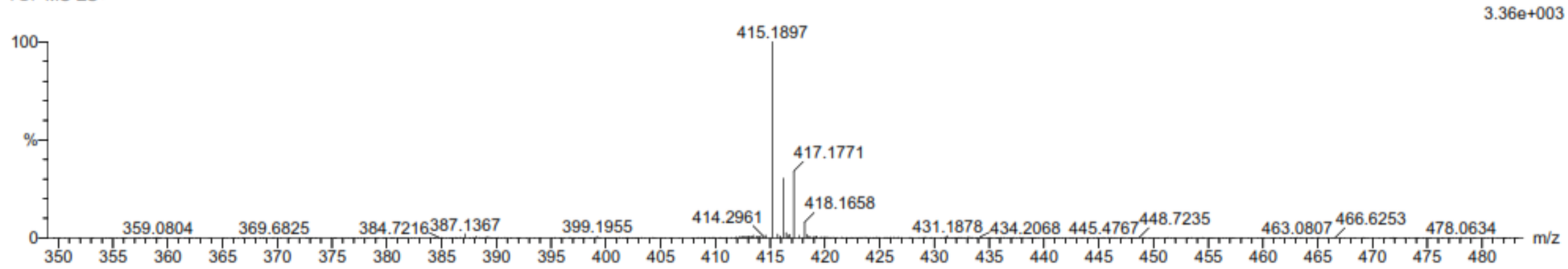

Minimum:				-1.5			
Maximum:		5.0	5.0	50.0			
Mass	Calc. Mass	mDa	PPM	DBE	i-FIT	i-FIT (Norm)	Formula
415.1897	415.1901	-0.4	-1.0	10.5	64.6	0.0	C22 H28 N4 O2 Cl

Spectrum ^{1}H NMR (500 MHz, DMSO-*d6*) of compound **8d**

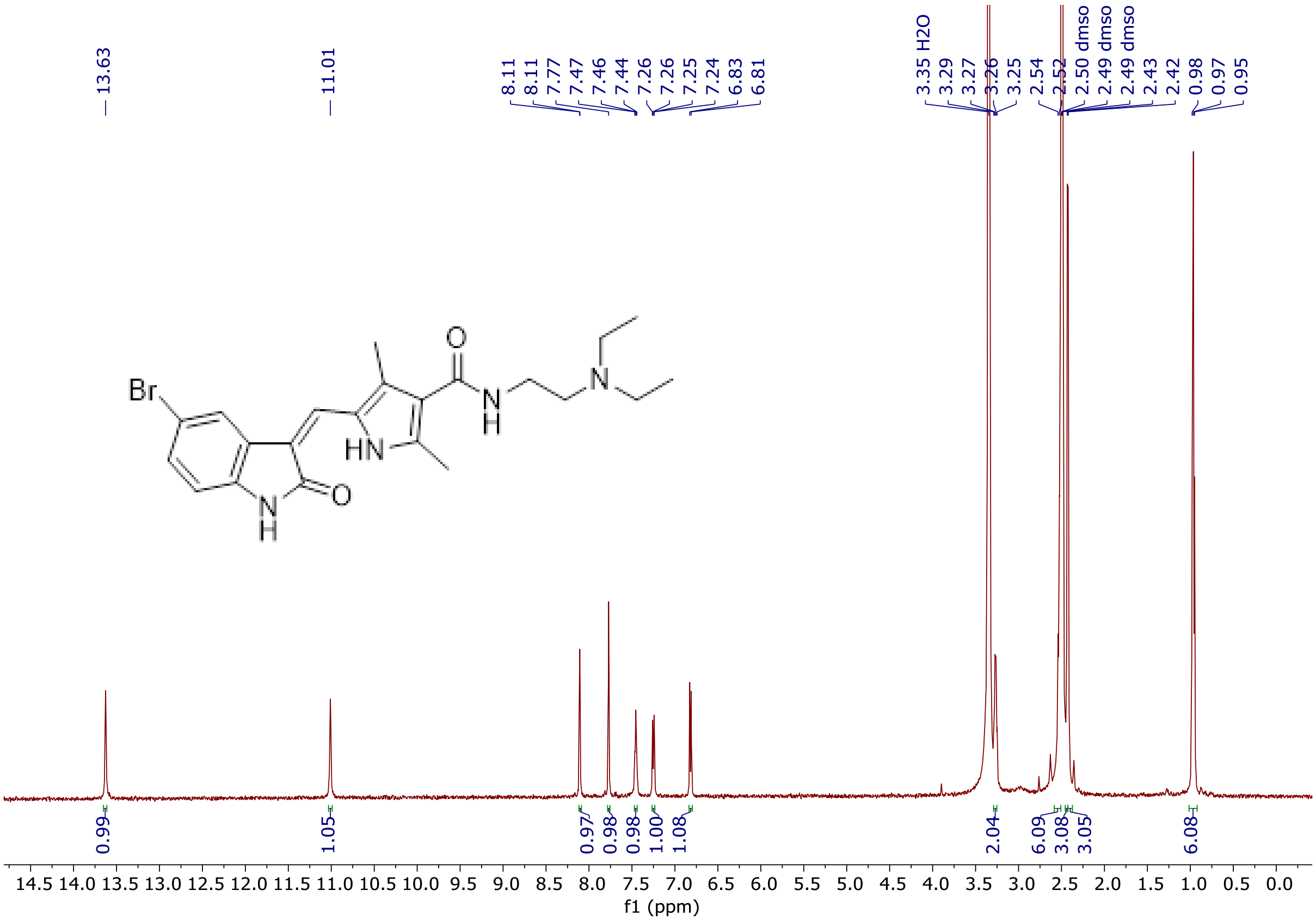

Spectrum ^{13}C NMR (125 MHz, DMSO-*d6*) of compound **8d**

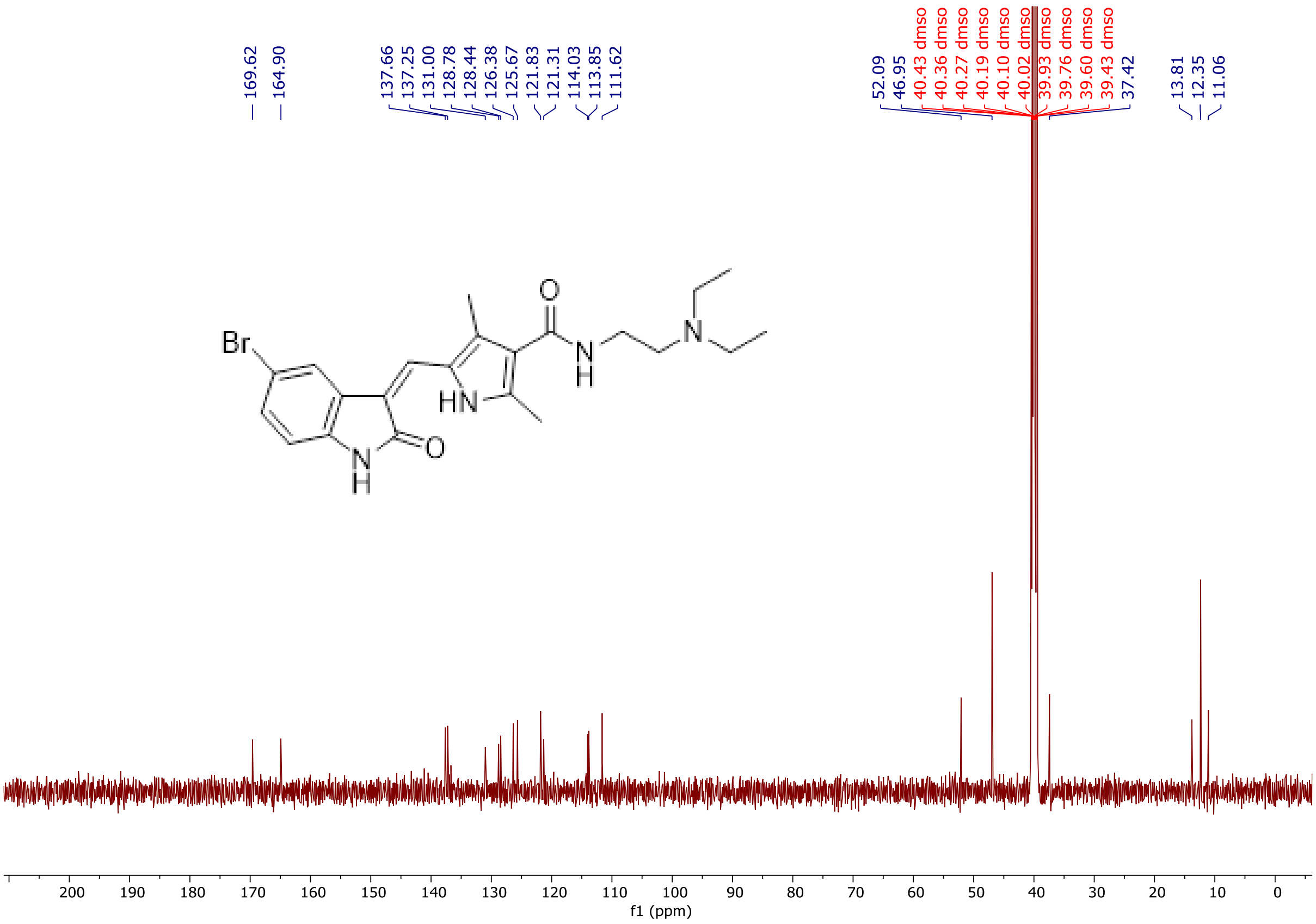

HRMS spectrum of compound **8d**

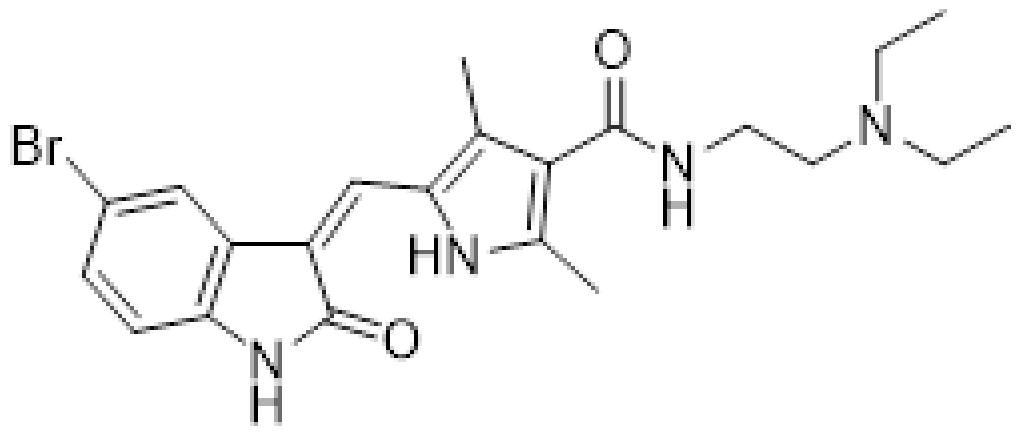

Single Mass Analysis
Tolerance = 5.0 PPM / DBE: min = -1.5, max = 50.0
Element prediction: Off
Number of isotope peaks used for i-FIT = 3

Monoisotopic Mass, Even Electron Ions
347 formula(e) evaluated with 1 results within limits (up to 50 best isotopic matches for each mass)
Elements Used:
C: 22-22 H: 0-100 N: 0-100 O: 0-100 Br: 1-1
Robby_RGB1_pos1 52 (0.870)
TOF MS ES+

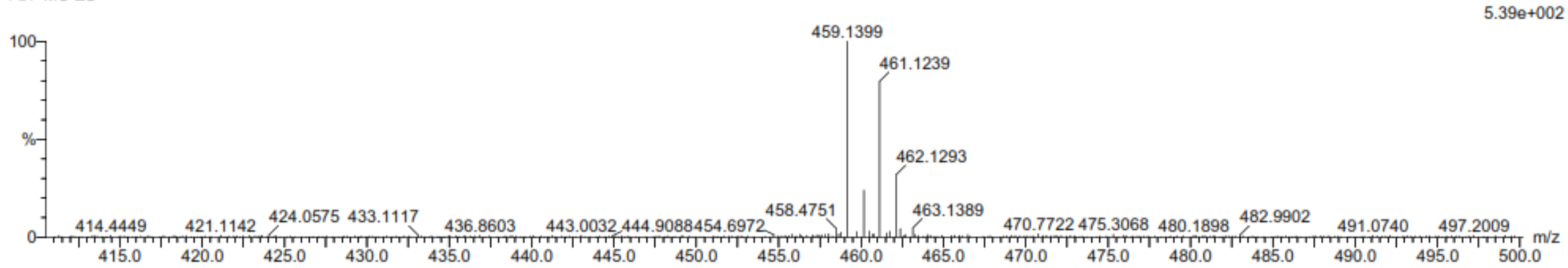

		mDa	PPM	DBE			
Minimum:				-1.5			
Maximum:		5.0	5.0	50.0			

Mass	Calc. Mass	mDa	PPM	DBE	i-FIT	i-FIT (Norm)	Formula
459.1399	459.1396	0.3	0.7	10.5	42.6	0.0	C22 H28 N4 O2 Br

Spectrum ^{1}H NMR (500 MHz, DMSO-*d6*) of compound **8e**

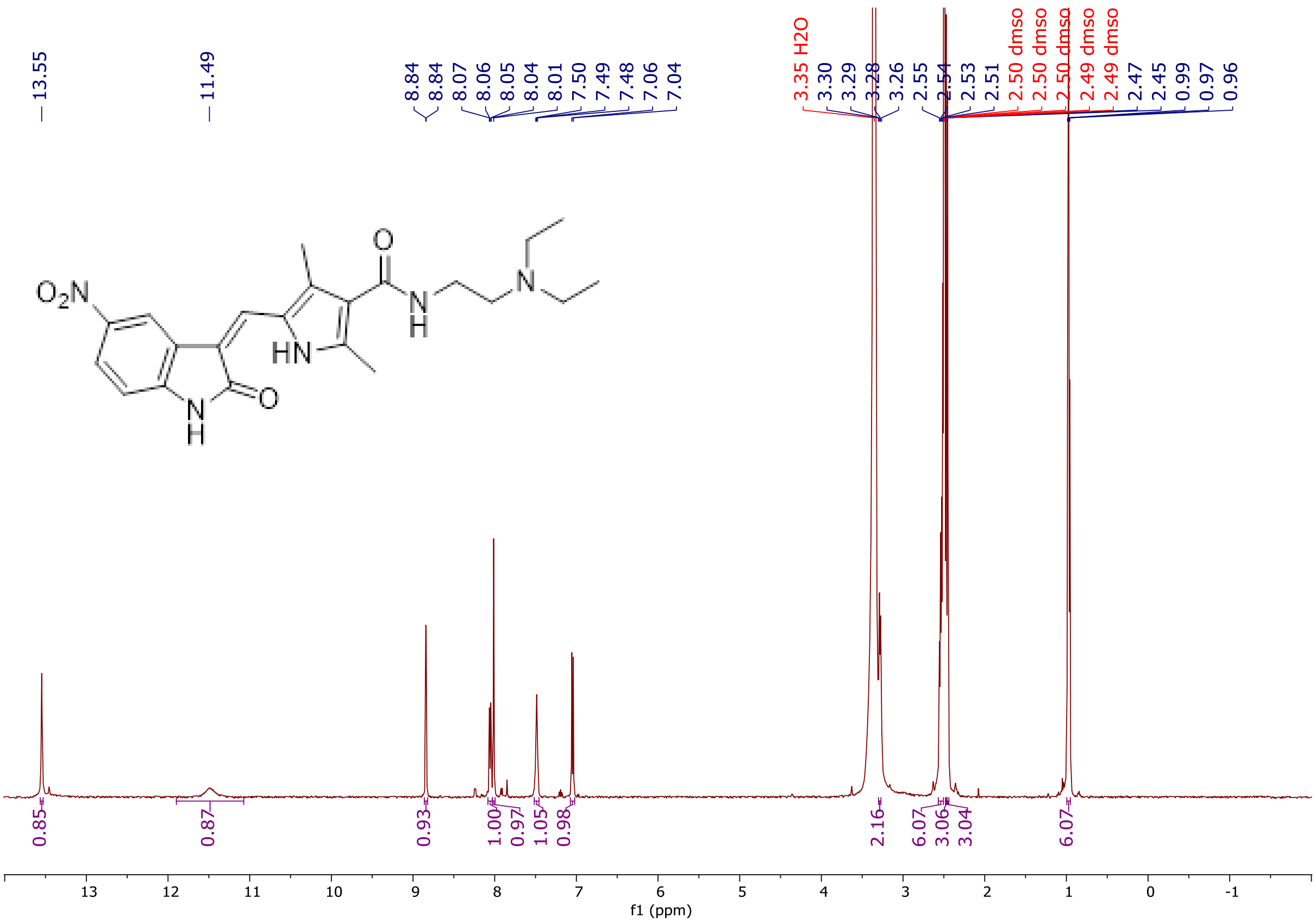

Spectrum ^{13}C NMR (125 MHz, DMSO-*d6*) of compound **8e**

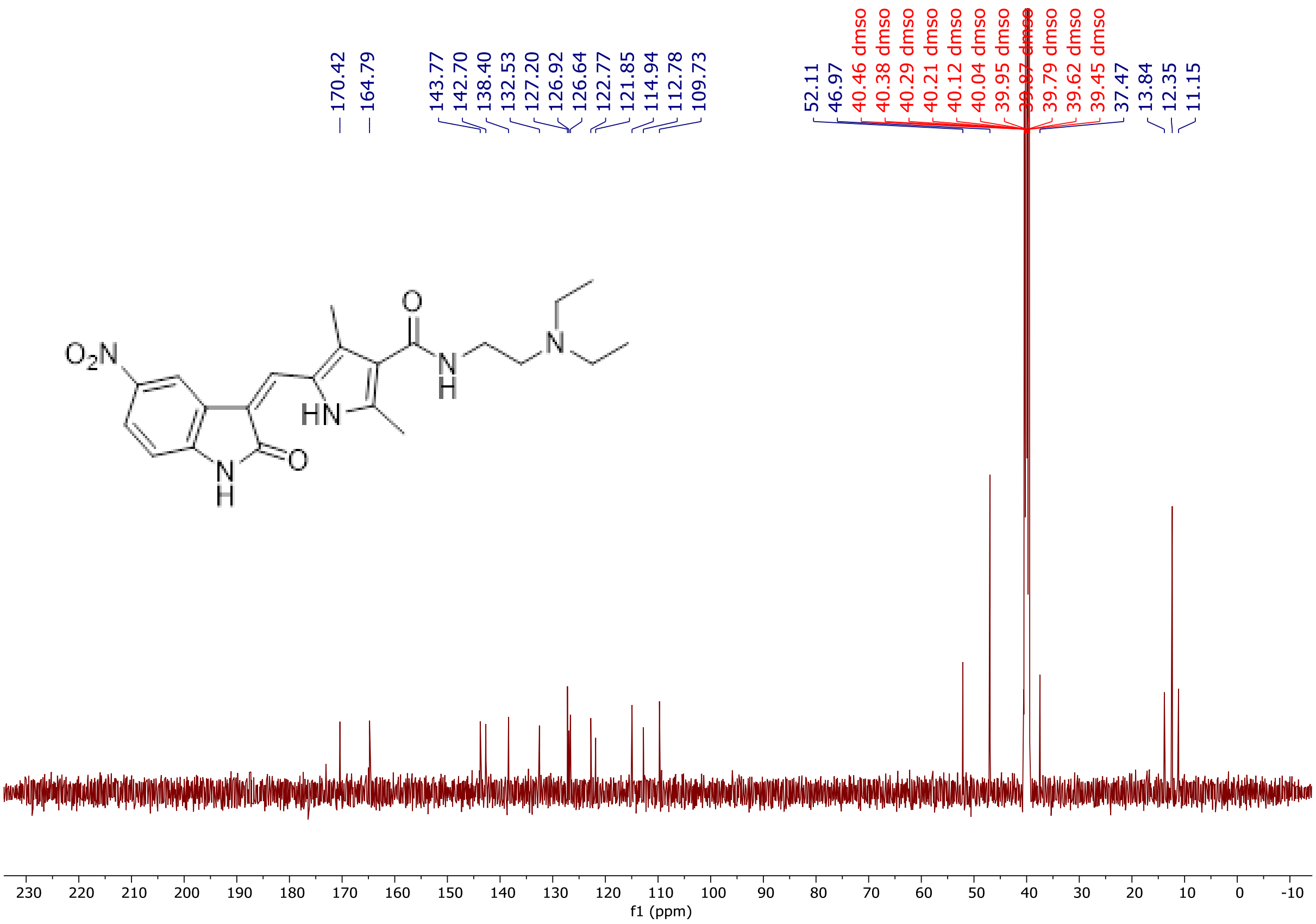

HRMS spectrum of compound **8e**

Single Mass Analysis
Tolerance = 5.0 PPM / DBE: min = -1.5, max = 50.0
Element prediction: Off
Number of isotope peaks used for i-FIT = 3

Monoisotopic Mass, Even Electron Ions
432 formula(e) evaluated with 1 results within limits (up to 50 best isotopic matches for each mass)
Elements Used:
C: 22-22 H: 0-100 N: 0-100 O: 0-100
Robby_RGN3_pos 7 (0.103)
TOF MS ES+

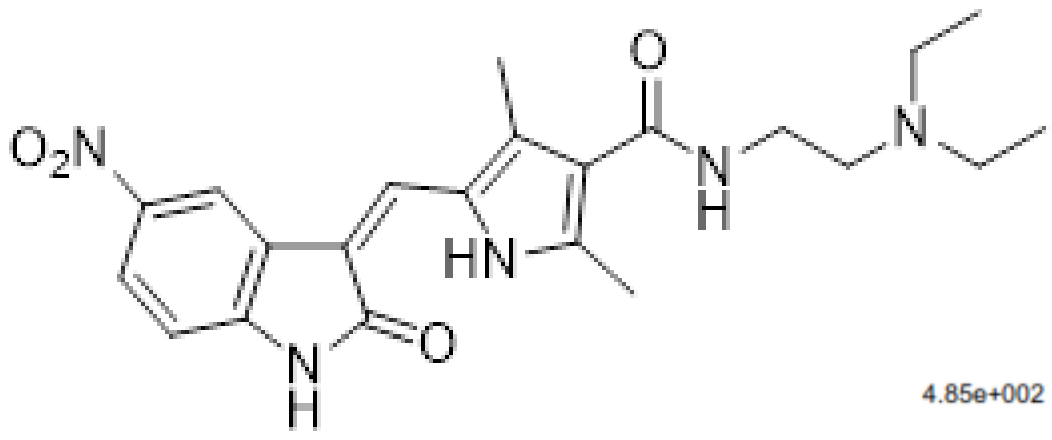

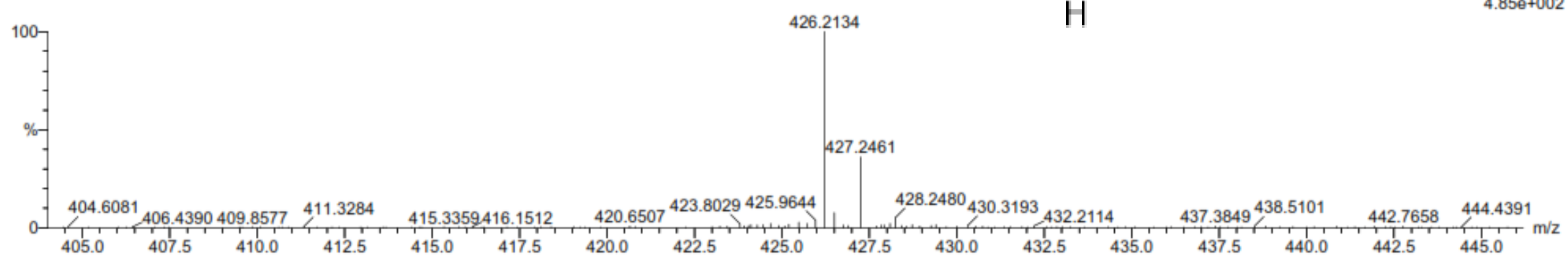

Minimum:				-1.5			
Maximum:		5.0	5.0	50.0			
Mass	Calc. Mass	mDa	PPM	DBE	i-FIT	i-FIT (Norm)	Formula
426.2134	426.2141	-0.7	-1.6	11.5	54.4	0.0	C22 H28 N5 O4

Spectrum ^{1}H NMR (500 MHz, DMSO-*d6*) of compound **8f**

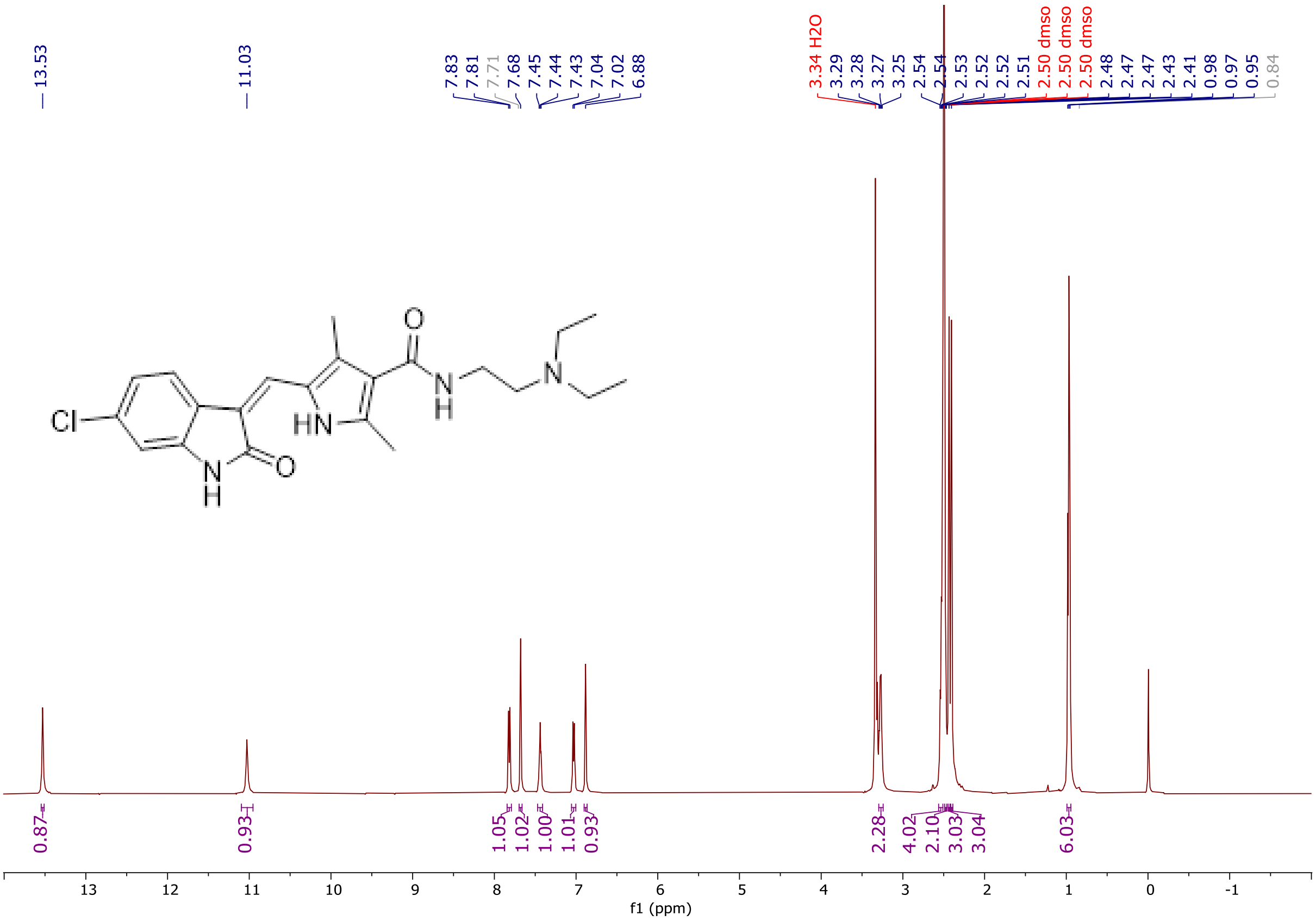

Spectrum ^{13}C NMR (125 MHz, DMSO-*d6*) of compound **8f**

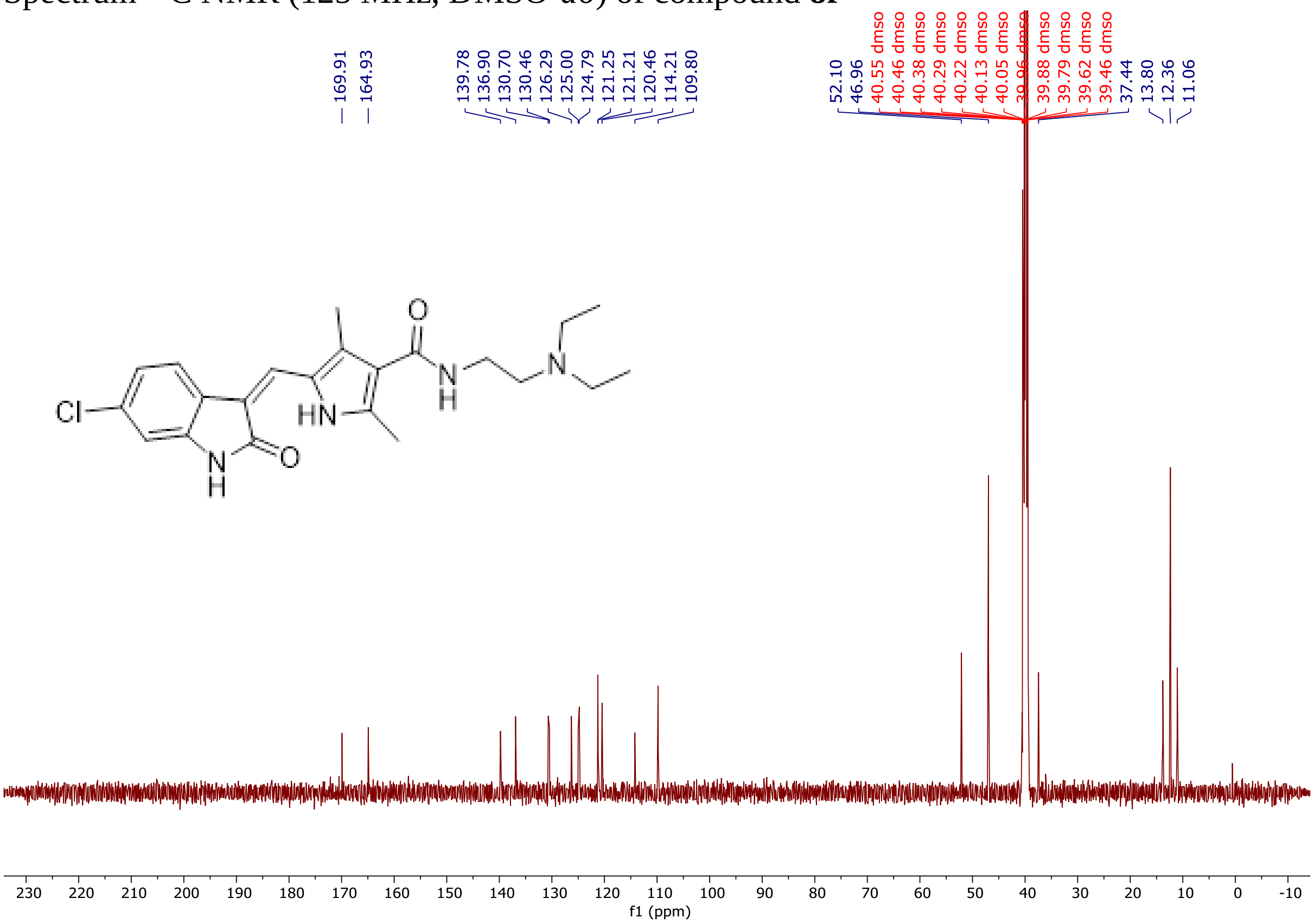

HRMS spectrum of compound **8f**

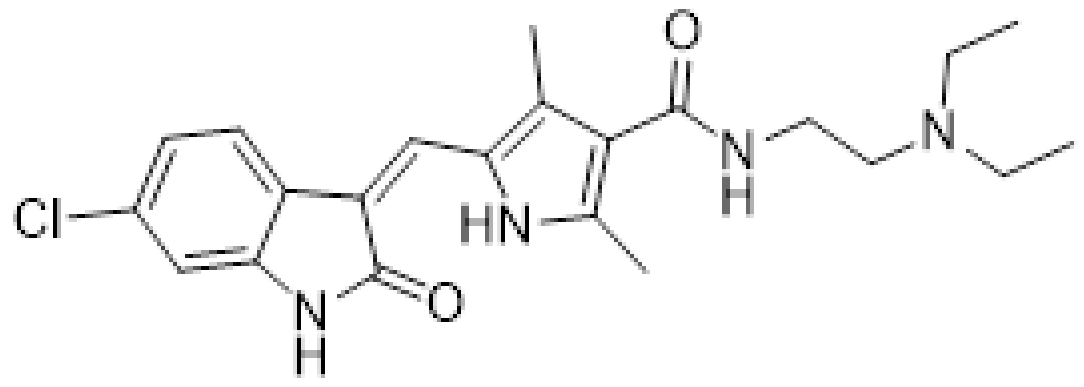

Single Mass Analysis
Tolerance = 5.0 PPM / DBE: min = -1.5, max = 50.0
Element prediction: Off
Number of isotope peaks used for i-FIT = 3

Monoisotopic Mass, Even Electron Ions
347 formula(e) evaluated with 1 results within limits (up to 50 best isotopic matches for each mass)
Elements Used:
C: 22-22 H: 0-100 N: 0-100 O: 0-100 Cl: 1-1
Robby_RGD1_pos 19 (0.307)
TOF MS ES+

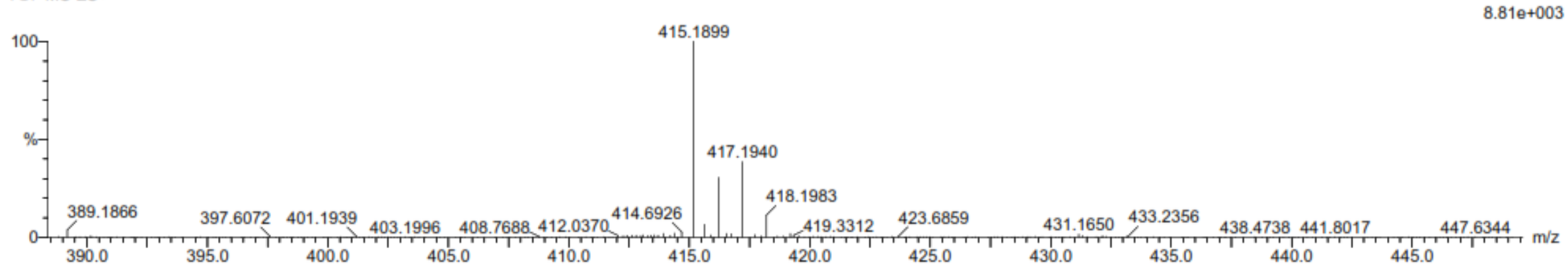

Minimum:				-1.5			
Maximum:		5.0	5.0	50.0			
Mass	Calc. Mass	mDa	PPM	DBE	i-FIT	i-FIT (Norm)	Formula
415.1899	415.1901	-0.2	-0.5	10.5	62.0	0.0	C22 H28 N4 O2 Cl

Spectrum ^{1}H NMR (500 MHz, DMSO-*d6*) of compound **8g**

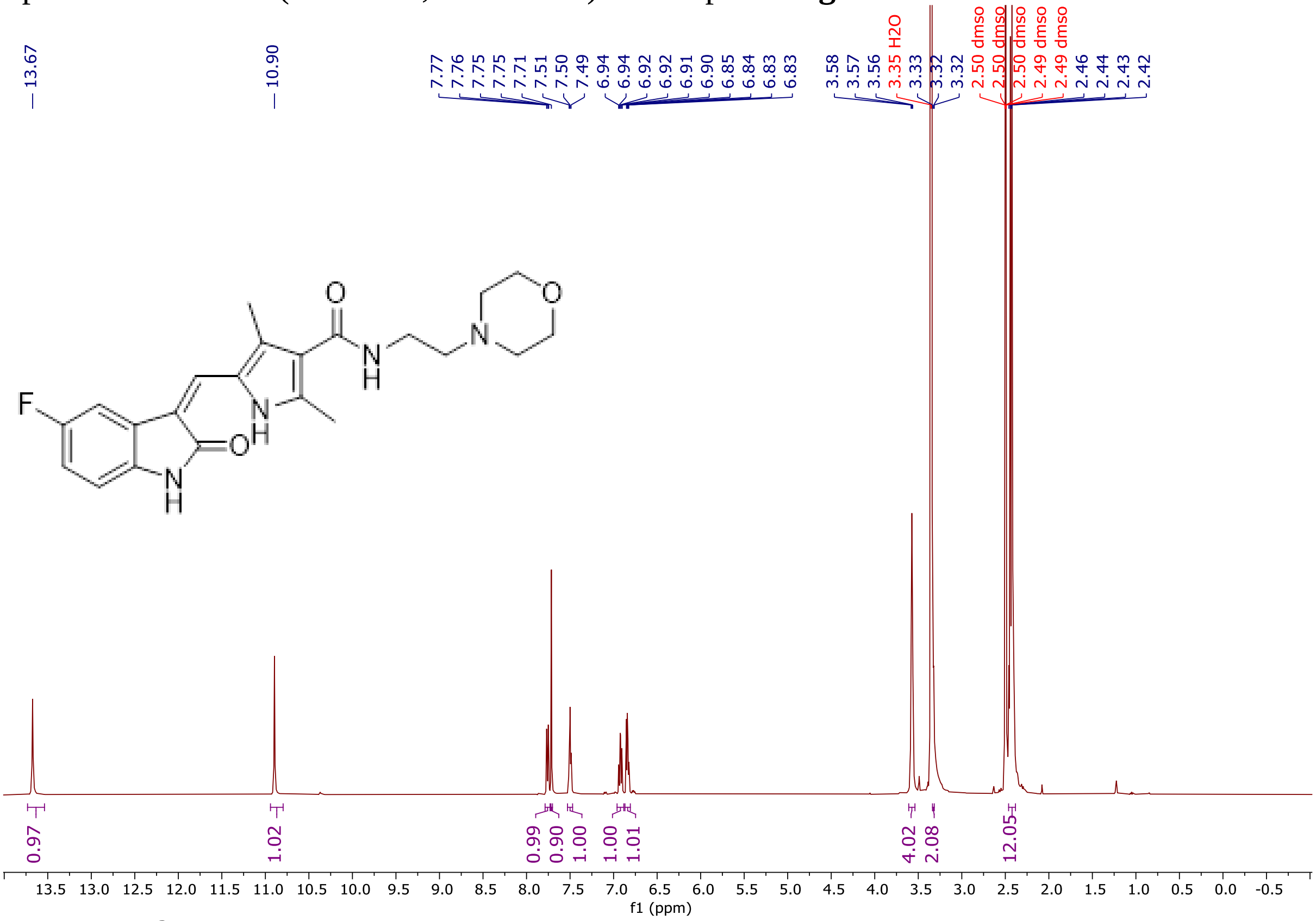

Spectrum ^{13}C NMR (125 MHz, DMSO-*d6*) of compound **8g**

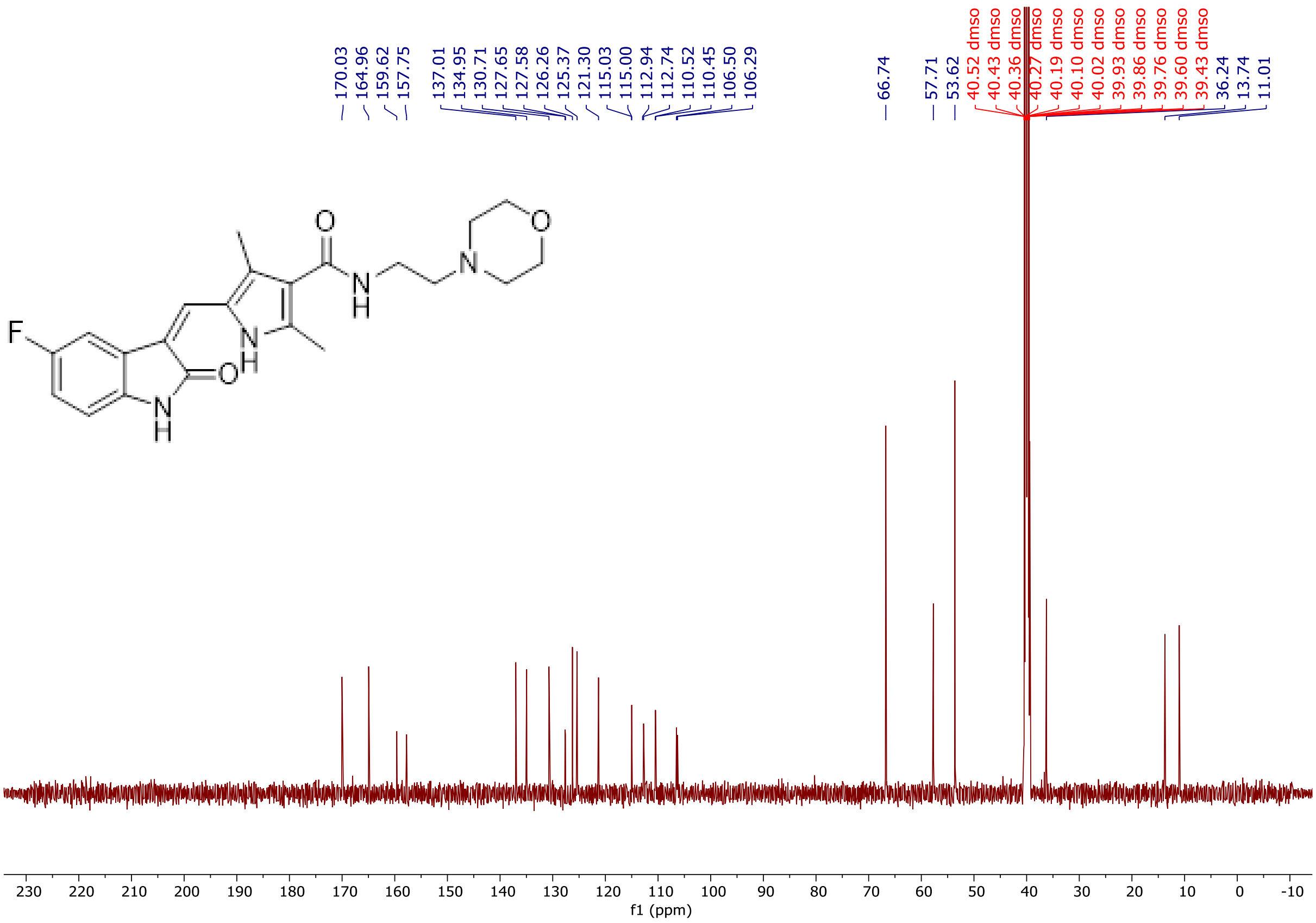

Spectrum ^{19}F NMR (470 MHz, DMSO-d_6) of compound **8g**

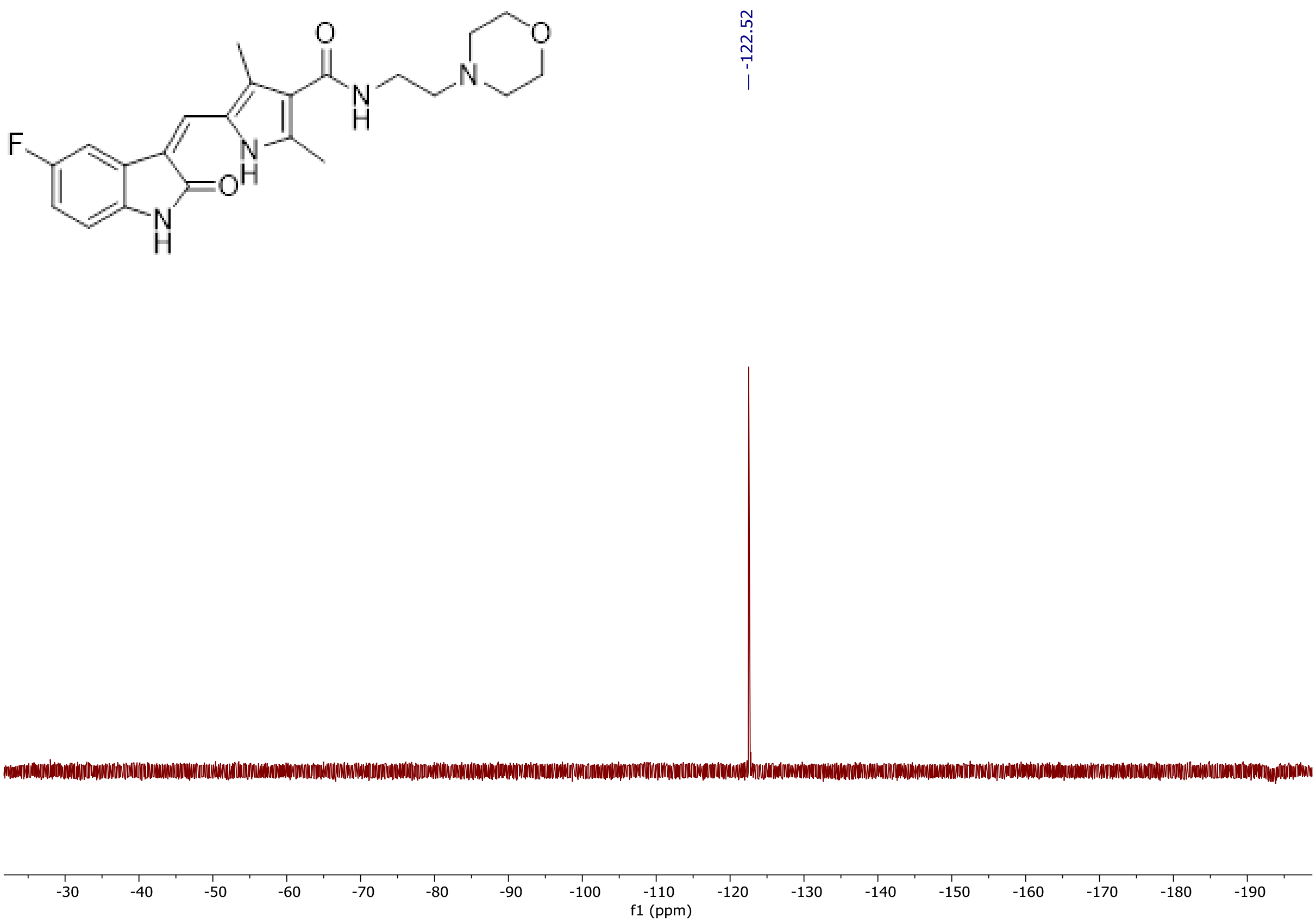

HRMS spectrum of compound **8g**

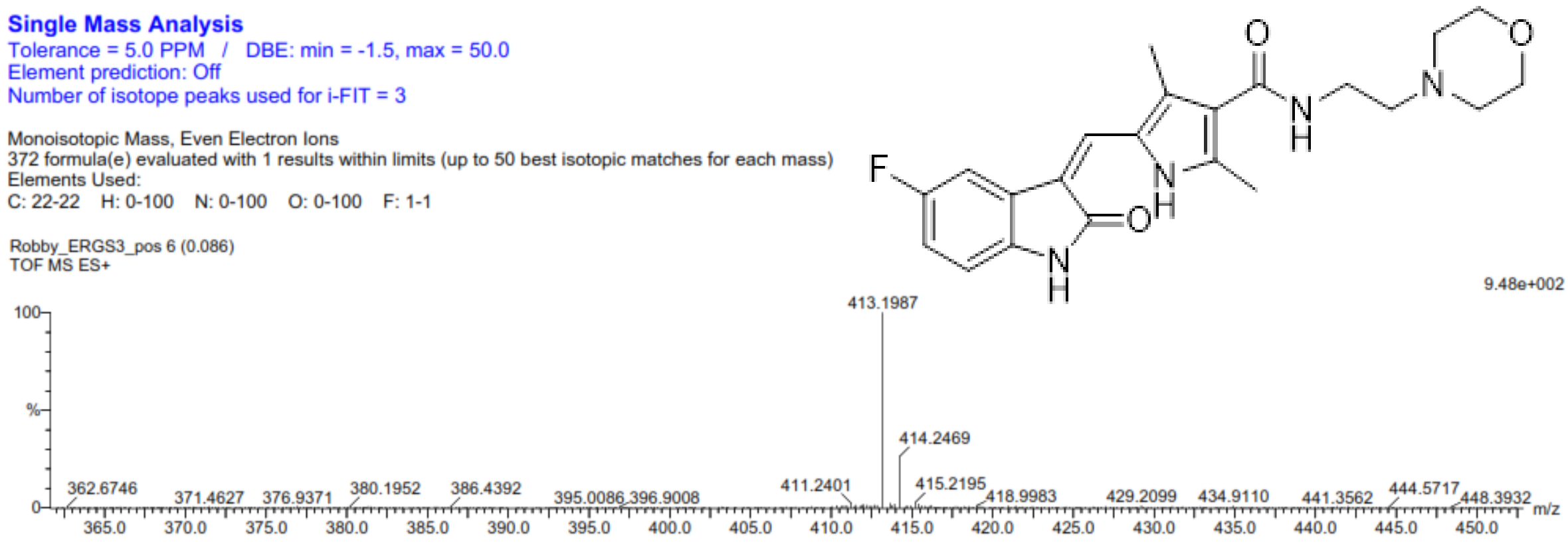

Minimum:				-1.5			
Maximum:		5.0	5.0	50.0			
Mass	Calc. Mass	mDa	PPM	DBE	i-FIT	i-FIT (Norm)	Formula
413.1987	413.1989	-0.2	-0.5	11.5	58.0	0.0	C22 H26 N4 O3 F

Spectrum ^{1}H NMR (500 MHz, DMSO-*d6*) of compound **8h**

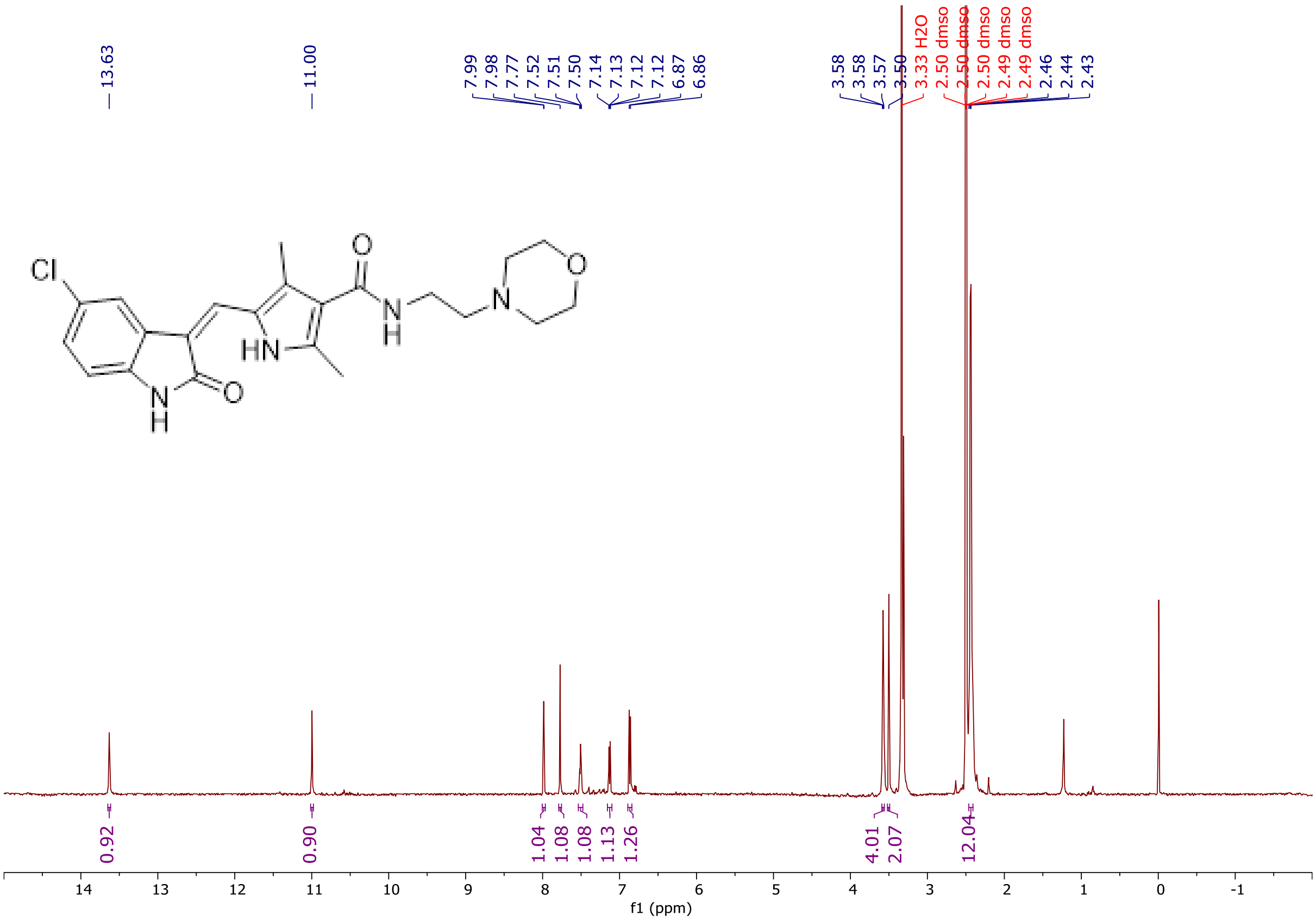

Spectrum ^{13}C NMR (125 MHz, DMSO-*d6*) of compound **8h**

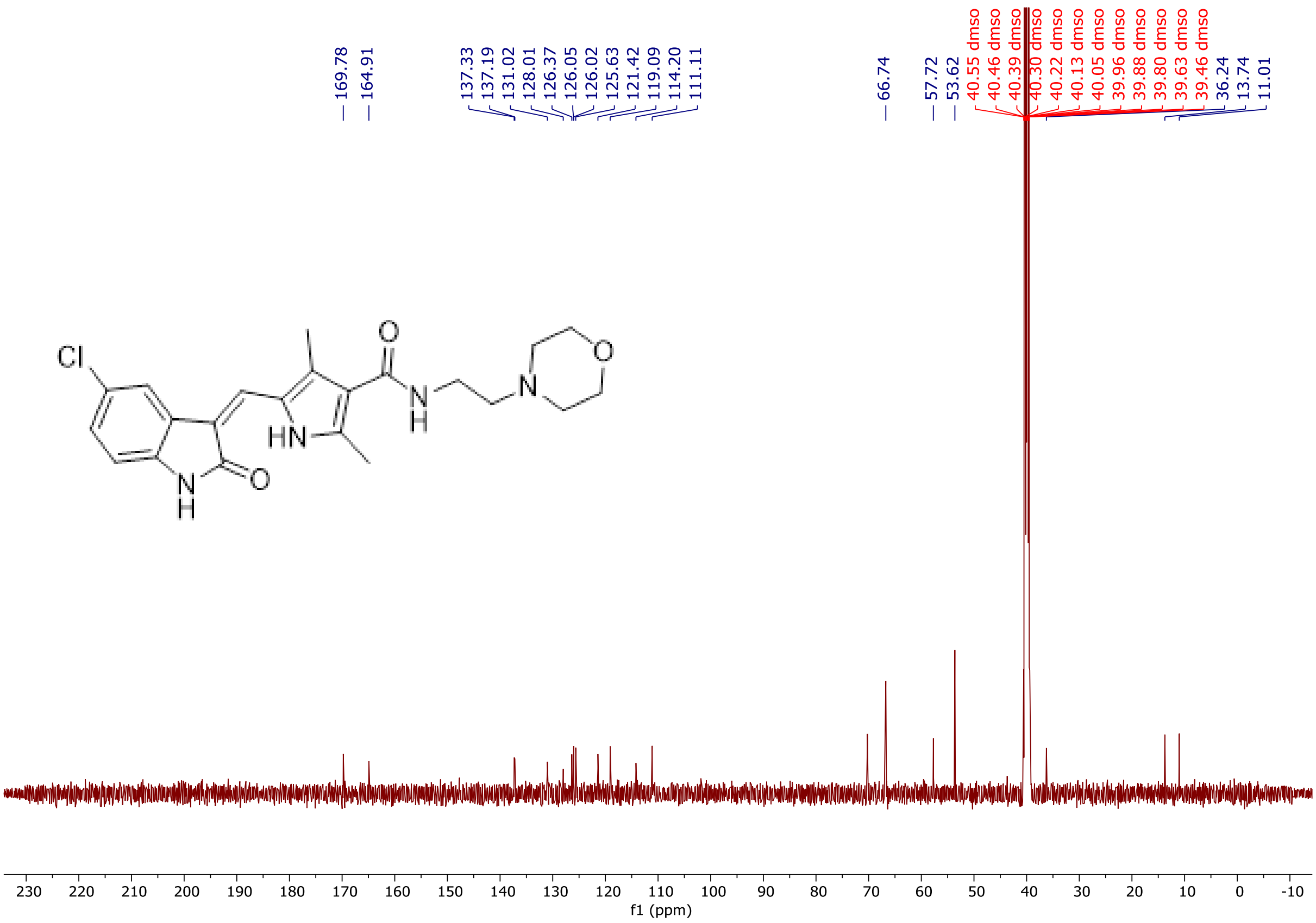

HRMS spectrum of compound **8h**

Single Mass Analysis
Tolerance = 5.0 PPM / DBE: min = -1.5, max = 50.0
Element prediction: Off
Number of isotope peaks used for i-FIT = 3

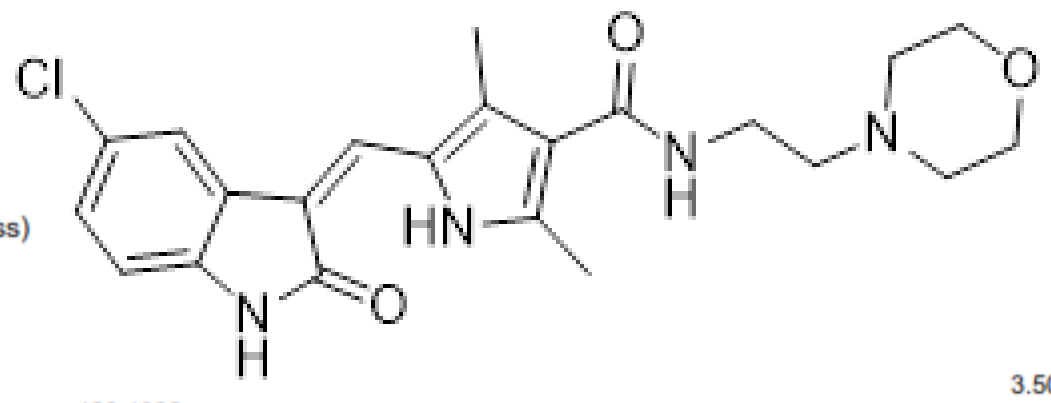

Monoisotopic Mass, Even Electron Ions
372 formula(e) evaluated with 1 results within limits (up to 50 best isotopic matches for each mass)
Elements Used:
C: 22-22 H: 0-100 N: 0-100 O: 0-100 Cl: 1-1
Robby_RGC3_pos 49 (0.819)
TOF MS ES+

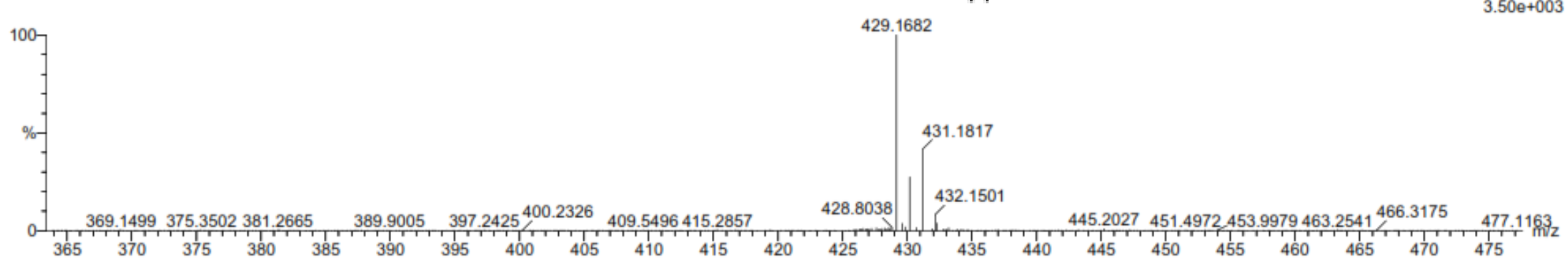

Minimum:				-1.5			
Maximum:		5.0	5.0	50.0			
Mass	Calc. Mass	mDa	PPM	DBE	i-FIT	i-FIT (Norm)	Formula
429.1682	429.1693	-1.1	-2.6	11.5	45.0	0.0	C22 H26 N4 O3 Cl

Spectrum ^{1}H NMR (500 MHz, DMSO-*d6*) of compound **8i**

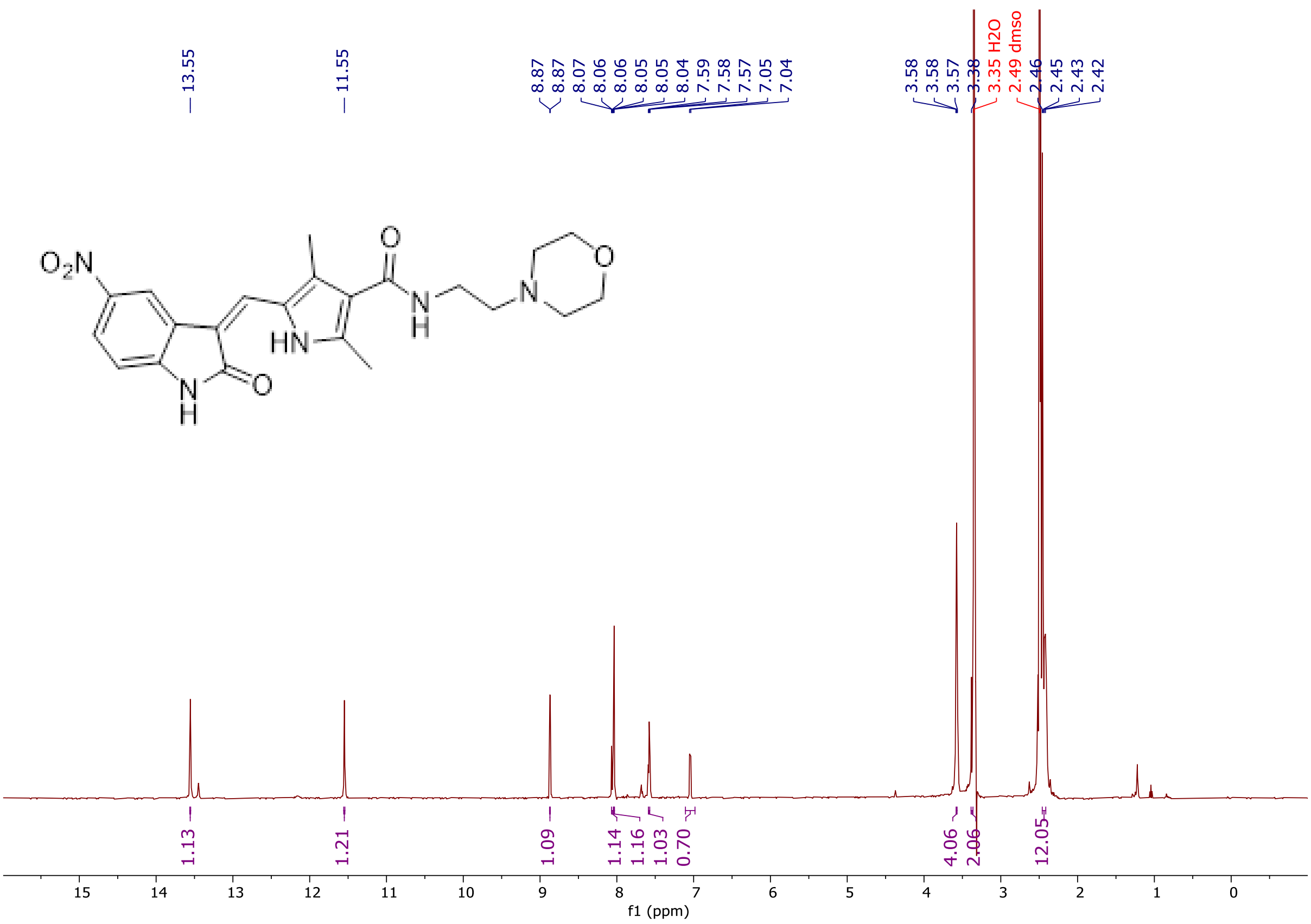

Spectrum ^{13}C NMR (125 MHz, DMSO-*d6*) of compound **8i**

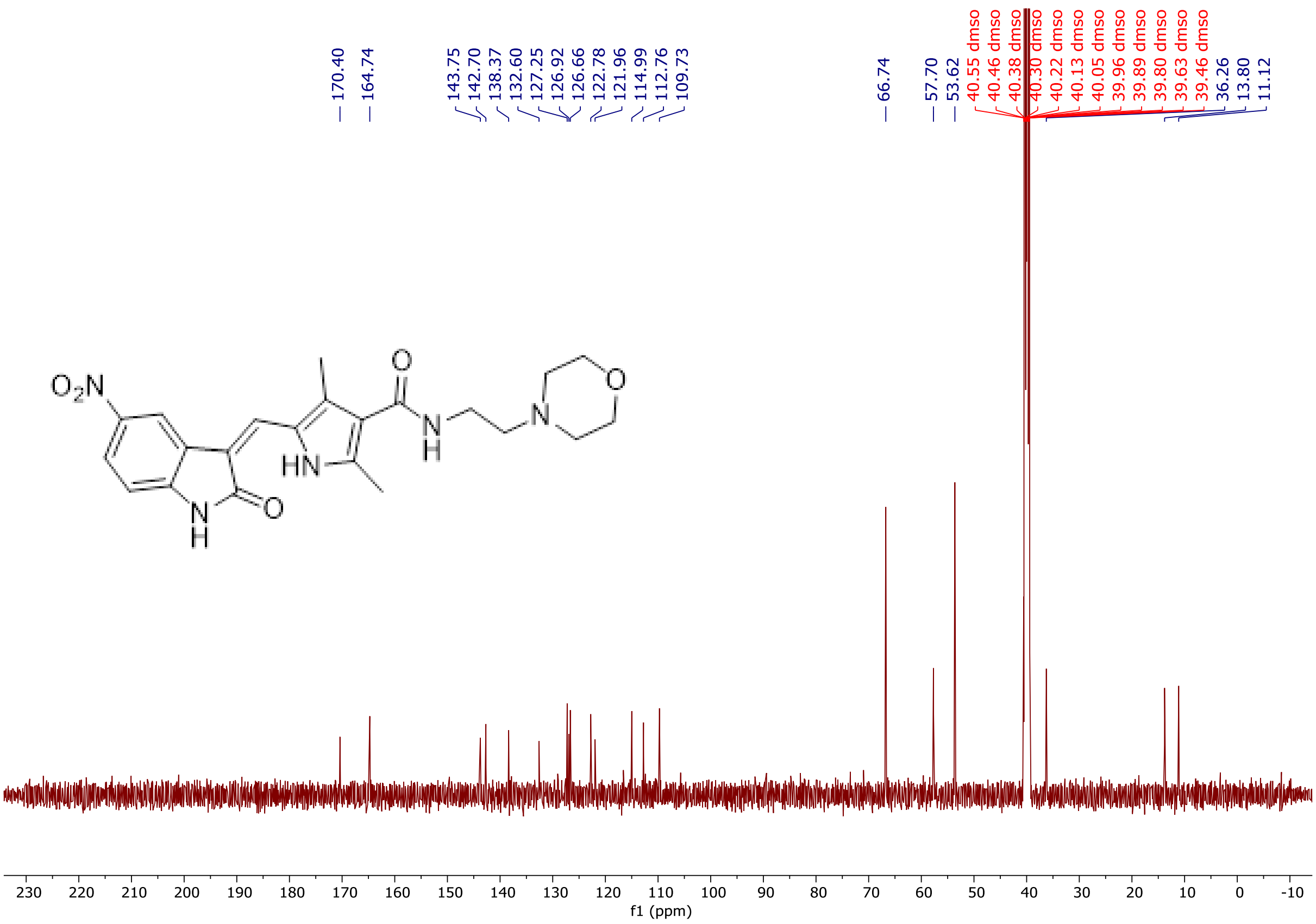

HRMS spectrum of compound **8i**

Single Mass Analysis
Tolerance = 5.0 PPM / DBE: min = -1.5, max = 50.0
Element prediction: Off
Number of isotope peaks used for i-FIT = 3

Monoisotopic Mass, Even Electron Ions
460 formula(e) evaluated with 1 results within limits (up to 50 best isotopic matches for each mass)
Elements Used:
C: 22-22 H: 0-100 N: 0-100 O: 0-100
Robby_RGN2_pos 43 (0.717)
TOF MS ES+

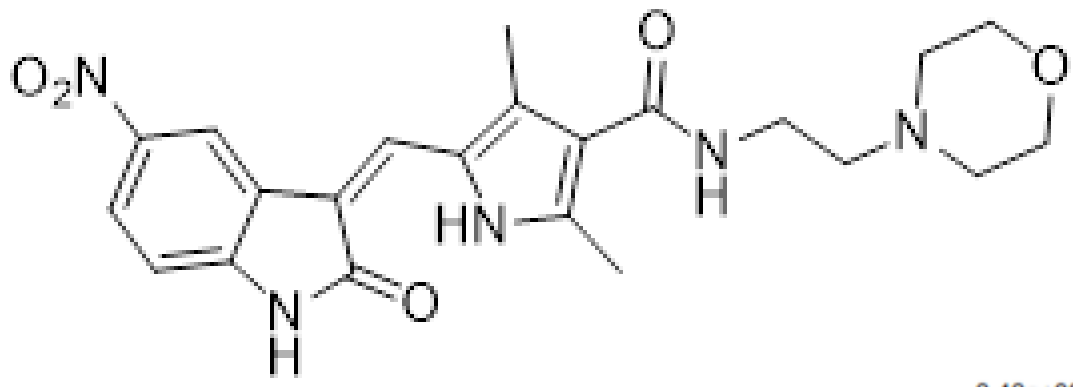

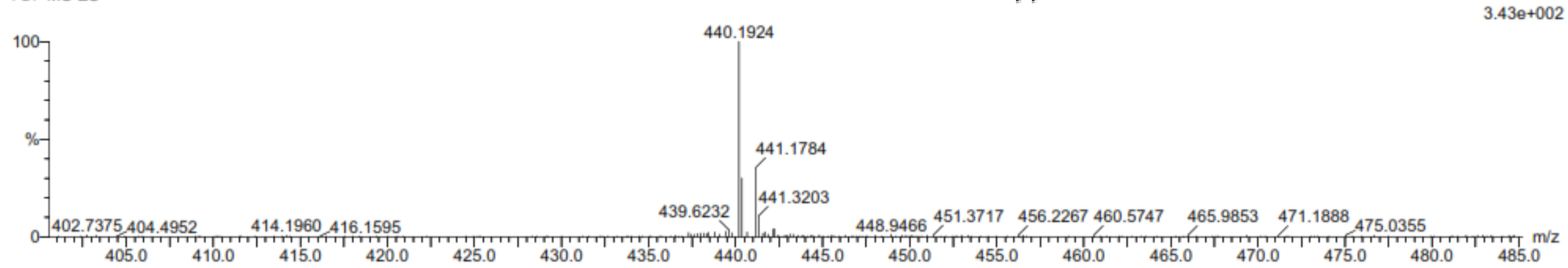

Minimum:				-1.5			
Maximum:		5.0	5.0	50.0			
Mass	Calc. Mass	mDa	PPM	DBE	i-FIT	i-FIT (Norm)	Formula
440.1924	440.1934	-1.0	-2.3	12.5	58.0	0.0	C22 H26 N5 O5

Spectrum ^{1}H NMR (500 MHz, DMSO-*d6*) of compound **8j**

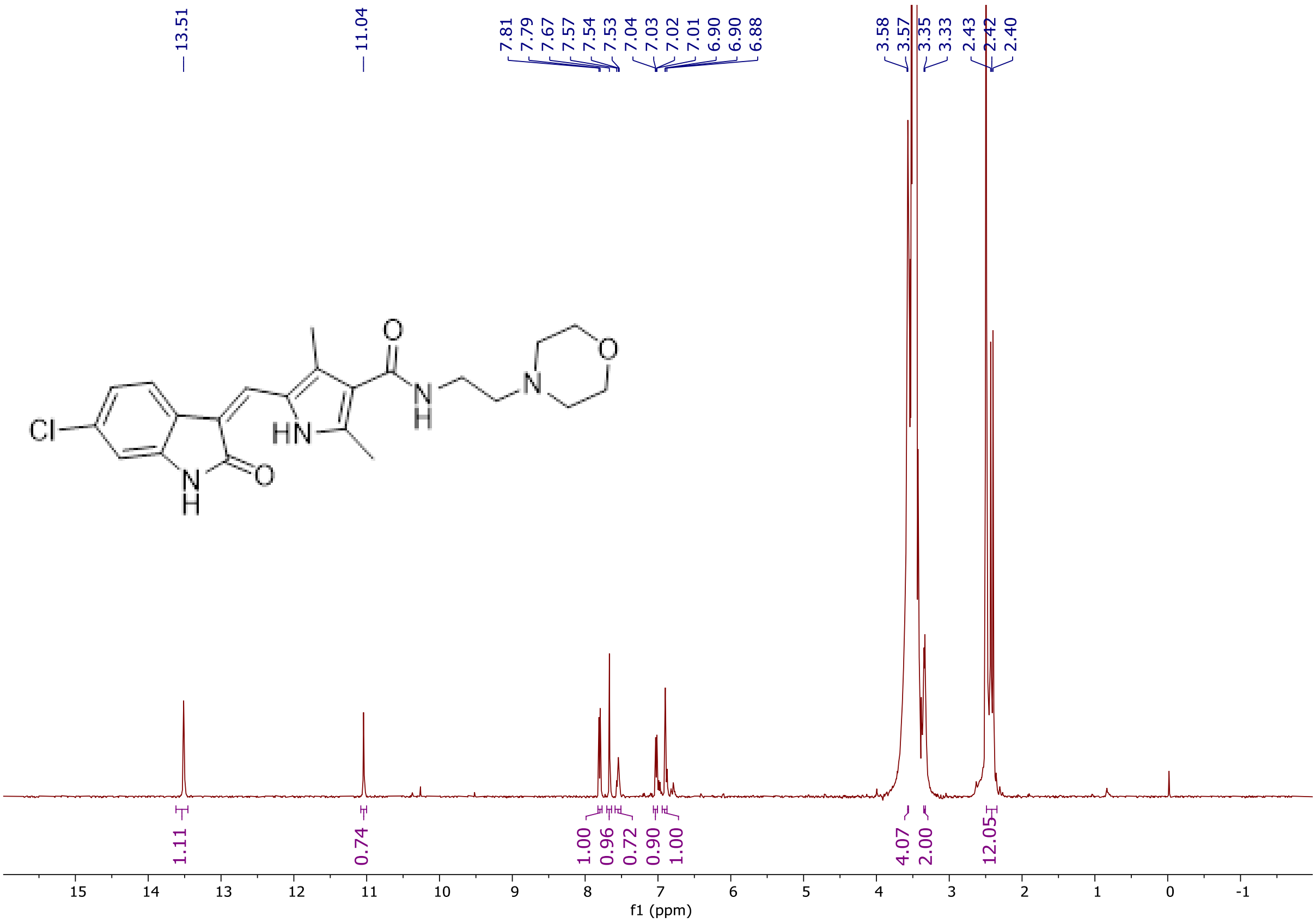

Spectrum ^{13}C NMR (125 MHz, DMSO-*d6*) of compound **8j**

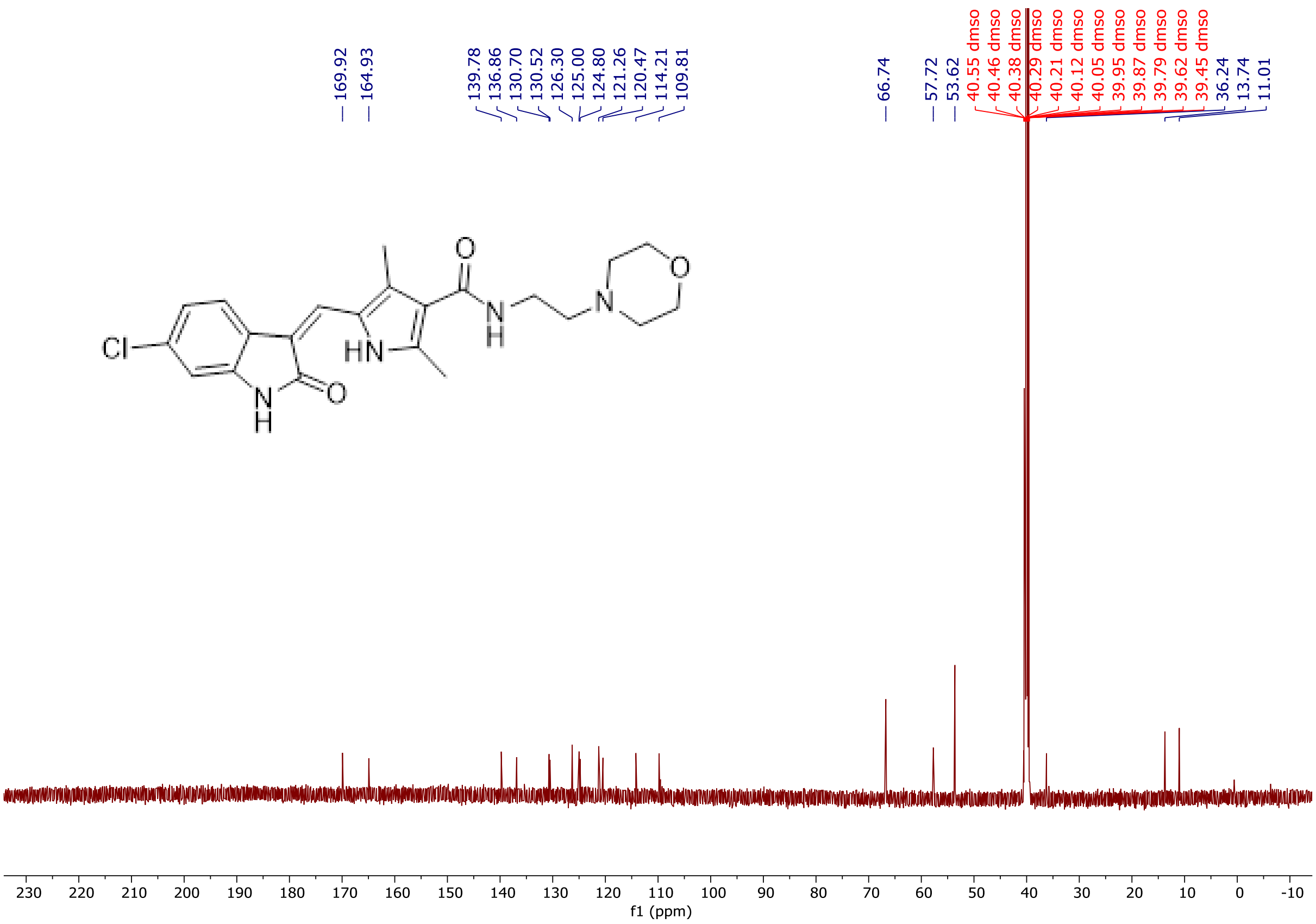

HRMS spectrum of compound **8j**

Single Mass Analysis
Tolerance = 5.0 PPM / DBE: min = -1.5, max = 50.0
Element prediction: Off
Number of isotope peaks used for i-FIT = 3

Monoisotopic Mass, Even Electron Ions
372 formula(e) evaluated with 1 results within limits (up to 50 best isotopic matches for each mass)
Elements Used:
C: 22-22 H: 0-100 N: 0-100 O: 0-100 Cl: 1-1
Robby_RGD3_pos 15 (0.239)
TOF MS ES+

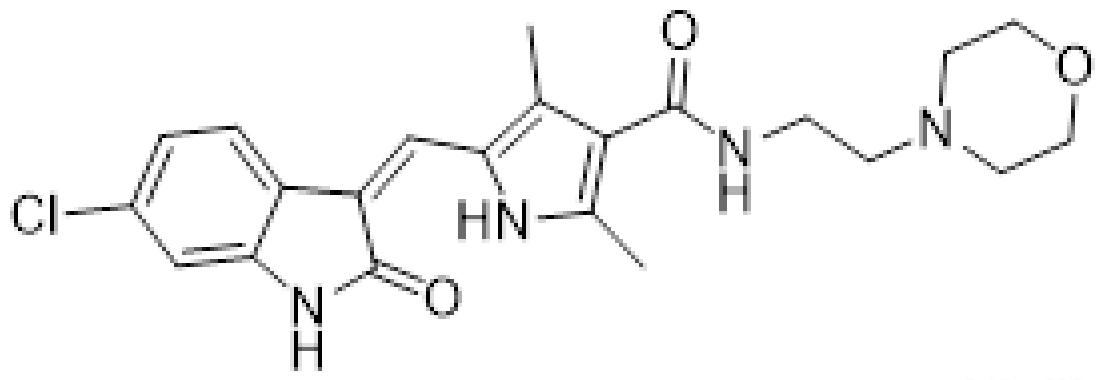

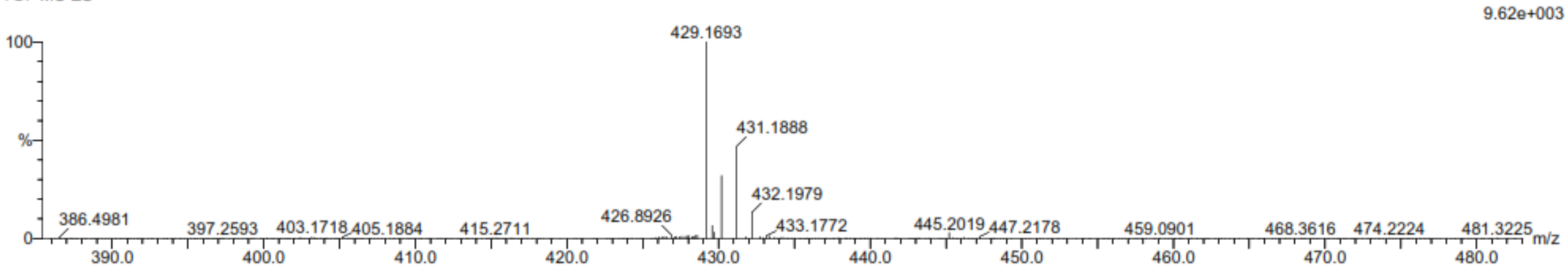

Mass	Calc. Mass	mDa	PPM	DBE	i-FIT	i-FIT (Norm)	Formula
Minimum:				-1.5			
Maximum:		5.0	5.0	50.0			
429.1693	429.1693	0.0	0.0	11.5	51.5	0.0	C22 H26 N4 O3 Cl

Spectrum ^{1}H NMR (500 MHz, DMSO-*d6*) of compound **8k**

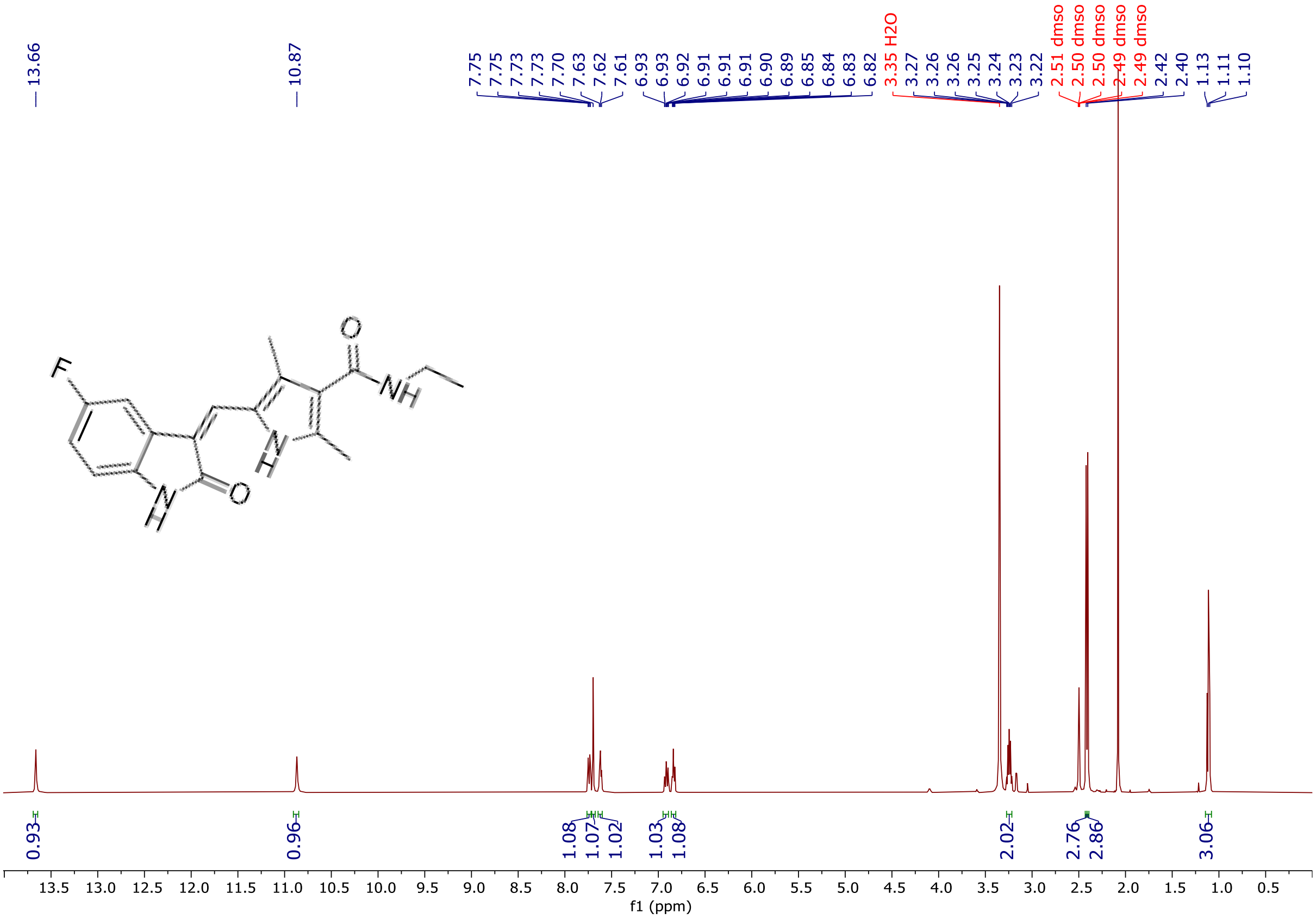

Spectrum ^{13}C NMR (125 MHz, DMSO-*d6*) of compound **8k**

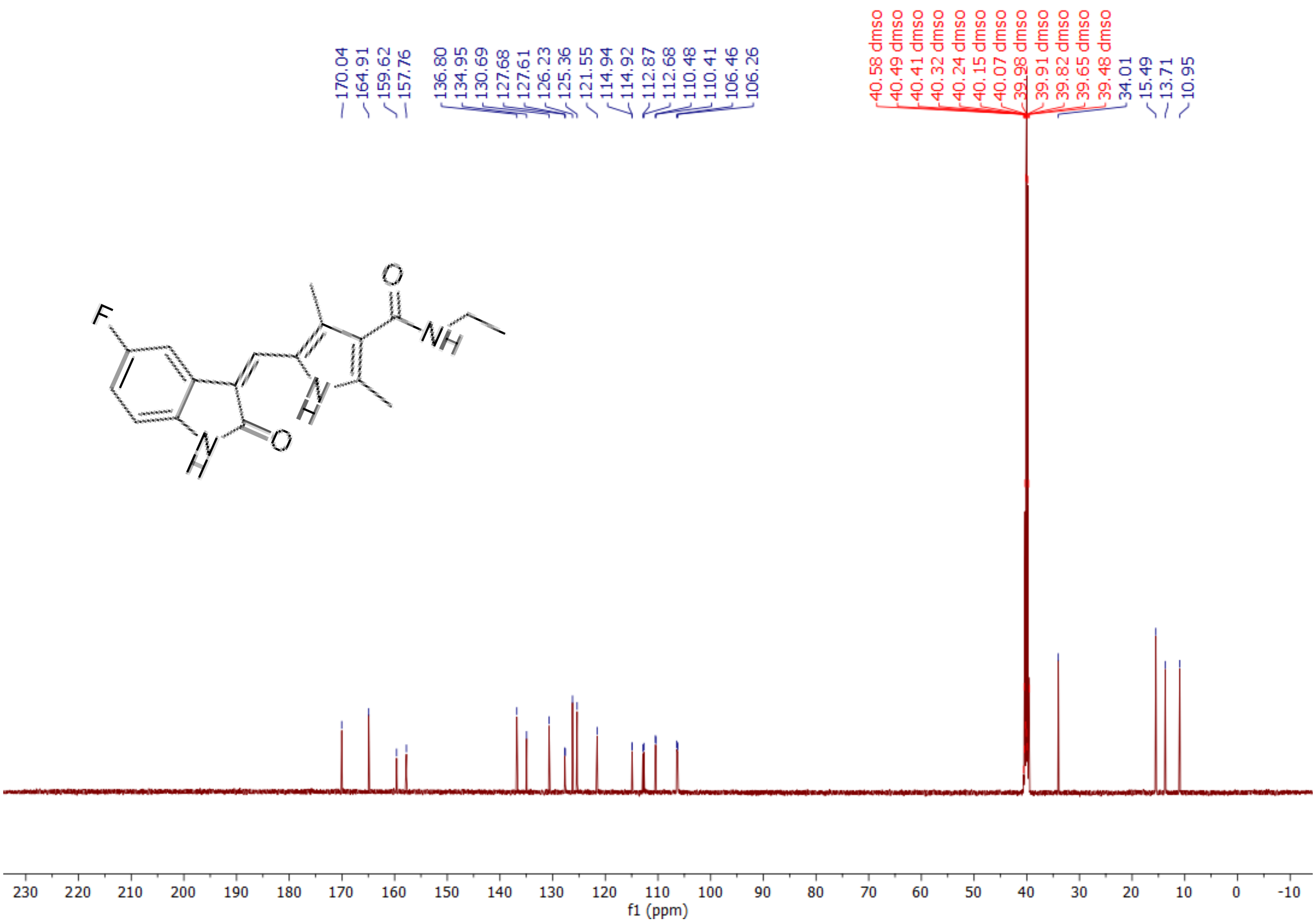

Spectrum ^{19}F NMR (470 MHz, DMSO-d_6) of compound **8k**

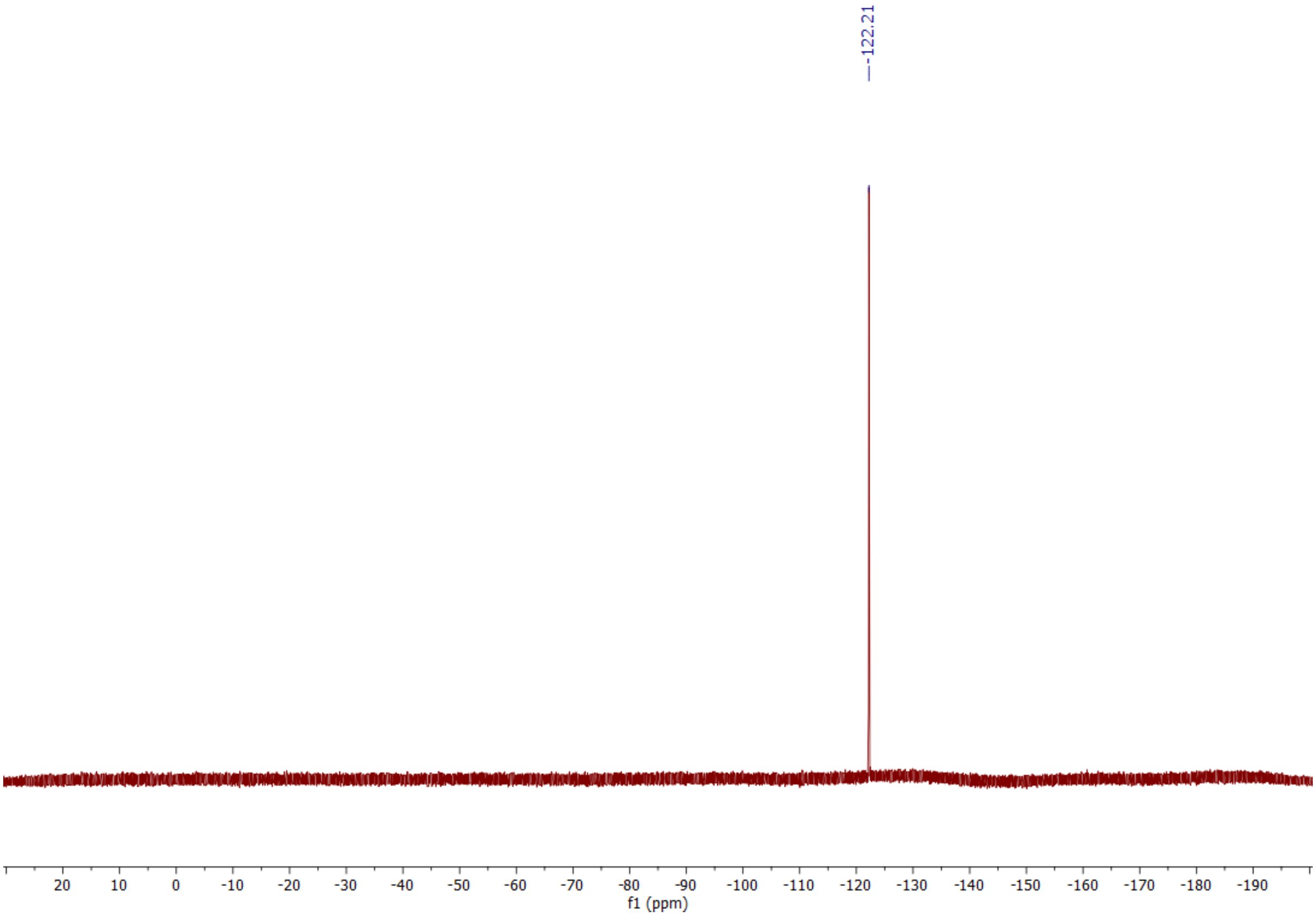

HRMS spectrum of compound **8k**

Single Mass Analysis
Tolerance = 10.0 PPM / DBE: min = -1.5, max = 50.0
Element prediction: Off
Number of isotope peaks used for i-FIT = 3

Monoisotopic Mass, Even Electron Ions
147 formula(e) evaluated with 1 results within limits (up to 3 best isotopic matches for each mass)
Elements Used:
C: 0-25 H: 0-30 N: 0-4 O: 0-10 F: 1-1

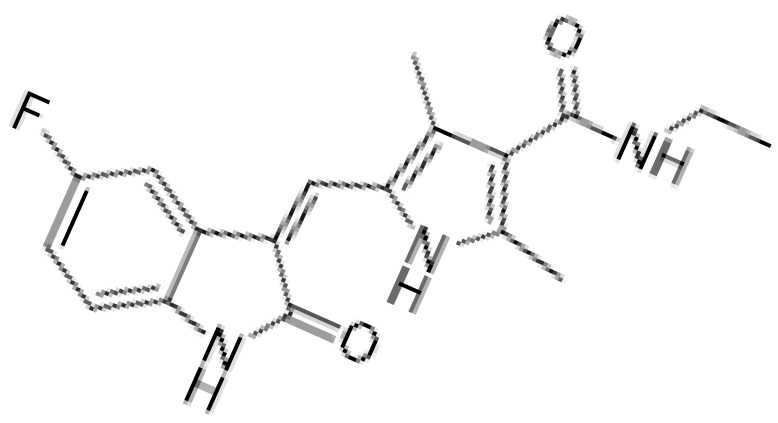

Robby_RGS06_neg 6 (0.085)
TOF MS ES-

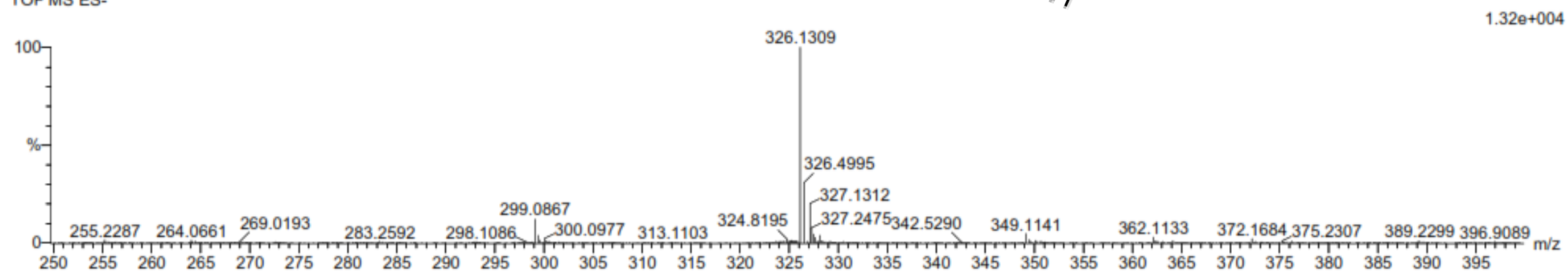

Minimum:				-1.5			
Maximum:		5.0	10.0	50.0			
Mass	Calc. Mass	mDa	PPM	DBE	i-FIT	i-FIT (Norm)	Formula
326.1309	326.1305	0.4	1.2	11.5	118.4	0.0	C18 H17 N3 O2 F

Spectrum ^{1}H NMR (500 MHz, DMSO-*d6*) of compound **8l**

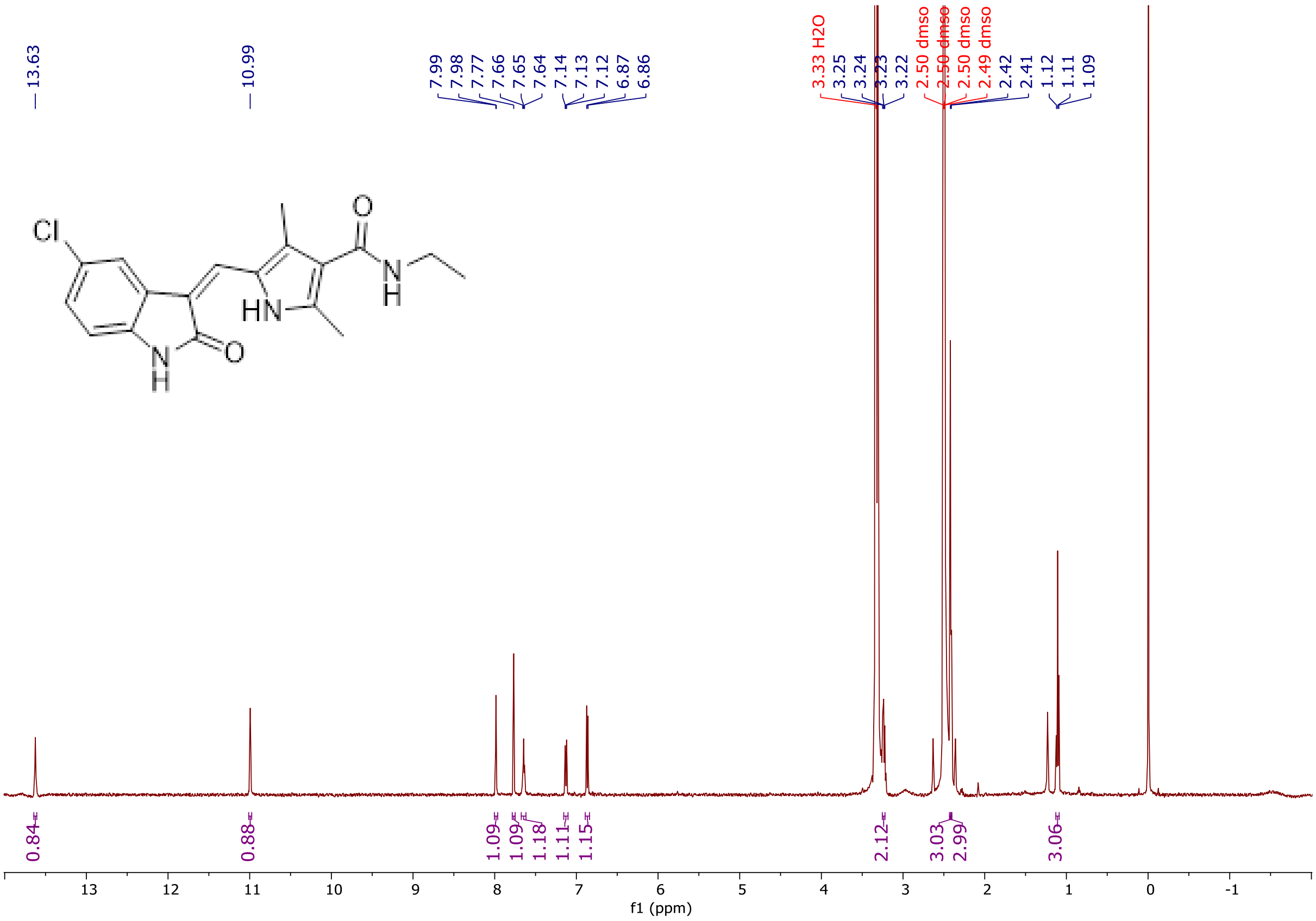

Spectrum ^{13}C NMR (125 MHz, DMSO-*d6*) of compound **8l**

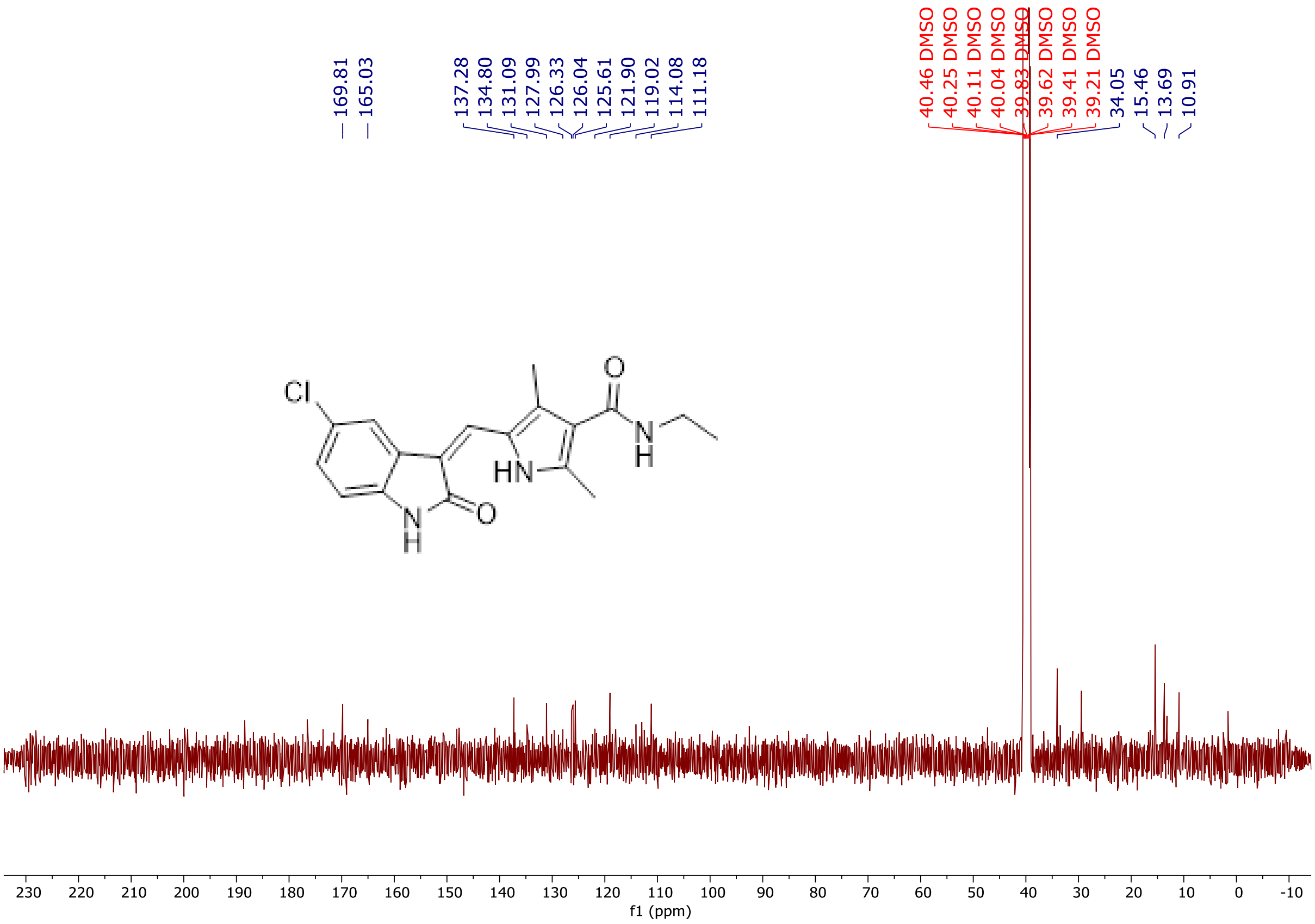

HRMS spectrum of compound **8l**

Single Mass Analysis
Tolerance = 5.0 PPM / DBE: min = -1.5, max = 50.0
Element prediction: Off
Number of isotope peaks used for i-FIT = 3

Monoisotopic Mass, Even Electron Ions
232 formula(e) evaluated with 1 results within limits (up to 50 best isotopic matches for each mass)
Elements Used:
C: 18-18 H: 0-100 N: 0-100 O: 0-100 Cl: 1-1
Robby_RGC5_pos 12 (0.188) Cm (11:14)
TOF MS ES+

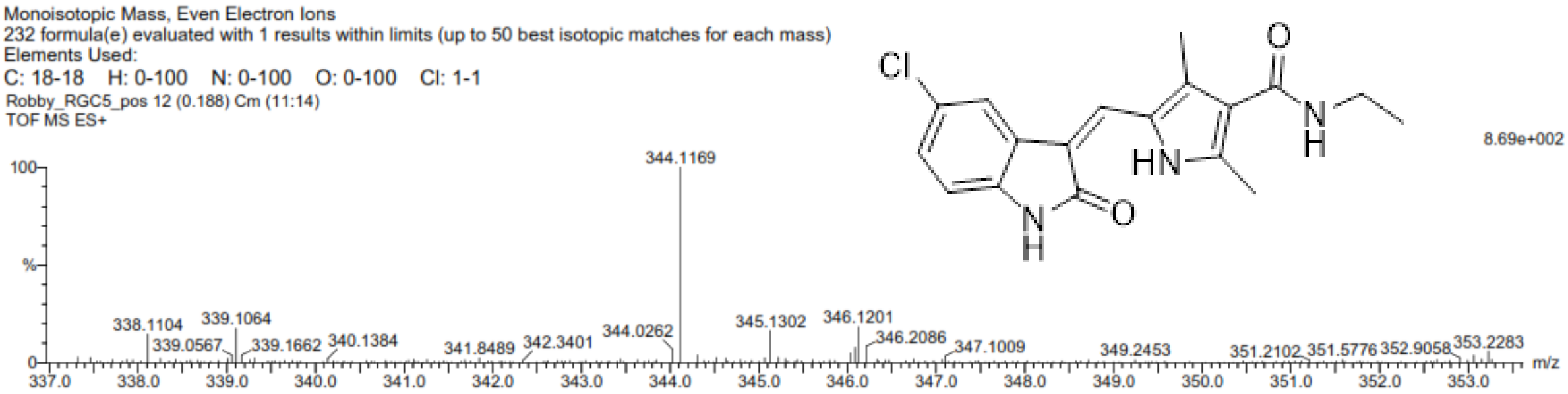

Minimum:				-1.5			
Maximum:		5.0	5.0	50.0			
Mass	Calc. Mass	mDa	PPM	DBE	i-FIT	i-FIT (Norm)	Formula
344.1169	344.1166	0.3	0.9	10.5	170.9	0.0	C18 H19 N3 O2 Cl

Spectrum ^{1}H NMR (500 MHz, DMSO-*d6*) of compound **8m**

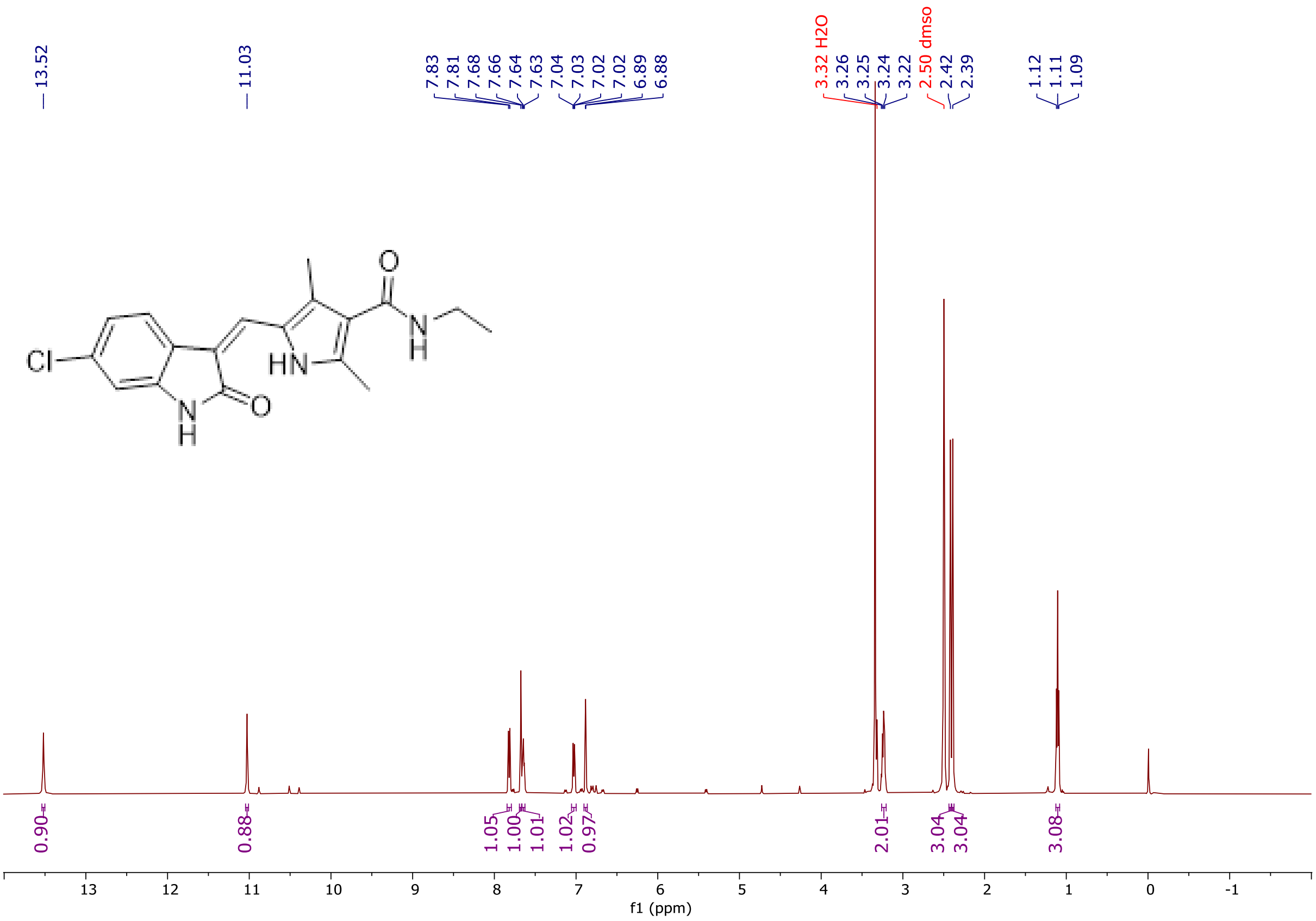

Spectrum ^{13}C NMR (125 MHz, DMSO-*d6*) of compound **8m**

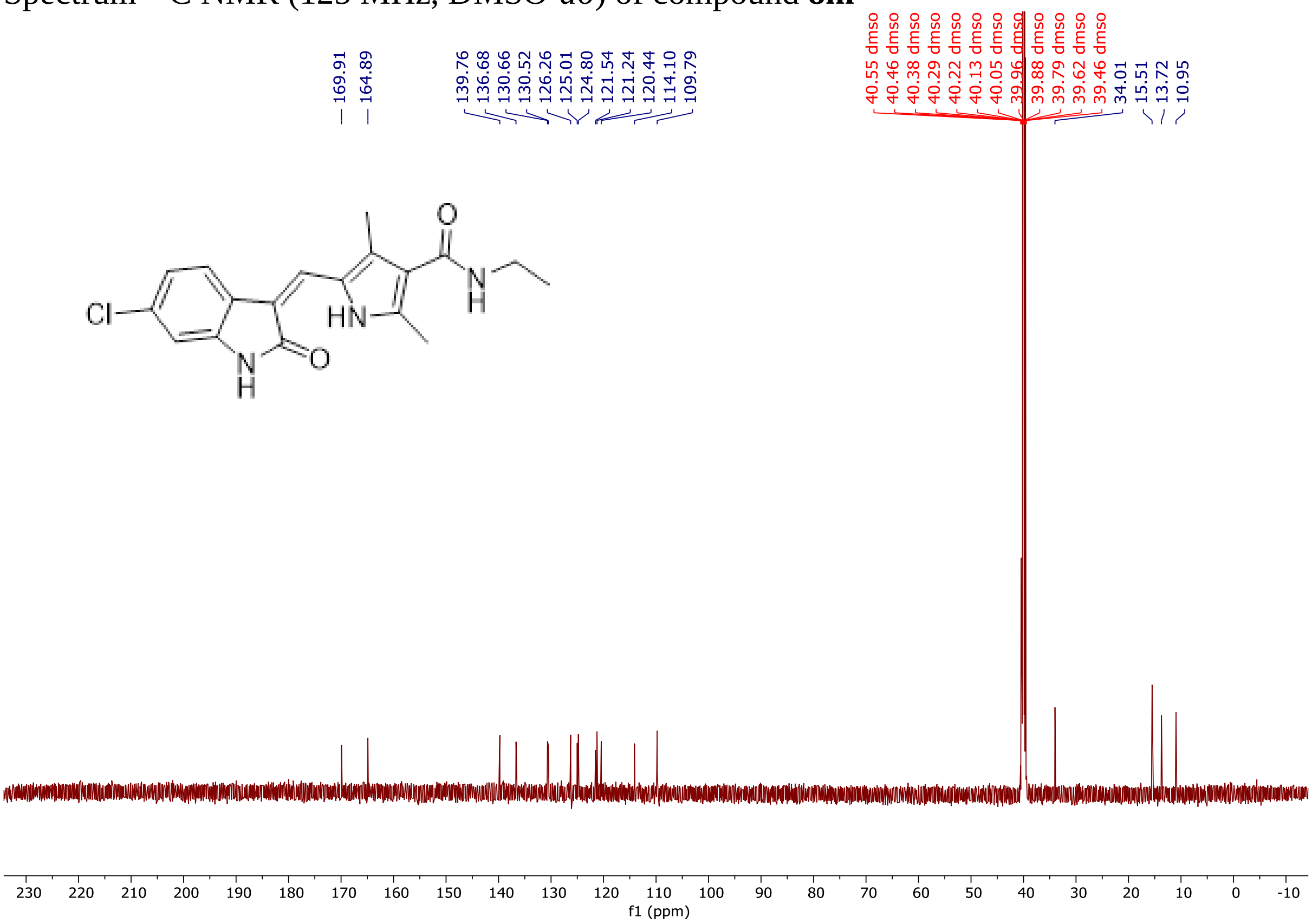

HRMS spectrum of compound **8m**

Single Mass Analysis
Tolerance = 5.0 PPM / DBE: min = -1.5, max = 50.0
Element prediction: Off
Number of isotope peaks used for i-FIT = 3

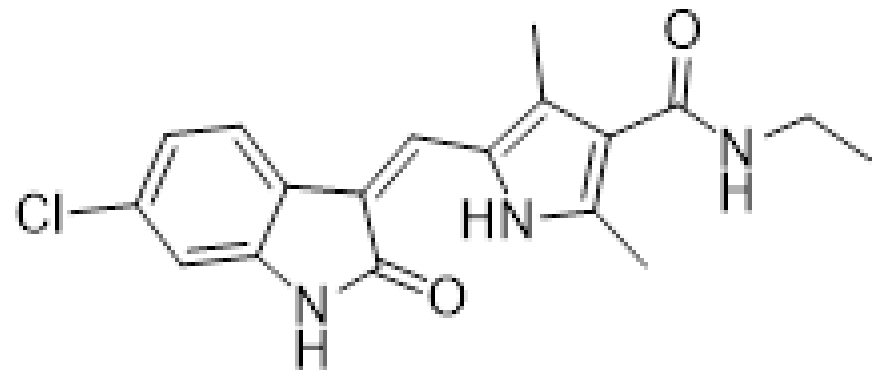

Monoisotopic Mass, Even Electron Ions
232 formula(e) evaluated with 1 results within limits (up to 50 best isotopic matches for each mass)
Elements Used:
C: 18-18 H: 0-100 N: 0-100 O: 0-100 Cl: 1-1
Robby_RGD5_pos 37 (0.614)
TOF MS ES+

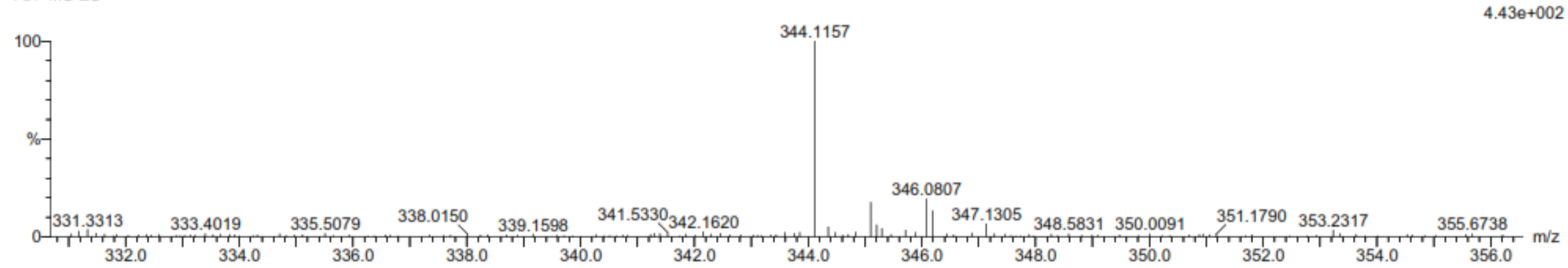

Minimum:				-1.5			
Maximum:		5.0	5.0	50.0			
Mass	Calc. Mass	mDa	PPM	DBE	i-FIT	i-FIT (Norm)	Formula
344.1157	344.1166	-0.9	-2.6	10.5	82.8	0.0	C18 H19 N3 O2 Cl

Spectrum ^{1}H NMR (500 MHz, DMSO-*d6*) of compound **8n**

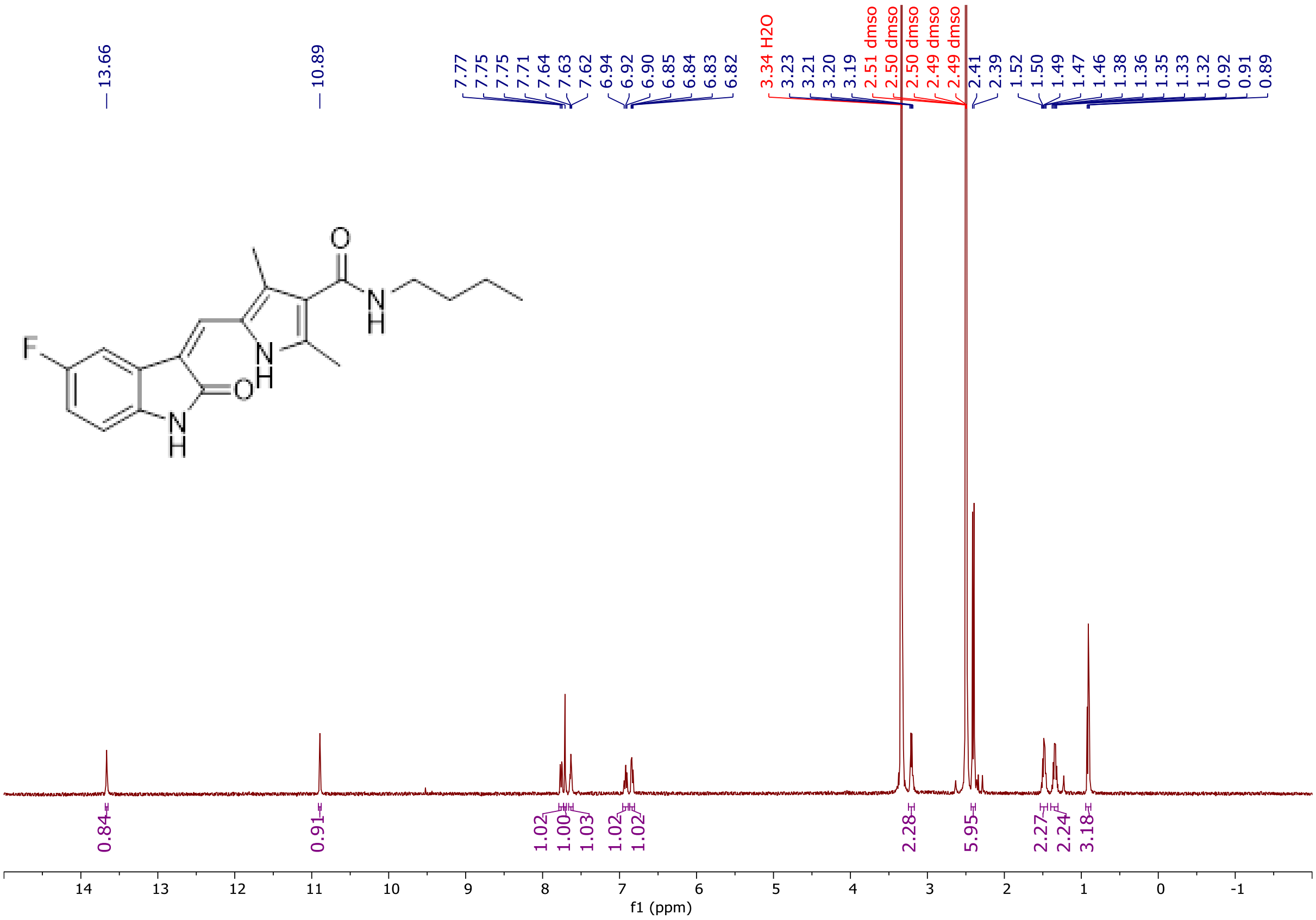

Spectrum ^{13}C NMR (125 MHz, DMSO-*d6*) of compound **8n**

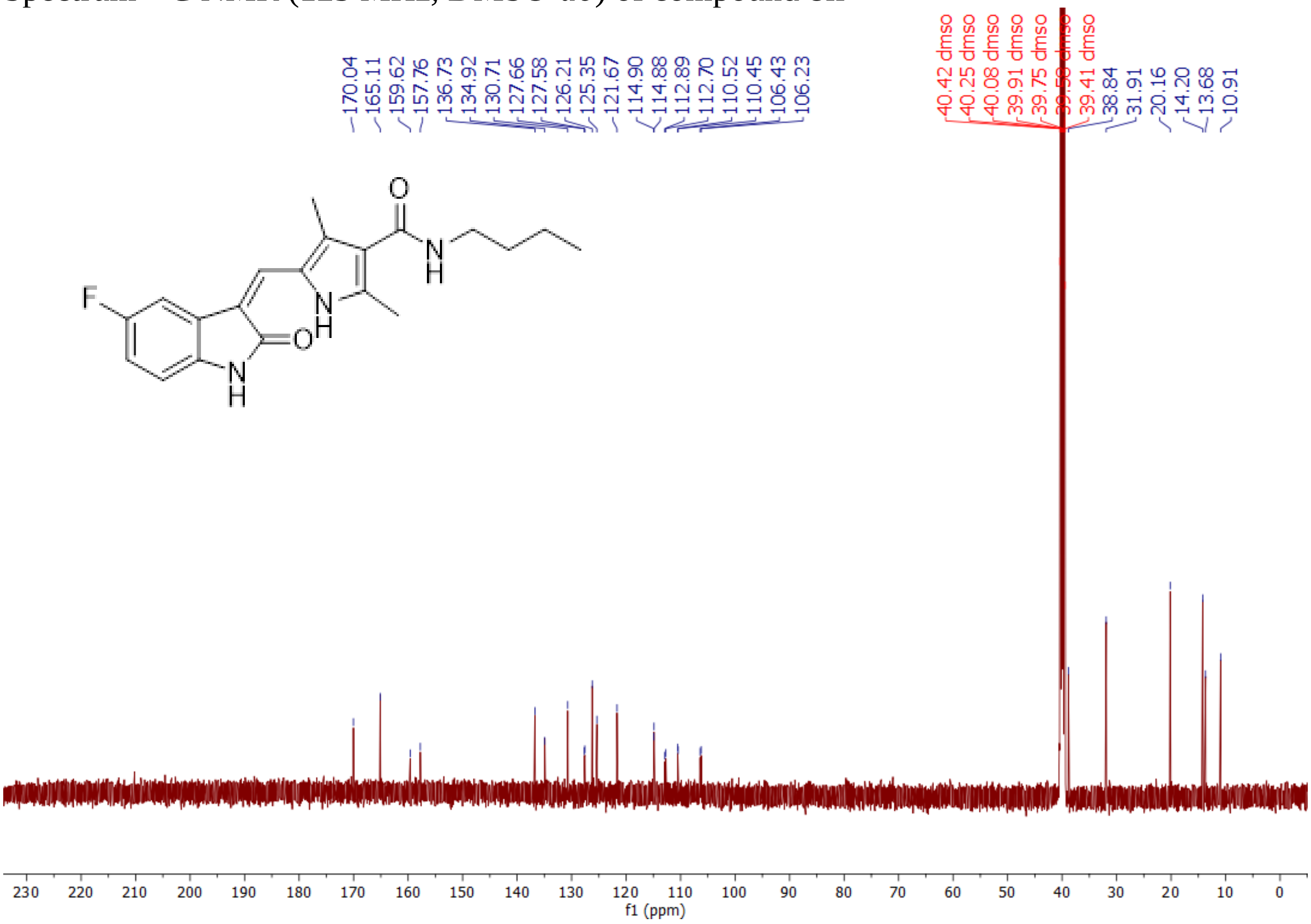

Spectrum ^{19}F NMR (470 MHz, DMSO-d_6) of compound **8n**

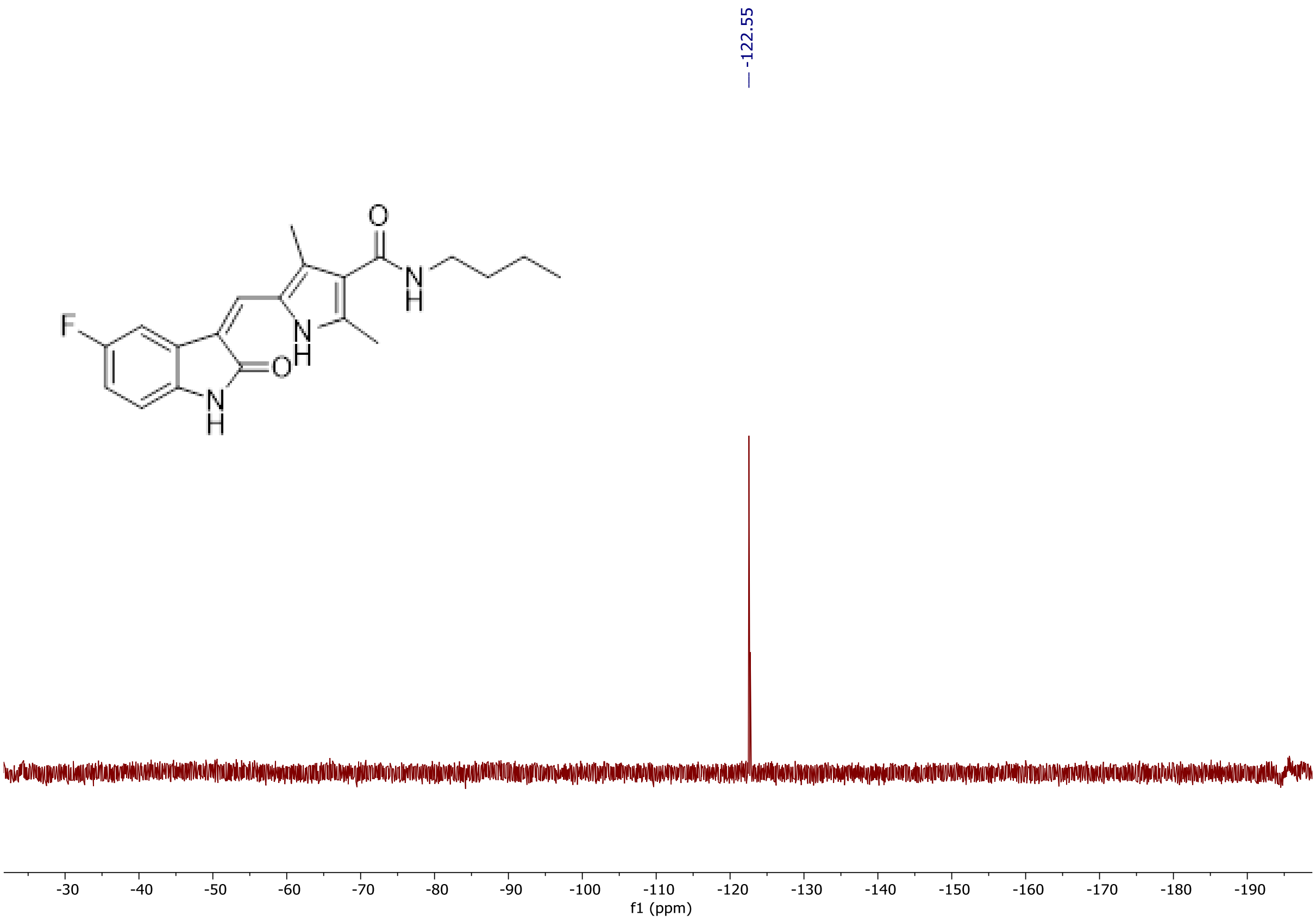

HRMS spectrum of compound **8n**

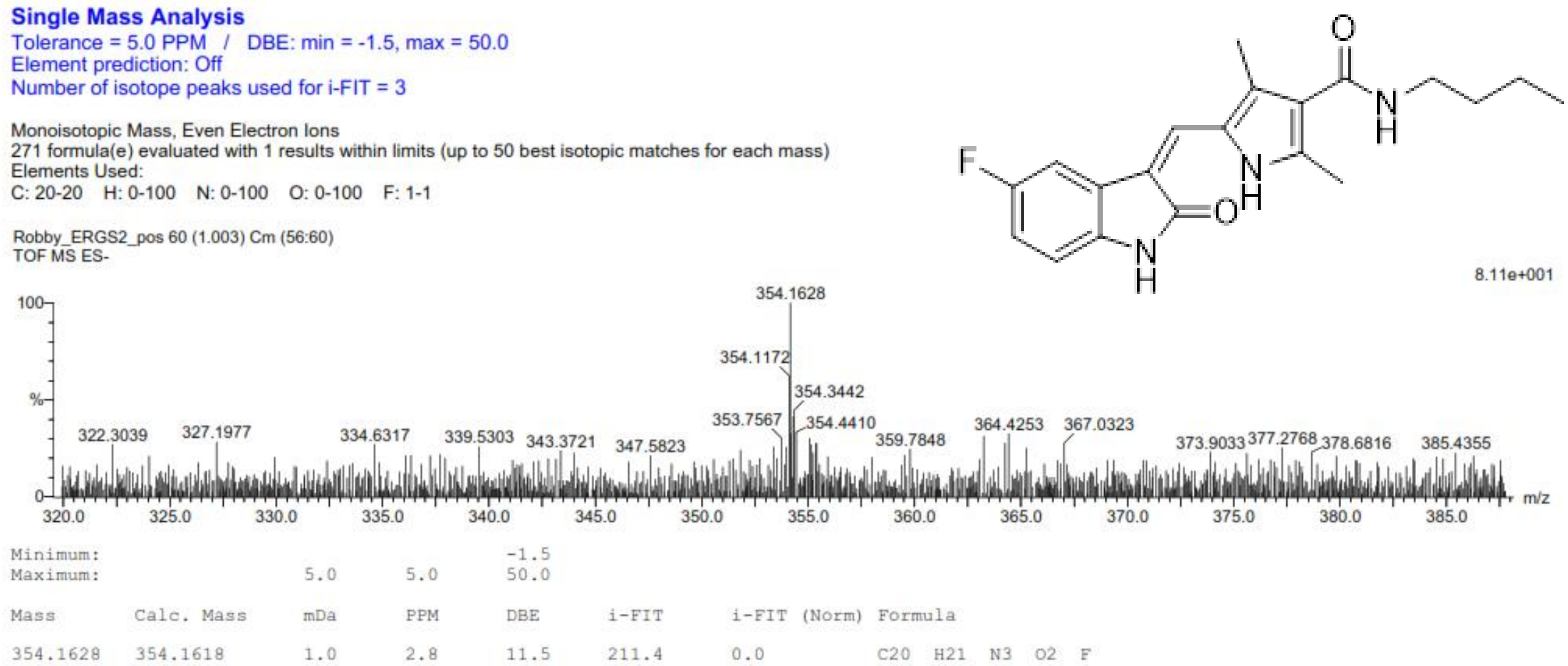

Spectrum ^{1}H NMR (500 MHz, DMSO-*d6*) of compound **8o**

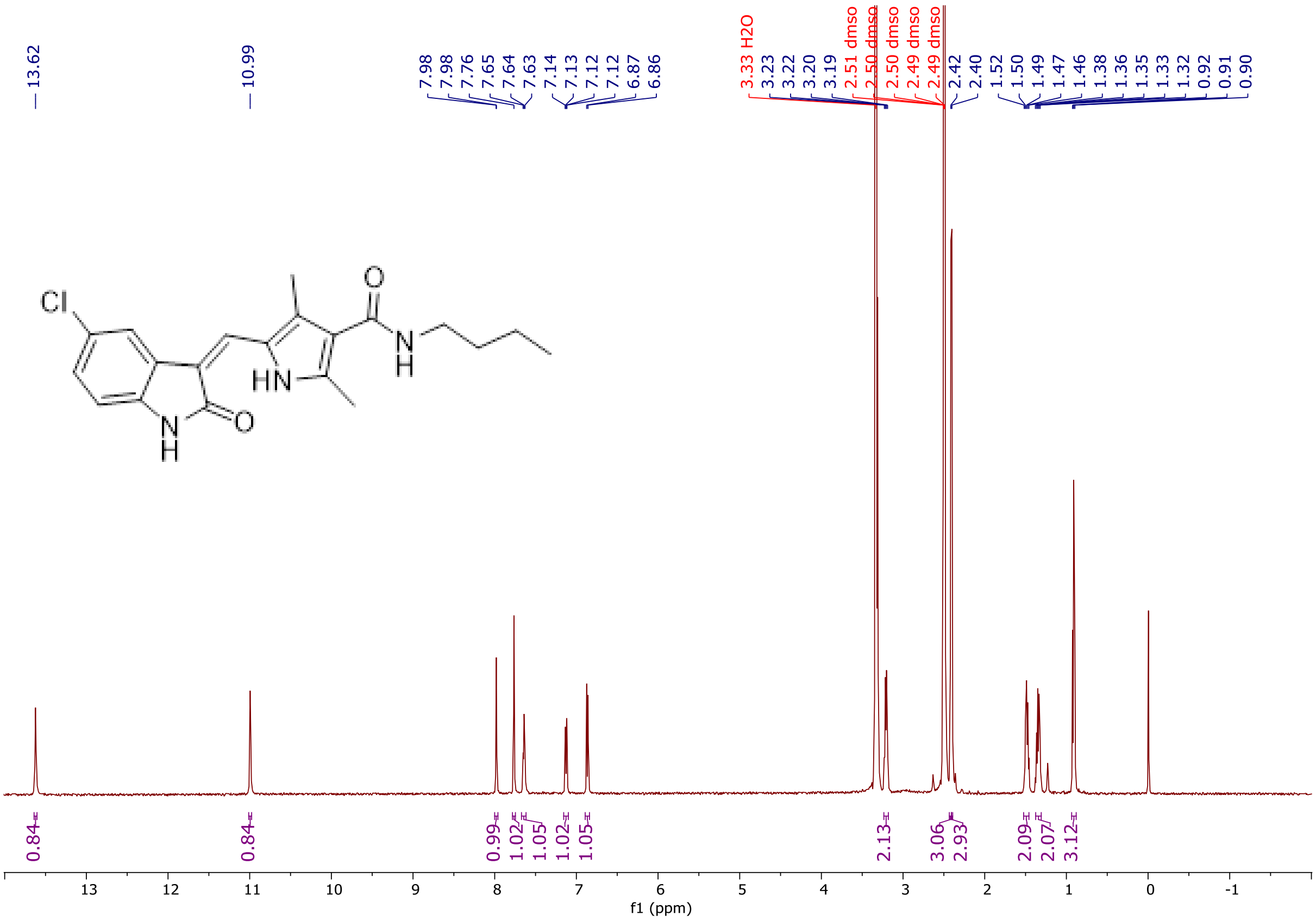

Spectrum ^{13}C NMR (125 MHz, DMSO-*d6*) of compound **8o**

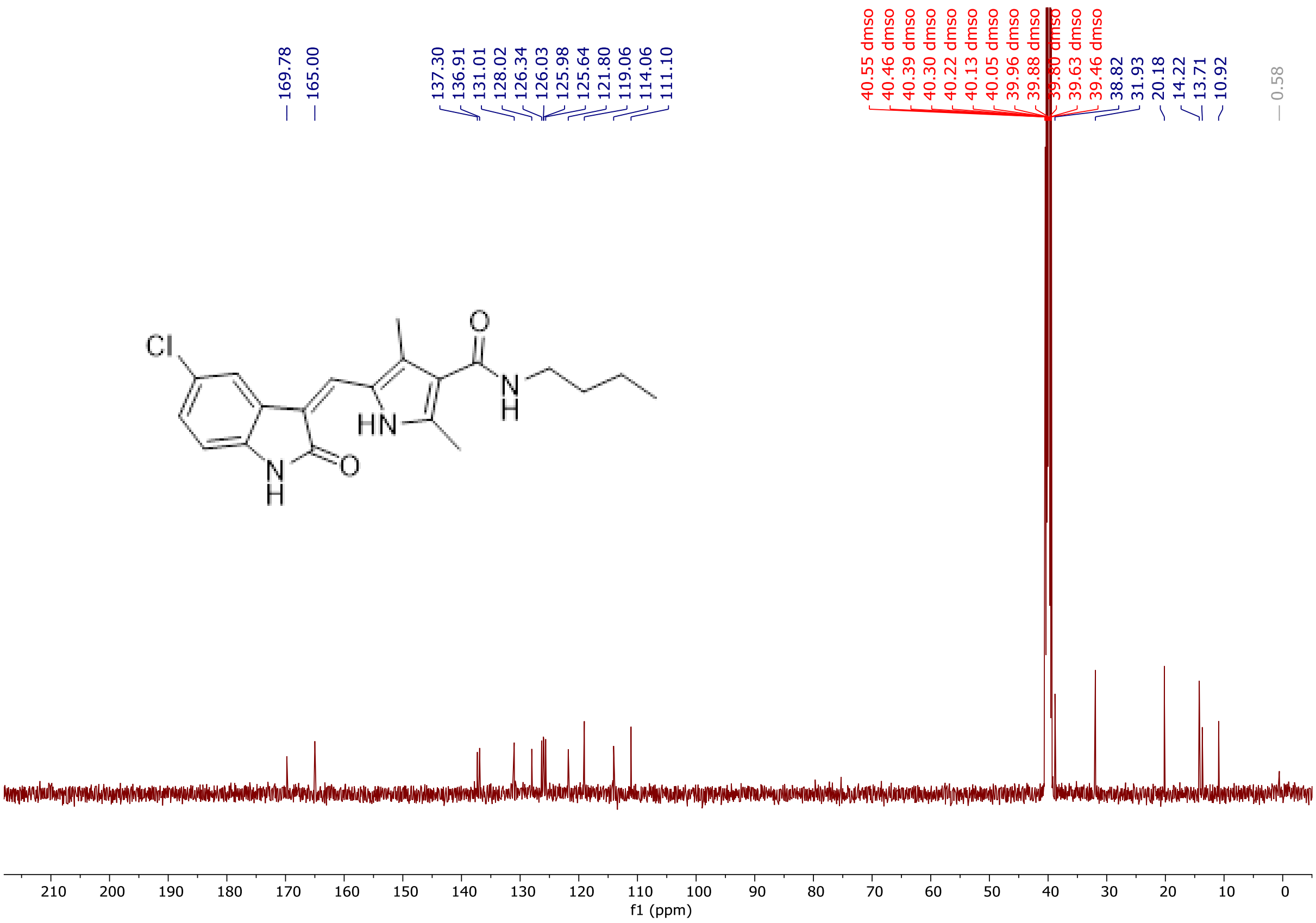

HRMS spectrum of compound **8o**

Single Mass Analysis
Tolerance = 5.0 PPM / DBE: min = -1.5, max = 50.0
Element prediction: Off
Number of isotope peaks used for i-FIT = 3

Monoisotopic Mass, Even Electron Ions
275 formula(e) evaluated with 1 results within limits (up to 50 best isotopic matches for each mass)
Elements Used:
C: 20-20 H: 0-100 N: 0-100 O: 0-100 Cl: 1-1
Robby_RGC6_pos 15 (0.239)
TOF MS ES+

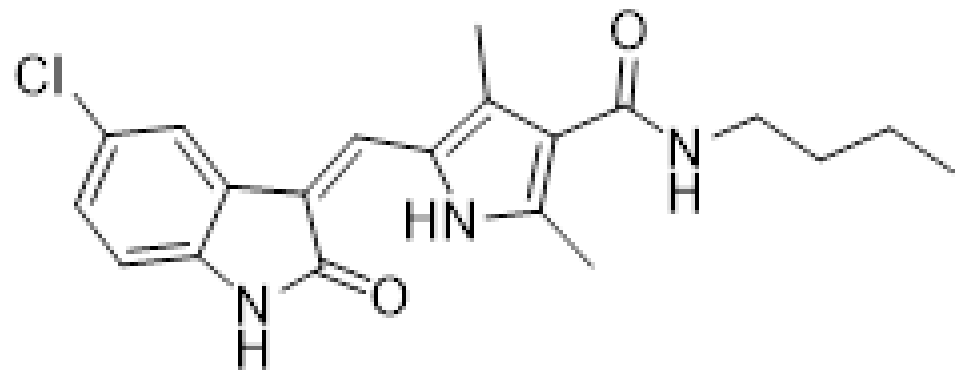

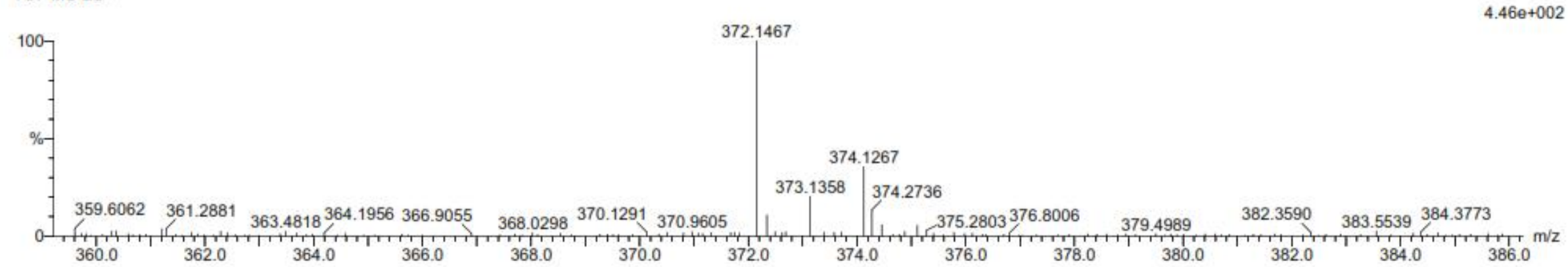

Mass	Calc. Mass	mDa	PPM	DBE	i-FIT	i-FIT (Norm)	Formula
Minimum:				-1.5			
Maximum:		5.0	5.0	50.0			
372.1467	372.1479	-1.2	-3.2	10.5	64.6	0.0	C20 H23 N3 O2 Cl

Spectrum ^{1}H NMR (500 MHz, DMSO-*d6*) of compound **8p**

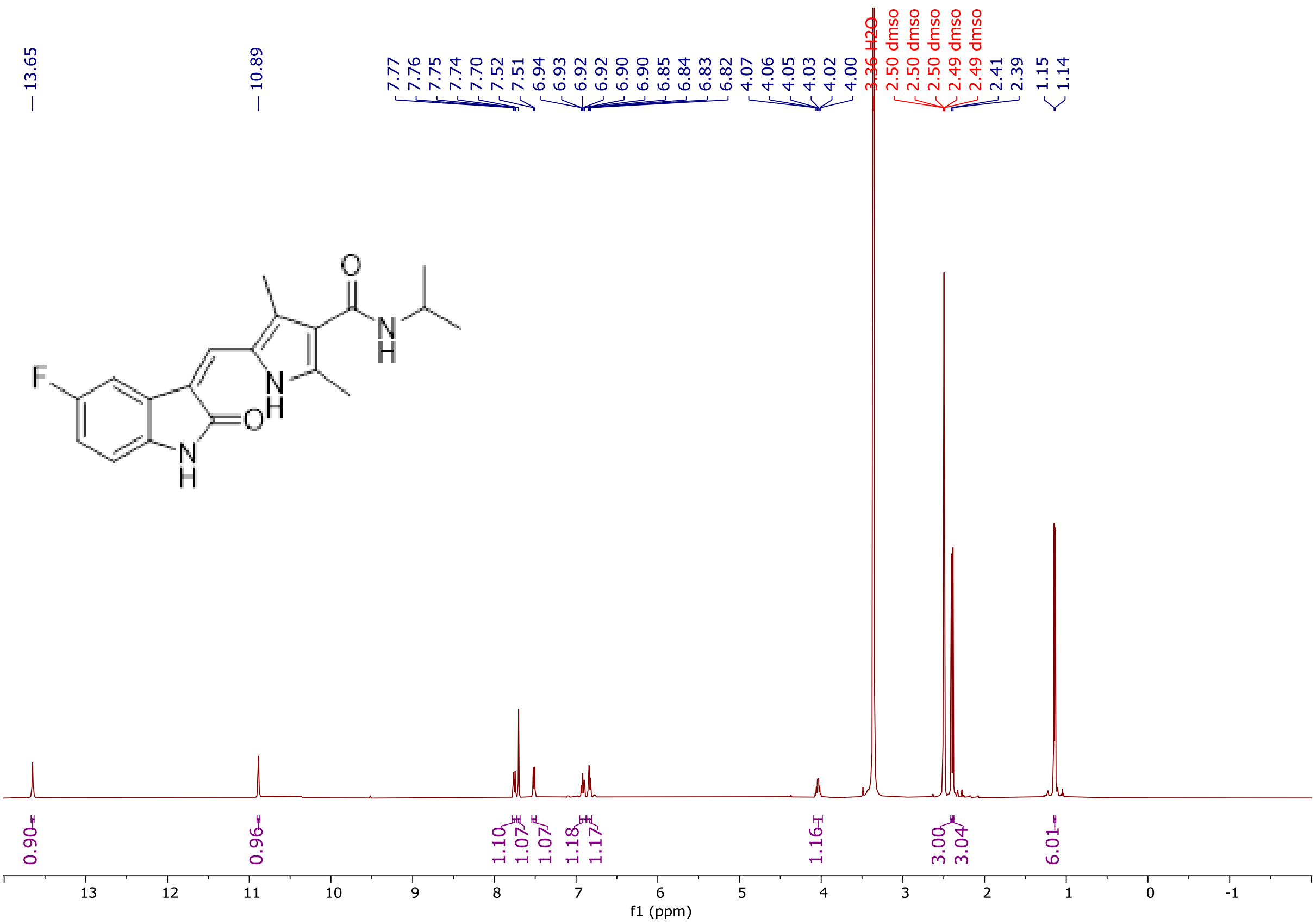

Spectrum ^{13}C NMR (125 MHz, DMSO-*d6*) of compound **8p**

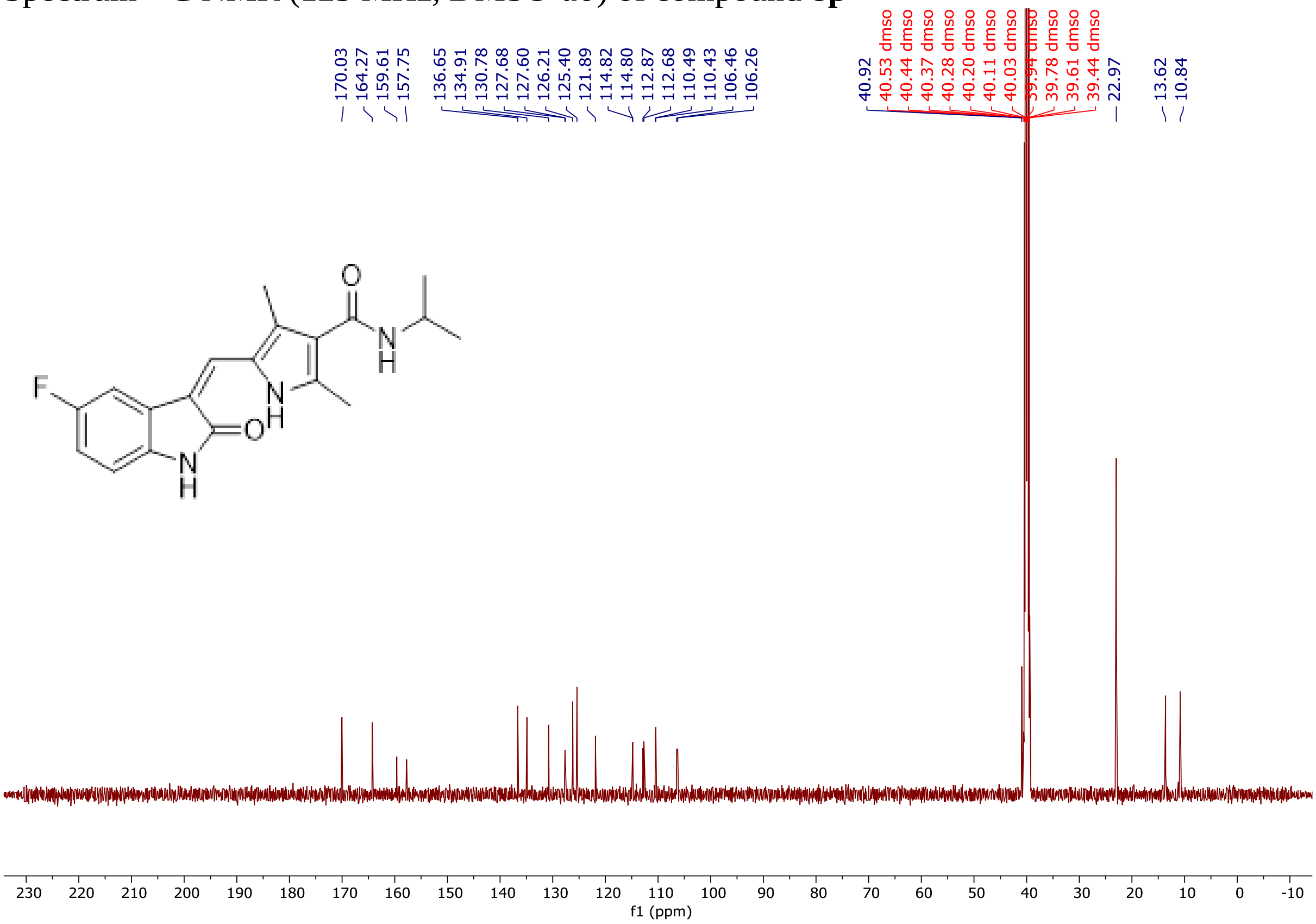

Spectrum ^{19}F NMR (470 MHz, DMSO-d_6) of compound **8p**

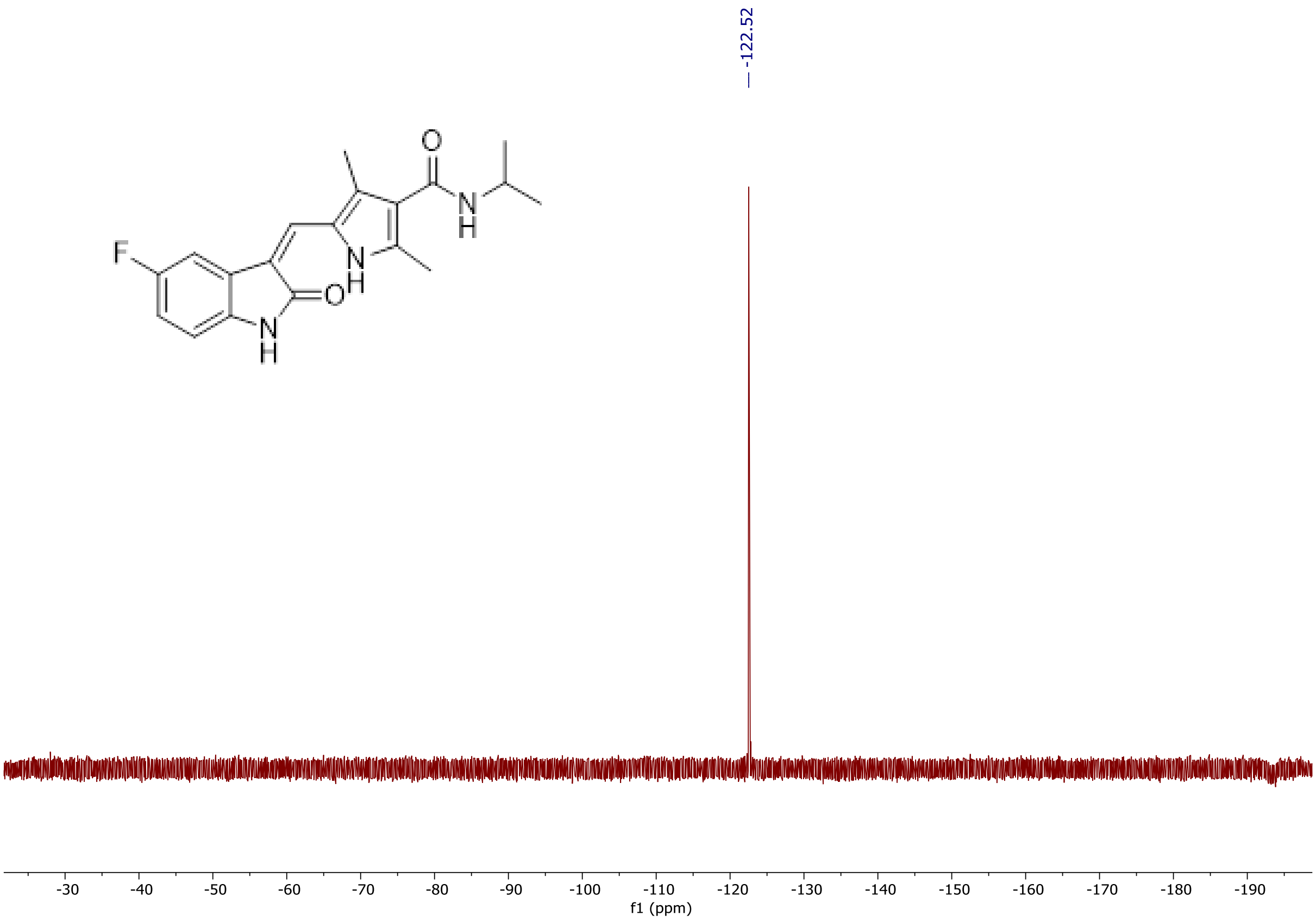

HRMS spectrum of compound **8p**

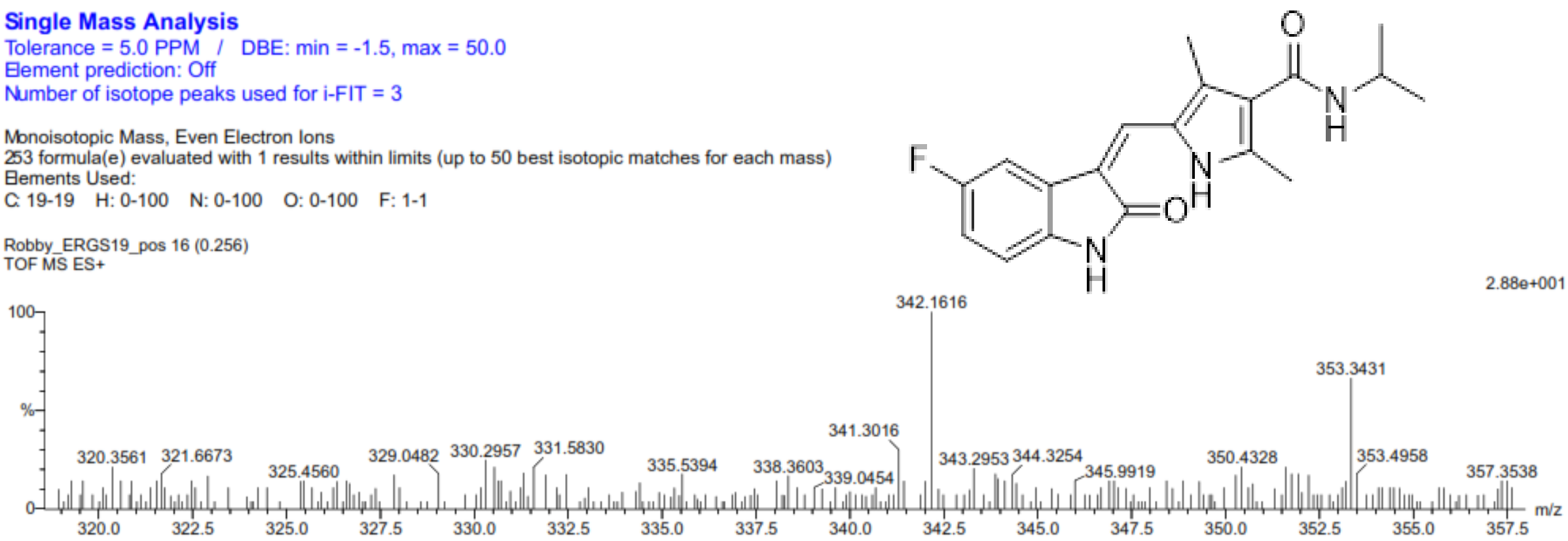

Minimum:				-1.5			
Maximum:		5.0	5.0	50.0			
Mass	Calc. Mass	mDa	PPM	DBE	i-FIT	i-FIT (Norm)	Formula
342.1616	342.1618	-0.2	-0.6	10.5	45.4	0.0	C19 H21 N3 O2 F

Spectrum ^{1}H NMR (500 MHz, DMSO-*d6*) of compound **8q**

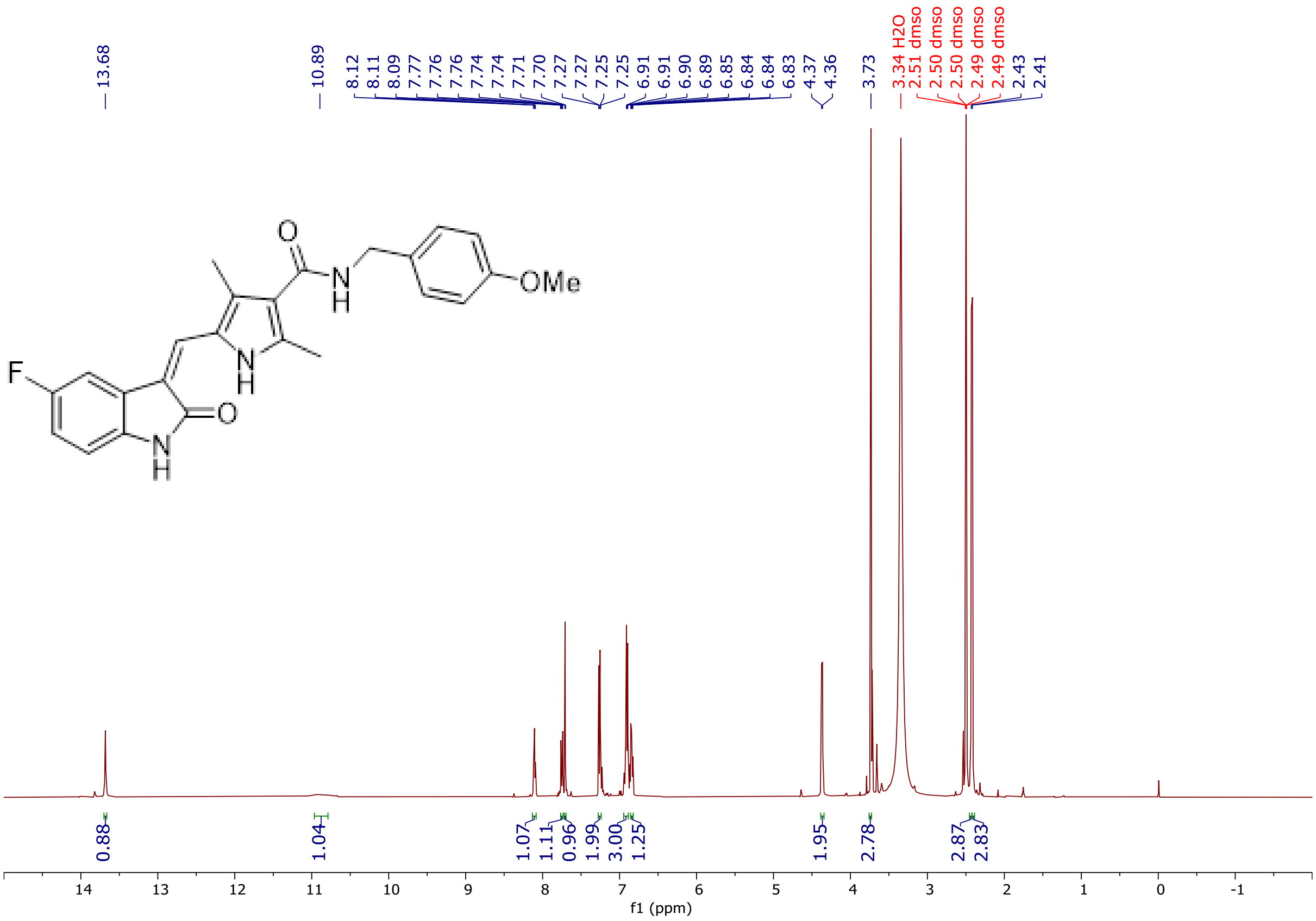

Spectrum ^{13}C NMR (125 MHz, DMSO-*d6*) of compound **8q**

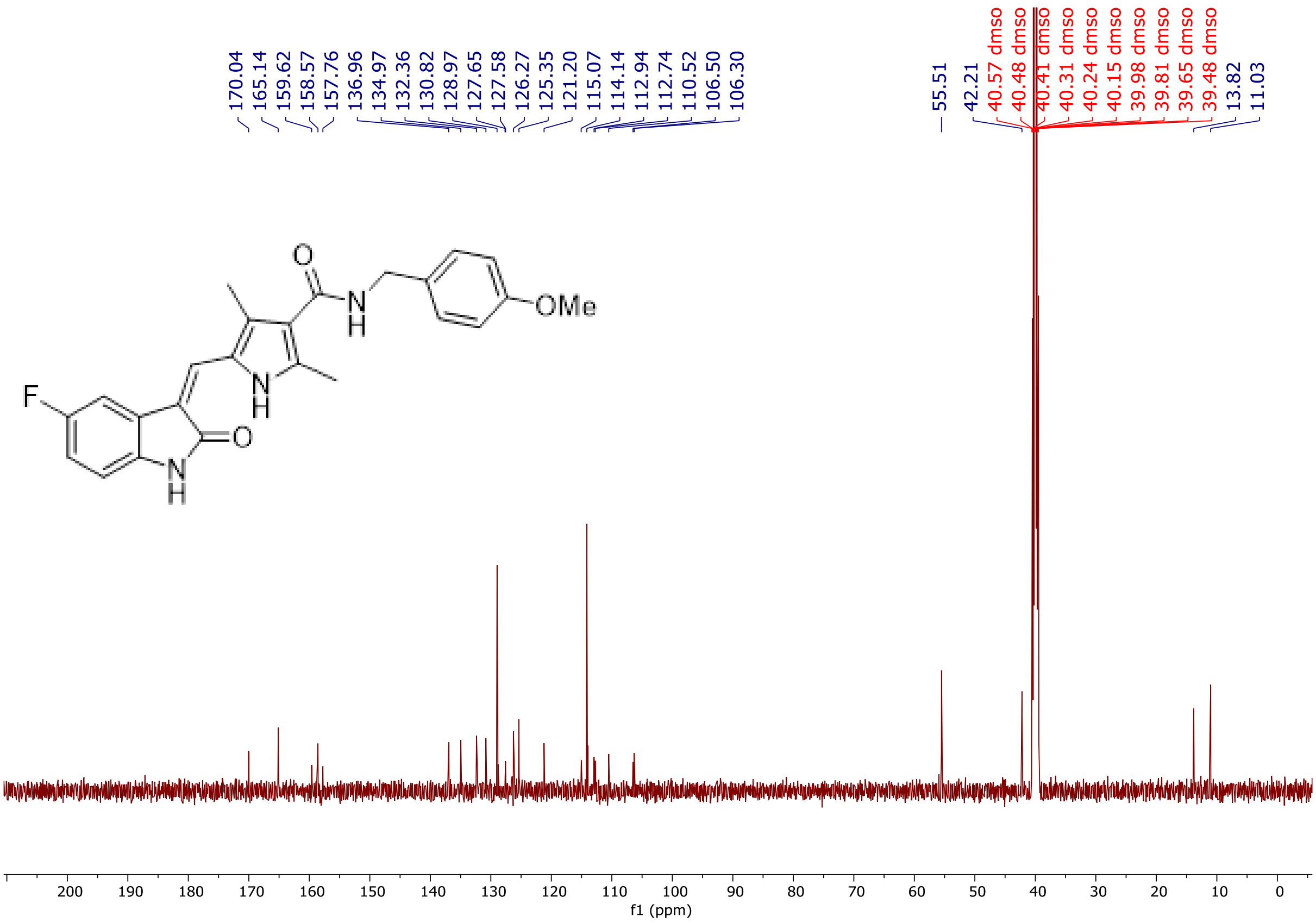

Spectrum ^{19}F NMR (470 MHz, DMSO-d_6) of compound **8q**

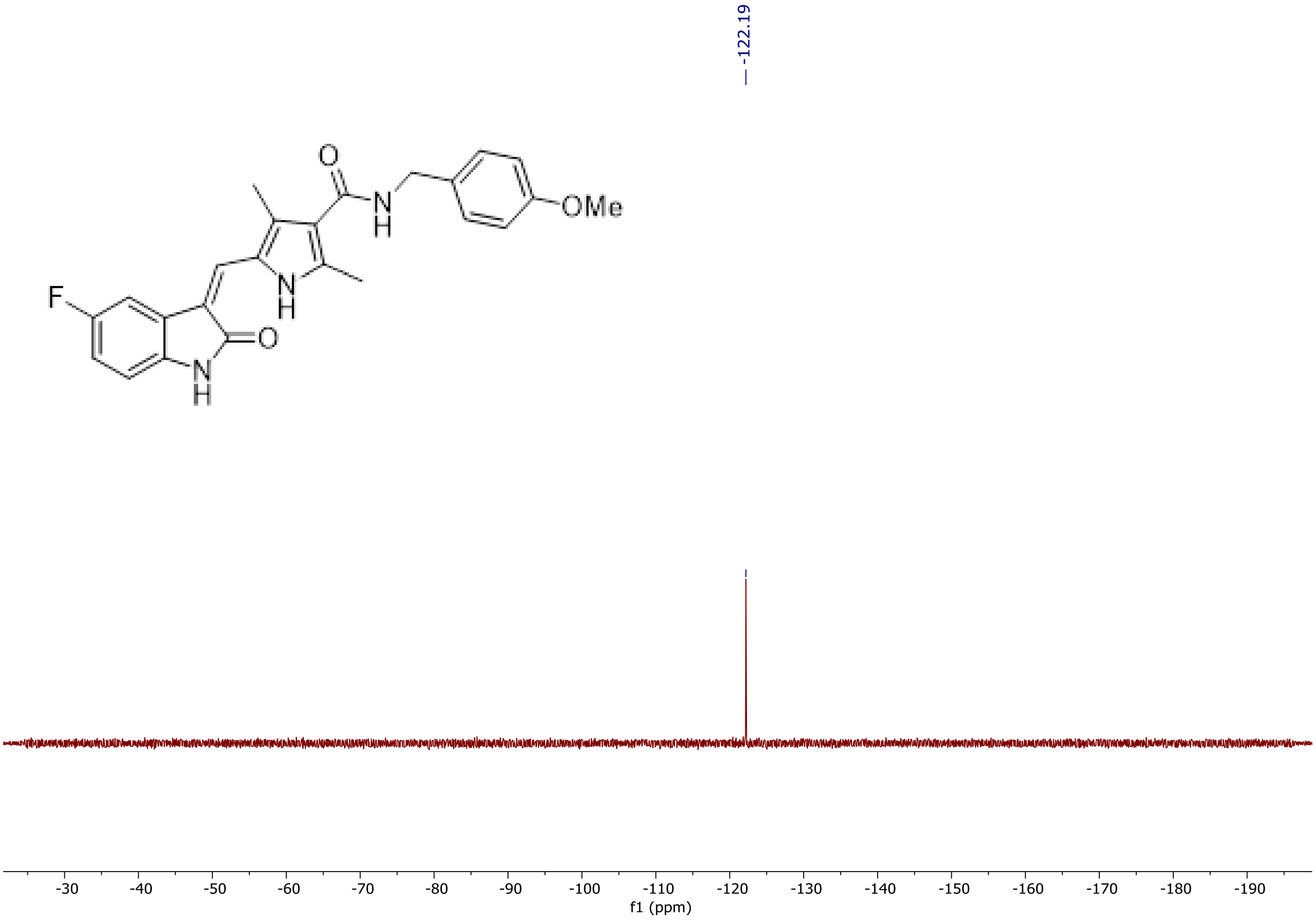

HRMS spectrum of compound **8q**

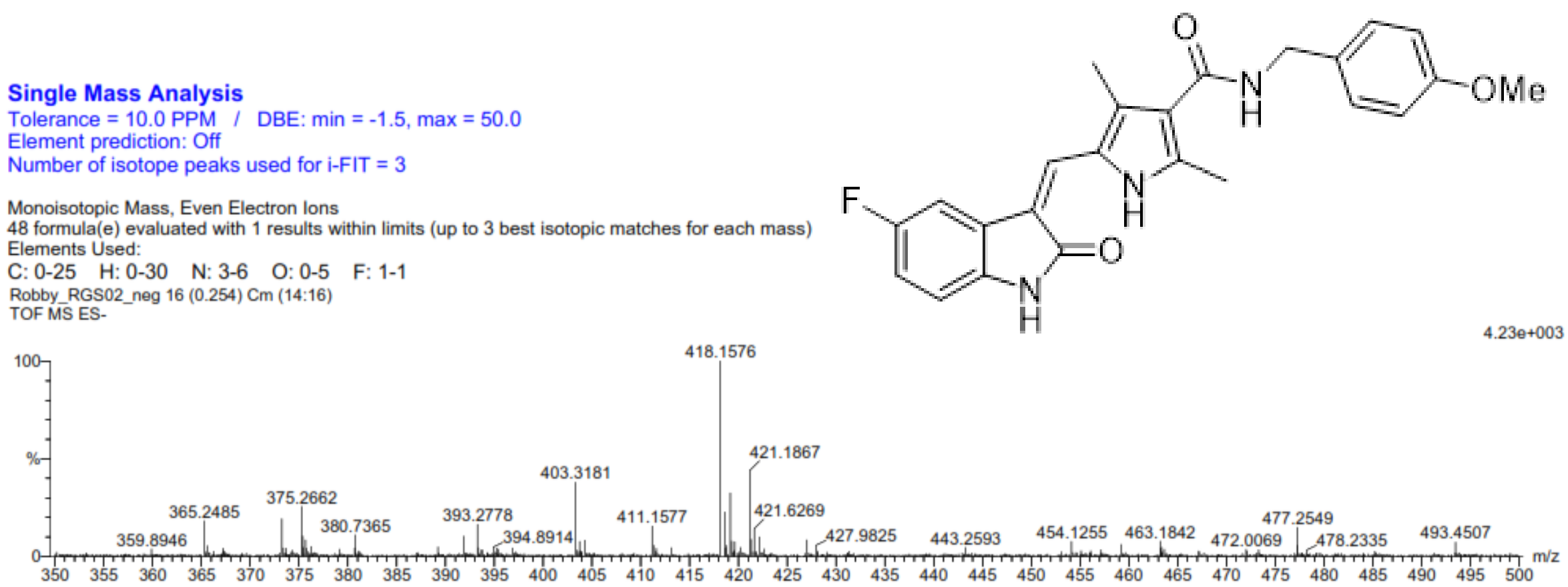

Minimum:				-1.5			
Maximum:		5.0	10.0	50.0			
Mass	Calc. Mass	mDa	PPM	DBE	i-FIT	i-FIT (Norm)	Formula
418.1576	418.1567	0.9	2.2	15.5	159.9	0.0	C24 H21 N3 O3 F

Spectrum ^{1}H NMR (500 MHz, DMSO-*d6*) of compound **8r**

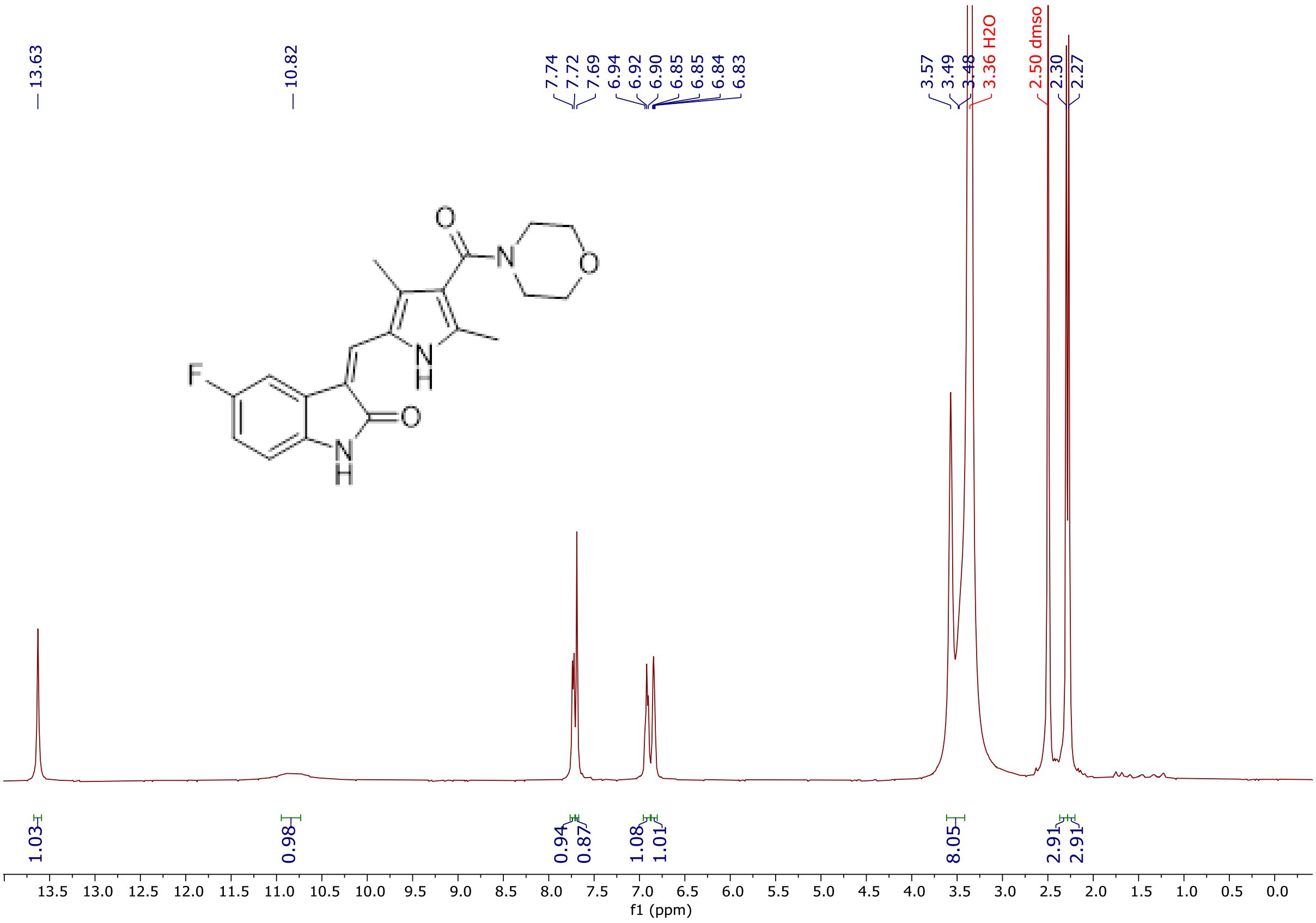

Spectrum ^{13}C NMR (125 MHz, DMSO-*d6*) of compound **8r**

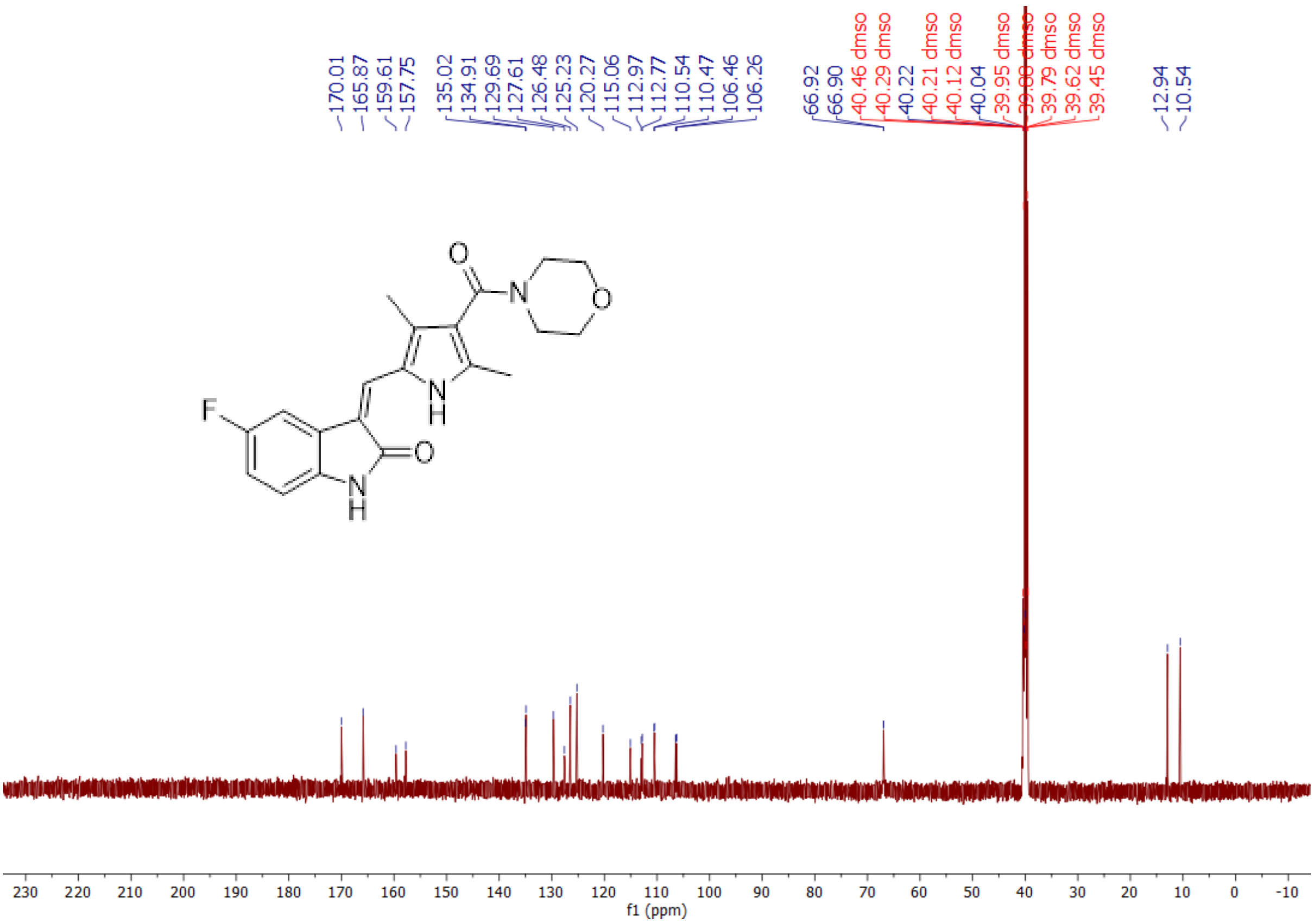

Spectrum ^{19}F NMR (470 MHz, DMSO-d_6) of compound **8r**

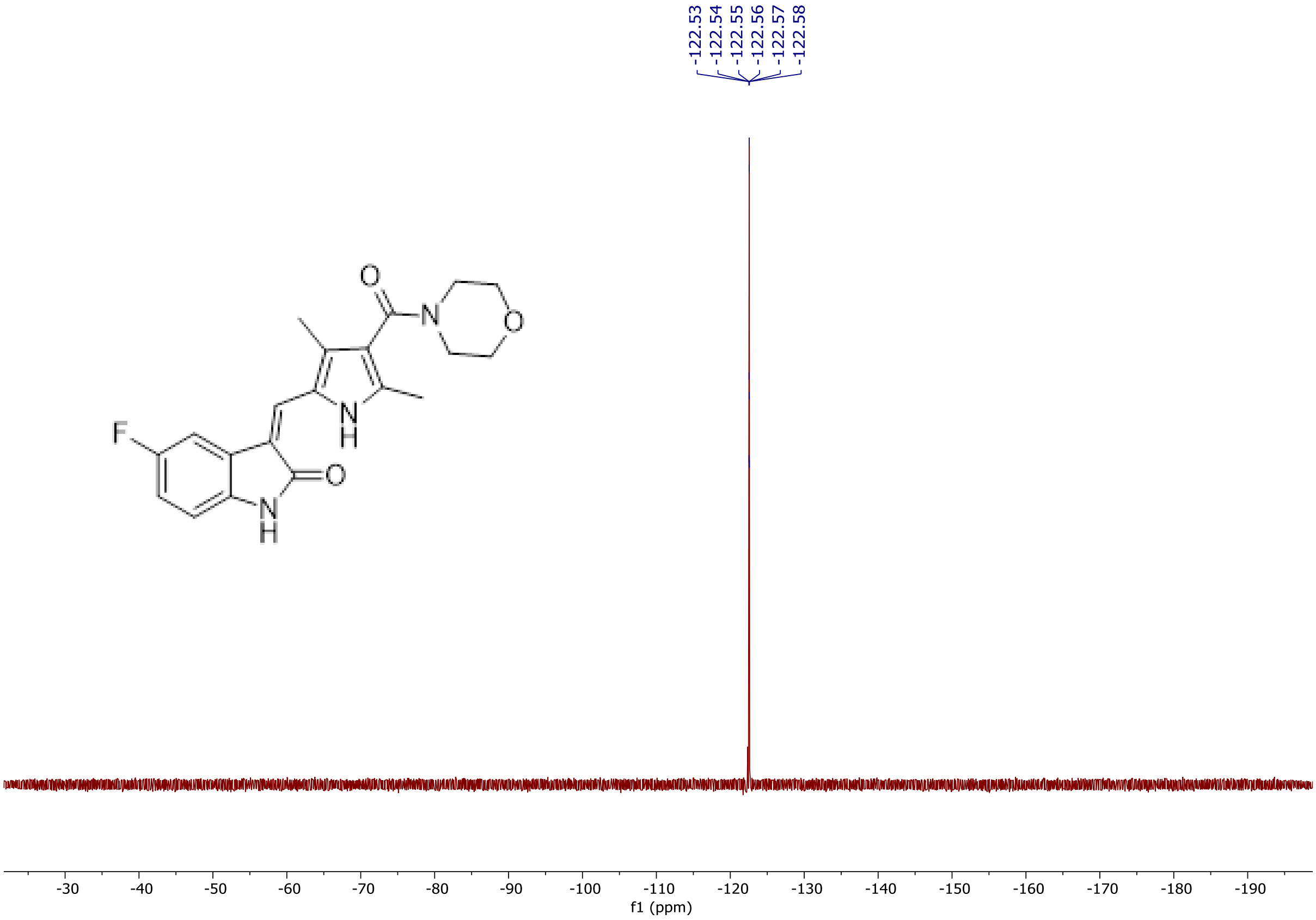

HRMS spectrum of compound **8r**

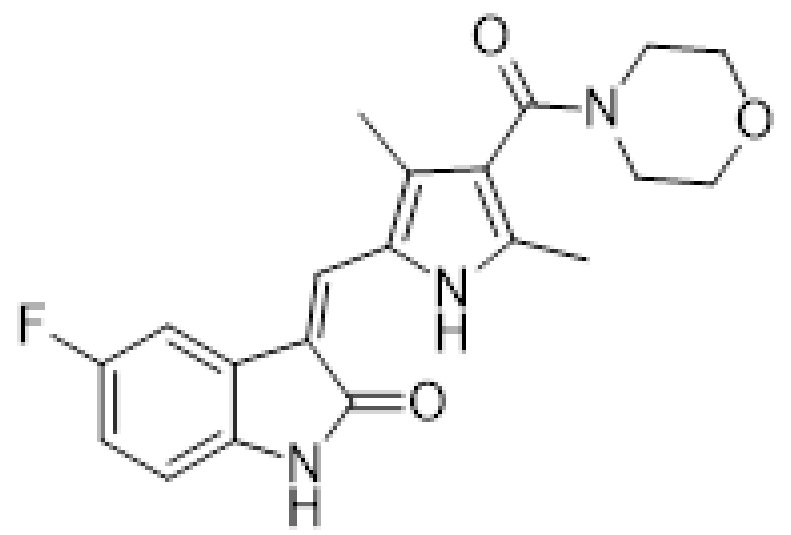

Single Mass Analysis
Tolerance = 5.0 PPM / DBE: min = -1.5, max = 50.0
Element prediction: Off
Number of isotope peaks used for i-FIT = 3

Monoisotopic Mass, Even Electron Ions
292 formula(e) evaluated with 1 results within limits (up to 50 best isotopic matches for each mass)
Elements Used:
C: 20-20 H: 0-100 N: 0-100 O: 0-100 F: 1-1

Robby_ERGS8_neg 33 (0.544) Cm (33:36)
TOF MS ES-

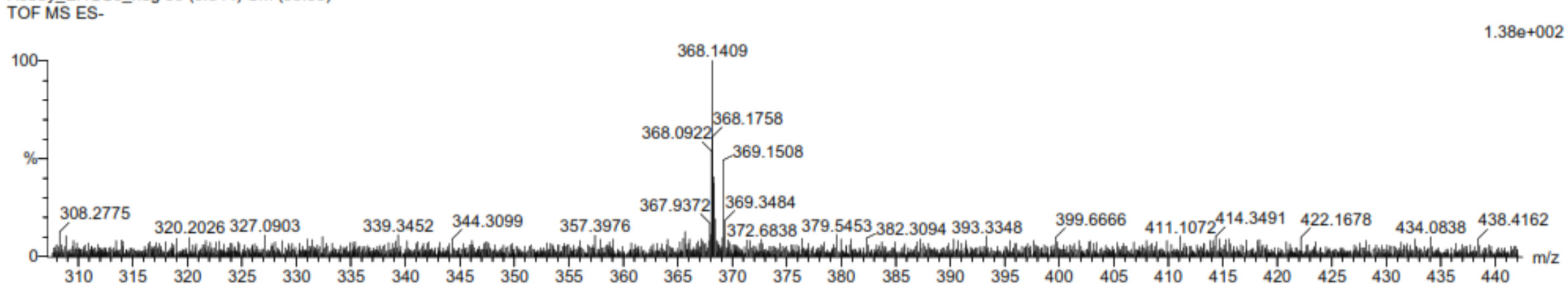

Minimum:				-1.5			
Maximum:		5.0	5.0	50.0			
Mass	Calc. Mass	mDa	PPM	DBE	i-FIT	i-FIT (Norm)	Formula
368.1409	368.1410	-0.1	-0.3	12.5	166.2	0.0	C20 H19 N3 O3 F

Spectrum ^{1}H NMR (500 MHz, DMSO-*d6*) of compound **8s**

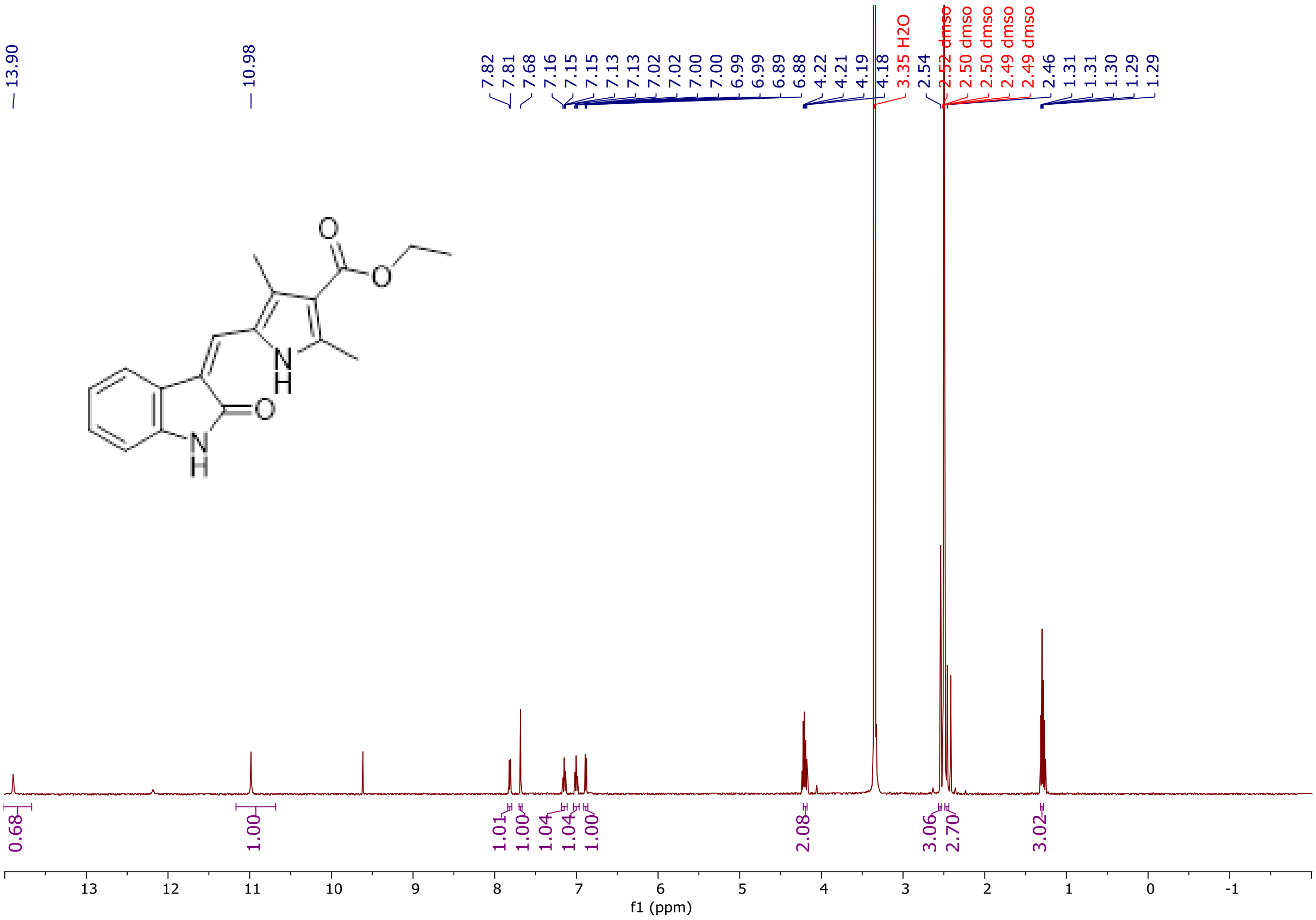

Spectrum ^{13}C NMR (125 MHz, DMSO-*d6*) of compound **8s**

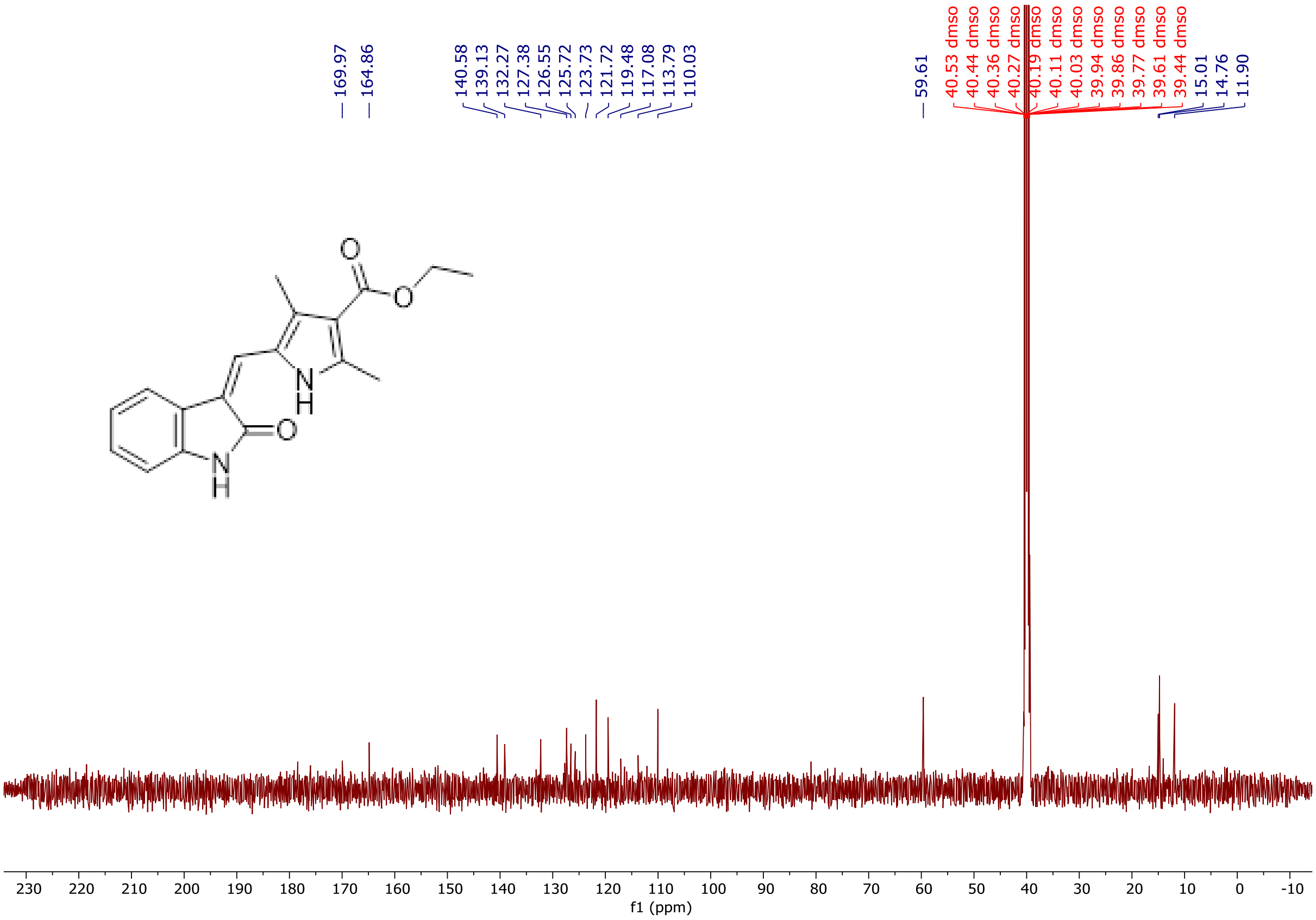

HRMS spectrum of compound **8s**

Single Mass Analysis
Tolerance = 5.0 PPM / DBE: min = -1.5, max = 50.0
Element prediction: Off
Number of isotope peaks used for i-FIT = 3

Monoisotopic Mass, Even Electron Ions
232 formula(e) evaluated with 1 results within limits (up to 50 best isotopic matches for each mass)
Elements Used:
C: 18-18 H: 0-100 N: 0-100 O: 0-100
Robby_ERI2_neg 3 (0.034) Cm (3:4)
TOF MS ES-

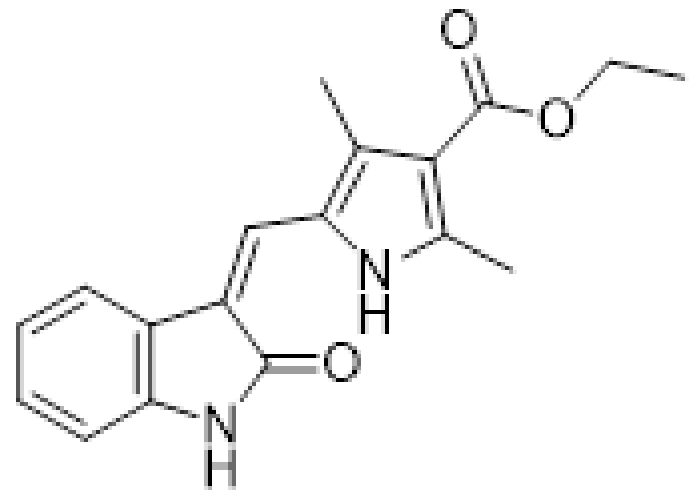

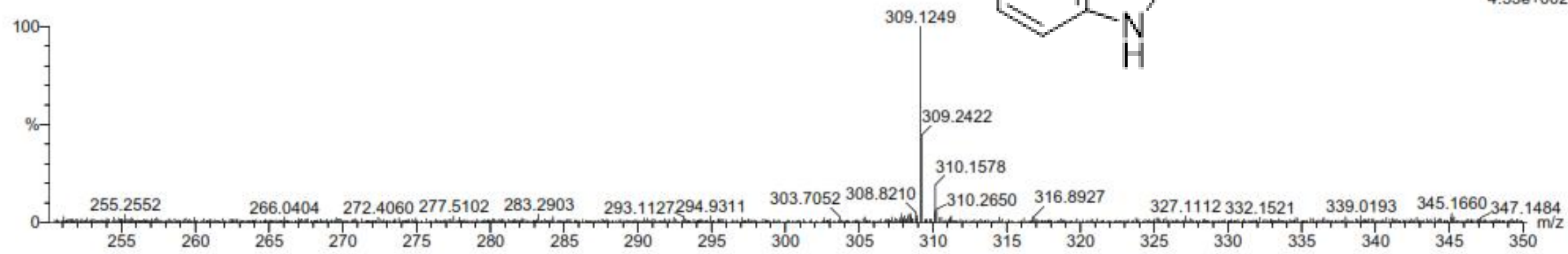

Minimum:				-1.5			
Maximum:		5.0	5.0	50.0			
Mass	Calc. Mass	mDa	PPM	DBE	i-FIT	i-FIT (Norm)	Formula
309.1249	309.1239	1.0	3.2	11.5	110.5	0.0	C18 H17 N2 O3

Spectrum ^{1}H NMR (500 MHz, DMSO-*d6*) of compound **4**

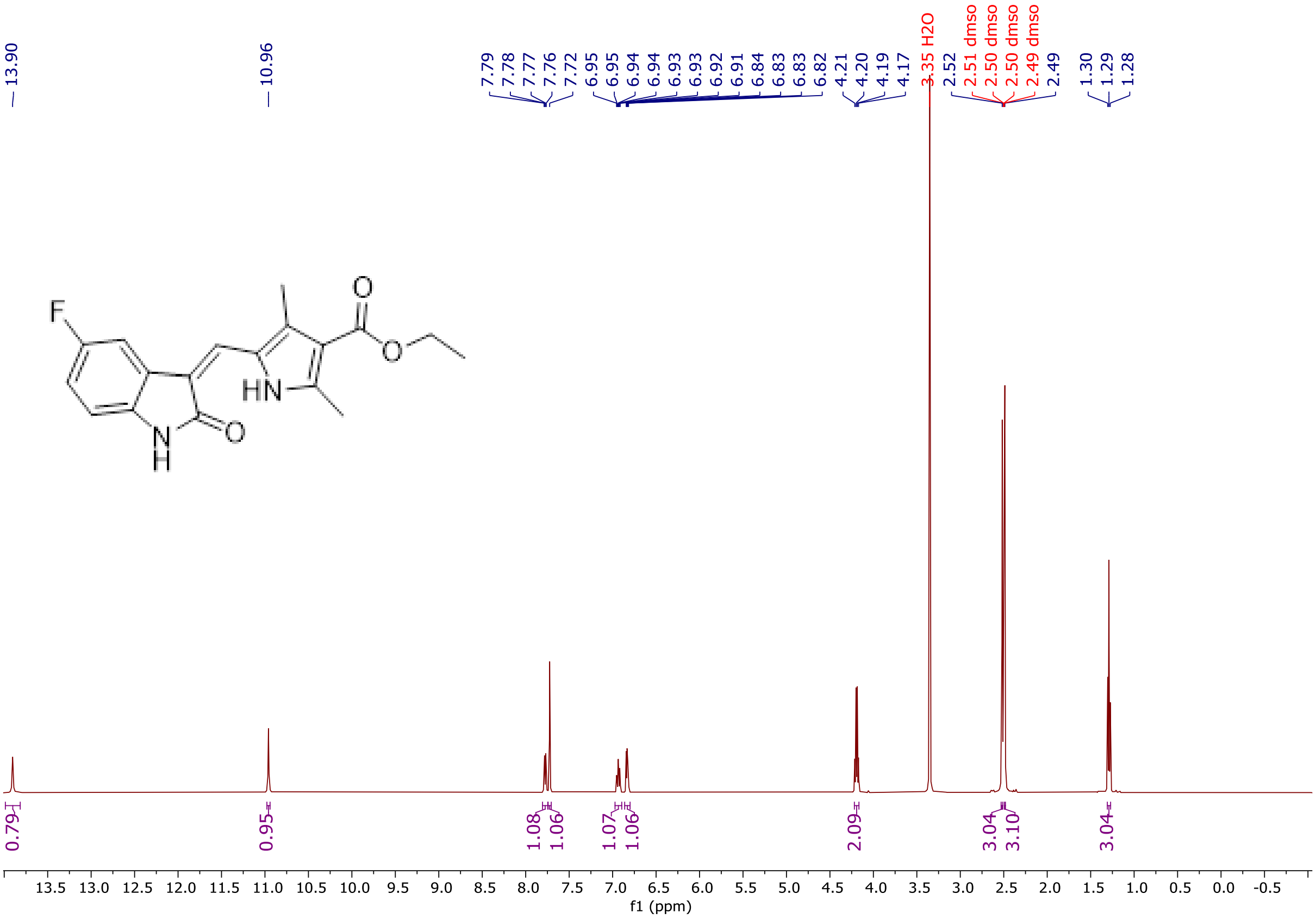

Spectrum ^{13}C NMR (125 MHz, DMSO-*d6*) of compound **4**

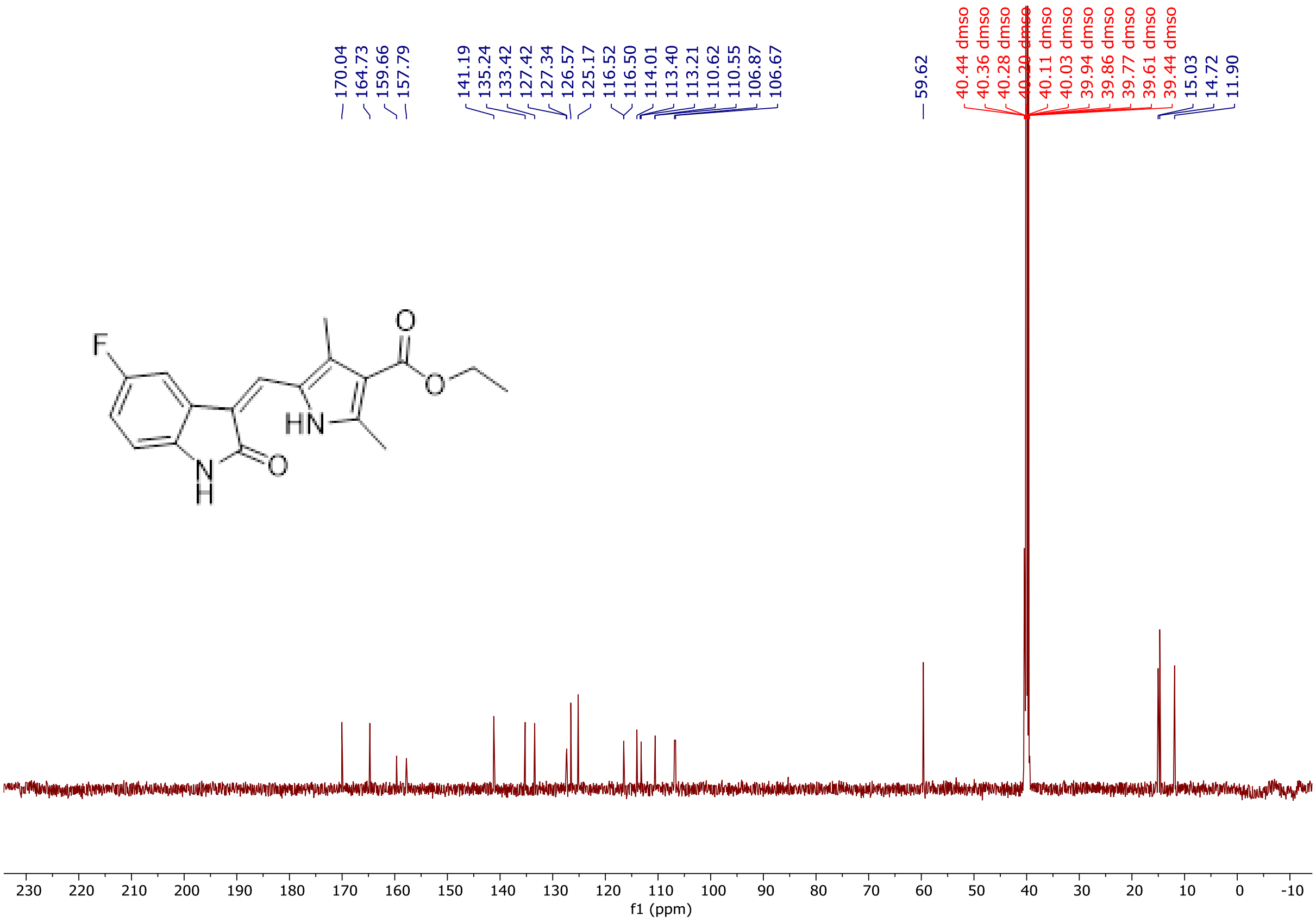

Spectrum ^{19}F NMR (470 MHz, DMSO-d_6) of compound **4**

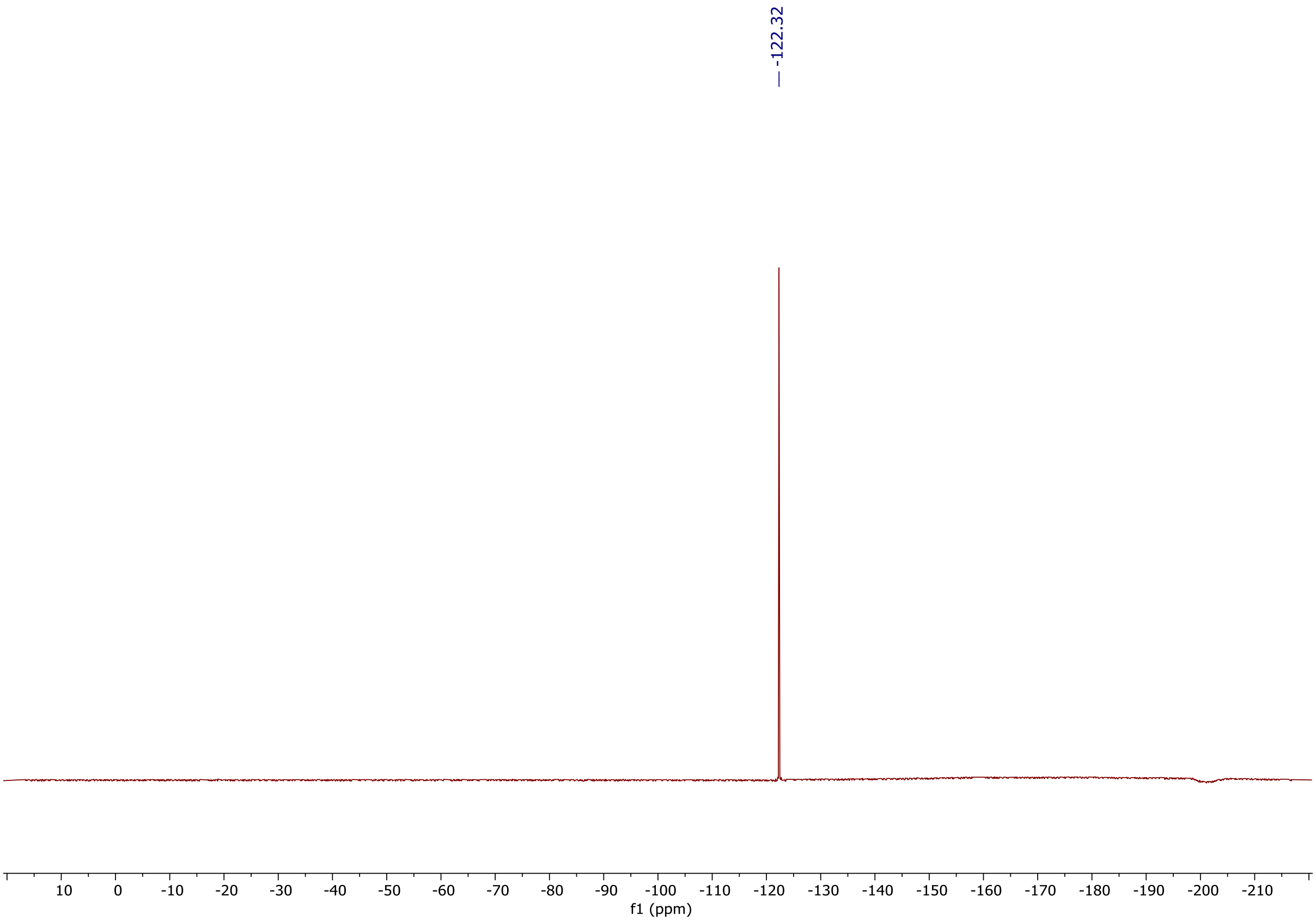

HRMS spectrum of compound **4**

Single Mass Analysis
Tolerance = 5.0 PPM / DBE: min = -1.5, max = 50.0
Element prediction: Off
Number of isotope peaks used for i-FIT = 3

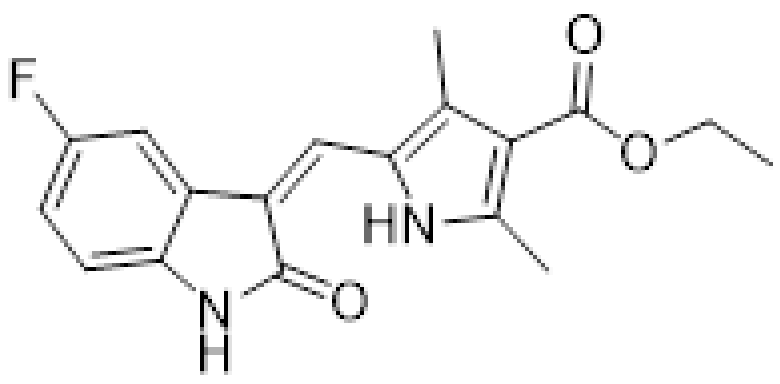

Monoisotopic Mass, Even Electron Ions
234 formula(e) evaluated with 1 results within limits (up to 50 best isotopic matches for each mass)
Elements Used:
C: 18-18 H: 0-100 N: 0-100 O: 0-100 F: 1-1

Robby_ERGS1_pos 73 (1.229)
TOF MS ES+

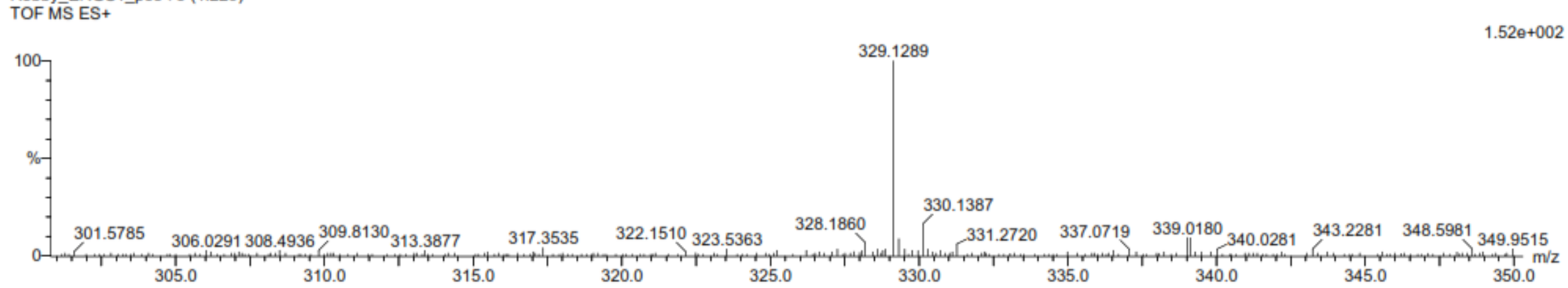

	Calc. Mass	mDa	PPM	DBE	i-FIT	i-FIT (Norm)	Formula
Minimum:				-1.5			
Maximum:		5.0	5.0	50.0			
**Mass**							
329.1289	329.1301	-1.2	-3.6	10.5	47.8	0.0	C18 H18 N2 O3 F

Spectrum ^{1}H NMR (500 MHz, DMSO-*d6*) of compound **8t**

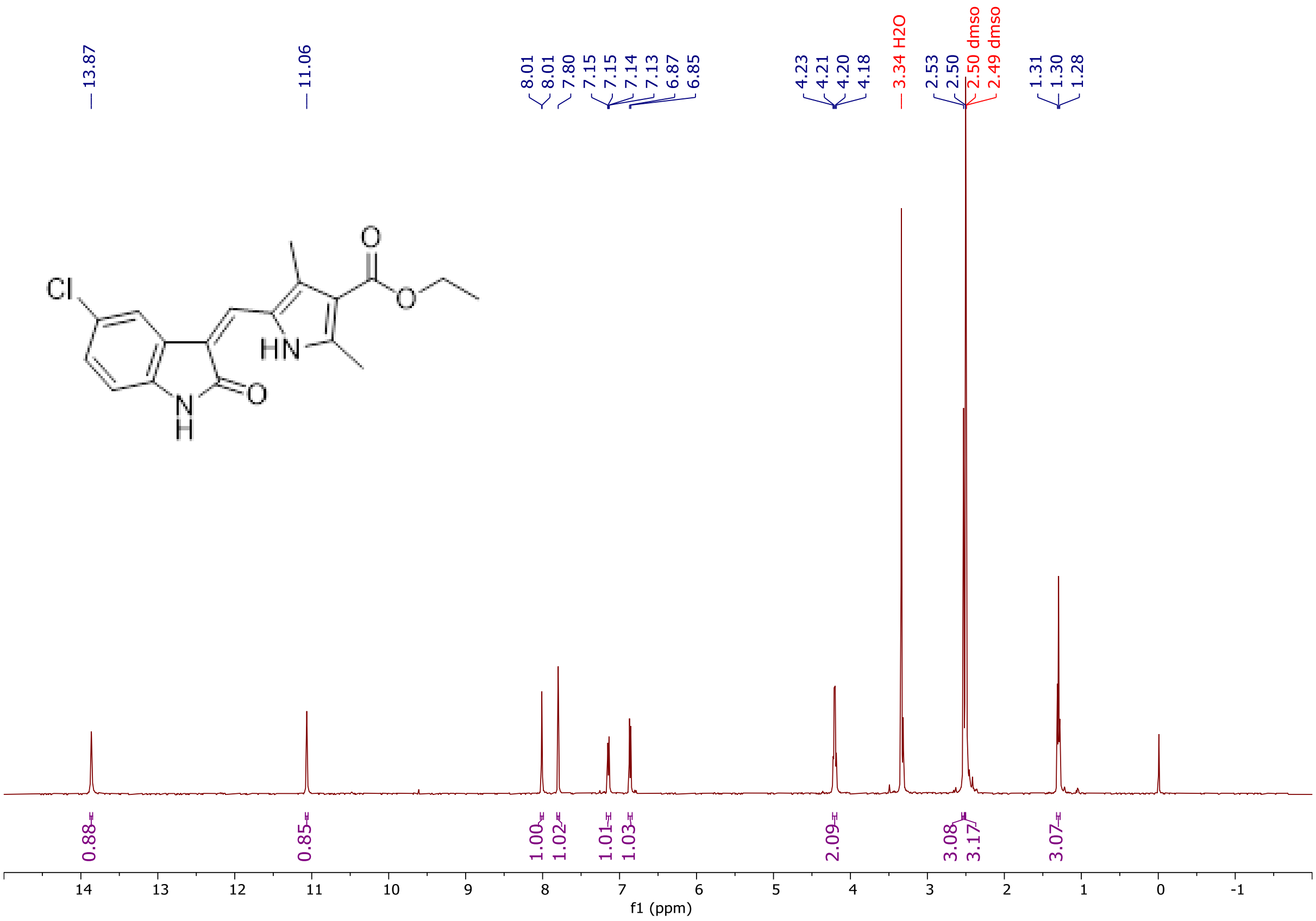

Spectrum ^{13}C NMR (125 MHz, DMSO-*d6*) of compound **8t**

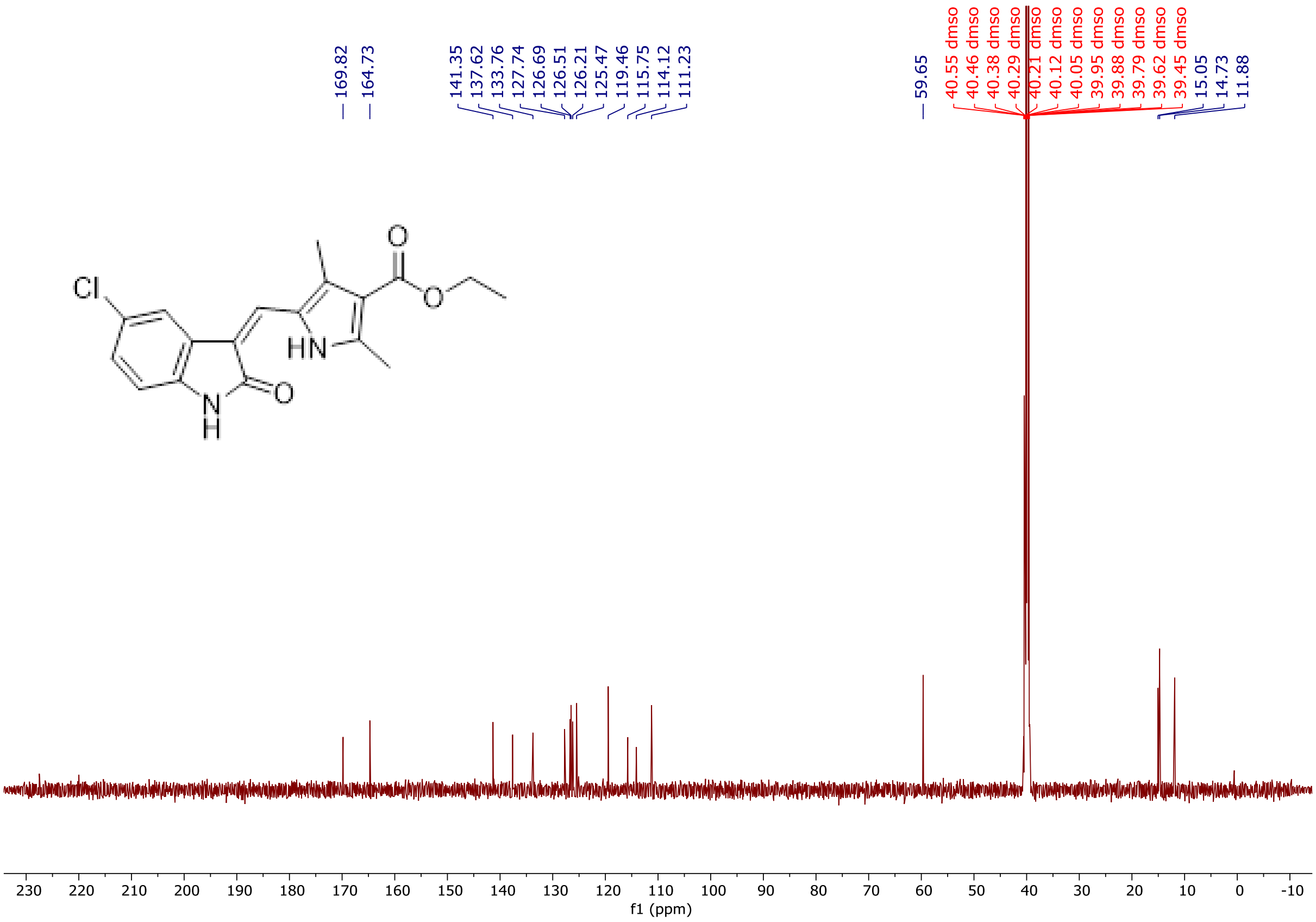

HRMS spectrum of compound **8t**

Single Mass Analysis
Tolerance = 5.0 PPM / DBE: min = -1.5, max = 50.0
Element prediction: Off
Number of isotope peaks used for i-FIT = 3

Monoisotopic Mass, Even Electron Ions
234 formula(e) evaluated with 1 results within limits (up to 50 best isotopic matches for each mass)
Elements Used:
C: 18-18 H: 0-100 N: 0-100 O: 0-100 Cl: 1-1
Robby_RGC2_pos 10 (0.154) Cm (10:12)
TOF MS ES+

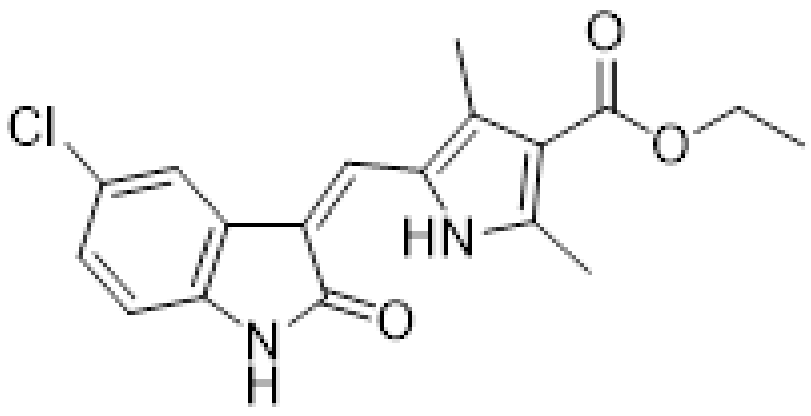

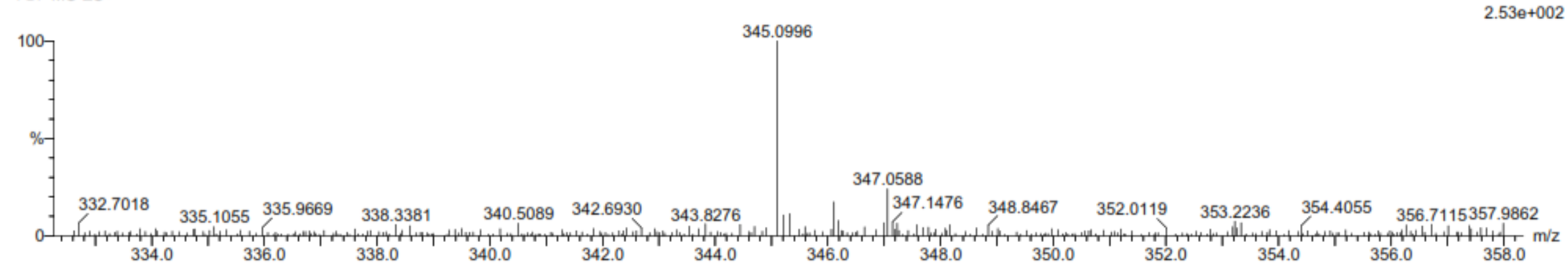

Minimum:				-1.5			
Maximum:		5.0	5.0	50.0			
Mass	Calc. Mass	mDa	PPM	DBE	i-FIT	i-FIT (Norm)	Formula
345.0996	345.1006	-1.0	-2.9	10.5	135.3	0.0	C18 H18 N2 O3 Cl

Spectrum ^{1}H NMR (500 MHz, DMSO-*d6*) of compound **8u**

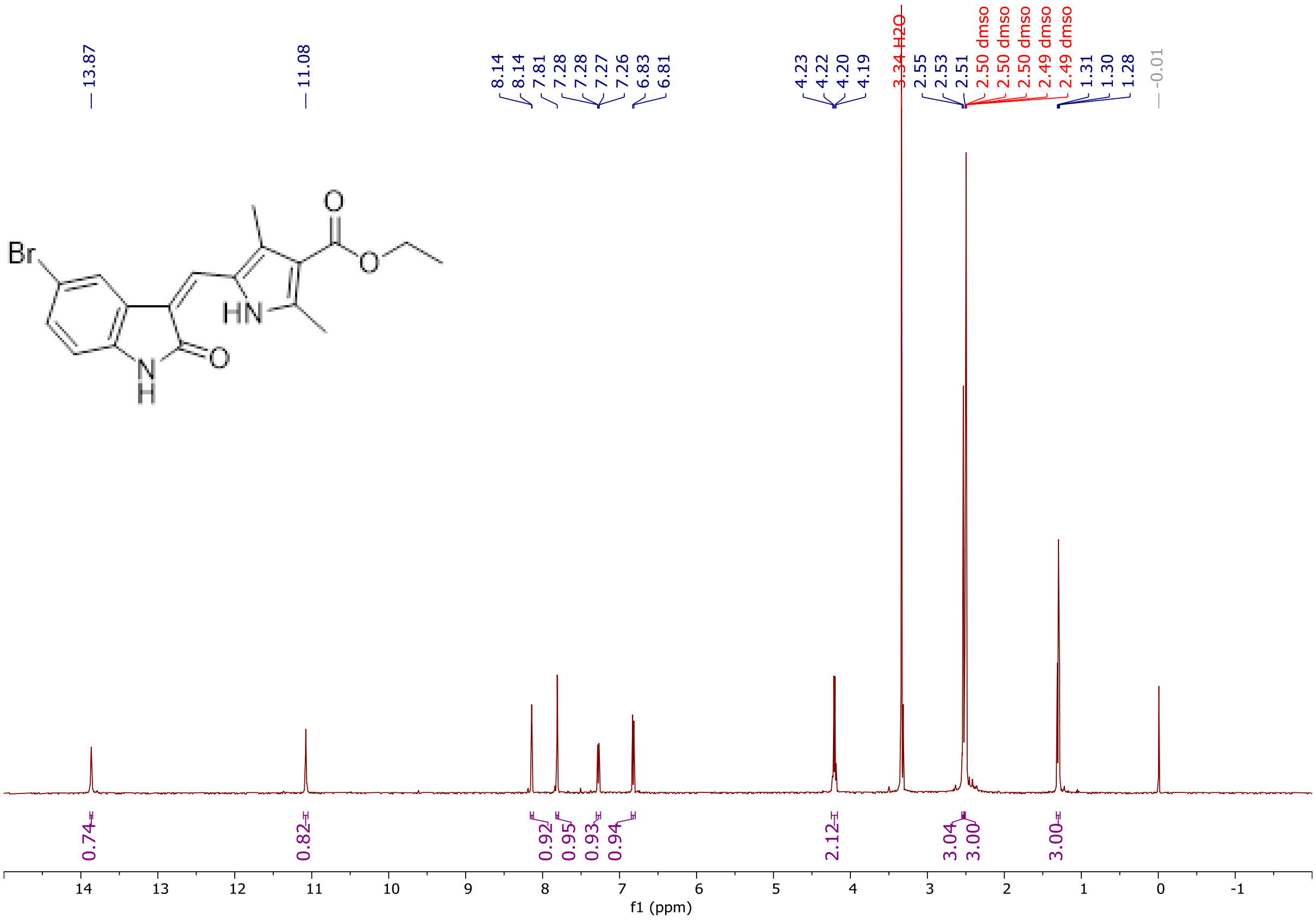

Spectrum ^{13}C NMR (125 MHz, DMSO-*d6*) of compound **8u**

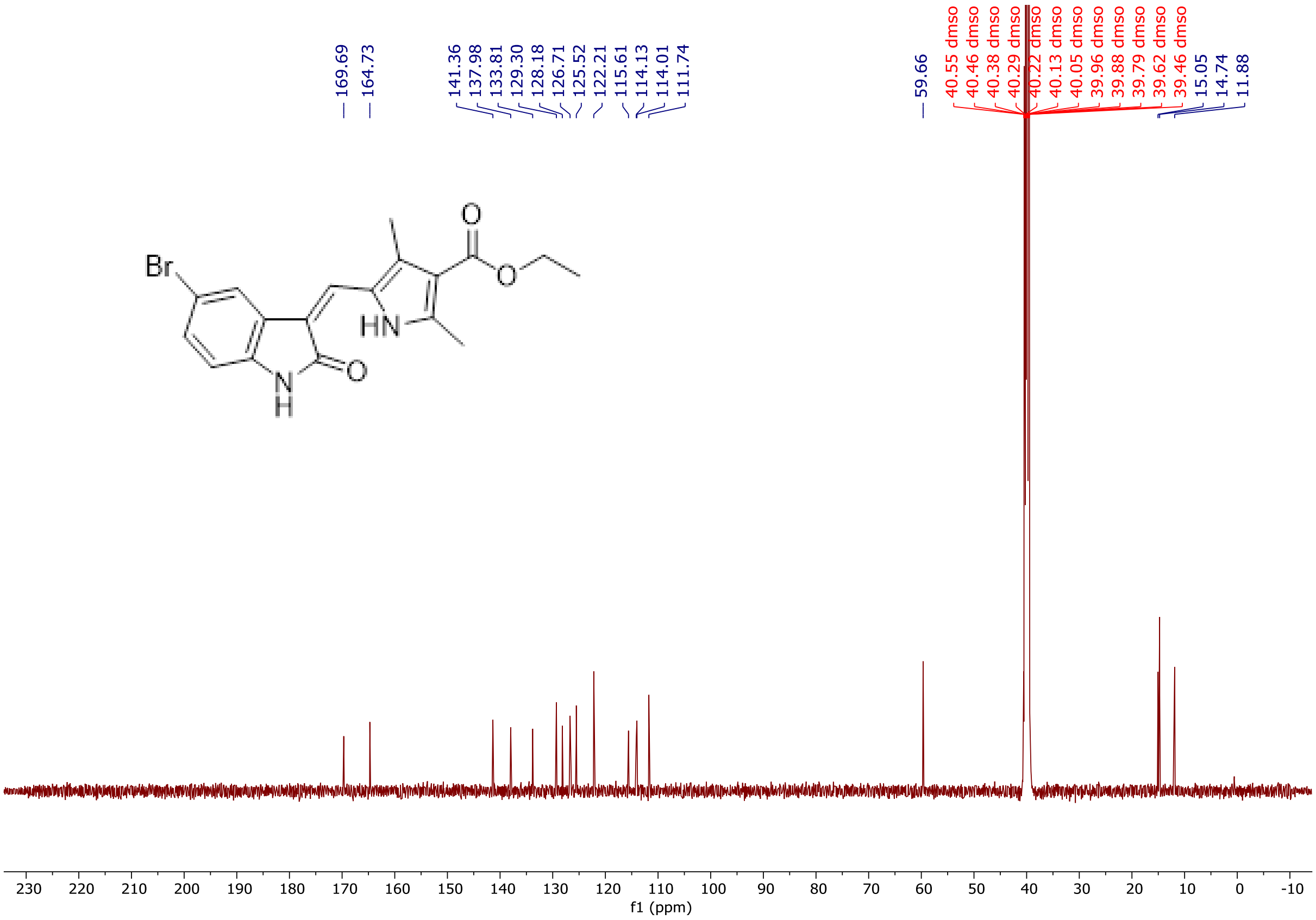

Spectrum ^{1}H NMR (500 MHz, DMSO-*d6*) of compound **8v**

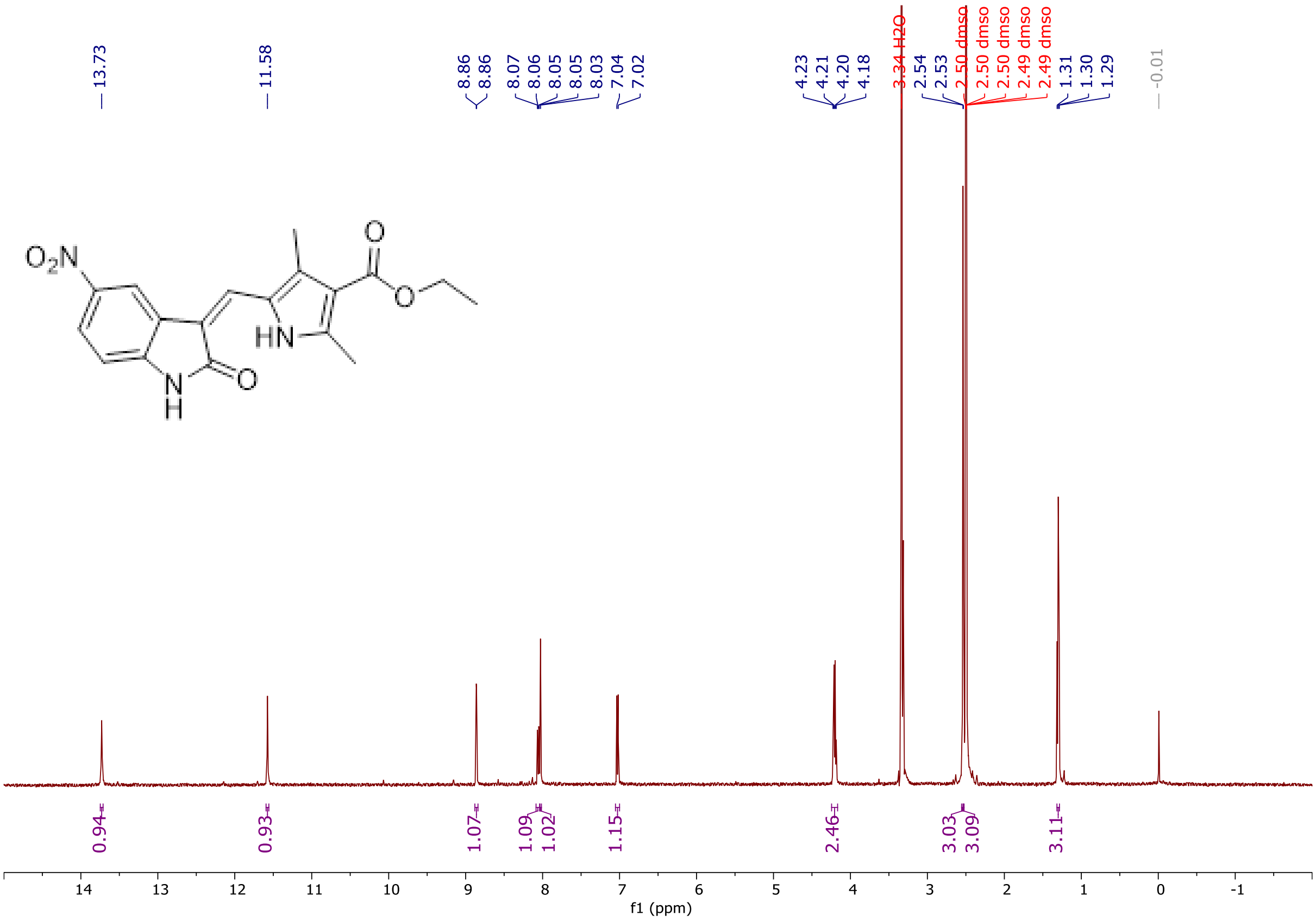

Spectrum ^{13}C NMR (125 MHz, DMSO-*d6*) of compound **8v**

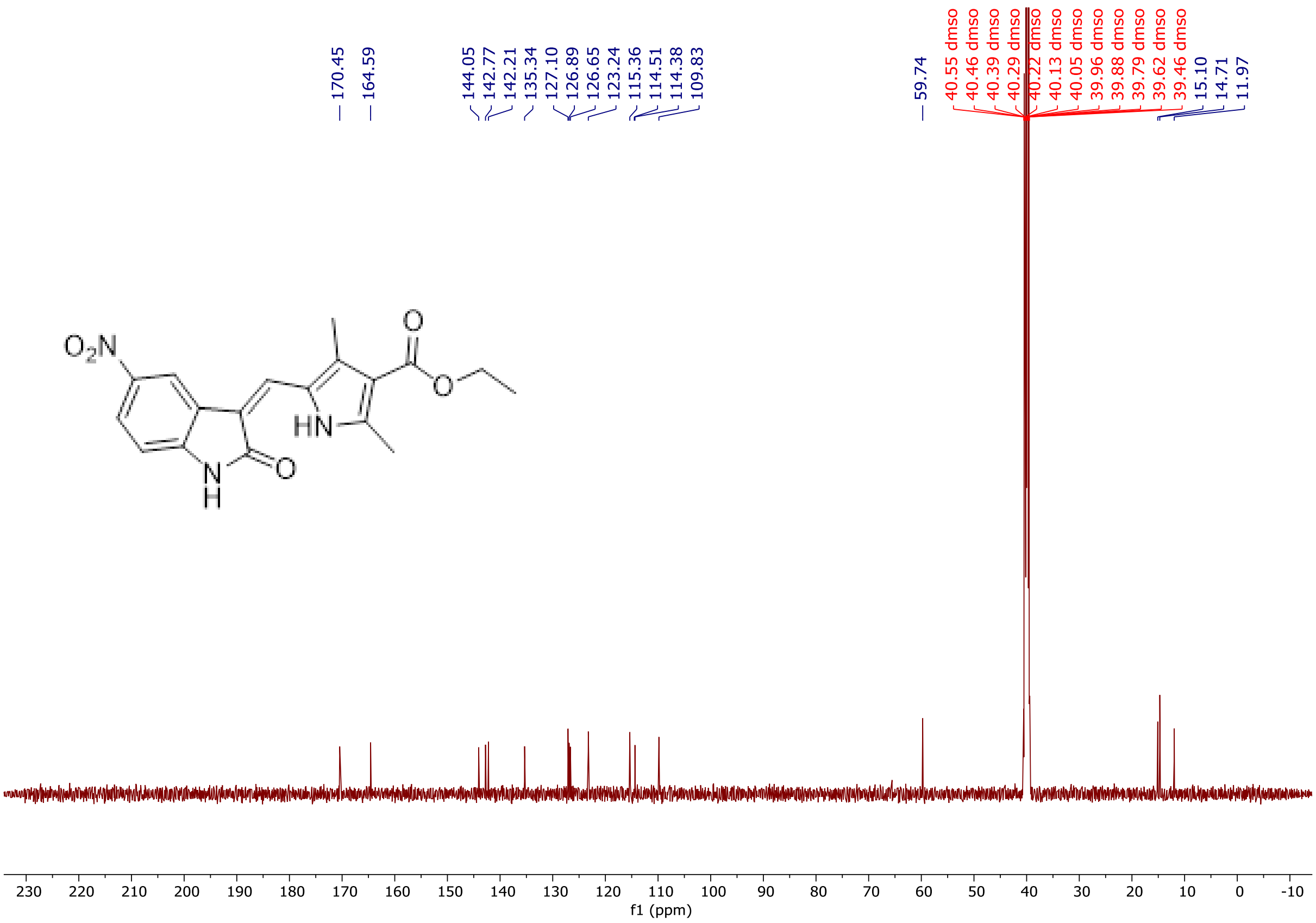

HRMS spectrum of compound **8v**

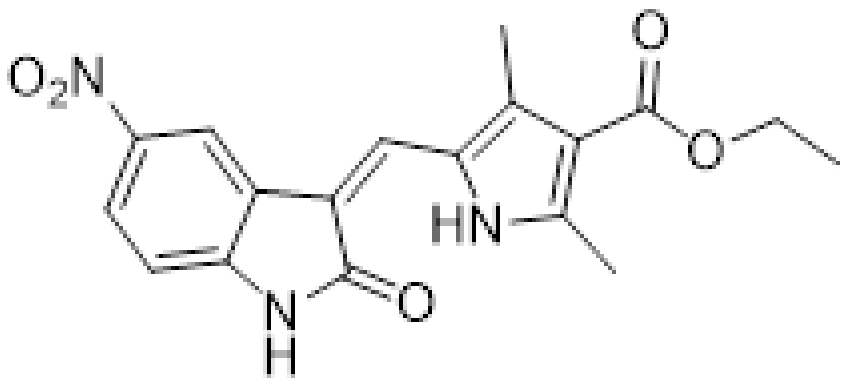

Single Mass Analysis
Tolerance = 5.0 PPM / DBE: min = -1.5, max = 50.0
Element prediction: Off
Number of isotope peaks used for i-FIT = 3

Monoisotopic Mass, Even Electron Ions
300 formula(e) evaluated with 1 results within limits (up to 50 best isotopic matches for each mass)
Elements Used:
C: 18-18 H: 0-100 N: 0-100 O: 0-100
Robby_RGN1_NEG 21 (0.340) Cm (17:22)
TOF MS ES-

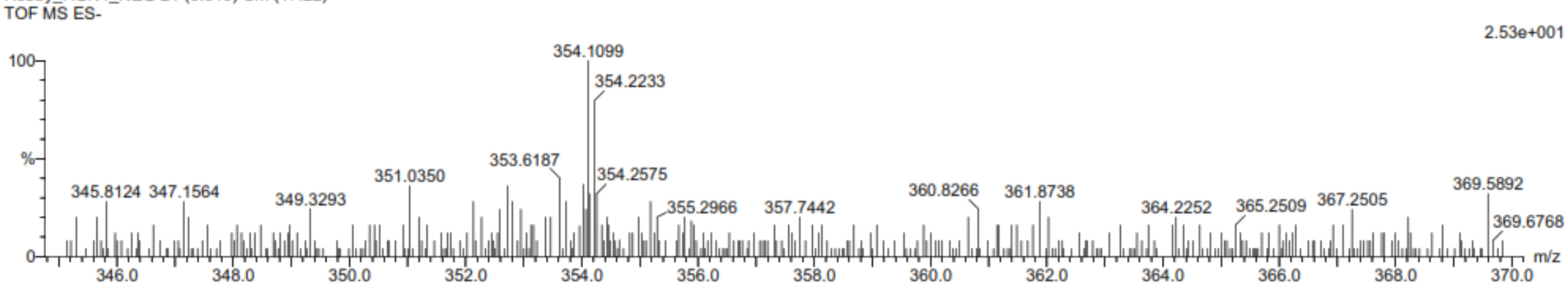

Minimum:				-1.5			
Maximum:		5.0	5.0	50.0			
Mass	Calc. Mass	mDa	PPM	DBE	i-FIT	i-FIT (Norm)	Formula
354.1099	354.1090	0.9	2.5	12.5	118.6	0.0	C18 H16 N3 O5

Spectrum ^{1}H NMR (500 MHz, DMSO-*d6*) of compound **8w**

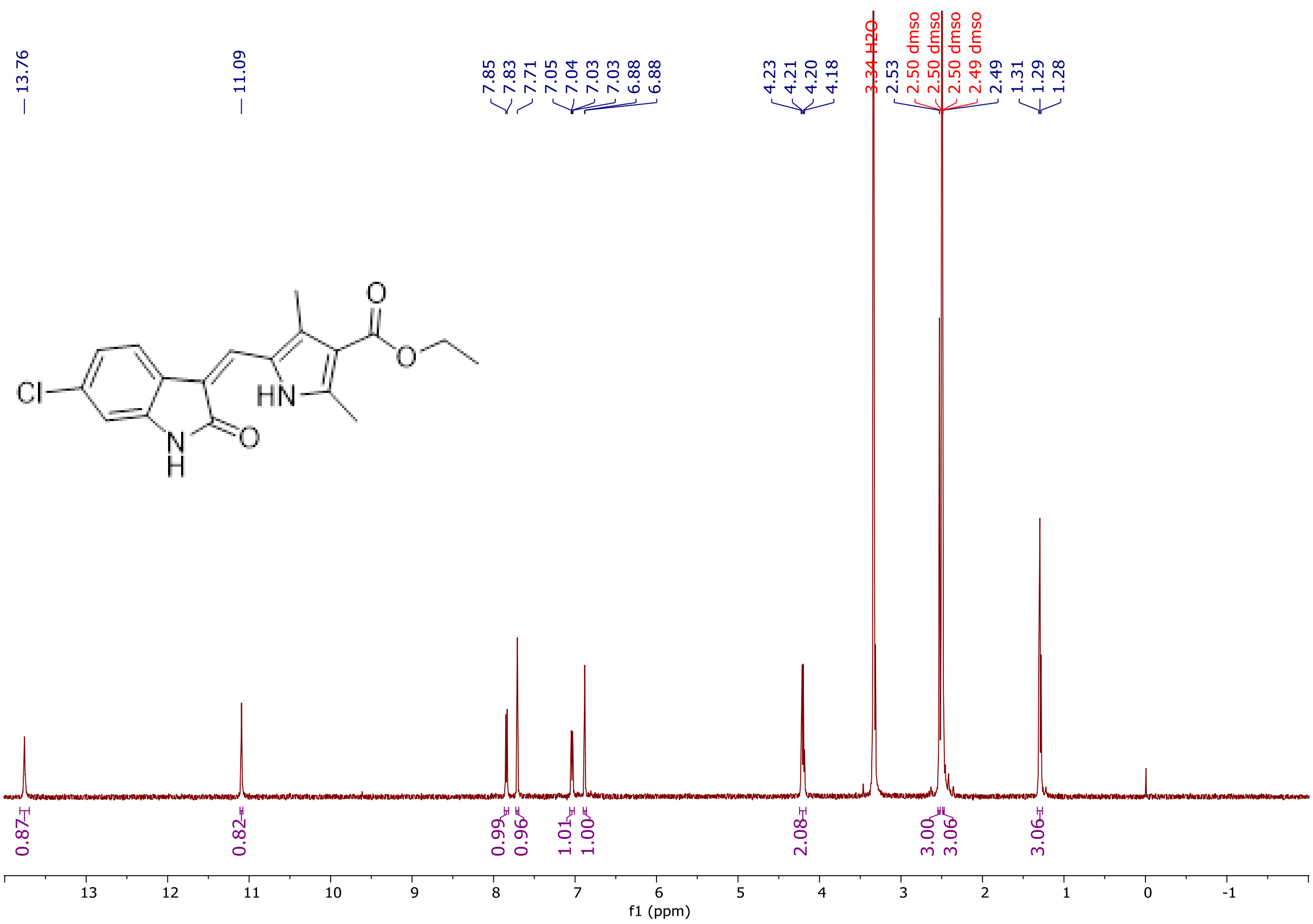

Spectrum ^{13}C NMR (125 MHz, DMSO-*d6*) of compound **8w**

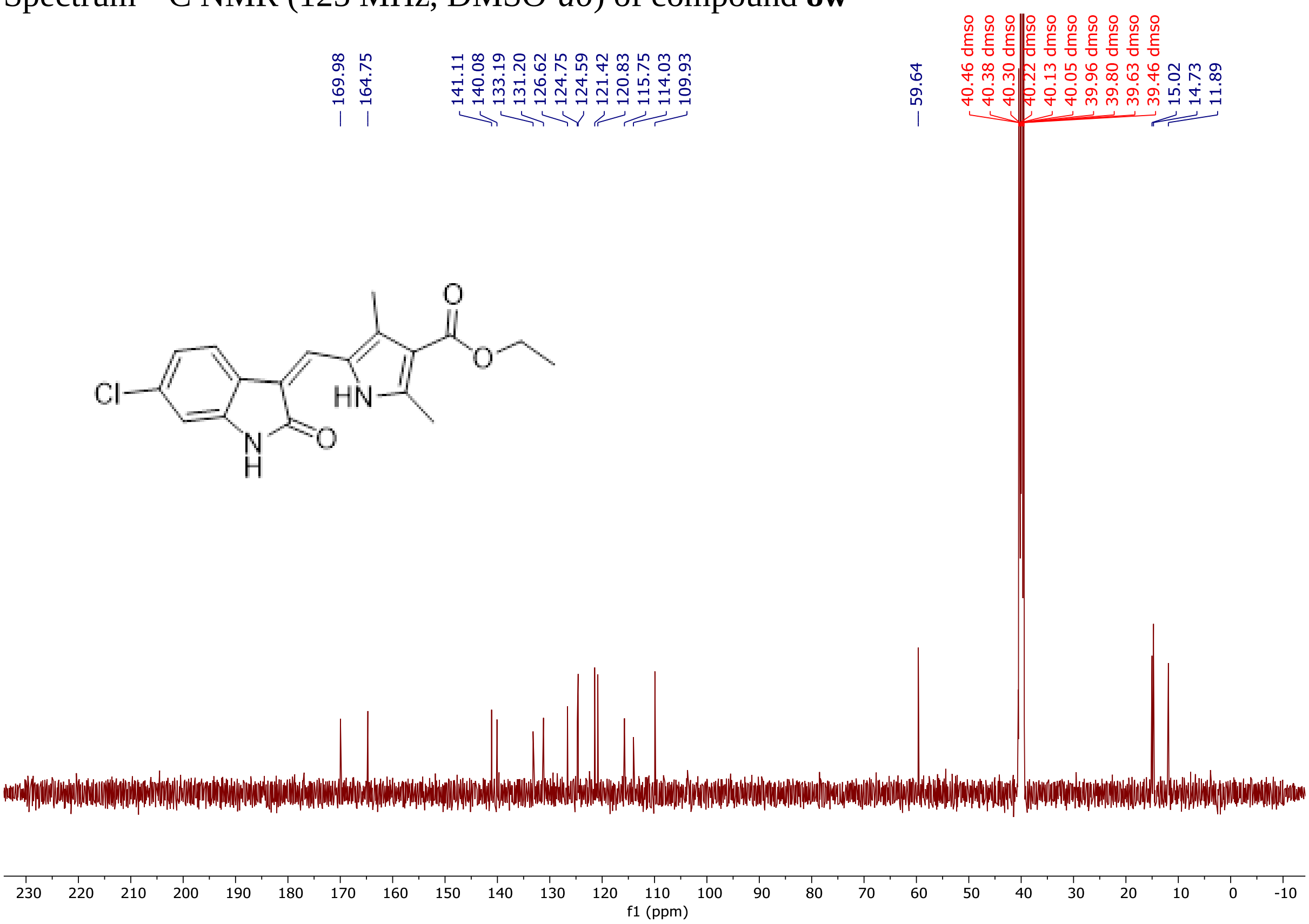

HRMS spectrum of compound **8w**

Single Mass Analysis
Tolerance = 5.0 PPM / DBE: min = -1.5, max = 50.0
Element prediction: Off
Number of isotope peaks used for i-FIT = 3

Monoisotopic Mass, Even Electron Ions
234 formula(e) evaluated with 1 results within limits (up to 50 best isotopic matches for each mass)
Elements Used:
C: 18-18 H: 0-100 N: 0-100 O: 0-100 Cl: 1-1
Robby_RGD2_pos 52 (0.870)
TOF MS ES+

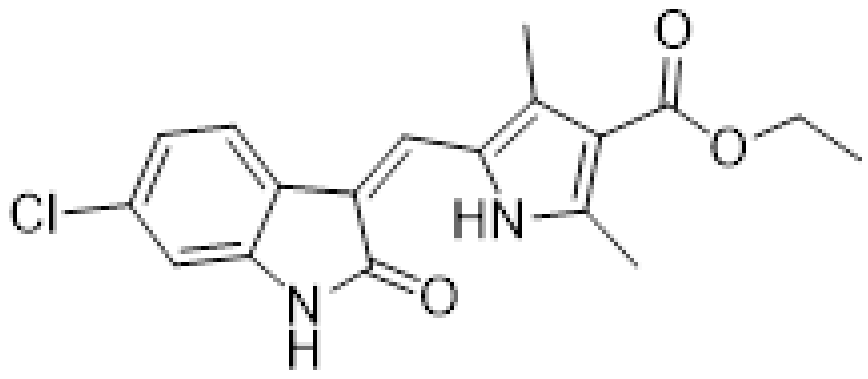

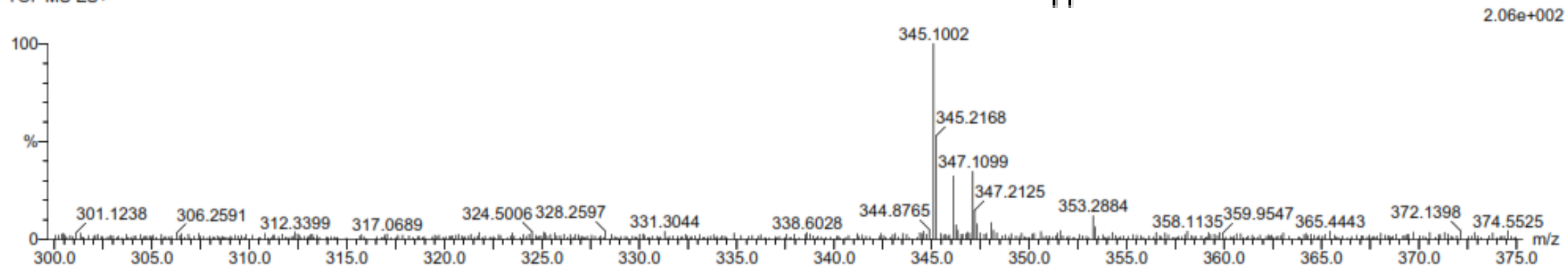

Minimum:				-1.5			
Maximum:		5.0	5.0	50.0			
Mass	Calc. Mass	mDa	PPM	DBE	i-FIT	i-FIT (Norm)	Formula
345.1002	345.1006	-0.4	-1.2	10.5	84.5	0.0	C18 H18 N2 O3 Cl

CheckCIF structure of **8r**

checkCIF/PLATON report

Structure factors have been supplied for datablock(s) mo_Robby_RGS8a_a

THIS REPORT IS FOR GUIDANCE ONLY. IF USED AS PART OF A REVIEW PROCEDURE FOR PUBLICATION, IT SHOULD NOT REPLACE THE EXPERTISE OF AN EXPERIENCED CRYSTALLOGRAPHIC REFEREE.

No syntax errors found. CIF dictionary Interpreting this report

Datablock: mo_Robby_RGS8a_a

Bond precision:	C-C = 0.0041 A		Wavelength=0.71073
Cell:	a=12.960(2)	b=12.426(2)	c=11.5398(17)
	alpha=90	beta=114.032(8)	gamma=90
Temperature:	100 K		

	Calculated	Reported
Volume	1697.3(5)	1697.2(5)
Space group	C c	C c
Hall group	C -2yc	C -2yc
Moiety formula	C20 H20 F N3 O3	?
Sum formula	C20 H20 F N3 O3	C20 H20 F N3 O3
Mr	369.39	369.39
Dx,g cm-3	1.446	1.446
Z	4	4
Mu (mm-1)	0.106	0.106
F000	776.0	776.0
F000'	776.39	
h,k,lmax	15,14,13	10,10,13
Nref	3011[1509]	3006
Tmin,Tmax	0.928,0.948	0.713,0.745
Tmin'	0.928	

Correction method= # Reported T Limits: Tmin=0.713 Tmax=0.745
AbsCorr = MULTI-SCAN

```
Data completeness= 1.99/1.00        Theta(max)= 25.027

                                                   wR2(reflections)=
R(reflections)= 0.0307( 2616)                      0.0864( 3006)

S = 0.617                  Npar= 246
```

```
The following ALERTS were generated. Each ALERT has the format
       test-name_ALERT_alert-type_alert-level.
Click on the hyperlinks for more details of the test.
```

Alert level C

```
GOODF01_ALERT_2_C  The least squares goodness of fit parameter lies
            outside the range 0.80 <> 2.00
            Goodness of fit given =      0.617
RINTA01_ALERT_3_C  The value of Rint is greater than 0.12
            Rint given   0.156
PLAT089_ALERT_3_C Poor Data / Parameter Ratio (Zmax <= 18) .......       6.14 Note
PLAT340_ALERT_3_C Low Bond Precision on  C-C Bonds ...............    0.00406 Ang.
PLAT911_ALERT_3_C Missing FCF Refl Between Thmin & STh/L=    0.596          2 Report
                4 14  1,   13  1  2,
```

Alert level G

```
PLAT007_ALERT_5_G Number of Unrefined Donor-H Atoms .............           2 Report
              H2      H3
PLAT020_ALERT_3_G The Value of Rint is Greater Than 0.12 .........      0.156 Report
PLAT032_ALERT_4_G Std. Uncertainty on Flack Parameter Value High .      0.400 Report
PLAT063_ALERT_4_G Crystal Size Possibly too Large for Beam Size ..       0.70 mm
PLAT398_ALERT_2_G Deviating  C-O-C   Angle From 120 for O1        .     109.7 Degree
PLAT432_ALERT_2_G Short Inter X...Y Contact  O3         ..C3      .      3.01 Ang.
                                     1/2+x,3/2-y,-1/2+z  =      4_564 Check
PLAT909_ALERT_3_G Percentage of I>2sig(I) Data at Theta(Max) Still        68% Note
PLAT950_ALERT_5_G Calculated (ThMax) and CIF-Reported Hmax Differ           5 Units
PLAT951_ALERT_5_G Calculated (ThMax) and CIF-Reported Kmax Differ           4 Units
PLAT953_ALERT_1_G Reported (CIF) and Actual (FCF) Hmax Differ by .          5 Units
PLAT954_ALERT_1_G Reported (CIF) and Actual (FCF) Kmax Differ by .          4 Units
PLAT969_ALERT_5_G The 'Henn et al.' R-Factor-gap value ..........       1.646 Note
              Predicted wR2: Based on SigI**2  5.25 or SHELX Weight 14.00
PLAT978_ALERT_2_G Number C-C Bonds with Positive Residual Density.          6 Info
```

```
   0 ALERT level A = Most likely a serious problem - resolve or explain
   0 ALERT level B = A potentially serious problem, consider carefully
   5 ALERT level C = Check. Ensure it is not caused by an omission or oversight
  13 ALERT level G = General information/check it is not something unexpected

   2 ALERT type 1 CIF construction/syntax error, inconsistent or missing data
   4 ALERT type 2 Indicator that the structure model may be wrong or deficient
   6 ALERT type 3 Indicator that the structure quality may be low
   2 ALERT type 4 Improvement, methodology, query or suggestion
   4 ALERT type 5 Informative message, check
```

It is advisable to attempt to resolve as many as possible of the alerts in all categories. Often the minor alerts point to easily fixed oversights, errors and omissions in your CIF or refinement strategy, so attention to these fine details can be worthwhile. It is up to the individual to critically assess their own results and, if necessary, seek expert advice.

PLATON version of 26/09/2025; check.def file version of 20/09/2025

Datablock mo_Robby_RGS8a_a - ellipsoid plot

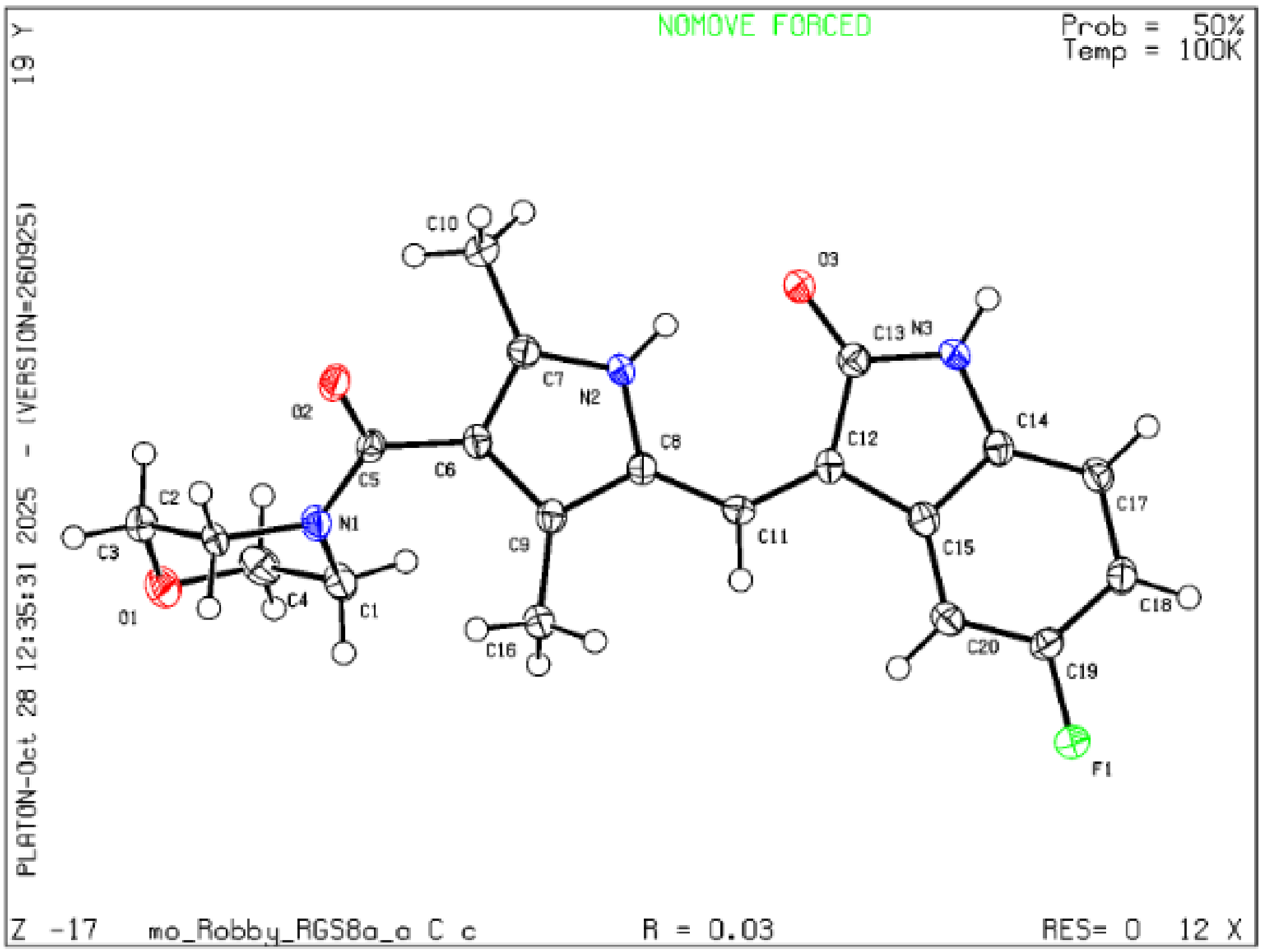

Supplement: Supplementary file 1 [file mmc1.pdf]
